# Identification of the Critical Life‐Stage of Obesity Contributing to Brain Functional Networks

**DOI:** 10.1111/cns.70510

**Published:** 2025-07-10

**Authors:** Pei Xiao, Yan Li, Jiayuan Dai, Jingfan Xiong, Jie Mi

**Affiliations:** ^1^ Center for Non‐Communicable Disease Management, Beijing Children's Hospital, Capital Medical University National Center for Children's Health Beijing China; ^2^ Department of Child and Adolescent Chronic Disease Prevention Shenzhen Center for Chronic Disease Control Shenzhen China; ^3^ Department of Rare Diseases, State Key Laboratory of Complex Severe and Rare Diseases, Peking Union Medical College Hospital Chinese Academy of Medical Science and Peking Union Medical College Beijing China

**Keywords:** body mass index, brain functional networks, life‐course, Mendelian randomization, obesity, transcriptome‐wide association study

## Abstract

**Aims:**

Observational studies suggest that obesity impacts brain functional connectivity, but critical developmental periods for these effects remain unclear. Herein, we aimed to investigate the causal relationships between life‐course body weight and brain functional connectivity.

**Methods:**

Mendelian randomization (MR) was applied to infer the causality between life‐course body weight (birth weight [*n* = 80,745], childhood body mass index [BMI; *n* = 39,620], and adulthood BMI [*n* = 322,154]) and 191 resting‐state functional magnetic resonance imaging traits (*n* = 34,691) using genome‐wide association data. Linkage disequilibrium score regression and colocalization analysis were conducted to reinforce the causality. Two‐step mediation MR, transcriptome‐wide association studies, and enrichment analyses were performed to explore the underlying mechanisms.

**Results:**

Adulthood BMI increased neural activity in the frontal lobe (*β* = 0.078, 95% CI: 0.029 ~ 0.127), whereas childhood BMI reduced functional connectivity between the subcortical‐cerebellum and motor or attention network (*β* = −0.087, 95% CI: −0.144 ~ −0.031). Birth weight decreased the functional connectivity of the central executive or default mode network in the temporal lobe (*β* = −0.147, 95% CI: −0.217 ~ −0.078). These causalities were consistent with the MR sensitivity analyses and colocalization results. The mediation MR identified neurexophilin‐3 as a potential mediator of the causal effect of birth weight on functional connectivity, explaining 27.3% of the total effect (95% CI: 2.6%–52.0%, *p* = 0.048). Furthermore, transcriptional analysis revealed prioritized genes and pathways that interconnect body weight at different life stages and brain functional connectivity.

**Conclusion:**

This study demonstrated distinct life‐stage‐specific effects of body weight on brain functional networks, highlighting the need for targeted interventions across the life course to mitigate the persistent effect of early‐life obesity on brain health.

## Graphical abstract

This integrative genetic study, using Mendelian randomization and transcriptome‐wide association analyses, demonstrates that birth weight decreases functional connectivity of the central executive or default mode network in the temporal lobe, childhood BMI reduces connectivity in subcortical‐cerebellum and motor/attention networks, and adulthood BMI increases neural activity in the frontal lobe.

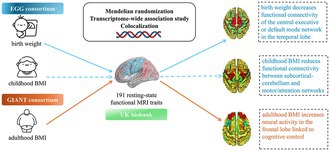

## 1 Introduction

The human brain operates as an intricate system in which the coordination and interaction of functional networks are fundamental to supporting behavior and cognitive processes [1]. Obesity is increasingly recognized as a complex health issue that not only affects physical health [2, 3] but also exerts profound implications for behavior and cognitive function [4, 5]. These effects are closely linked to significant alterations in brain function and structure, as highlighted in recent studies [6, 7, 8]. Despite this growing understanding, evidence on how obesity influences brain functional network characteristics remains limited. In particular, the critical life stage during which body weight exerts the greatest influence on specific brain functional networks remains largely unclear and poorly explored. Hence, there is a pressing need to identify this pivotal stage to guide targeted interventions aimed at mitigating the adverse neurobiological effects of obesity and improving brain health outcomes.

Resting‐state functional magnetic resonance imaging (rsfMRI), which measures intrinsic brain connectivity and activity utilizing alterations in blood oxygen level‐dependent signals, has become a powerful tool for understanding brain functional networks [9]. A variety of resting‐state networks (RSNs) within the neurotypical human brain have been identified by rsfMRI, including the default mode, central executive, salience, limbic, attention, et al., each contributing distinct roles in brain function and connectivity [10]. Growing evidence indicates that dynamic interactions and functional organization of brain networks are essential for the progression of various psychiatric disorders. For example, alterations in RSNs have been widely observed across psychiatric and neurological conditions, including schizophrenia [11], major depressive disorder [12], and Alzheimer's disease [13], compared with neurotypical controls. On the other hand, it remains a serious problem with the high prevalence of psychiatric disorders and comorbid obesity [14, 15]. However, the relationship between obesity and changes in brain functional connectivity networks remains underexplored, leaving gaps in our understanding of how obesity may influence mental health risk.

A recent longitudinal, multisite study involving over 45,000 participants examined the bidirectional relationship between obesity and brain structure measured by MRI across the lifespan and demonstrated an inverse association between body mass index (BMI) and reduced cortical thickness within fronto‐temporal regions [16]. Another cross‐sectional study highlighted the increased dysconnectivity of the anterior cingulate cortex and hippocampus with fronto‐limbic reward networks observed in overweight youth exhibiting increased levels of insulin resistance [17]. The observational data also revealed a positive association between BMI and white matter hyperintensity volume. However, these findings are often limited by observational designs, residual confounding, a lack of sufficiently comprehensive brain functional assessment, and the inability to infer causality. In addition, to date, no research has dissected the temporally distinct effects of body weight on brain functional networks, highlighting a critical gap in understanding the dynamic interplay between them over time. Utilizing genetic variants as instrumental variables (IVs), Mendelian randomization (MR) [18] has revolutionized the ability to infer causal relationships between biological phenotypes in recent years. In particular, multivariable MR (MVMR) has emerged as an extension of MR and has been increasingly applied in life‐course epidemiology [19, 20, 21]. This approach has proven effective in disentangling the temporally distinct effects of obesity at different life stages on outcomes later in life, providing valuable insights into complex causal relationships. Transcriptome‐wide association study (TWAS), another post genome‐wide association study (GWAS) analytic approach, has been applied to identify candidate tissue‐specific causal gene expression involved in traits and diseases [22]. This method provides a valuable framework for investigating the underlying mechanisms between obesity across the lifespan and functional connectivity in distinct brain regions.

By leveraging these advanced methods, we aimed to address the aforementioned gaps by employing an integrative genetic analytic framework to unravel the distinct causalities between life‐course body weight (including adulthood BMI, childhood BMI, and birth weight) and 191 rsfMRI phenotypes. To further elucidate potential pathological mechanisms and enhance the robustness of the findings, we conducted TWAS, colocalization, and enrichment analyses to identify the gene expression profiles and biological pathways underlying the observed causal associations.

## 2 Methods

### 2.1 Study Design

**FIGURE 1:**
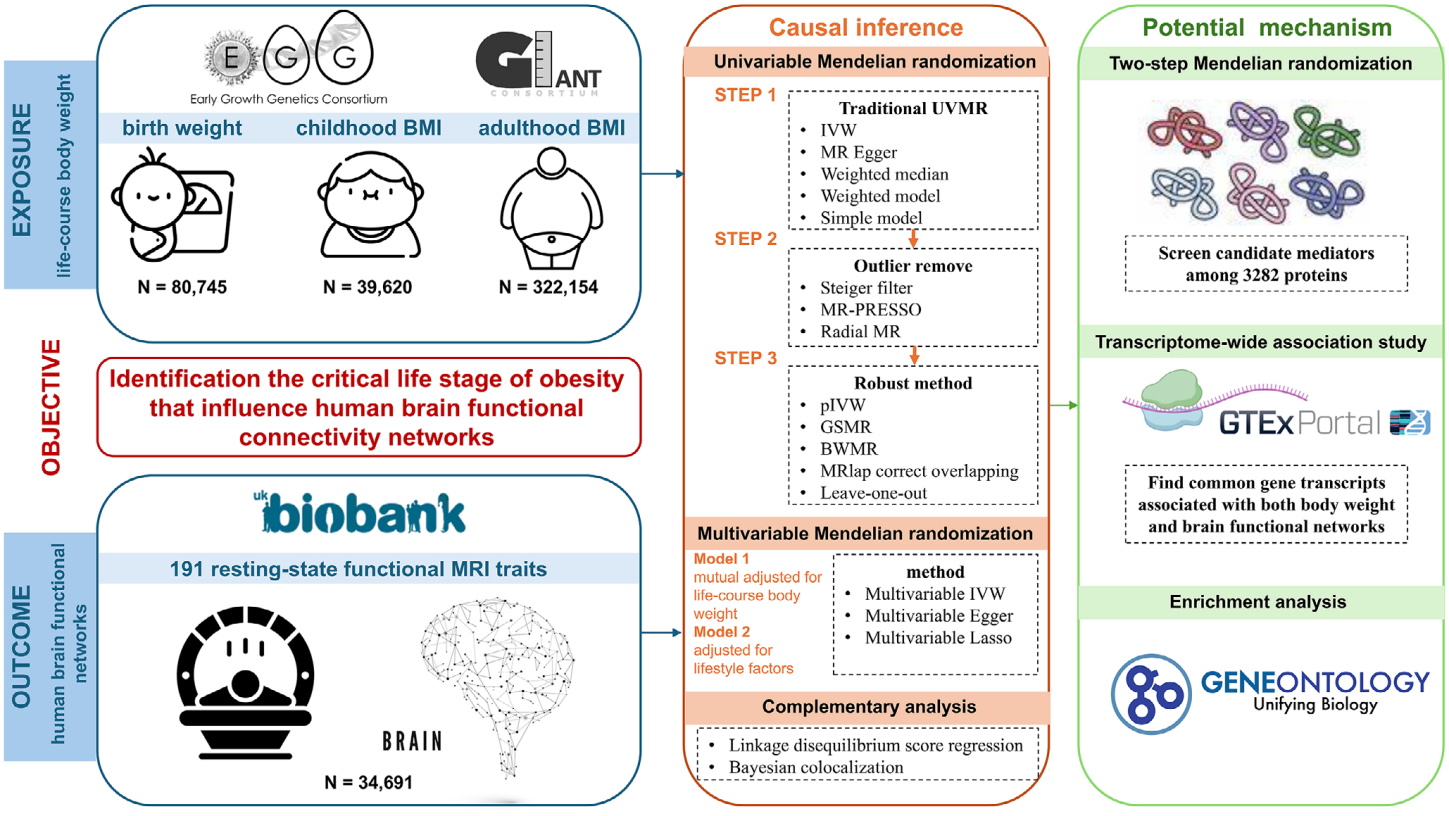
Study design.

Our study adheres to the Strengthening the Reporting of Observational Studies in Epidemiology—Mendelian Randomization reporting guidelines (STROBE‐MR) (Supporting Information) [23]. The study design is illustrated in Figure 1. We limited the study population exclusively to European ancestry participants to minimize bias due to population stratification (Table S1).

The summary‐level GWAS data of childhood BMI [24] and birth weight [25] were sourced from the Early Growth Genetics (EGG) consortium. Briefly, genetic associations with birth weight were obtained from a GWAS meta‐analysis of 298,142 individuals, where birth weight measurements were standardized as *Z* scores and adjusted for gestational age, sex, and study‐specific covariates in the original analysis to ensure consistency and accuracy [25]. To avoid potential sample overlap bias, we used a subset of the data excluding individuals from the UK Biobank, resulting in a final sample size of 80,745 individuals for our analysis. Summary‐level data on childhood BMI were obtained from a GWAS meta‐analysis of up to 39,620 European ancestry children aged 2–10 years, with BMI standardized as age‐ and sex‐adjusted *Z* scores based on the same external reference [24]. For adulthood BMI, we used previously published BMI GWAS summary data from the Genetic Investigation of Anthropometric Traits (GIANT) consortium, which included approximately 322,154 individuals of European ancestry, as reported by Locke et al. [26]. This dataset was chosen to avoid sample overlap with the UK Biobank.

The summary‐level genetic data for outcomes were sourced from a previous GWAS conducted in the UK Biobank (*n* = 34,691) by Zhao et al. [9]. They initially analyzed 1777 intrinsic brain activity phenotypes measured by rsfMRI, encompassing pairwise functional connectivity (edges), regional neural activity (nodes), and global functional connectivity. Due to the relatively weaker genetic effects on brain functional networks than structural traits, only phenotypes significantly associated with genetic variants (*p* < 5 × 10^−8^) were retained, resulting in 191 traits for GWAS analysis. These included 75 node amplitude traits reflecting spontaneous neural activity, 111 pairwise functional connectivity measures quantifying node coactivity, and 5 global functional connectivity traits summarizing whole‐brain functional connectivity. Table S2 details the assigned locations and associated functional networks. These phenotypes were mapped onto major functional networks, including central executive, default mode, somatomotor, attention, salience, limbic, and visual networks.

### 2.2 Genetic Instruments Selection

IVs used in MR analysis must satisfy three key principles: be strongly associated with the exposure, remain independent of confounders, and influence the outcome exclusively through the exposure. We selected single nucleotide polymorphisms (SNPs) at genome‐wide significance (*p* < 5 × 10^−8^), followed by linkage disequilibrium (LD) clumping (*r* ^2^ < 0.001) to ensure independence among IVs. The alleles were subsequently harmonized across datasets. The strength, heterogeneity, and horizontal pleiotropy of IVs were evaluated by the *F* statistic, Cochran's *Q* test, and MR‐Egger regression intercept, respectively. As illustrated in Figure 1, in step 2, we used Steiger filtering to exclude IVs potentially inducing reverse causality. We then utilized the MR pleiotropy residual sum and outlier test (MR‐PRESSO) and Radial MR to detect and remove IVs exhibiting heterogeneity or pleiotropy. Following this, a set of robust MR methods was performed in step 3 to test the robustness of the findings.

### 2.3 Two‐Sample MR Analysis

Within the life‐course MR analytic framework, we first applied univariable MR (UVMR) to assess the total effects of body weight at birth, childhood, and adulthood on rsfMRI phenotypes. Subsequently, we utilized MVMR to estimate the independent effects of body weight at each life stage on brain functional networks, accounting for mutual adjustment across life stages and controlling for potential lifestyle risk factors. Finally, a two‐step MR analysis was performed to identify potential mediators of these associations, utilizing protein quantitative trait locus (pQTL) data for up to 3282 proteins derived from a human plasma proteome GWAS involving 3301 participants of European ancestry [27]. The summary‐level data of plasma proteome were obtained from the INTERVAL study conducted by Sun BB [27], which was available on the integrative epidemiology unit open GWAS project (https://gwas.mrcieu.ac.uk, GWAS ID from prot‐a‐1 to prot‐a‐3282).

#### 2.3.1 UVMR

In UVMR (step 1), the random‐effects inverse‐variance weighted (IVW) method was employed as the primary analytical approach. *p* Values were corrected using the Benjamini‐Hochberg (BH) method with a false discovery rate threshold of 0.10 to determine significance. To address potential pleiotropy, complementary sensitivity analyses were conducted using four additional MR methods: the MR‐Egger, weighted median, simple mode, and weighted mode. After removing outliers as previously described (step 2), we applied a series of robust MR methods (step 3). The penalized IVW (pIVW) method extends traditional IVW by addressing biases from weak instrumental variables and horizontal pleiotropy. The generalized summary‐data‐based MR (GSMR) method utilizes GWAS summary statistics and a multi‐SNP model to test for causal associations between risk factors and diseases, offering flexibility, robustness against environmental confounding, and wide applicability across various fields [28]. The Bayesian weighted MR (BWMR) method addresses challenges in causal inference from GWAS data by accounting for polygenic effects and pleiotropy through Bayesian weighting and outlier detection, offering computational efficiency and improved accuracy via a variational expectation–maximization algorithm and posterior covariance correction [29]. Additionally, we used the MRlap method to address biases from sample overlap, weak instruments, and Winner's curse by integrating cross‐trait linkage disequilibrium score (LDSC) regression to approximate overlap, relying solely on GWAS summary statistics, and providing corrected effect estimates [30]. Finally, a leave‐one‐out analysis was carried out to test whether a single SNP drove the association.

#### 2.3.2 MVMR

Considering the irreversible temporal influence of adiposity across different life stages, we further employed MVMR to estimate the life‐stage‐specific effects of obesity on rsfMRI traits. First, we simultaneously included adulthood BMI, childhood BMI, and birth weight in Model 1 to disentangle the distinct causal effects of body weight at different life stages on rsfMRI traits. Additionally, we conducted MVMR analyses in Model 2, incorporating body weight at each life stage alongside potential lifestyle confounders (alcohol consumption, GWAS ID: ieu‐b‐73; smoking, GWAS ID: ukb‐b‐223; sleep duration, GWAS ID: ukb‐b‐4424; physical activity, GWAS ID: ebi‐a‐GCST006097). The direct causal effects between traits were calculated using the multivariable IVW, Egger, and LASSO methods.

#### 2.3.3 Mediation MR

We also used two‐step mediation MR analysis to explore potential pathways mediated by proteins in the association between life‐course body weight and brain functional networks. In the first step, we assessed the causal effect of the exposure on the mediator using UVMR. In the second step, we evaluated the causal effect of the mediator on the outcome with adjustment of exposure using MVMR. The indirect effect was estimated by multiplying the exposure's causal effect on the mediator with the mediator's effect on the outcome. The proportion of mediation effect was estimated as the ratio of the indirect effect to the total effect, with standard errors computed using the delta method.

### 2.4 Cross‐Trait LDSC Regression

To investigate the genetic correlation (*r*_g_) between life‐course body weight and rsfMRI traits identified as significant in prior UVMR analyses, we applied cross‐trait LDSC regression leveraging GWAS summary statistics. This approach allowed us to estimate SNP‐based heritability (ℎ^2^) for each trait and coheritability between traits. The *p* values for genetic correlation were adjusted for multiple testing using the Bonferroni correction. The analysis ensured alignment with the European ancestry of the GWAS samples by utilizing the LD reference panel from the 1000 Genomes Project.

### 2.5 TWAS

We employed the FUSION method for TWAS to identify genes whose expression was significantly associated with both life‐course body weight and rsfMRI traits. FUSION utilizes expression quantitative trait locus (eQTL) data to construct predictive models of gene expression, which are then integrated with GWAS summary statistics. For this study, we conducted TWAS using transcriptomic reference data from Genotype‐Tissue Expression version 8 (GTEx v8), focusing specifically on brain tissues relevant to the associated functional connectivity. Fisher's combined *p* value (FCP) method was used to integrate *p* values for body weight and rsfMRI traits. To further explore the potential mechanisms underlying the observed causal relationships, we performed Gene Ontology (GO) enrichment analysis on the co‐associated expressed genes to identify relevant biological pathways.

### 2.6 Colocalization Analysis

We reinforced the MR findings using Bayesian colocalization analyses, which can identify shared causal variants between traits. Specifically, we examined the overlap of genetic signals between GWASs for life‐course body weight and rsfMRI traits, estimating the posterior probability that a single causal variant influences both traits within the same genomic region. Additionally, we integrated SNPs within 1 Mb of the cis‐expression eQTL data with GWAS summary statistics to identify genetic variants that may simultaneously regulate gene expression and impact life‐course body weight and rsfMRI traits. The eQTL data were extracted from GTEx v8, with a focus on brain tissues relevant to the rsfMRI phenotypes. We used a posterior probability (PP.H4) of 0.8 as our threshold for strong evidence of colocalization, which represents a stringent but commonly used threshold in colocalization analyses that balances sensitivity and specificity [31].

### 2.7 Statistical Analysis

All MR, LDSC, and colocalization analyses were performed using R software (v 4.2.2) with the following packages: ldscr (0.1.0), TwoSampleMR (v 0.5.10), MVMR (v 0.4), RadialMR (v 1.1), mr.pivw (v 0.1.1), gsmr (v 1.0.9), BWMR (v 0.1.1), MRPRESSO (v 1.0), MRlap (v 0.0.3.0), and coloc (v 5.1.0.1). TWAS analyses were conducted using the FUSION software and database (http://gusevlab.org/projects/fusion/). Statistical significance was determined using a two‐tailed *p* value threshold of 0.05. For the primary UVMR analysis (IVW method), BH correction was applied, with a significance threshold of 0.10. The IVW estimates were deemed causal only if they passed the BH correction and were aligned in direction and significance with at least one sensitivity analysis and one robust method.

### 2.8 Ethics Statement

This study utilized publicly accessible summary‐level datasets from previously ethically approved GWASs (Table S1), eliminating the need for further ethical approval.

## 3 Results

### 3.1 IVs Selection

A total of 42, 16, and 448 SNPs were identified for birth weight (explaining 1.5% of the variance; *F* statistic = 55.8), childhood BMI (explaining 2.2% of the variance; *F* statistic =52.8), and adulthood BMI (explaining 4.7% of the variance; *F* statistic =71.6) in step 1 of the UVMR analysis, respectively. The *F* statistics for all individual IVs ranged from 28.6 to 822.8, indicating no evidence of weak instrument bias.

### 3.2 Birth Weight and Brain Functional Networks

**FIGURE 2:**
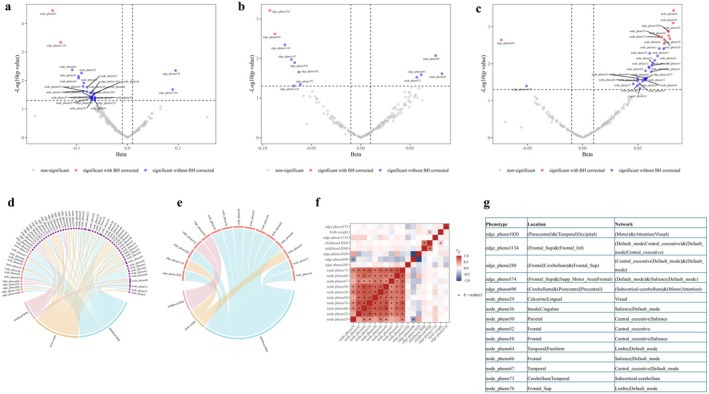
Findings from the primary univariable Mendelian randomization and cross‐trait linkage disequilibrium score regression analysis. The volcano plot displays the IVW effect size of birth weight (a), childhood BMI (b), and adulthood BMI (c) on the *x*‐axis, with the *y*‐axis representing the −log10 transformed *p* values. The chord diagram illustrates the correlation between life‐course body weight and brain functional connectivity traits before (d) and after (e) Benjamini‐Hochberg correction. Purple indicates outcome traits with a *p* value < 0.05 that are not significant after correction, whereas pink represents traits that remain significant after correction. The heatmap illustrates the genetic correlations across all GWAS summary statistics for exposures and outcomes that were found to be significant (*p* < 0.05) in the primary UVMR analyses (f). The table lists the brain location and functional network for each phenotype ID (g).

**FIGURE 3:**
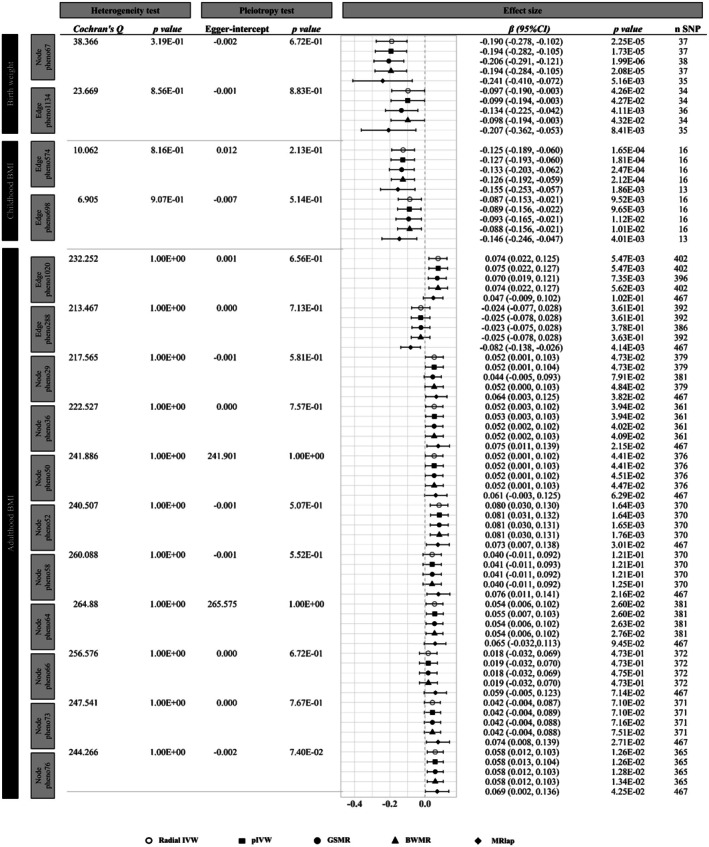
Robust univariable Mendelian randomization estimates for the total causal effect of life‐course body weight on brain function connectivity after outlier removal.

The volcano plot (Figure 2a) illustrates the causal relationships between birth weight and 191 rsfMRI traits identified using the IVW method in step 1 of the UVMR analysis. Among these, 28 rsfMRI traits (21 amplitude traits and 7 pairwise functional connectivities) were significantly associated with birth weight (*p* < 0.05), with node_pheno67 and edge_pheno1134 remaining significant after BH correction. The location and network of these rsfMRI traits are presented in Figure 2g. A genetically predicted 1‐SD increase in birth weight was significantly associated with a 0.131‐SD decrease in functional connectivity between the default model network (DMN) and central executive network (CEN) (*β* = −0.131, 95% confidence interval [CI]: −0.205 ~ −0.058, BH corrected *p* = 0.044; edge_pheno1134) (Table S3). Besides, genetically determinant birth weight was inversely associated with neural activity in the temporal lobe (*β* = −0.147, 95% CI: −0.217 ~ −0.078, BH corrected *p* = 0.007; node_pheno67). The causal associations of birth weight with edge_pheno1134 and node_pheno67 identified in the primary analysis were supported by at least one traditional sensitivity analysis (Table S4). The MR‐Egger intercepts were close to zero (edge_pheno1134: *p* = 5.17E‐02; node_pheno67: *p* = 7.48E‐01), indicating no evidence of substantial unbalanced horizontal pleiotropy (Table S5). However, the Cochran *Q* statistic revealed some heterogeneity in the instrument–exposure associations (Table S5). Using the methods described earlier, we removed outliers and performed robust MR analyses focusing on the two observed causal effects. Heterogeneity among the IVs was effectively addressed, and all robust MR analyses consistently supported the findings of the primary analysis (Figure 3 and Figure S1).

**FIGURE 4:**
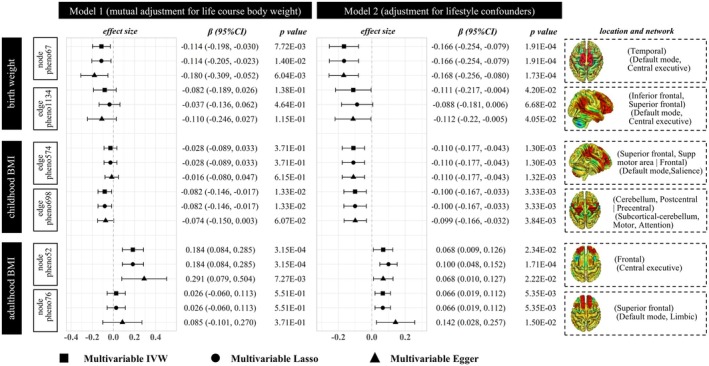
Multivariable Mendelian randomization estimates for the direct causal effects of body weight at each life‐stage on brain function connectivity. Model 1 included all life‐course body weight traits (birth weight, childhood BMI, and adulthood BMI). Model 2 was further adjusted for potential lifestyle factors, including current tobacco smoking, alcoholic drinks per week, sleep duration, and moderate to vigorous physical activity levels.

When modeled together with body weight at different life stages in the MVMR analyses (model 1), there was strong evidence of a direct causal effect of birth weight on neural activity in the temporal lobe (multivariable IVW *β* = −0.114, 95% CI: −0.198 ~ −0.030, *p* = 7.72E‐03; node_pheno67), independent of childhood and adulthood BMI (Figure 4). This causal association remained significant after further adjustment for lifestyle factors in model 2. However, for the functional connectivity between the DMN and CEN (edge_pheno1134), the causal effect was attenuated to null (multivariable IVW *β* = −0.082, 95% CI: −0.189 ~ −0.026, *p* = 1.38E‐01) after accounting for BMI later in life. Given the robust causal relationship between birth weight and node_pheno67, mediation MR was performed to identify potential mediating proteins using proteomics GWAS data. Among the 19 proteins associated with both the exposure and the outcome (Table S6), only neurexophilin‐3 significantly mediated the effects, accounting for 27.3% of the total effect (95% CI: 2.6 ~ 52.0%, *p* = 0.048).

**FIGURE 5:**
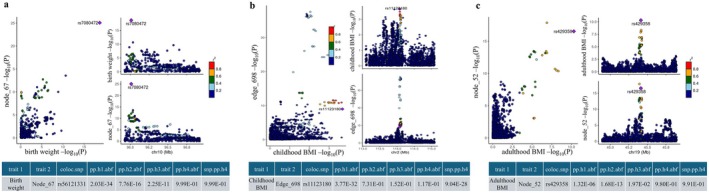
Colocalization analyses between GWASs of life‐course body weight and GWASs of brain functional connectivity traits. (a) LocusCompare plot comparing GWAS for birth weight and GWAS for node_pheno67; (b) LocusCompare plot comparing GWAS for childhood BMI and GWAS for edge_pheno698; (c) LocusCompare plot comparing GWAS for adulthood BMI and GWAS for node_pheno52.

A positive genetic correlation was identified between birth weight and childhood BMI (*r*_g_ = 0.22, *p* = 8.31E‐08) in the cross‐trait LDSC (Figure 1f and Table S7). However, no significant genetic correlation was observed between birth weight and rsfMRI traits. The LDSC intercepts for all investigated traits were close to 1, suggesting no evidence of population stratification (Table S8). Strong evidence of colocalization was observed between birth weight and neural activity in the temporal lobe at rs7080472 (SNP.PP.H4 = 0.99; Figure 5a), further supporting the causal association between birth weight and node_pheno67.

**FIGURE 6:**
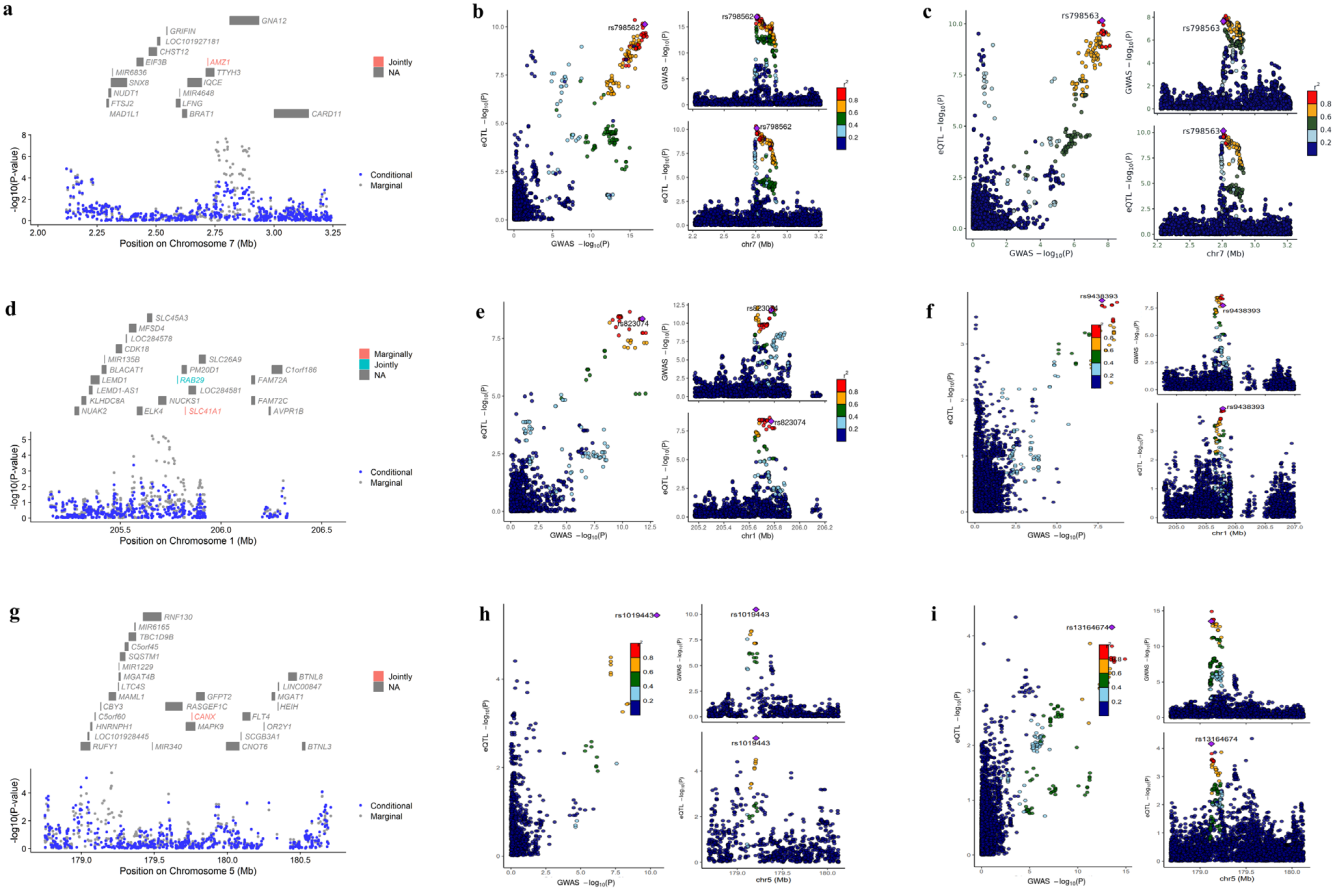
Colocalization analysis between eQTLs of genes and GWASs of brain functional connectivity traits. Manhattan plot illustrates the genomic location of genes within the locus identified in the TWAS of birth weight (a), childhood BMI (d), and adulthood BMI (g), with gray and blue dots representing before and after adjustment of jointly significant genes. LocusCompare plot compares (b) eQTLs of *AMZ1* versus GWAS of birth weight, (c) eQTLs of *AMZ1* versus GWAS of node_pheno67, (e) eQTLs of *SLC41A1* versus GWAS of childhood BMI, (f) eQTLs of *SLC41A1* versus GWAS of edge_pheno698, (h) eQTLs of *CANX* versus GWAS of adulthood BMI, and (i) eQTLs of *CANX* versus GWAS of node_pheno52.

**FIGURE 7:**
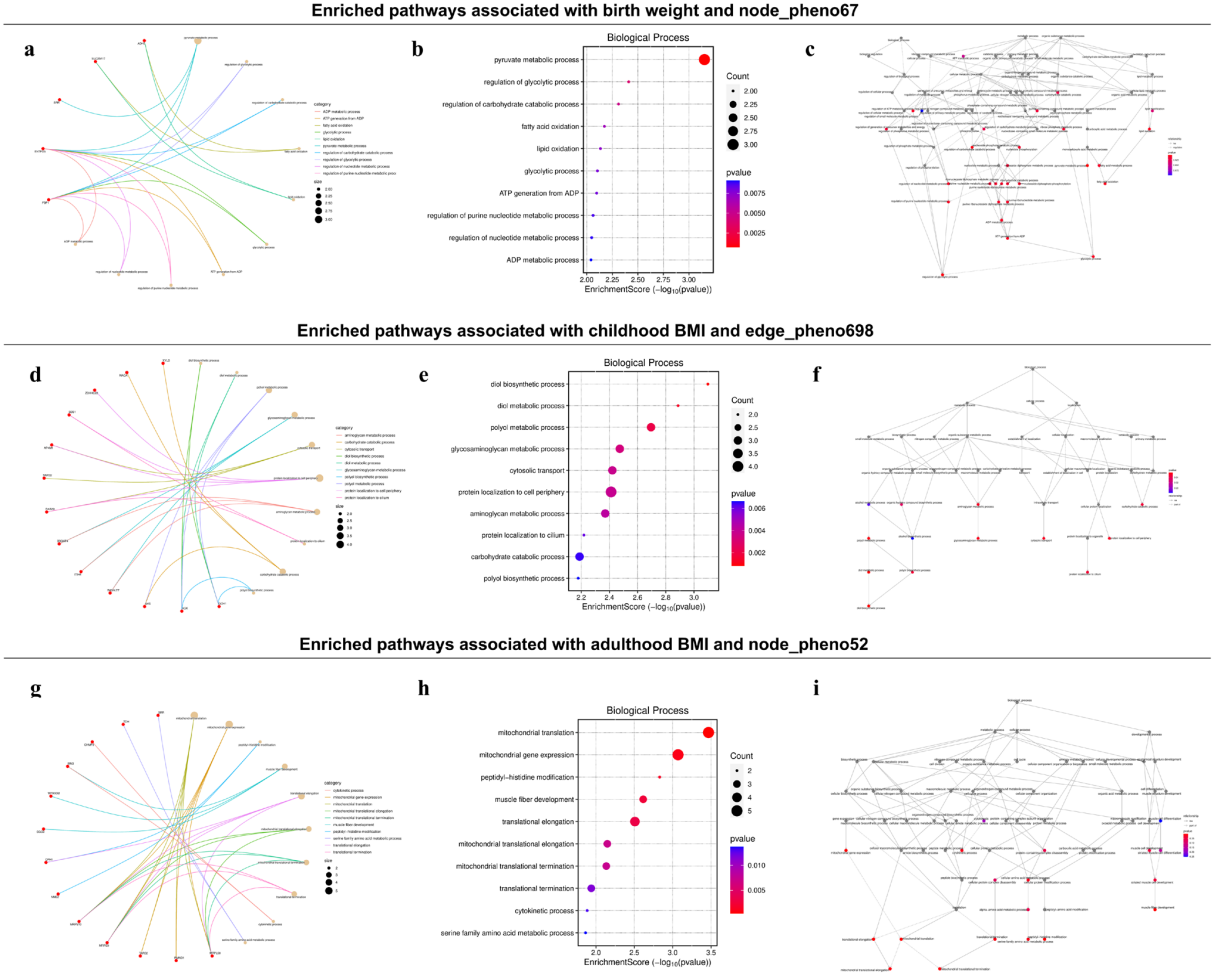
Enrichment analysis of genes associated with both life‐course body weight and brain functional connectivity. The enriched biological processes of genes associated with birth weight and node_pheno67 are illustrated in the cnetplot (a), dot plot (b), and GOplot (c). The enriched biological processes of genes associated with childhood BMI and edge_pheno698 are illustrated in the cnetplot (d), dot plot (e), and GOplot (f). The enriched biological processes of genes associated with adulthood BMI and node_pheno52 are illustrated in the cnetplot (g), dot plot (h), and GOplot (i).

Using the TWAS, we identified 37 hippocampus‐specific genes whose expression was associated with both birth weight and neural activity in the temporal lobe (Table S9). Among these, eQTL‐GWAS colocalization analysis provided further evidence supporting a causal relationship between *AMZ1* gene expression and both birth weight and node_pheno67 (Figure 6a–c). The GO enrichment results, presented in Figure 7a–c, revealed that the most common biological process was the pyruvate metabolic process.

### 3.3 Childhood BMI and Brain Functional Networks

A total of 12 rsfMRI traits were significantly associated with childhood BMI (*p* < 0.05) (Figure 2b and Table S3). After BH correction, childhood BMI demonstrated a causal effect on reduced functional connectivity between the DMN and salience network (SN) (*β* = −0.092, 95% CI: −0.145 ~ −0.040, BH corrected *p* = 0.076; edge_pheno574). Similarly, a causal relationship was observed for subcortical‐cerebellum connectivity with the motor network (MN) or attention network (AN) (*β* = −0.087, 95% CI: −0.144 ~ −0.031, BH corrected *p* = 0.076; edge_pheno698). Both associations were robust, persisting across at least one traditional sensitivity analysis and all robust methods after outlier removal (Table S4, Figure 3, and Figure S1). After adjusting for birthweight and adult body weight in the MVMR, childhood BMI remained significantly associated with edge_pheno698 (multivariable IVW *β* = −0.082, 95% CI: −0.146 ~ −0.017, *p* = 1.33E‐02) but not with edge_pheno574. We identified four proteins associated with both childhood BMI and edge_pheno698 (Table S6); however, none were found to mediate the relationship between them.

The cross‐trait LDSC (Figure 2f and Table S7) and colocalization analyses (Figure 5b) failed to identify a significant genetic association or shared causal variation between childhood BMI and the functional connectivity between the subcortical‐cerebellum and the MN or AN. The TWAS analysis identified 52 genes whose expression in cerebellum tissue was associated with both childhood BMI and edge_pheno698 (Table S9). These genes were enriched primarily in pathways related to metabolic processes, cellular processes, and localization (Figure 7d–f).

### 3.4 Adulthood BMI and Brain Functional Networks

The adulthood BMI was associated with 36 rsfMRI traits (*p* < 0.05), 11 of which remained significant after BH correction (Figure 2c and Table S3). The UVMR sensitivity and robust analyses consistently demonstrated causal associations between adulthood BMI and functional connectivity between the MN and the AN or visual network (VN) (edge_pheno1020), as well as amplitude traits in the frontal lobe (node_pheno52), fusiform gyrus (node_pheno64), and superior frontal region (node_pheno76) (Table S4, Figure 3, and Figure S1). However, after correcting for overlapping samples using the MRlap method, the significant associations between adulthood BMI and edge_pheno1020 (*β* = 0.047, 95% CI: −0.009 ~ 0.102, *p* = 1.02E‐01) and node_pheno64 (*β* = 0.065, 95% CI: −0.032 ~ 0.069, *p* = 9.45E‐02) were attenuated to null. Given these findings, we proceeded with MVMR analyses to further validate the causal associations between adulthood BMI and node_pheno52 as well as node_pheno76 (Figure 4). Adulthood BMI remained positively associated with neural activity in the frontal lobe (node_pheno52) after adjusting for early‐life body weight (multivariable IVW *β* = 0.068, 95% CI: 0.009 ~ 0.126, *p* = 2.34E‐02) and lifestyle factors (multivariable IVW *β* = 0.066, 95% CI: 0.019 ~ 0.112, *p* = 5.35E‐03). Nevertheless, the association between adulthood BMI and amplitude trait in the superior frontal region (node_pheno76) was no longer significant after adjusting for lifestyle factors (multivariable IVW *p* = 5.51E‐01). While LDSC failed to detect a significant genetic correlation (*r*_g_ = 0.09, *p* = 4.85E‐02) between adulthood BMI and neural activity in the temporal lobe after Bonferroni correction (Figure 2f and Table S7), strong evidence of colocalization at the rs42935 locus (SNP.PP.H4 = 0.991) suggests a shared causal variant (Figure 5c), further supporting the causal relationship between them.

According to the TWAS analysis (Table S9), 162 genes expressed in frontal cortex tissue were significantly associated with both adulthood BMI and node_pheno52. GO enrichment analysis revealed significant enrichment of these genes in processes related to mitochondrial translation and gene expression, emphasizing the importance of mitochondrial function (Figure 7g–i). Additionally, eQTL‐GWAS colocalization analysis provided strong evidence of colocalization (PP.H4 = 0.956) between the *CANX* gene and adulthood BMI (Figure 6h), as well as moderate evidence of colocalization (PP.H4 = 0.671) with node_pheno52 (Figure 6i).

## 4 Discussion

**FIGURE 8:**
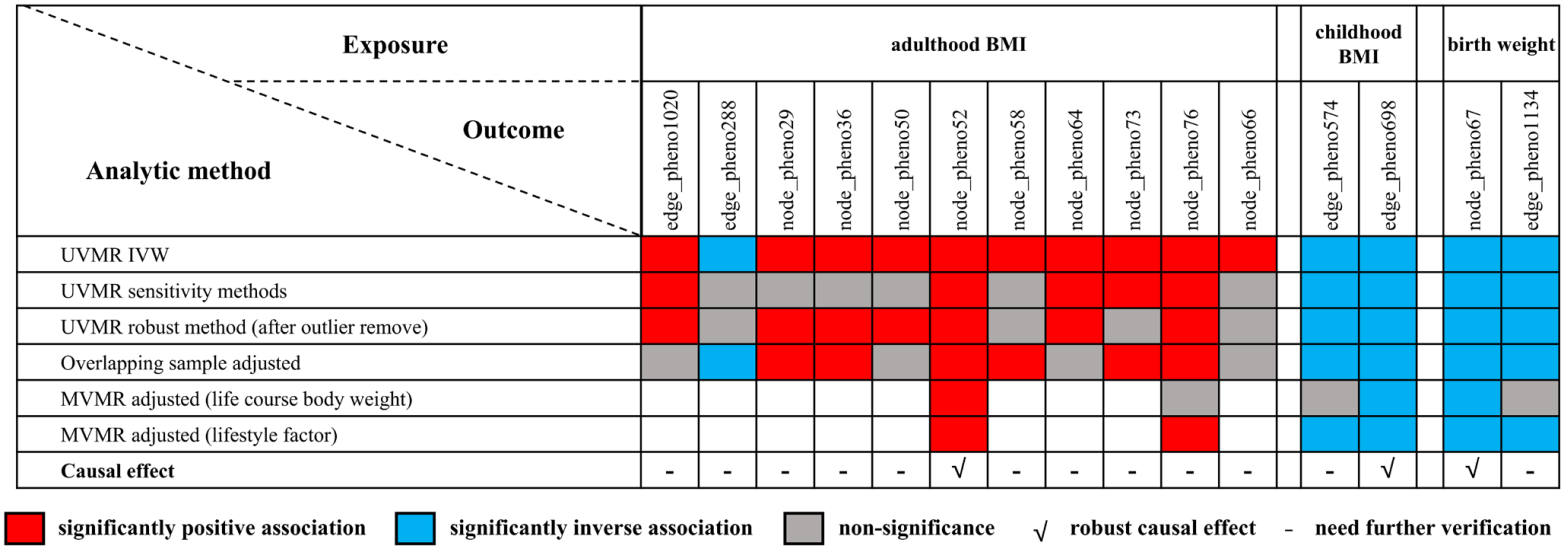
Summary of the results from all the Mendelian randomization methods.

This study provides novel insights into the life‐course effect of obesity on brain functional networks, identifying distinct critical periods during which body weight significantly influences neural connectivity and activity. Overall, a total of 66 brain functional network traits were found to be associated with body weight across different life stages (*p* < 0.05), some of which were linked to body weight at multiple stages (Figure 2d). After multiple comparison corrections, 15 of the 66 traits remained significant, with no overlap observed between life stages (Figure 2e). As summarized in Figure 8, our MR results demonstrated that adulthood BMI increased neural activity in the frontal lobe (node_pheno52), childhood BMI reduced functional connectivity between the subcortical‐cerebellum and the MN or AN (edge_pheno698), and birth weight decreased functional connectivity of the CEN or DMN in the temporal lobe (node_pheno67). These findings underscore the importance of considering life‐stage‐specific obesity interventions to mitigate their adverse effects on brain function.

The relationship between body weight and brain functional networks throughout the life course is a complex interplay that has garnered significant attention in recent neuroscience research. Various studies have highlighted how fluctuations in body weight, particularly obesity, can influence brain structure and function, ultimately affecting cognitive ability and mental health outcomes. For example, obesity has been associated with alterations in brain morphology, including reduced gray matter volume and changes in white matter integrity, which could play a role in cognitive deterioration and increase the likelihood of developing neurodegenerative conditions such as Alzheimer's disease [16, 32].

Several studies have suggested that the influence of body weight on brain functional networks varies across the lifespan, reflecting distinct patterns at different life stages [16, 33, 34]. In childhood and adolescence, elevated BMI has been associated with poorer cognitive flexibility and executive function in adulthood, indicating that early‐life obesity may predispose individuals to later cognitive challenges [33]. Leveraging rsfMRI data, our study found that childhood BMI reduces functional connectivity between the subcortical‐cerebellum and motor/attention networks, offering a potential mechanistic explanation for the connection between childhood obesity and cognitive impairments later in life. Consistent with our findings, the Adolescent Brain Cognitive Development (ABCD) study also underscored the influence of childhood BMI on cognitive performance and brain connectivity [35]. This large‐scale cohort study reported that higher childhood BMI is associated with reduced functional connectivity in key multimodal brain regions, reinforcing the link between early‐life obesity and disrupted neural networks. Additionally, research examining the connectivity between the cerebellum and motor network revealed that obesity‐related alterations in this connectivity may impair the cerebellum's role in motor execution [36]. Such disruptions could contribute to challenges in motor planning and execution, which are essential for various daily cognitive and physical activities. Together, these findings underscore the critical role of childhood BMI in shaping brain connectivity and its long‐term implications for cognitive function.

Previous studies have focused primarily on the association between low birth weight and altered brain connectivity, consistently demonstrating that individuals with low birth weight or preterm birth exhibit disrupted connectivity within critical brain networks [37, 38, 39], such as the DMN and salience network. Our study extends previous findings by identifying an inverse relationship between higher birth weight and functional connectivity between the CEN and DMN in the temporal lobe, a region crucial for sensory integration and social processing. This finding highlights a complex, non‐linear association between birth weight and brain functional networks. These results suggest that both low and high birth weights may adversely affect brain functional network development, emphasizing the need to achieve optimal birth weight for healthy connectivity. Our study demonstrates a direct association between adulthood BMI and increased neural activity in the frontal lobe, independent of early‐life body weight. This rsfMRI trait, linked to the CEN, plays a critical role in cognitive control, decision‐making, and self‐regulation—key processes for managing dietary habits and resisting unhealthy food choices [40]. These findings align with prior research suggesting that obesity‐related brain changes in connectivity disrupt self‐control and food‐related decision‐making [41]. Additionally, heightened frontal lobe activity has been associated with increased sensitivity to food cues, potentially reinforcing maladaptive dietary habits [42].

Interestingly, the biological pathways identified through TWAS and enrichment analyses in this study offer novel mechanistic insights into the observed associations, which have not been previously characterized. For example, the enrichment of mitochondrial translation pathways in adulthood BMI analysis highlights the role of mitochondrial dysfunction in obesity‐related neural changes. Similarly, our study revealed an association between childhood BMI and functional connectivity between the subcortical‐cerebellum and the MN/AN, with GO enrichment analysis implicating protein localization to the cell periphery pathway. This pathway is critical for cellular signaling, synaptic plasticity, and neural communication, which underlies motor coordination and attentional processes. Obesity‐related metabolic stress and inflammation may disrupt protein trafficking and synaptic function, contributing to neural network dysregulation [43]. These findings highlight potential cellular mechanisms linking childhood BMI to altered brain functional connectivity. Furthermore, the mediation MR analysis revealed that the causal link between birth weight and temporal lobe activity was mediated by neurexophilin‐3. This protein is known to influence synaptic strength and plasticity, particularly in brain regions associated with cognitive control and integrative processing [44]. Given the temporal lobe's role in sensory integration and higher‐order cognitive functions, neurexophilin‐3 dysfunction may alter network dynamics, contributing to impaired coordination between task‐positive and task‐negative systems. Our findings indicate that more targeted and life‐stage‐specific strategies are needed to mitigate the neurobiological consequences of obesity. For example, the distinct effects of childhood BMI on subcortical‐cerebellum connectivity to motor and attention networks suggest that early‐life interventions may be particularly critical for preserving these specific neural systems. This differs from interventions targeting adult obesity, which may be more relevant for frontal lobe activity. Practically, this could inform the development of age‐appropriate obesity management strategies: childhood interventions might emphasize motor skill development and attention training alongside weight management, whereas adult interventions might prioritize executive function support and cognitive behavioral approaches to eating behavior.

The strengths of this study lie in its robust design, integrating MR, TWAS, colocalization, and enrichment analysis to disentangle complex life‐course effects of body weight on brain functional networks. Additionally, our focus on 191 rsfMRI traits provides a comprehensive assessment of brain connectivity. However, several limitations should be noted. First, the study was restricted to European ancestry populations, potentially limiting the generalizability of our findings to other ethnic groups. Second, although MR minimizes confounding and reverse causation, it is not immune to bias from horizontal pleiotropy or weak instrument selection. While sensitivity analyses addressed these concerns, residual biases cannot be fully excluded. It is also important to note that our MR analyses assume linearity in the causal relationships between body weight and brain functional connectivity traits. While this is a standard and necessary assumption for these methods, the true biological relationships could be non‐linear (e.g., threshold effects, U‐shaped curves). Future studies with individual‐level data could explore potential non‐linear effects of BMI, particularly at extremes of the distribution. Third, the study's reliance on summary‐level data precluded subgroup analyses or interaction testing, such as sex‐specific effects or environmental modifiers. Besides, we chose BMI as the sole measure of obesity to ensure comparability between childhood and adulthood measures, given the lack of large‐scale GWAS summary data for alternative obesity indicators in children. While BMI is a widely used and practical measure of obesity, it has significant limitations including its inability to distinguish between fat mass and lean mass, as well as its limited ability to capture central adiposity or metabolic health. Future studies could benefit from the integration of additional obesity‐related biomarkers, such as lipid profiles, fasting glucose, inflammatory markers (e.g., C‐reactive protein, interleukin‐6), and adipokines (e.g., leptin, adiponectin). Multimodal obesity phenotyping combining anthropometric measurements (waist‐hip ratio, visceral adiposity index), blood biomarkers, and even advanced imaging techniques such as dual‐energy X‐ray absorptiometry (DXA) or magnetic resonance spectroscopy could provide more granular insights into specific fat distribution patterns that may differentially impact brain networks. This multidimensional approach would enhance the mechanistic understanding of how specific metabolic dysregulations influence neural systems and potentially identify more precise intervention targets. Finally, while the MVMR analyses suggested differential patterns of associations between body weight at different life stages and brain functional networks, these findings should be interpreted with caution. For example, the attenuation of birth weight effects when adjusted for later‐life BMI may reflect not only discrete developmental windows but also potential collinearity between exposures, shared genetic architecture across life stages, or cumulative effects that are challenging to disentangle through MVMR approaches. The significant genetic correlations observed between birth weight and childhood BMI underscore the shared biological pathways that may influence body weight across development. Rather than representing strictly isolated effects, our findings may reflect the complex interplay of developmentally continuous processes, biological pleiotropy, and measurement interdependence. Future longitudinal studies with repeated measures across the lifespan would help clarify whether these effects truly represent critical developmental windows or reflect more complex developmental trajectories with cascading influences.

In conclusion, this study highlights the critical influence of body weight across the life course on brain functional networks, emphasizing the distinct contributions of birth weight, childhood BMI, and adulthood BMI. By integrating advanced genetic tools, we provide robust evidence of causal relationships and elucidate potential mechanisms underlying these associations. These findings have significant implications for the development of life‐stage‐specific strategies to mitigate the neurobiological consequences of obesity.

## Author Contributions

Conceptualization and investigation: P.X.; writing – original draft and supervision: P.X. and Y.L.; writing – review and editing: J.M. and J.X.; formal analysis: J.D.

## Ethics Statement

Ethics approval and patient consent had already been received in each data source, and thus, they were waived in this study.

## Conflicts of Interest

The authors declare no conflicts of interest.

## Supporting information


**Figure S1.** MR leave‐one‐out sensitivity analysis after outlier removal.

**Table S1.** Summary of data sources used in the study.
**Table S2.** List of ID, Location, and network of 191 resting‐state functional MRI traits (75 node amplitude +116 functional connectivity).
**Table S3.** Associations of genetically predicted life‐course body weight with rsfMRI traits in univariable Mendelian randomization with IVW method.
**Table S4.** Associations of genetically predicted life‐course body weight with rsfMRI traits in univariable Mendelian randomization with traditional sensitivity methods.
**Table S5.** Heterogeneity and pleiotropy test results from MR‐Egger regression analysis.
**Table S6.** Screen the candidate proteomic mediators of the causal association between the life‐course body weight and rsfMRI traits using two‐step Mendelian randomization.
**Table S7.** Genetic correlations between investigated traits by linkage disequilibrium score regression.
**Table S8.** The heritability of investigated traits by linkage disequilibrium score regression.
**Table S9.** Common genes between life‐course body weight and rsfMRI traits in TWAS analysis.

Data S1

## Data Availability

The data that support the findings of this study are available from the corresponding author upon reasonable request.

## References

[1] H.‐J. Park and K. Friston , “Structural and Functional Brain Networks: From Connections to Cognition,” Science 342, no. 6158 (2013): 1238411. 24179229 10.1126/science.1238411

[2] P. L. Valenzuela , P. Carrera‐Bastos , A. Castillo‐García , D. E. Lieberman , A. Santos‐Lozano , and A. Lucia , “Obesity and the Risk of Cardiometabolic Diseases,” Nature Reviews. Cardiology 20, no. 7 (2023): 475–494. 36927772 10.1038/s41569-023-00847-5

[3] Y. Shang , X. Wang , S. Su , et al., “Identifying of Immune‐Associated Genes for Assessing the Obesity‐Associated Risk to the Offspring in Maternal Obesity: A Bioinformatics and Machine Learning,” CNS Neuroscience & Therapeutics 30, no. 3 (2024): e14700, 10.1111/cns.14700. 38544384 PMC10973700

[4] L. Dye , N. B. Boyle , C. Champ , and C. Lawton , “The Relationship Between Obesity and Cognitive Health and Decline,” Proceedings of the Nutrition Society 76, no. 4 (2017): 443–454. 28889822 10.1017/S0029665117002014

[5] L. Forcano , F. Mata , R. de la Torre , and A. Verdejo‐Garcia , “Cognitive and Neuromodulation Strategies for Unhealthy Eating and Obesity: Systematic Review and Discussion of Neurocognitive Mechanisms,” Neuroscience and Biobehavioral Reviews 87 (2018): 161–191. 29432784 10.1016/j.neubiorev.2018.02.003

[6] S. Saeed , A. Bonnefond , and P. Froguel , “Obesity: Exploring Its Connection to Brain Function Through Genetic and Genomic Perspectives,” Molecular Psychiatry 30 (2024): 651–658. 39237720 10.1038/s41380-024-02737-9 PMC11746128

[7] O. Le Thuc and C. García‐Cáceres , “Obesity‐Induced Inflammation: Connecting the Periphery to the Brain,” Nature Metabolism 6, no. 7 (2024): 1237–1252. 10.1038/s42255-024-01079-8 38997442

[8] G. Li , Y. Hu , W. Zhang , et al., “Brain Functional and Structural Magnetic Resonance Imaging of Obesity and Weight Loss Interventions,” Molecular Psychiatry 28, no. 4 (2023): 1466–1479. 36918706 10.1038/s41380-023-02025-y PMC10208984

[9] B. Zhao , T. Li , S. M. Smith , et al., “Common Variants Contribute to Intrinsic Human Brain Functional Networks,” Nature Genetics 54, no. 4 (2022): 508–517. 35393594 10.1038/s41588-022-01039-6 PMC11987081

[10] E. S. Finn , X. Shen , D. Scheinost , et al., “Functional Connectome Fingerprinting: Identifying Individuals Using Patterns of Brain Connectivity,” Nature Neuroscience 18, no. 11 (2015): 1664–1671. 26457551 10.1038/nn.4135 PMC5008686

[11] G. Horga , C. M. Cassidy , X. Xu , et al., “Dopamine‐Related Disruption of Functional Topography of Striatal Connections in Unmedicated Patients With Schizophrenia,” JAMA Psychiatry 73, no. 8 (2016): 862–870. 27145361 10.1001/jamapsychiatry.2016.0178 PMC5310843

[12] C.‐G. Yan , X. Chen , L. Li , et al., “Reduced Default Mode Network Functional Connectivity in Patients With Recurrent Major Depressive Disorder,” Proceedings of the National Academy of Sciences of the United States of America 116, no. 18 (2019): 9078–9083. 30979801 10.1073/pnas.1900390116 PMC6500168

[13] A. Badhwar , A. Tam , C. Dansereau , P. Orban , F. Hoffstaedter , and P. Bellec , “Resting‐State Network Dysfunction in Alzheimer's Disease: A Systematic Review and Meta‐Analysis,” Alzheimers Dement (Amst) 8 (2017): 73–85. 28560308 10.1016/j.dadm.2017.03.007 PMC5436069

[14] C. Avila , A. C. Holloway , M. K. Hahn , et al., “An Overview of Links Between Obesity and Mental Health,” Current Obesity Reports 4, no. 3 (2015): 303–310. 26627487 10.1007/s13679-015-0164-9

[15] W. Guan , D.‐W. Xu , C.‐H. Ji , et al., “Hippocampal miR‐206‐3p Participates in the Pathogenesis of Depression via Regulating the Expression of BDNF,” Pharmacological Research 174 (2021): 105932, 10.1016/j.phrs.2021.105932. 34628001

[16] F. Morys , C. Tremblay , S. Rahayel , et al., “Neural Correlates of Obesity Across the Lifespan,” Communications Biology 7, no. 1 (2024): 656. 38806652 10.1038/s42003-024-06361-9 PMC11133431

[17] M. K. Singh , S. M. Leslie , M. M. Packer , et al., “Brain and Behavioral Correlates of Insulin Resistance in Youth With Depression and Obesity,” Hormones and Behavior 108 (2019): 73–83. 29596854 10.1016/j.yhbeh.2018.03.009 PMC6173667

[18] E. Sanderson , M. M. Glymour , M. V. Holmes , et al., “Mendelian Randomization,” Nature Reviews Methods Primers 2, no. 1 (2022): 6. 10.1038/s43586-021-00092-5 PMC7614635 37325194

[19] T. G. Richardson , E. Sanderson , B. Elsworth , K. Tilling , and S. G. Davey , “Use of Genetic Variation to Separate the Effects of Early and Later Life Adiposity on Disease Risk: Mendelian Randomisation Study,” BMJ 369 (2020): m1203. 32376654 10.1136/bmj.m1203 PMC7201936

[20] P. Xiao , C. Li , J. Mi , and J. Wu , “Evaluating the Distinct Effects of Body Mass Index at Childhood and Adulthood on Adult Major Psychiatric Disorders,” Science Advances 10, no. 37 (2024): eadq2452. 39270013 10.1126/sciadv.adq2452 PMC11397431

[21] P. Xiao , C. Li , J. Wu , and J. Dai , “Unravel the Distinct Effects of Adiposity at Different Life Stages on COVID‐19 Susceptibility and Severity: A Life‐Course Mendelian Randomization Study,” Journal of Medical Virology 96, no. 10 (2024): e29943. 39360640 10.1002/jmv.29943

[22] C. Jin , X. Tao , W. Zhang , et al., “Multi‐Omics and Multi‐Stages Integration Identified a Novel Variant Associated With Silicosis Risk,” Archives of Toxicology 98, no. 9 (2024): 2907–2918, 10.1007/s00204-024-03795-2. 38811393

[23] V. W. Skrivankova , R. C. Richmond , B. A. R. Woolf , et al., “Strengthening the Reporting of Observational Studies in Epidemiology Using Mendelian Randomization: The STROBE‐MR Statement,” JAMA 326, no. 16 (2021): 1614–1621. 34698778 10.1001/jama.2021.18236

[24] S. Vogelezang , J. P. Bradfield , T. S. Ahluwalia , et al., “Novel Loci for Childhood Body Mass Index and Shared Heritability With Adult Cardiometabolic Traits,” PLoS Genetics 16, no. 10 (2020): e1008718. 33045005 10.1371/journal.pgen.1008718 PMC7581004

[25] N. M. Warrington , R. N. Beaumont , M. Horikoshi , et al., “Maternal and Fetal Genetic Effects on Birth Weight and Their Relevance to Cardio‐Metabolic Risk Factors,” Nature Genetics 51, no. 5 (2019): 804–814. 31043758 10.1038/s41588-019-0403-1 PMC6522365

[26] A. E. Locke , B. Kahali , S. I. Berndt , et al., “Genetic Studies of Body Mass Index Yield New Insights for Obesity Biology,” Nature 518, no. 7538 (2015): 197–206. 25673413 10.1038/nature14177 PMC4382211

[27] B. B. Sun , J. C. Maranville , J. E. Peters , et al., “Genomic Atlas of the Human Plasma Proteome,” Nature 558, no. 7708 (2018): 73–79. 29875488 10.1038/s41586-018-0175-2 PMC6697541

[28] Z. Zhu , Z. Zheng , F. Zhang , et al., “Causal Associations Between Risk Factors and Common Diseases Inferred From GWAS Summary Data,” Nature Communications 9, no. 1 (2018): 224. 10.1038/s41467-017-02317-2 PMC5768719 29335400

[29] J. Zhao , J. Ming , X. Hu , G. Chen , J. Liu , and C. Yang , “Bayesian Weighted Mendelian Randomization for Causal Inference Based on Summary Statistics,” Bioinformatics 36, no. 5 (2020): 1501–1508. 31593215 10.1093/bioinformatics/btz749

[30] N. Mounier and Z. Kutalik , “Bias Correction for Inverse Variance Weighting Mendelian Randomization,” Genetic Epidemiology 47, no. 4 (2023): 314–331. 37036286 10.1002/gepi.22522

[31] S. M. Williams , C. Giambartolomei , D. Vukcevic , et al., “Bayesian Test for Colocalisation Between Pairs of Genetic Association Studies Using Summary Statistics,” PLoS Genetics 10, no. 5 (2014): e1004383. 24830394 10.1371/journal.pgen.1004383 PMC4022491

[32] M. H. C. Nota , D. Vreeken , M. Wiesmann , E. O. Aarts , E. J. Hazebroek , and A. J. Kiliaan , “Obesity Affects Brain Structure and Function‐ Rescue by Bariatric Surgery?,” Neuroscience and Biobehavioral Reviews 108 (2020): 646–657. 31794778 10.1016/j.neubiorev.2019.11.025

[33] L. Kupis , Z. T. Goodman , L. Kircher , et al., “Altered Patterns of Brain Dynamics Linked With Body Mass Index in Youth With Autism,” Autism Research: Official Journal of the International Society for Autism Research 14, no. 5 (2021): 873–886. 33616282 10.1002/aur.2488

[34] I. K. Karlsson , K. Lehto , M. Gatz , C. A. Reynolds , and A. K. Dahl Aslan , “Age‐Dependent Effects of Body Mass Index Across the Adult Life Span on the Risk of Dementia: A Cohort Study With a Genetic Approach,” BMC Medicine 18, no. 1 (2020): 131. 32513281 10.1186/s12916-020-01600-2 PMC7282125

[35] D. Tomasi and N. D. Volkow , “Childhood Obesity's Effect on Cognition and Brain Connectivity Worsens With Low Family Income,” JCI Insight 9, no. 16 (2024): e181690. 38980723 10.1172/jci.insight.181690 PMC11343596

[36] J. H. Jung , B. H. Kim , S. J. Chung , et al., “Motor Cerebellar Connectivity and Future Development of Freezing of Gait in De Novo Parkinson's Disease,” Movement Disorders: Official Journal of the Movement Disorder Society 35, no. 12 (2020): 2240–2249. 32926481 10.1002/mds.28243

[37] C. Nosarti , “Social Relationships, Preterm Birth or Low Birth Weight, and the Brain,” JAMA Network Open 2, no. 7 (2019): e196960. 31298710 10.1001/jamanetworkopen.2019.6960

[38] S. Atzil , W. Gao , I. Fradkin , and L. F. Barrett , “Growing a Social Brain,” Nature Human Behaviour 2, no. 9 (2018): 624–636. 10.1038/s41562-018-0384-6 31346259

[39] T. P. White , I. Symington , N. P. Castellanos , et al., “Dysconnectivity of Neurocognitive Networks at Rest in Very‐Preterm Born Adults,” NeuroImage. Clinical 4 (2014): 352–365. 24567907 10.1016/j.nicl.2014.01.005 PMC3930099

[40] N. P. Friedman and T. W. Robbins , “The Role of Prefrontal Cortex in Cognitive Control and Executive Function,” Neuropsychopharmacology 47, no. 1 (2022): 72–89. 34408280 10.1038/s41386-021-01132-0 PMC8617292

[41] S. Carnell , C. Gibson , L. Benson , C. N. Ochner , and A. Geliebter , “Neuroimaging and Obesity: Current Knowledge and Future Directions,” Obesity Reviews: An Official Journal of the International Association for the Study of Obesity 13, no. 1 (2012): 43–56. 21902800 10.1111/j.1467-789X.2011.00927.x PMC3241905

[42] S. D. Donofry , C. M. Stillman , and K. I. Erickson , “A Review of the Relationship Between Eating Behavior, Obesity and Functional Brain Network Organization,” Social Cognitive and Affective Neuroscience 15, no. 10 (2020): 1157–1181. 31680149 10.1093/scan/nsz085 PMC7657447

[43] F. Gómez‐Pinilla , “Brain Foods: The Effects of Nutrients on Brain Function,” Nature Reviews. Neuroscience 9, no. 7 (2008): 568–578. 18568016 10.1038/nrn2421 PMC2805706

[44] T. C. Südhof , “Towards an Understanding of Synapse Formation,” Neuron 100, no. 2 (2018): 276–293. 30359597 10.1016/j.neuron.2018.09.040 PMC6226307

