# Supplementary figures and images for: Identification of the Critical Life‐Stage of Obesity Contributing to Brain Functional Networks
Source: CNS Neurosci Ther. 2025 Jul 10;31(7):e70510. doi: 10.1111/cns.70510 (PMC12241826; doi:10.1111/cns.70510)

**Figure S1. MR leave-one-out sensitivity analysis after outlier removal.**

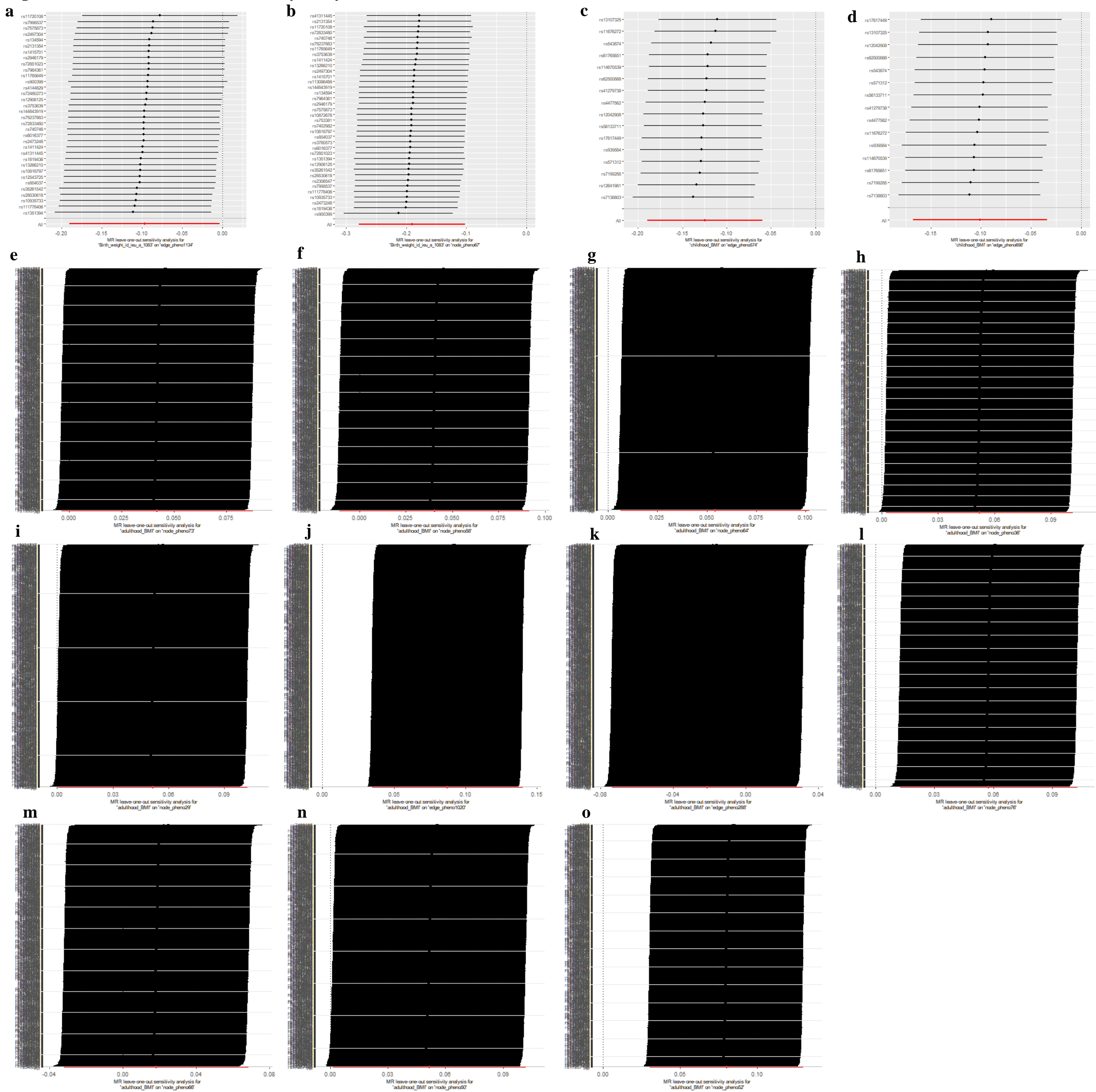

Supplement: Supplementary file 1 — Figure S1. MR leave‐one‐out sensitivity analysis after outlier removal. [file CNS-31-e70510-s001.pdf]
