# Supplementary material for: Identification of the Critical Life‐Stage of Obesity Contributing to Brain Functional Networks
Source: CNS Neurosci Ther. 2025 Jul 10;31(7):e70510. doi: 10.1111/cns.70510 (PMC12241826; doi:10.1111/cns.70510)
Supplement: Supplementary file 2 — Table S1. Summary of data sources used in the study. Table S2. List of ID, Location, and network of 191 resting‐state functional MRI traits (75 node amplitude +116 functional connectivity). Table S3. Associations of genetically predicted life‐course body weight with rsfMRI traits in univariable Mendelian randomization with IVW method. Table S4. Associations of genetically predicted life‐course body weight with rsfMRI traits in univariable Mendelian randomization with traditional sensitivity methods. Table S5. Heterogeneity and pleiotropy test results from MR‐Egger regression analysis. Table S6. Screen the candidate proteomic mediators of the causal association between the life‐course body weight and rsfMRI traits using two‐step Mendelian randomization. Table S7. Genetic correlations between investigated traits by linkage disequilibrium score regression. Table S8. The heritability of investigated traits by linkage disequilibrium score regression. Table S9. Common genes between life‐course body weight and rsfMRI traits in TWAS analysis. [file CNS-31-e70510-s002.docx]

**Supplementary tables content**

Table S1. Summary of data sources used in the study.

Table S2. List of ID, Location, and network of 191 resting-state functional MRI traits (75 node amplitude + 116 functional connectivity).

Table S3. Associations of genetically predicted life-course body weight with rsfMRI traits in univariable Mendelian randomization with IVW method.

Table S4. Associations of genetically predicted life-course body weight with rsfMRI traits in univariable Mendelian randomization with traditional sensitivity methods.

Table S5. Heterogeneity and pleiotropy test results from MR-Egger regression analysis.

Table S6. Screen the candidate proteomic mediators of the causal association between the life-course body weight and rsfMRI traits using two-step Mendelian randomization.

Table S7. Genetic correlations between investigated traits by linkage disequilibrium score regression.

Table S8. The heritability of investigated traits by linkage disequilibrium score regression.

Table S9. Common genes between life-course body weight and rsfMRI traits in TWAS analysis.

**Table S1. Summary of data source used in the study.**

| **Category** | **Trait** | **Unit** | **Lead author** | **Consortium** | **Pubmed ID** | **Sample size (European ancestry)** | **Year** | **Data source** |
| --- | --- | --- | --- | --- | --- | --- | --- | --- |
| exposure | birth weight | sex- and age-adjusted SD | Warrington NM | Early Growth Genetics Consortium | 31043758 | 80,745 individuals | 2019 | https://egg-consortium.org/birth-weight-2019.html |
| exposure | childhood body mass index | sex- and age-adjusted SD | Vogelezang S | Early Growth Genetics Consortium | 33045005 | 39,620 children aged between 2 and 10 years | 2020 | https://egg-consortium.org/Childhood-body-mass-index-2020.html |
| exposure | adulthood body mass index | kg/m^2^ | Locke AE | Genetic Investigation of ANthropometric Traits consortium | 25673413 | 322,154 individuals | 2015 | https://portals.broadinstitute.org/collaboration/giant/index.php |
| outcome | 191 resting-state functional MRI (rs-fMRI) traits | SD | Bingxin Z | - | 35393594 | 34,691 individuals | 2022 | https://doi.org/10.5281/zenodo.5775047 |
| covariate | Current tobacco smoking | SD | Ben Elsworth | MRC-IEU | - | 462,434 individuals | 2021 | https://gwas.mrcieu.ac.uk/datasets/ukb-b-223/ |
| covariate | Alcoholic drinks per week | SD | Liu M | GSCAN | 30643251 | 335,394 individuals | 2019 | https://gwas.mrcieu.ac.uk/datasets/ieu-b-73/ |
| covariate | Sleep duration | SD | Ben Elsworth | MRC-IEU | - | 460,099 individuals | 2018 | https://gwas.mrcieu.ac.uk/datasets/z/ |
| covariate | Moderate to vigorous physical activity levels | SD | Klimentidis YC | - | 29899525 | 377,234 individuals | 2018 | https://gwas.mrcieu.ac.uk/datasets/ebi-a-GCST006097/ |
| mediator | Plasma proteome (3282 traits) | SD | Sun BB | - | 29875488 | 3,301 individuals | 2019 | https://gwas.mrcieu.ac.uk/datasets |

**Table S2. List of ID, Location, and network of 191 resting-state functional MRI traits (75 node amplitude + 116 functional connectivity).**

| **Pheno_ID** | **Location** | **Network** |
| --- | --- | --- |
| edge_pheno55 | (Postcentral\|Precentral)&(Postcentral\|Precentral) | (Motor)&(Motor) |
| edge_pheno58 | (Pariental)&(Paracentral\|Postcentral) | (Attention\|Salience\|Central_executive)&(Motor) |
| edge_pheno65 | (Postcentral\|Precentral)&(Paracentral\|Postcentral) | (Motor)&(Motor) |
| edge_pheno66 | (Postcentral\|Precentral)&(Paracentral\|Postcentral) | (Motor)&(Motor) |
| edge_pheno87 | (Parietal\|Temporal)&(Cingulate\|Frontal) | (Default_mode\|Central_executive)&(Default_mode\|Central_executive\|Salience) |
| edge_pheno101 | (Postcentral\|Precentral)&(Cerebellum) | (Motor)&(Subcortical-cerebellum) |
| edge_pheno102 | (Postcentral\|Precentral)&(Cerebellum) | (Motor)&(Subcortical-cerebellum) |
| edge_pheno103 | (Paracentral\|Postcentral)&(Cerebellum) | (Motor)&(Subcortical-cerebellum) |
| edge_pheno132 | (Paracentral\|Postcentral)&(Temporal) | (Motor)&(Default_mode\|Motor) |
| edge_pheno135 | (Cerebellum)&(Temporal) | (Subcortical-cerebellum)&(Default_mode\|Motor) |
| edge_pheno146 | (Postcentral\|Precentral)&(Subcortical) | (Motor)&(Subcortical-cerebellum) |
| edge_pheno147 | (Postcentral\|Precentral)&(Subcortical) | (Motor)&(Subcortical-cerebellum) |
| edge_pheno151 | (Cerebellum)&(Subcortical) | (Subcortical-cerebellum)&(Subcortical-cerebellum) |
| edge_pheno249 | (Lingual\|Fusiform)&(OccipitalPrecuneus) | (Visual)&(Default_mode\|Central_executive) |
| edge_pheno253 | (Frontal\|Cingulate)&(OccipitalPrecuneus) | (Default_mode\|Limbic)&(Default_mode\|Central_executive) |
| edge_pheno262 | (Frontal\|Cingulate)&(Angular\|Temporal) | (Default_mode\|Limbic)&(Default_mode\|Central_executive) |
| edge_pheno286 | (OccipitalPrecuneus)&(Frontal_Sup) | (Default_mode\|Central_executive)&(Default_mode) |
| edge_pheno288 | (Frontal\|Cerebellum)&(Frontal_Sup) | (Central_executive\|Default_mode)&(Default_mode) |
| edge_pheno303 | (RolandicOper\|SupraMarginal\|Insula)&(Insula\|Cingulate) | (Salience\|Motor)&(Salience\|Default_mode) |
| edge_pheno389 | (Calcarine\|Lingual)&(Postcentral\|Precentral) | (Visual)&(Motor\|Attention) |
| edge_pheno405 | (Precuneus\|Angular\|Cingulate)&(Frontal_Sup) | (Default_mode\|Central_executive)&(Default_mode\|Central_executive) |
| edge_pheno447 | (Postcentral\|Precentral)&(Cerebellum) | (Motor)&(Subcortical-cerebellum) |
| edge_pheno449 | (Calcarine\|Lingual)&(Cerebellum) | (Visual)&(Subcortical-cerebellum) |
| edge_pheno460 | (Precuneus\|Cuneus\|Cingulate)&(Cerebellum) | (Default_mode\|Central_executive)&(Subcortical-cerebellum) |
| edge_pheno461 | (Postcentral\|Precentral)&(Cerebellum) | (Motor\|Attention)&(Subcortical-cerebellum) |
| edge_pheno491 | (Precuneus\|Angular\|Cingulate)&(Frontal\|Precentral) | (Default_mode\|Central_executive)&(Central_executive) |
| edge_pheno537 | (RolandicOper\|SupraMarginal\|Insula)&(Precentral\|Frontal\|Supp_Motor_Area) | (Salience\|Motor)&(Attention\|Salience\|Motor) |
| edge_pheno558 | (Cerebellum)&(Precentral\|Frontal\|Supp_Motor_Area) | (Subcortical-cerebellum)&(Attention\|Salience\|Motor) |
| edge_pheno574 | (Frontal_Sup)&(Supp_Motor_Area\|Frontal) | (Default_mode)&(Salience\|Default_mode) |
| edge_pheno590 | (RolandicOper\|SupraMarginal\|Insula)&(Parietal) | (Salience\|Motor)&(Central_executive\|Salience) |
| edge_pheno593 | (Precuneus\|Angular\|Cingulate)&(Parietal) | (Default_mode\|Central_executive)&(Central_executive\|Salience) |
| edge_pheno597 | (Temporal)&(Parietal) | (Default_mode)&(Central_executive\|Salience) |
| edge_pheno599 | (Angular\|Temporal)&(Parietal) | (Default_mode\|Central_executive)&(Central_executive\|Salience) |
| edge_pheno601 | (Frontal_Sup)&(Parietal) | (Default_mode)&(Central_executive\|Salience) |
| edge_pheno606 | (Parietal)&(Parietal) | (Attention\|Central_executive\|Salience)&(Central_executive\|Salience) |
| edge_pheno609 | (Frontal_Sup)&(Parietal) | (Default_mode\|Central_executive)&(Central_executive\|Salience) |
| edge_pheno621 | (Precuneus\|Angular\|Cingulate)&(Paracentral) | (Default_mode\|Central_executive)&(Motor) |
| edge_pheno624 | (Calcarine\|Lingual)&(Paracentral) | (Visual)&(Motor) |
| edge_pheno636 | (Postcentral\|Precentral)&(Paracentral) | (Motor\|Attention)&(Motor) |
| edge_pheno639 | (Cerebellum)&(Paracentral) | (Subcortical-cerebellum)&(Motor) |
| edge_pheno674 | (Parietal)&(Frontal) | (Central_executive\|Salience)&(Central_executive) |
| edge_pheno681 | (Postcentral\|Precentral)&(Postcentral\|Precentral) | (Motor)&(Motor\|Attention) |
| edge_pheno683 | (Calcarine\|Lingual)&(Postcentral\|Precentral) | (Visual)&(Motor\|Attention) |
| edge_pheno695 | (Postcentral\|Precentral)&(Postcentral\|Precentral) | (Motor\|Attention)&(Motor\|Attention) |
| edge_pheno698 | (Cerebellum)&(Postcentral\|Precentral) | (Subcortical-cerebellum)&(Motor\|Attention) |
| edge_pheno705 | (Paracentral)&(Postcentral\|Precentral) | (Motor)&(Motor\|Attention) |
| edge_pheno716 | (OccipitalPrecuneus)&(Temporal\|Frontal\|Supp_Motor_Area) | (Default_mode\|Central_executive)&(Default_mode\|Salience) |
| edge_pheno767 | (Parietal)&(Parietal\|Postcentral\|Precuneus) | (Central_executive\|Salience)&(Attention) |
| edge_pheno777 | (Postcentral\|Precentral)&(Postcentral\|Precentral) | (Motor)&(Motor) |
| edge_pheno789 | (Parietal)&(Postcentral\|Precentral) | (Attention\|Central_executive\|Salience)&(Motor) |
| edge_pheno794 | (Cerebellum)&(Postcentral\|Precentral) | (Subcortical-cerebellum)&(Motor) |
| edge_pheno801 | (Paracentral)&(Postcentral\|Precentral) | (Motor)&(Motor) |
| edge_pheno810 | (Precuneus\|Angular\|Cingulate)&(Precuneus) | (Default_mode\|Central_executive)&(Default_mode\|Central_executive) |
| edge_pheno812 | (Frontal\|Cingulate)&(Precuneus) | (Default_mode\|Limbic)&(Default_mode\|Central_executive) |
| edge_pheno815 | (OccipitalPrecuneus)&(Precuneus) | (Default_mode\|Central_executive)&(Default_mode\|Central_executive) |
| edge_pheno816 | (Angular\|Temporal)&(Precuneus) | (Default_mode\|Central_executive)&(Default_mode\|Central_executive) |
| edge_pheno824 | (Precuneus\|Cuneus\|Cingulate)&(Precuneus) | (Default_mode\|Central_executive)&(Default_mode\|Central_executive) |
| edge_pheno867 | (Precentral\|Frontal\|Supp_Motor_Area)&(Frontal) | (Attention\|Salience\|Motor)&(Central_executive\|Salience) |
| edge_pheno869 | (Parietal)&(Frontal) | (Central_executive\|Salience)&(Central_executive\|Salience) |
| edge_pheno882 | (Postcentral\|Precentral)&(Subcortical) | (Motor)&(Subcortical-cerebellum) |
| edge_pheno899 | (Cerebellum)&(Subcortical) | (Subcortical-cerebellum)&(Subcortical-cerebellum) |
| edge_pheno903 | (Precentral\|Frontal\|Supp_Motor_Area)&(Subcortical) | (Attention\|Salience\|Motor)&(Subcortical-cerebellum) |
| edge_pheno908 | (Postcentral\|Precentral)&(Subcortical) | (Motor\|Attention)&(Subcortical-cerebellum) |
| edge_pheno918 | (Precuneus\|Angular\|Cingulate)&(Precuneus\|Parietal_Sup) | (Default_mode\|Central_executive)&(Attention\|Central_executive) |
| edge_pheno932 | (Precuneus\|Cuneus\|Cingulate)&(Precuneus\|Parietal_Sup) | (Default_mode\|Central_executive)&(Attention\|Central_executive) |
| edge_pheno942 | (Parietal)&(Precuneus\|Parietal_Sup) | (Central_executive\|Salience)&(Attention\|Central_executive) |
| edge_pheno956 | (Precuneus\|Angular\|Cingulate)&(Frontal) | (Default_mode\|Central_executive)&(Central_executive) |
| edge_pheno965 | (Cuneus\|Occipital)&(Frontal) | (Visual)&(Central_executive) |
| edge_pheno1013 | (Cerebellum)&(Temporal\|Occipital) | (Subcortical-cerebellum)&(Attention\|Visual) |
| edge_pheno1020 | (Paracentral)&(Temporal\|Occipital) | (Motor)&(Attention\|Visual) |
| edge_pheno1022 | (Postcentral\|Precentral)&(Temporal\|Occipital) | (Motor\|Attention)&(Attention\|Visual) |
| edge_pheno1041 | (Angular\|Temporal)&(Temporal\|Occipital) | (Default_mode\|Central_executive)&(Attention\|Visual) |
| edge_pheno1059 | (Parietal)&(Temporal\|Occipital) | (Central_executive\|Salience)&(Attention\|Visual) |
| edge_pheno1122 | (Temporal)&(Frontal_Inf) | (Default_mode)&(Default_mode\|Central_executive) |
| edge_pheno1126 | (Frontal_Sup)&(Frontal_Inf) | (Default_mode)&(Default_mode\|Central_executive) |
| edge_pheno1134 | (Frontal_Sup)&(Frontal_Inf) | (Default_mode\|Central_executive)&(Default_mode\|Central_executive) |
| edge_pheno1137 | (Parietal)&(Frontal_Inf) | (Central_executive\|Attention)&(Default_mode\|Central_executive) |
| edge_pheno1141 | (Supp_Motor_Area\|Frontal)&(Frontal_Inf) | (Salience\|Default_mode)&(Default_mode\|Central_executive) |
| edge_pheno1142 | (Parietal)&(Frontal_Inf) | (Central_executive\|Salience)&(Default_mode\|Central_executive) |
| edge_pheno1161 | (Precuneus\|Angular\|Cingulate)&(Frontal) | (Default_mode\|Central_executive)&(Salience\|Default_mode) |
| edge_pheno1167 | (Angular\|Temporal)&(Frontal) | (Default_mode\|Central_executive)&(Salience\|Default_mode) |
| edge_pheno1171 | (Insula\|Cingulate)&(Frontal) | (Salience\|Default_mode)&(Salience\|Default_mode) |
| edge_pheno1175 | (Precuneus\|Cuneus\|Cingulate)&(Frontal) | (Default_mode\|Central_executive)&(Salience\|Default_mode) |
| edge_pheno1183 | (Precentral\|Frontal\|Supp_Motor_Area)&(Frontal) | (Attention\|Salience\|Motor)&(Salience\|Default_mode) |
| edge_pheno1184 | (Supp_Motor_Area\|Frontal)&(Frontal) | (Salience\|Default_mode)&(Salience\|Default_mode) |
| edge_pheno1189 | (Temporal\|Frontal\|Supp_Motor_Area)&(Frontal) | (Default_mode\|Salience)&(Salience\|Default_mode) |
| edge_pheno1205 | (Precuneus\|Angular\|Cingulate)&(Temporal) | (Default_mode\|Central_executive)&(Central_executive\|Default_mode) |
| edge_pheno1211 | (Angular\|Temporal)&(Temporal) | (Default_mode\|Central_executive)&(Central_executive\|Default_mode) |
| edge_pheno1221 | (Frontal_Sup)&(Temporal) | (Default_mode\|Central_executive)&(Central_executive\|Default_mode) |
| edge_pheno1225 | (Frontal\|Precentral)&(Temporal) | (Central_executive)&(Central_executive\|Default_mode) |
| edge_pheno1250 | (Precuneus\|Angular\|Cingulate)&(Frontal) | (Default_mode\|Central_executive)&(Central_executive\|Salience\|Default_mode) |
| edge_pheno1256 | (Angular\|Temporal)&(Frontal) | (Default_mode\|Central_executive)&(Central_executive\|Salience\|Default_mode) |
| edge_pheno1257 | (Frontal\|Cerebellum)&(Frontal) | (Central_executive\|Default_mode)&(Central_executive\|Salience\|Default_mode) |
| edge_pheno1269 | (Parietal)&(Frontal) | (Central_executive\|Attention)&(Central_executive\|Salience\|Default_mode) |
| edge_pheno1270 | (Frontal\|Precentral)&(Frontal) | (Central_executive)&(Central_executive\|Salience\|Default_mode) |
| edge_pheno1273 | (Supp_Motor_Area\|Frontal)&(Frontal) | (Salience\|Default_mode)&(Central_executive\|Salience\|Default_mode) |
| edge_pheno1276 | (Frontal)&(Frontal) | (Central_executive)&(Central_executive\|Salience\|Default_mode) |
| edge_pheno1293 | (RolandicOper\|SupraMarginal\|Insula)&(Temporal) | (Salience\|Motor)&(Default_mode\|Central_executive) |
| edge_pheno1296 | (Precuneus\|Angular\|Cingulate)&(Temporal) | (Default_mode\|Central_executive)&(Default_mode\|Central_executive) |
| edge_pheno1300 | (Temporal)&(Temporal) | (Default_mode)&(Default_mode\|Central_executive) |
| edge_pheno1301 | (OccipitalPrecuneus)&(Temporal) | (Default_mode\|Central_executive)&(Default_mode\|Central_executive) |
| edge_pheno1302 | (Angular\|Temporal)&(Temporal) | (Default_mode\|Central_executive)&(Default_mode\|Central_executive) |
| edge_pheno1309 | (Parietal)&(Temporal) | (Attention\|Central_executive\|Salience)&(Default_mode\|Central_executive) |
| edge_pheno1311 | (Postcentral\|Precentral)&(Temporal) | (Motor\|Attention)&(Default_mode\|Central_executive) |
| edge_pheno1317 | (Parietal\|Frontal)&(Temporal) | (Attention\|Central_executive)&(Default_mode\|Central_executive) |
| edge_pheno1319 | (Supp_Motor_Area\|Frontal)&(Temporal) | (Salience\|Default_mode)&(Default_mode\|Central_executive) |
| edge_pheno1322 | (Frontal)&(Temporal) | (Central_executive)&(Default_mode\|Central_executive) |
| edge_pheno1325 | (Parietal\|Postcentral\|Precuneus)&(Temporal) | (Attention)&(Default_mode\|Central_executive) |
| edge_pheno1328 | (Frontal)&(Temporal) | (Central_executive\|Salience)&(Default_mode\|Central_executive) |
| edge_pheno1359 | (Frontal_Sup)&(Temporal_Mid\|Angular) | (Default_mode\|Central_executive)&(Default_mode) |
| edge_pheno1382 | (Frontal_Inf)&(Temporal_Mid\|Angular) | (Default_mode\|Central_executive)&(Default_mode) |
| edge_pheno1696 | NA(Global_measure) | Motor\|Subcortical-cerebellum |
| edge_pheno1697 | NA(Global_measure) | Triple_netwoks(Default_mode,Central_executive,Salience) |
| edge_pheno1698 | NA(Global_measure) | Triple_netwoks(Default_mode,Central_executive,Salience)\|Attention |
| edge_pheno1699 | NA(Global_measure) | Triple_netwoks(Default_mode,Central_executive,Salience)\|Attention |
| edge_pheno1701 | NA(Global_measure) | Triple_netwoks(Default_mode,Central_executive,Salience) |
| node_pheno1 | Cerebellum\|Precuneus | Default_mode |
| node_pheno2 | Occipital | Visual\|Attention |
| node_pheno3 | Pariental | Attention\|Salience\|Central_executive |
| node_pheno4 | Calcarine\|Lingual\|Cuneus | Visual |
| node_pheno5 | Parietal\|Cerebellum | Central_executive\|Attention\|Default_mode |
| node_pheno7 | Precuneus\|Occipital | Default_mode\|Central_executive |
| node_pheno8 | Occipital | Visual |
| node_pheno9 | Parietal\|Temporal | Default_mode\|Central_executive |
| node_pheno10 | Postcentral\|Precentral | Motor |
| node_pheno11 | Postcentral\|Precentral | Motor |
| node_pheno12 | Paracentral\|Postcentral | Motor |
| node_pheno13 | Frontal\|Supp_Motor_Area\|Temporal | Default_mode\|Salience |
| node_pheno14 | Cingulate\|Frontal | Default_mode\|Central_executive\|Salience |
| node_pheno15 | Cerebellum | Subcortical-cerebellum |
| node_pheno16 | Frontal | Salience\|Central_executive |
| node_pheno17 | Temporal | Default_mode\|Motor |
| node_pheno18 | Subcortical | Subcortical-cerebellum |
| node_pheno19 | Occipital | Visual |
| node_pheno20 | Precuneus\|Cingulate | Default_mode\|Central_executive |
| node_pheno21 | Frontal | Default_mode\|Central_executive |
| node_pheno22 | Calcarine\|Lingual\|Cuneus | Visual |
| node_pheno23 | RolandicOper\|SupraMarginal\|Insula | Salience\|Motor |
| node_pheno24 | Lingual\|Fusiform | Visual |
| node_pheno25 | Occipital | Attention\|Visual |
| node_pheno26 | Precuneus\|Angular\|Cingulate | Default_mode\|Central_executive |
| node_pheno27 | Postcentral\|Precentral | Motor |
| node_pheno28 | Frontal\|Cingulate | Default_mode\|Limbic |
| node_pheno29 | Calcarine\|Lingual | Visual |
| node_pheno30 | Temporal | Default_mode |
| node_pheno31 | OccipitalPrecuneus | Default_mode\|Central_executive |
| node_pheno32 | Angular\|Temporal | Default_mode\|Central_executive |
| node_pheno33 | Frontal\|Cerebellum | Central_executive\|Default_mode |
| node_pheno34 | Frontal_Sup | Default_mode |
| node_pheno35 | Cuneus\|Occipital | Visual |
| node_pheno36 | Insula\|Cingulate | Salience\|Default_mode |
| node_pheno37 | Occipital | Visual |
| node_pheno38 | Cerebellum | Subcortical-cerebellum |
| node_pheno39 | Parietal | Attention\|Central_executive\|Salience |
| node_pheno40 | Precuneus\|Cuneus\|Cingulate | Default_mode\|Central_executive |
| node_pheno41 | Postcentral\|Precentral | Motor\|Attention |
| node_pheno42 | Frontal_Sup | Default_mode\|Central_executive |
| node_pheno43 | Temporal_Sup | Default_mode\|Motor |
| node_pheno44 | Cerebellum | Subcortical-cerebellum |
| node_pheno45 | Parietal | Central_executive\|Attention |
| node_pheno46 | Frontal\|Precentral | Central_executive |
| node_pheno47 | Parietal\|Frontal | Attention\|Central_executive |
| node_pheno48 | Precentral\|Frontal\|Supp_Motor_Area | Attention\|Salience\|Motor |
| node_pheno49 | Supp_Motor_Area\|Frontal | Salience\|Default_mode |
| node_pheno50 | Parietal | Central_executive\|Salience |
| node_pheno51 | Paracentral | Motor |
| node_pheno52 | Frontal | Central_executive |
| node_pheno53 | Postcentral\|Precentral | Motor\|Attention |
| node_pheno54 | Temporal\|Frontal\|Supp_Motor_Area | Default_mode\|Salience |
| node_pheno55 | Parietal\|Postcentral\|Precuneus | Attention |
| node_pheno56 | Postcentral\|Precentral | Motor |
| node_pheno57 | Precuneus | Default_mode\|Central_executive |
| node_pheno58 | Frontal | Central_executive\|Salience |
| node_pheno59 | Subcortical | Subcortical-cerebellum |
| node_pheno60 | Precuneus\|Parietal_Sup | Attention\|Central_executive |
| node_pheno61 | Frontal | Central_executive |
| node_pheno62 | Temporal\|Occipital | Attention\|Visual |
| node_pheno63 | Temporal\|Occipital | Attention\|Visual |
| node_pheno64 | Temporal\|Fusiform | Limbic\|Default_mode |
| node_pheno65 | Frontal_Inf | Default_mode\|Central_executive |
| node_pheno66 | Frontal | Salience\|Default_mode |
| node_pheno67 | Temporal | Central_executive\|Default_mode |
| node_pheno68 | Frontal | Central_executive\|Salience\|Default_mode |
| node_pheno69 | Temporal | Default_mode\|Central_executive |
| node_pheno70 | Temporal_Mid\|Angular | Default_mode |
| node_pheno71 | Orbitofrontal | Limbic |
| node_pheno72 | Cerebellum\|Temporal | Subcortical-cerebellum |
| node_pheno73 | Cerebellum\|Temporal | Subcortical-cerebellum |
| node_pheno74 | Frontal | Limbic |
| node_pheno75 | Temporal\|Orbitofrontal | Limbic |
| node_pheno76 | Frontal_Sup | Limbic\|Default_mode |

**Table S3. Associations of genetically predicted life-course body weight with rsfMRI traits in univariable Mendelian randomization with IVW method.**

| **outcome** | **exposure** | **beta** | **se** | **lower 95%CI** | **upper 95%CI** | **p value** | **BH corrected p value** |
| --- | --- | --- | --- | --- | --- | --- | --- |
| edge_pheno101 | birth weight | 0.018 | 0.0323 | -0.045 | 0.082 | 5.68e-01 | 0.781009171 |
| edge_pheno1013 | birth weight | 0.002 | 0.0328 | -0.062 | 0.066 | 9.45e-01 | 0.984942154 |
| edge_pheno102 | birth weight | 0.008 | 0.0333 | -0.057 | 0.073 | 8.12e-01 | 0.893304743 |
| edge_pheno1020 | birth weight | -0.017 | 0.0320 | -0.080 | 0.046 | 5.97e-01 | 0.784483138 |
| edge_pheno1022 | birth weight | 0.017 | 0.0321 | -0.046 | 0.080 | 6.04e-01 | 0.784483138 |
| edge_pheno103 | birth weight | 0.009 | 0.0361 | -0.062 | 0.079 | 8.14e-01 | 0.893304743 |
| edge_pheno1041 | birth weight | -0.013 | 0.0371 | -0.085 | 0.060 | 7.34e-01 | 0.859787797 |
| edge_pheno1059 | birth weight | 0.023 | 0.0369 | -0.049 | 0.095 | 5.29e-01 | 0.768693083 |
| edge_pheno1122 | birth weight | -0.030 | 0.0361 | -0.101 | 0.041 | 4.04e-01 | 0.676540635 |
| edge_pheno1126 | birth weight | -0.014 | 0.0338 | -0.081 | 0.052 | 6.73e-01 | 0.819105111 |
| edge_pheno1134 | birth weight | -0.131 | 0.0374 | -0.205 | -0.058 | 4.56e-04 | 0.043557987 |
| edge_pheno1137 | birth weight | 0.045 | 0.0359 | -0.026 | 0.115 | 2.15e-01 | 0.548542485 |
| edge_pheno1141 | birth weight | -0.016 | 0.0338 | -0.082 | 0.050 | 6.32e-01 | 0.792452671 |
| edge_pheno1142 | birth weight | -0.007 | 0.0324 | -0.070 | 0.057 | 8.34e-01 | 0.904602663 |
| edge_pheno1161 | birth weight | 0.048 | 0.0379 | -0.027 | 0.122 | 2.10e-01 | 0.548542485 |
| edge_pheno1167 | birth weight | 0.066 | 0.0378 | -0.008 | 0.140 | 8.17e-02 | 0.426974142 |
| edge_pheno1171 | birth weight | -0.018 | 0.0355 | -0.087 | 0.052 | 6.17e-01 | 0.784483138 |
| edge_pheno1175 | birth weight | -0.012 | 0.0379 | -0.086 | 0.062 | 7.53e-01 | 0.871473529 |
| edge_pheno1183 | birth weight | -0.071 | 0.0319 | -0.133 | -0.008 | 2.69e-02 | 0.328662934 |
| edge_pheno1184 | birth weight | -0.025 | 0.0388 | -0.101 | 0.051 | 5.22e-01 | 0.768693083 |
| edge_pheno1189 | birth weight | -0.054 | 0.0354 | -0.123 | 0.015 | 1.27e-01 | 0.442095731 |
| edge_pheno1205 | birth weight | -0.070 | 0.0335 | -0.135 | -0.004 | 3.77e-02 | 0.328662934 |
| edge_pheno1211 | birth weight | -0.057 | 0.0354 | -0.127 | 0.012 | 1.05e-01 | 0.426974142 |
| edge_pheno1221 | birth weight | -0.018 | 0.0362 | -0.090 | 0.053 | 6.10e-01 | 0.784483138 |
| edge_pheno1225 | birth weight | 0.018 | 0.0329 | -0.046 | 0.083 | 5.82e-01 | 0.784483138 |
| edge_pheno1250 | birth weight | -0.026 | 0.0359 | -0.096 | 0.045 | 4.76e-01 | 0.747028564 |
| edge_pheno1256 | birth weight | -0.035 | 0.0336 | -0.101 | 0.031 | 2.93e-01 | 0.636739512 |
| edge_pheno1257 | birth weight | -0.064 | 0.0319 | -0.127 | -0.002 | 4.36e-02 | 0.333014358 |
| edge_pheno1269 | birth weight | 0.012 | 0.0317 | -0.050 | 0.074 | 7.05e-01 | 0.842371857 |
| edge_pheno1270 | birth weight | -0.041 | 0.0356 | -0.110 | 0.029 | 2.54e-01 | 0.613802894 |
| edge_pheno1273 | birth weight | 0.035 | 0.0355 | -0.035 | 0.104 | 3.29e-01 | 0.639683764 |
| edge_pheno1276 | birth weight | -0.018 | 0.0352 | -0.087 | 0.051 | 6.16e-01 | 0.784483138 |
| edge_pheno1293 | birth weight | 0.008 | 0.0389 | -0.068 | 0.085 | 8.27e-01 | 0.903015998 |
| edge_pheno1296 | birth weight | -0.001 | 0.0341 | -0.068 | 0.066 | 9.85e-01 | 0.995775418 |
| edge_pheno1300 | birth weight | -0.010 | 0.0366 | -0.082 | 0.061 | 7.77e-01 | 0.873029865 |
| edge_pheno1301 | birth weight | 0.012 | 0.0328 | -0.053 | 0.076 | 7.23e-01 | 0.852973014 |
| edge_pheno1302 | birth weight | -0.009 | 0.0322 | -0.073 | 0.054 | 7.71e-01 | 0.873029865 |
| edge_pheno1309 | birth weight | -0.051 | 0.0357 | -0.121 | 0.019 | 1.51e-01 | 0.471515767 |
| edge_pheno1311 | birth weight | 0.007 | 0.0380 | -0.068 | 0.081 | 8.60e-01 | 0.92265667 |
| edge_pheno1317 | birth weight | -0.023 | 0.0437 | -0.109 | 0.062 | 5.93e-01 | 0.784483138 |
| edge_pheno1319 | birth weight | 0.023 | 0.0318 | -0.040 | 0.085 | 4.77e-01 | 0.747028564 |
| edge_pheno132 | birth weight | 0.002 | 0.0344 | -0.065 | 0.069 | 9.53e-01 | 0.984942154 |
| edge_pheno1322 | birth weight | 0.029 | 0.0332 | -0.036 | 0.094 | 3.78e-01 | 0.673327074 |
| edge_pheno1325 | birth weight | 0.037 | 0.0339 | -0.030 | 0.103 | 2.81e-01 | 0.626833844 |
| edge_pheno1328 | birth weight | -0.057 | 0.0374 | -0.131 | 0.016 | 1.24e-01 | 0.442095731 |
| edge_pheno135 | birth weight | 0.033 | 0.0333 | -0.032 | 0.099 | 3.18e-01 | 0.639683764 |
| edge_pheno1359 | birth weight | 0.036 | 0.0369 | -0.037 | 0.108 | 3.33e-01 | 0.639683764 |
| edge_pheno1382 | birth weight | -0.048 | 0.0348 | -0.116 | 0.020 | 1.66e-01 | 0.504020055 |
| edge_pheno146 | birth weight | 0.035 | 0.0335 | -0.031 | 0.101 | 2.97e-01 | 0.638186265 |
| edge_pheno147 | birth weight | 0.032 | 0.0326 | -0.032 | 0.096 | 3.24e-01 | 0.639683764 |
| edge_pheno151 | birth weight | -0.029 | 0.0334 | -0.095 | 0.036 | 3.80e-01 | 0.673327074 |
| edge_pheno1696 | birth weight | -0.021 | 0.0359 | -0.091 | 0.050 | 5.68e-01 | 0.781009171 |
| edge_pheno1697 | birth weight | -0.053 | 0.0408 | -0.133 | 0.028 | 1.98e-01 | 0.531133843 |
| edge_pheno1698 | birth weight | -0.019 | 0.0383 | -0.094 | 0.056 | 6.19e-01 | 0.784483138 |
| edge_pheno1699 | birth weight | -0.061 | 0.0387 | -0.137 | 0.014 | 1.12e-01 | 0.429118736 |
| edge_pheno1701 | birth weight | 0.089 | 0.0386 | 0.014 | 0.165 | 2.07e-02 | 0.328662934 |
| edge_pheno249 | birth weight | 0.000 | 0.0354 | -0.069 | 0.070 | 9.91e-01 | 0.995775418 |
| edge_pheno253 | birth weight | 0.021 | 0.0320 | -0.042 | 0.084 | 5.12e-01 | 0.768693083 |
| edge_pheno262 | birth weight | 0.035 | 0.0317 | -0.027 | 0.097 | 2.73e-01 | 0.626833844 |
| edge_pheno286 | birth weight | -0.028 | 0.0331 | -0.093 | 0.036 | 3.90e-01 | 0.675859582 |
| edge_pheno288 | birth weight | 0.042 | 0.0320 | -0.021 | 0.105 | 1.88e-01 | 0.519750272 |
| edge_pheno303 | birth weight | -0.027 | 0.0349 | -0.095 | 0.042 | 4.44e-01 | 0.713430473 |
| edge_pheno389 | birth weight | 0.049 | 0.0334 | -0.016 | 0.114 | 1.42e-01 | 0.466088029 |
| edge_pheno405 | birth weight | -0.031 | 0.0314 | -0.093 | 0.030 | 3.17e-01 | 0.639683764 |
| edge_pheno447 | birth weight | 0.157 | 0.1209 | -0.080 | 0.394 | 1.93e-01 | 0.526881838 |
| edge_pheno449 | birth weight | -0.182 | 0.1356 | -0.447 | 0.084 | 1.80e-01 | 0.5141785 |
| edge_pheno460 | birth weight | 0.009 | 0.0361 | -0.062 | 0.080 | 8.00e-01 | 0.888203779 |
| edge_pheno461 | birth weight | -0.002 | 0.0358 | -0.072 | 0.068 | 9.57e-01 | 0.984942154 |
| edge_pheno491 | birth weight | 0.048 | 0.0327 | -0.017 | 0.112 | 1.46e-01 | 0.466088029 |
| edge_pheno537 | birth weight | -0.070 | 0.0351 | -0.139 | -0.001 | 4.59e-02 | 0.333473418 |
| edge_pheno55 | birth weight | 0.022 | 0.0360 | -0.049 | 0.092 | 5.45e-01 | 0.771039514 |
| edge_pheno558 | birth weight | 0.016 | 0.0331 | -0.048 | 0.081 | 6.19e-01 | 0.784483138 |
| edge_pheno574 | birth weight | 0.012 | 0.0316 | -0.050 | 0.074 | 7.00e-01 | 0.842371857 |
| edge_pheno58 | birth weight | 0.024 | 0.0307 | -0.036 | 0.084 | 4.38e-01 | 0.709654013 |
| edge_pheno590 | birth weight | 0.022 | 0.0353 | -0.047 | 0.092 | 5.26e-01 | 0.768693083 |
| edge_pheno593 | birth weight | -0.050 | 0.0334 | -0.116 | 0.015 | 1.30e-01 | 0.444616125 |
| edge_pheno597 | birth weight | -0.042 | 0.0378 | -0.116 | 0.032 | 2.67e-01 | 0.621127136 |
| edge_pheno599 | birth weight | 0.016 | 0.0330 | -0.049 | 0.080 | 6.35e-01 | 0.792452671 |
| edge_pheno601 | birth weight | -0.028 | 0.0319 | -0.091 | 0.034 | 3.76e-01 | 0.673327074 |
| edge_pheno606 | birth weight | -0.009 | 0.0328 | -0.073 | 0.055 | 7.77e-01 | 0.873029865 |
| edge_pheno609 | birth weight | -0.018 | 0.0350 | -0.086 | 0.051 | 6.15e-01 | 0.784483138 |
| edge_pheno621 | birth weight | 0.022 | 0.0334 | -0.043 | 0.088 | 5.04e-01 | 0.768693083 |
| edge_pheno624 | birth weight | 0.021 | 0.0325 | -0.043 | 0.084 | 5.24e-01 | 0.768693083 |
| edge_pheno636 | birth weight | -0.027 | 0.0320 | -0.090 | 0.036 | 3.99e-01 | 0.675859582 |
| edge_pheno639 | birth weight | 0.009 | 0.0332 | -0.056 | 0.074 | 7.90e-01 | 0.882685093 |
| edge_pheno65 | birth weight | -0.059 | 0.0367 | -0.131 | 0.013 | 1.07e-01 | 0.426974142 |
| edge_pheno66 | birth weight | 0.023 | 0.0353 | -0.046 | 0.092 | 5.12e-01 | 0.768693083 |
| edge_pheno674 | birth weight | 0.095 | 0.0334 | 0.030 | 0.160 | 4.43e-03 | 0.208600247 |
| edge_pheno681 | birth weight | 0.034 | 0.0366 | -0.037 | 0.106 | 3.49e-01 | 0.640154779 |
| edge_pheno683 | birth weight | 0.031 | 0.0359 | -0.040 | 0.101 | 3.94e-01 | 0.675859582 |
| edge_pheno695 | birth weight | 0.027 | 0.0334 | -0.038 | 0.093 | 4.11e-01 | 0.676540635 |
| edge_pheno698 | birth weight | -0.003 | 0.0324 | -0.066 | 0.061 | 9.32e-01 | 0.983178838 |
| edge_pheno705 | birth weight | 0.039 | 0.0355 | -0.031 | 0.108 | 2.77e-01 | 0.626833844 |
| edge_pheno716 | birth weight | 0.003 | 0.0333 | -0.062 | 0.069 | 9.18e-01 | 0.974228531 |
| edge_pheno767 | birth weight | 0.021 | 0.0352 | -0.049 | 0.090 | 5.61e-01 | 0.781009171 |
| edge_pheno777 | birth weight | 0.022 | 0.0349 | -0.047 | 0.090 | 5.31e-01 | 0.768693083 |
| edge_pheno789 | birth weight | 0.029 | 0.0331 | -0.036 | 0.094 | 3.84e-01 | 0.673327074 |
| edge_pheno794 | birth weight | -0.045 | 0.0323 | -0.109 | 0.018 | 1.60e-01 | 0.492999311 |
| edge_pheno801 | birth weight | -0.012 | 0.0325 | -0.076 | 0.052 | 7.13e-01 | 0.845341286 |
| edge_pheno810 | birth weight | 0.020 | 0.0340 | -0.046 | 0.087 | 5.51e-01 | 0.77410582 |
| edge_pheno812 | birth weight | -0.022 | 0.0330 | -0.087 | 0.042 | 4.98e-01 | 0.768693083 |
| edge_pheno815 | birth weight | 0.065 | 0.0395 | -0.013 | 0.142 | 1.01e-01 | 0.426974142 |
| edge_pheno816 | birth weight | -0.063 | 0.0385 | -0.139 | 0.012 | 9.97e-02 | 0.426974142 |
| edge_pheno824 | birth weight | -0.022 | 0.0366 | -0.094 | 0.049 | 5.42e-01 | 0.771039514 |
| edge_pheno867 | birth weight | -0.022 | 0.0350 | -0.091 | 0.046 | 5.25e-01 | 0.768693083 |
| edge_pheno869 | birth weight | 0.034 | 0.0387 | -0.042 | 0.110 | 3.83e-01 | 0.673327074 |
| edge_pheno87 | birth weight | -0.039 | 0.0374 | -0.113 | 0.034 | 2.92e-01 | 0.636739512 |
| edge_pheno882 | birth weight | 0.032 | 0.0334 | -0.034 | 0.097 | 3.45e-01 | 0.639895559 |
| edge_pheno899 | birth weight | -0.038 | 0.0400 | -0.116 | 0.041 | 3.45e-01 | 0.639895559 |
| edge_pheno903 | birth weight | -0.057 | 0.0352 | -0.126 | 0.012 | 1.03e-01 | 0.426974142 |
| edge_pheno908 | birth weight | 0.002 | 0.0340 | -0.065 | 0.068 | 9.59e-01 | 0.984942154 |
| edge_pheno918 | birth weight | 0.022 | 0.0359 | -0.049 | 0.092 | 5.44e-01 | 0.771039514 |
| edge_pheno932 | birth weight | -0.058 | 0.0338 | -0.124 | 0.008 | 8.52e-02 | 0.426974142 |
| edge_pheno942 | birth weight | 0.036 | 0.0366 | -0.036 | 0.107 | 3.31e-01 | 0.639683764 |
| edge_pheno956 | birth weight | -0.056 | 0.0353 | -0.126 | 0.013 | 1.10e-01 | 0.426974142 |
| edge_pheno965 | birth weight | -0.042 | 0.0336 | -0.107 | 0.024 | 2.15e-01 | 0.548542485 |
| node_pheno1 | birth weight | -0.018 | 0.0368 | -0.090 | 0.054 | 6.20e-01 | 0.784483138 |
| node_pheno10 | birth weight | -0.073 | 0.0332 | -0.139 | -0.008 | 2.71e-02 | 0.328662934 |
| node_pheno11 | birth weight | -0.030 | 0.0315 | -0.092 | 0.031 | 3.37e-01 | 0.639683764 |
| node_pheno12 | birth weight | -0.060 | 0.0346 | -0.128 | 0.008 | 8.21e-02 | 0.426974142 |
| node_pheno13 | birth weight | -0.042 | 0.0352 | -0.111 | 0.027 | 2.29e-01 | 0.576430032 |
| node_pheno14 | birth weight | -0.044 | 0.0329 | -0.109 | 0.020 | 1.80e-01 | 0.5141785 |
| node_pheno15 | birth weight | -0.040 | 0.0310 | -0.100 | 0.021 | 2.00e-01 | 0.531133843 |
| node_pheno16 | birth weight | -0.052 | 0.0306 | -0.112 | 0.008 | 8.82e-02 | 0.426974142 |
| node_pheno17 | birth weight | -0.055 | 0.0334 | -0.121 | 0.010 | 9.70e-02 | 0.426974142 |
| node_pheno18 | birth weight | -0.070 | 0.0342 | -0.137 | -0.003 | 4.07e-02 | 0.328662934 |
| node_pheno19 | birth weight | -0.024 | 0.0307 | -0.084 | 0.036 | 4.35e-01 | 0.709654013 |
| node_pheno2 | birth weight | -0.050 | 0.0312 | -0.111 | 0.011 | 1.08e-01 | 0.426974142 |
| node_pheno20 | birth weight | -0.079 | 0.0334 | -0.145 | -0.014 | 1.73e-02 | 0.328662934 |
| node_pheno21 | birth weight | -0.039 | 0.0331 | -0.104 | 0.026 | 2.40e-01 | 0.587617848 |
| node_pheno22 | birth weight | -0.044 | 0.0328 | -0.108 | 0.021 | 1.84e-01 | 0.516853269 |
| node_pheno23 | birth weight | -0.057 | 0.0365 | -0.128 | 0.015 | 1.21e-01 | 0.442095731 |
| node_pheno24 | birth weight | -0.029 | 0.0339 | -0.095 | 0.038 | 4.00e-01 | 0.675859582 |
| node_pheno25 | birth weight | -0.071 | 0.0346 | -0.138 | -0.003 | 4.13e-02 | 0.328662934 |
| node_pheno26 | birth weight | -0.067 | 0.0311 | -0.128 | -0.006 | 3.13e-02 | 0.328662934 |
| node_pheno27 | birth weight | -0.054 | 0.0348 | -0.122 | 0.014 | 1.22e-01 | 0.442095731 |
| node_pheno28 | birth weight | -0.003 | 0.0306 | -0.063 | 0.057 | 9.14e-01 | 0.974228531 |
| node_pheno29 | birth weight | -0.015 | 0.0331 | -0.080 | 0.050 | 6.52e-01 | 0.804035602 |
| node_pheno3 | birth weight | -0.087 | 0.0383 | -0.162 | -0.012 | 2.35e-02 | 0.328662934 |
| node_pheno30 | birth weight | -0.048 | 0.0355 | -0.118 | 0.022 | 1.76e-01 | 0.5141785 |
| node_pheno31 | birth weight | -0.006 | 0.0310 | -0.067 | 0.055 | 8.40e-01 | 0.906641516 |
| node_pheno32 | birth weight | -0.061 | 0.0326 | -0.125 | 0.003 | 6.08e-02 | 0.400311248 |
| node_pheno33 | birth weight | -0.086 | 0.0342 | -0.153 | -0.019 | 1.21e-02 | 0.288412813 |
| node_pheno34 | birth weight | 0.011 | 0.0363 | -0.060 | 0.083 | 7.52e-01 | 0.871473529 |
| node_pheno35 | birth weight | 0.002 | 0.0297 | -0.057 | 0.060 | 9.56e-01 | 0.984942154 |
| node_pheno36 | birth weight | -0.054 | 0.0319 | -0.116 | 0.009 | 9.32e-02 | 0.426974142 |
| node_pheno37 | birth weight | -0.023 | 0.0305 | -0.082 | 0.037 | 4.58e-01 | 0.729058078 |
| node_pheno38 | birth weight | -0.054 | 0.0321 | -0.117 | 0.009 | 9.41e-02 | 0.426974142 |
| node_pheno39 | birth weight | -0.074 | 0.0357 | -0.144 | -0.004 | 3.78e-02 | 0.328662934 |
| node_pheno4 | birth weight | -0.048 | 0.0328 | -0.112 | 0.017 | 1.46e-01 | 0.466088029 |
| node_pheno40 | birth weight | -0.071 | 0.0340 | -0.137 | -0.004 | 3.68e-02 | 0.328662934 |
| node_pheno41 | birth weight | -0.087 | 0.0362 | -0.158 | -0.016 | 1.61e-02 | 0.328662934 |
| node_pheno42 | birth weight | -0.014 | 0.0372 | -0.087 | 0.059 | 7.06e-01 | 0.842371857 |
| node_pheno43 | birth weight | -0.058 | 0.0343 | -0.125 | 0.010 | 9.33e-02 | 0.426974142 |
| node_pheno44 | birth weight | -0.065 | 0.0298 | -0.124 | -0.007 | 2.82e-02 | 0.328662934 |
| node_pheno45 | birth weight | -0.079 | 0.0353 | -0.148 | -0.010 | 2.45e-02 | 0.328662934 |
| node_pheno46 | birth weight | -0.040 | 0.0400 | -0.119 | 0.038 | 3.12e-01 | 0.639683764 |
| node_pheno47 | birth weight | -0.059 | 0.0337 | -0.125 | 0.007 | 8.08e-02 | 0.426974142 |
| node_pheno48 | birth weight | -0.038 | 0.0372 | -0.111 | 0.035 | 3.07e-01 | 0.639683764 |
| node_pheno49 | birth weight | -0.057 | 0.0346 | -0.124 | 0.011 | 1.01e-01 | 0.426974142 |
| node_pheno5 | birth weight | -0.064 | 0.0312 | -0.126 | -0.003 | 3.91e-02 | 0.328662934 |
| node_pheno50 | birth weight | -0.051 | 0.0301 | -0.110 | 0.008 | 8.96e-02 | 0.426974142 |
| node_pheno51 | birth weight | -0.033 | 0.0339 | -0.099 | 0.034 | 3.32e-01 | 0.639683764 |
| node_pheno52 | birth weight | -0.090 | 0.0325 | -0.154 | -0.027 | 5.46e-03 | 0.208600247 |
| node_pheno53 | birth weight | -0.096 | 0.0357 | -0.166 | -0.026 | 7.13e-03 | 0.225182056 |
| node_pheno54 | birth weight | -0.035 | 0.0327 | -0.099 | 0.029 | 2.82e-01 | 0.626833844 |
| node_pheno55 | birth weight | -0.069 | 0.0348 | -0.137 | -0.001 | 4.71e-02 | 0.333473418 |
| node_pheno56 | birth weight | -0.053 | 0.0391 | -0.129 | 0.024 | 1.76e-01 | 0.5141785 |
| node_pheno57 | birth weight | -0.046 | 0.0309 | -0.107 | 0.014 | 1.33e-01 | 0.446353597 |
| node_pheno58 | birth weight | -0.055 | 0.0325 | -0.118 | 0.009 | 9.14e-02 | 0.426974142 |
| node_pheno59 | birth weight | -0.063 | 0.0308 | -0.123 | -0.003 | 4.05e-02 | 0.328662934 |
| node_pheno60 | birth weight | -0.096 | 0.0364 | -0.167 | -0.025 | 8.25e-03 | 0.225182056 |
| node_pheno61 | birth weight | -0.016 | 0.0366 | -0.088 | 0.056 | 6.61e-01 | 0.808701751 |
| node_pheno62 | birth weight | 0.010 | 0.0332 | -0.056 | 0.075 | 7.73e-01 | 0.873029865 |
| node_pheno63 | birth weight | -0.038 | 0.0317 | -0.100 | 0.024 | 2.34e-01 | 0.581404509 |
| node_pheno64 | birth weight | -0.034 | 0.0340 | -0.101 | 0.033 | 3.19e-01 | 0.639683764 |
| node_pheno65 | birth weight | -0.051 | 0.0330 | -0.115 | 0.014 | 1.25e-01 | 0.442095731 |
| node_pheno66 | birth weight | -0.037 | 0.0333 | -0.102 | 0.028 | 2.66e-01 | 0.621127136 |
| node_pheno67 | birth weight | -0.147 | 0.0356 | -0.217 | -0.078 | 3.47e-05 | 0.006620221 |
| node_pheno68 | birth weight | 0.015 | 0.0324 | -0.049 | 0.078 | 6.51e-01 | 0.804035602 |
| node_pheno69 | birth weight | -0.108 | 0.0379 | -0.183 | -0.034 | 4.21e-03 | 0.208600247 |
| node_pheno7 | birth weight | 0.001 | 0.0360 | -0.070 | 0.071 | 9.87e-01 | 0.995775418 |
| node_pheno70 | birth weight | 0.000 | 0.0337 | -0.066 | 0.066 | 9.99e-01 | 0.999212946 |
| node_pheno71 | birth weight | -0.017 | 0.0318 | -0.080 | 0.045 | 5.84e-01 | 0.784483138 |
| node_pheno72 | birth weight | -0.032 | 0.0333 | -0.097 | 0.033 | 3.38e-01 | 0.639683764 |
| node_pheno73 | birth weight | -0.051 | 0.0301 | -0.110 | 0.008 | 8.85e-02 | 0.426974142 |
| node_pheno74 | birth weight | -0.029 | 0.0348 | -0.097 | 0.039 | 4.08e-01 | 0.676540635 |
| node_pheno75 | birth weight | -0.035 | 0.0310 | -0.095 | 0.026 | 2.66e-01 | 0.621127136 |
| node_pheno76 | birth weight | 0.000 | 0.0289 | -0.057 | 0.056 | 9.89e-01 | 0.995775418 |
| node_pheno8 | birth weight | -0.009 | 0.0304 | -0.068 | 0.051 | 7.73e-01 | 0.873029865 |
| node_pheno9 | birth weight | -0.064 | 0.0326 | -0.128 | 0.000 | 4.98e-02 | 0.339797518 |
| edge_pheno101 | childhood_BMI | -0.058 | 0.0342 | -0.125 | 0.009 | 8.84e-02 | 0.486924804 |
| edge_pheno1013 | childhood_BMI | -0.002 | 0.0309 | -0.063 | 0.058 | 9.44e-01 | 0.975128003 |
| edge_pheno102 | childhood_BMI | -0.063 | 0.0275 | -0.117 | -0.009 | 2.22e-02 | 0.274978068 |
| edge_pheno1020 | childhood_BMI | 0.024 | 0.0276 | -0.030 | 0.079 | 3.74e-01 | 0.802264449 |
| edge_pheno1022 | childhood_BMI | 0.007 | 0.0298 | -0.051 | 0.065 | 8.15e-01 | 0.958241885 |
| edge_pheno103 | childhood_BMI | -0.049 | 0.0270 | -0.102 | 0.004 | 6.87e-02 | 0.471779995 |
| edge_pheno1041 | childhood_BMI | 0.013 | 0.0274 | -0.041 | 0.067 | 6.37e-01 | 0.900920809 |
| edge_pheno1059 | childhood_BMI | -0.046 | 0.0274 | -0.100 | 0.008 | 9.20e-02 | 0.486924804 |
| edge_pheno1122 | childhood_BMI | -0.068 | 0.0346 | -0.136 | 0.000 | 4.92e-02 | 0.390400558 |
| edge_pheno1126 | childhood_BMI | -0.042 | 0.0279 | -0.097 | 0.012 | 1.29e-01 | 0.530912634 |
| edge_pheno1134 | childhood_BMI | -0.017 | 0.0275 | -0.071 | 0.036 | 5.27e-01 | 0.843109619 |
| edge_pheno1137 | childhood_BMI | -0.034 | 0.0273 | -0.087 | 0.020 | 2.16e-01 | 0.666251865 |
| edge_pheno1141 | childhood_BMI | -0.077 | 0.0272 | -0.130 | -0.024 | 4.55e-03 | 0.108750058 |
| edge_pheno1142 | childhood_BMI | -0.019 | 0.0274 | -0.073 | 0.035 | 4.94e-01 | 0.828836593 |
| edge_pheno1161 | childhood_BMI | -0.021 | 0.0331 | -0.086 | 0.044 | 5.34e-01 | 0.84585362 |
| edge_pheno1167 | childhood_BMI | -0.019 | 0.0314 | -0.080 | 0.043 | 5.52e-01 | 0.853067207 |
| edge_pheno1171 | childhood_BMI | 0.014 | 0.0269 | -0.039 | 0.067 | 6.08e-01 | 0.880763225 |
| edge_pheno1175 | childhood_BMI | 0.010 | 0.0292 | -0.047 | 0.067 | 7.27e-01 | 0.956764954 |
| edge_pheno1183 | childhood_BMI | 0.016 | 0.0286 | -0.040 | 0.072 | 5.68e-01 | 0.853067207 |
| edge_pheno1184 | childhood_BMI | 0.007 | 0.0332 | -0.058 | 0.072 | 8.30e-01 | 0.958241885 |
| edge_pheno1189 | childhood_BMI | -0.008 | 0.0330 | -0.072 | 0.057 | 8.16e-01 | 0.958241885 |
| edge_pheno1205 | childhood_BMI | 0.003 | 0.0269 | -0.050 | 0.055 | 9.25e-01 | 0.970919907 |
| edge_pheno1211 | childhood_BMI | -0.008 | 0.0282 | -0.063 | 0.047 | 7.80e-01 | 0.958241885 |
| edge_pheno1221 | childhood_BMI | 0.001 | 0.0271 | -0.052 | 0.054 | 9.80e-01 | 0.992440173 |
| edge_pheno1225 | childhood_BMI | 0.011 | 0.0324 | -0.052 | 0.075 | 7.31e-01 | 0.956764954 |
| edge_pheno1250 | childhood_BMI | -0.010 | 0.0310 | -0.071 | 0.051 | 7.41e-01 | 0.958241885 |
| edge_pheno1256 | childhood_BMI | -0.051 | 0.0305 | -0.111 | 0.008 | 9.25e-02 | 0.486924804 |
| edge_pheno1257 | childhood_BMI | -0.007 | 0.0345 | -0.074 | 0.061 | 8.46e-01 | 0.958241885 |
| edge_pheno1269 | childhood_BMI | -0.029 | 0.0299 | -0.087 | 0.030 | 3.34e-01 | 0.763375435 |
| edge_pheno1270 | childhood_BMI | 0.005 | 0.0325 | -0.059 | 0.069 | 8.75e-01 | 0.958241885 |
| edge_pheno1273 | childhood_BMI | 0.044 | 0.0307 | -0.016 | 0.104 | 1.48e-01 | 0.564634214 |
| edge_pheno1276 | childhood_BMI | 0.005 | 0.0265 | -0.047 | 0.056 | 8.65e-01 | 0.958241885 |
| edge_pheno1293 | childhood_BMI | 0.023 | 0.0310 | -0.038 | 0.084 | 4.57e-01 | 0.816249798 |
| edge_pheno1296 | childhood_BMI | -0.034 | 0.0300 | -0.093 | 0.025 | 2.59e-01 | 0.733013865 |
| edge_pheno1300 | childhood_BMI | 0.011 | 0.0280 | -0.043 | 0.066 | 6.83e-01 | 0.924814508 |
| edge_pheno1301 | childhood_BMI | -0.026 | 0.0330 | -0.091 | 0.039 | 4.29e-01 | 0.813746745 |
| edge_pheno1302 | childhood_BMI | -0.015 | 0.0272 | -0.069 | 0.038 | 5.69e-01 | 0.853067207 |
| edge_pheno1309 | childhood_BMI | -0.006 | 0.0276 | -0.060 | 0.048 | 8.37e-01 | 0.958241885 |
| edge_pheno1311 | childhood_BMI | -0.028 | 0.0321 | -0.091 | 0.035 | 3.83e-01 | 0.802264449 |
| edge_pheno1317 | childhood_BMI | -0.027 | 0.0349 | -0.095 | 0.041 | 4.37e-01 | 0.813746745 |
| edge_pheno1319 | childhood_BMI | 0.058 | 0.0349 | -0.011 | 0.126 | 9.81e-02 | 0.486924804 |
| edge_pheno132 | childhood_BMI | 0.001 | 0.0274 | -0.052 | 0.055 | 9.59e-01 | 0.976467254 |
| edge_pheno1322 | childhood_BMI | -0.003 | 0.0274 | -0.057 | 0.051 | 9.16e-01 | 0.970919907 |
| edge_pheno1325 | childhood_BMI | -0.007 | 0.0275 | -0.061 | 0.047 | 8.08e-01 | 0.958241885 |
| edge_pheno1328 | childhood_BMI | 0.032 | 0.0275 | -0.022 | 0.086 | 2.46e-01 | 0.715628738 |
| edge_pheno135 | childhood_BMI | -0.028 | 0.0274 | -0.081 | 0.026 | 3.10e-01 | 0.754858317 |
| edge_pheno1359 | childhood_BMI | 0.019 | 0.0272 | -0.034 | 0.073 | 4.75e-01 | 0.821100715 |
| edge_pheno1382 | childhood_BMI | 0.024 | 0.0270 | -0.028 | 0.077 | 3.63e-01 | 0.800194823 |
| edge_pheno146 | childhood_BMI | -0.021 | 0.0320 | -0.083 | 0.042 | 5.16e-01 | 0.834490969 |
| edge_pheno147 | childhood_BMI | -0.070 | 0.0276 | -0.124 | -0.016 | 1.07e-02 | 0.183722413 |
| edge_pheno151 | childhood_BMI | 0.046 | 0.0273 | -0.008 | 0.099 | 9.56e-02 | 0.486924804 |
| edge_pheno1696 | childhood_BMI | 0.049 | 0.0276 | -0.005 | 0.103 | 7.49e-02 | 0.486924804 |
| edge_pheno1697 | childhood_BMI | -0.022 | 0.0266 | -0.074 | 0.031 | 4.20e-01 | 0.813746745 |
| edge_pheno1698 | childhood_BMI | 0.018 | 0.0273 | -0.035 | 0.072 | 5.04e-01 | 0.828836593 |
| edge_pheno1699 | childhood_BMI | 0.005 | 0.0294 | -0.052 | 0.063 | 8.52e-01 | 0.958241885 |
| edge_pheno1701 | childhood_BMI | 0.008 | 0.0344 | -0.059 | 0.076 | 8.07e-01 | 0.958241885 |
| edge_pheno249 | childhood_BMI | 0.020 | 0.0290 | -0.037 | 0.077 | 4.98e-01 | 0.828836593 |
| edge_pheno253 | childhood_BMI | 0.002 | 0.0276 | -0.052 | 0.057 | 9.29e-01 | 0.970919907 |
| edge_pheno262 | childhood_BMI | -0.013 | 0.0304 | -0.073 | 0.047 | 6.69e-01 | 0.92292935 |
| edge_pheno286 | childhood_BMI | -0.031 | 0.0325 | -0.095 | 0.032 | 3.34e-01 | 0.763375435 |
| edge_pheno288 | childhood_BMI | 0.005 | 0.0276 | -0.049 | 0.059 | 8.44e-01 | 0.958241885 |
| edge_pheno303 | childhood_BMI | -0.010 | 0.0288 | -0.066 | 0.047 | 7.40e-01 | 0.958241885 |
| edge_pheno389 | childhood_BMI | -0.045 | 0.0277 | -0.099 | 0.010 | 1.07e-01 | 0.512456707 |
| edge_pheno405 | childhood_BMI | 0.042 | 0.0319 | -0.021 | 0.105 | 1.89e-01 | 0.643404717 |
| edge_pheno447 | childhood_BMI | -0.055 | 0.0716 | -0.195 | 0.086 | 4.44e-01 | 0.813746745 |
| edge_pheno449 | childhood_BMI | -0.016 | 0.0723 | -0.157 | 0.126 | 8.29e-01 | 0.958241885 |
| edge_pheno460 | childhood_BMI | 0.022 | 0.0279 | -0.032 | 0.077 | 4.22e-01 | 0.813746745 |
| edge_pheno461 | childhood_BMI | -0.023 | 0.0274 | -0.077 | 0.030 | 3.93e-01 | 0.806929704 |
| edge_pheno491 | childhood_BMI | -0.001 | 0.0275 | -0.055 | 0.053 | 9.80e-01 | 0.992440173 |
| edge_pheno537 | childhood_BMI | -0.026 | 0.0267 | -0.079 | 0.026 | 3.25e-01 | 0.757790853 |
| edge_pheno55 | childhood_BMI | 0.045 | 0.0299 | -0.014 | 0.103 | 1.35e-01 | 0.53810511 |
| edge_pheno558 | childhood_BMI | -0.025 | 0.0306 | -0.085 | 0.035 | 4.20e-01 | 0.813746745 |
| edge_pheno574 | childhood_BMI | -0.092 | 0.0270 | -0.145 | -0.040 | 6.15e-04 | 0.076392839 |
| edge_pheno58 | childhood_BMI | -0.020 | 0.0272 | -0.073 | 0.033 | 4.62e-01 | 0.816249798 |
| edge_pheno590 | childhood_BMI | 0.007 | 0.0286 | -0.049 | 0.064 | 7.96e-01 | 0.958241885 |
| edge_pheno593 | childhood_BMI | 0.011 | 0.0273 | -0.042 | 0.065 | 6.84e-01 | 0.924814508 |
| edge_pheno597 | childhood_BMI | 0.006 | 0.0271 | -0.047 | 0.059 | 8.13e-01 | 0.958241885 |
| edge_pheno599 | childhood_BMI | 0.005 | 0.0274 | -0.049 | 0.059 | 8.62e-01 | 0.958241885 |
| edge_pheno601 | childhood_BMI | 0.009 | 0.0313 | -0.052 | 0.071 | 7.64e-01 | 0.958241885 |
| edge_pheno606 | childhood_BMI | -0.030 | 0.0293 | -0.088 | 0.027 | 2.98e-01 | 0.746547599 |
| edge_pheno609 | childhood_BMI | -0.016 | 0.0285 | -0.072 | 0.039 | 5.67e-01 | 0.853067207 |
| edge_pheno621 | childhood_BMI | -0.007 | 0.0286 | -0.063 | 0.049 | 8.01e-01 | 0.958241885 |
| edge_pheno624 | childhood_BMI | -0.002 | 0.0275 | -0.055 | 0.052 | 9.56e-01 | 0.976467254 |
| edge_pheno636 | childhood_BMI | -0.007 | 0.0316 | -0.069 | 0.055 | 8.24e-01 | 0.958241885 |
| edge_pheno639 | childhood_BMI | -0.038 | 0.0271 | -0.091 | 0.015 | 1.61e-01 | 0.587251108 |
| edge_pheno65 | childhood_BMI | 0.049 | 0.0274 | -0.004 | 0.103 | 7.08e-02 | 0.47471619 |
| edge_pheno66 | childhood_BMI | 0.006 | 0.0288 | -0.050 | 0.063 | 8.29e-01 | 0.958241885 |
| edge_pheno674 | childhood_BMI | 0.010 | 0.0278 | -0.045 | 0.064 | 7.22e-01 | 0.956764954 |
| edge_pheno681 | childhood_BMI | 0.061 | 0.0276 | 0.007 | 0.116 | 2.59e-02 | 0.274978068 |
| edge_pheno683 | childhood_BMI | -0.008 | 0.0275 | -0.062 | 0.046 | 7.66e-01 | 0.958241885 |
| edge_pheno695 | childhood_BMI | 0.023 | 0.0274 | -0.031 | 0.076 | 4.08e-01 | 0.813746745 |
| edge_pheno698 | childhood_BMI | -0.087 | 0.0287 | -0.144 | -0.031 | 2.40e-03 | 0.076392839 |
| edge_pheno705 | childhood_BMI | 0.018 | 0.0288 | -0.038 | 0.075 | 5.30e-01 | 0.843109619 |
| edge_pheno716 | childhood_BMI | -0.044 | 0.0275 | -0.098 | 0.009 | 1.05e-01 | 0.508933678 |
| edge_pheno767 | childhood_BMI | -0.024 | 0.0308 | -0.084 | 0.037 | 4.41e-01 | 0.813746745 |
| edge_pheno777 | childhood_BMI | -0.061 | 0.0307 | -0.121 | -0.001 | 4.56e-02 | 0.378292571 |
| edge_pheno789 | childhood_BMI | 0.052 | 0.0268 | -0.001 | 0.105 | 5.24e-02 | 0.392804542 |
| edge_pheno794 | childhood_BMI | -0.067 | 0.0270 | -0.120 | -0.014 | 1.28e-02 | 0.189626828 |
| edge_pheno801 | childhood_BMI | 0.022 | 0.0330 | -0.042 | 0.087 | 4.96e-01 | 0.828836593 |
| edge_pheno810 | childhood_BMI | 0.013 | 0.0265 | -0.039 | 0.065 | 6.31e-01 | 0.900029003 |
| edge_pheno812 | childhood_BMI | 0.000 | 0.0274 | -0.054 | 0.054 | 9.97e-01 | 0.996646331 |
| edge_pheno815 | childhood_BMI | -0.020 | 0.0272 | -0.073 | 0.034 | 4.70e-01 | 0.818980792 |
| edge_pheno816 | childhood_BMI | 0.000 | 0.0265 | -0.052 | 0.052 | 9.95e-01 | 0.996646331 |
| edge_pheno824 | childhood_BMI | 0.039 | 0.0330 | -0.026 | 0.103 | 2.41e-01 | 0.706744706 |
| edge_pheno867 | childhood_BMI | -0.002 | 0.0288 | -0.058 | 0.055 | 9.54e-01 | 0.976467254 |
| edge_pheno869 | childhood_BMI | -0.006 | 0.0352 | -0.075 | 0.063 | 8.69e-01 | 0.958241885 |
| edge_pheno87 | childhood_BMI | 0.005 | 0.0335 | -0.061 | 0.070 | 8.85e-01 | 0.960570209 |
| edge_pheno882 | childhood_BMI | -0.012 | 0.0315 | -0.074 | 0.050 | 7.04e-01 | 0.944069706 |
| edge_pheno899 | childhood_BMI | 0.028 | 0.0275 | -0.026 | 0.082 | 3.14e-01 | 0.754858317 |
| edge_pheno903 | childhood_BMI | 0.012 | 0.0273 | -0.042 | 0.065 | 6.72e-01 | 0.92292935 |
| edge_pheno908 | childhood_BMI | 0.027 | 0.0275 | -0.027 | 0.081 | 3.25e-01 | 0.757790853 |
| edge_pheno918 | childhood_BMI | 0.005 | 0.0280 | -0.050 | 0.060 | 8.62e-01 | 0.958241885 |
| edge_pheno932 | childhood_BMI | 0.021 | 0.0268 | -0.032 | 0.073 | 4.37e-01 | 0.813746745 |
| edge_pheno942 | childhood_BMI | -0.013 | 0.0271 | -0.066 | 0.040 | 6.39e-01 | 0.900920809 |
| edge_pheno956 | childhood_BMI | -0.038 | 0.0327 | -0.102 | 0.026 | 2.48e-01 | 0.715628738 |
| edge_pheno965 | childhood_BMI | -0.011 | 0.0286 | -0.067 | 0.045 | 6.93e-01 | 0.931892066 |
| node_pheno1 | childhood_BMI | -0.022 | 0.0318 | -0.084 | 0.040 | 4.88e-01 | 0.828836593 |
| node_pheno10 | childhood_BMI | 0.034 | 0.0297 | -0.025 | 0.092 | 2.57e-01 | 0.731655758 |
| node_pheno11 | childhood_BMI | -0.020 | 0.0306 | -0.080 | 0.040 | 5.21e-01 | 0.839421998 |
| node_pheno12 | childhood_BMI | 0.050 | 0.0332 | -0.015 | 0.115 | 1.28e-01 | 0.530912634 |
| node_pheno13 | childhood_BMI | -0.029 | 0.0270 | -0.082 | 0.024 | 2.84e-01 | 0.746547599 |
| node_pheno14 | childhood_BMI | 0.023 | 0.0305 | -0.036 | 0.083 | 4.45e-01 | 0.813746745 |
| node_pheno15 | childhood_BMI | 0.040 | 0.0306 | -0.020 | 0.100 | 1.96e-01 | 0.655313302 |
| node_pheno16 | childhood_BMI | 0.049 | 0.0293 | -0.009 | 0.106 | 9.69e-02 | 0.486924804 |
| node_pheno17 | childhood_BMI | 0.018 | 0.0330 | -0.046 | 0.083 | 5.77e-01 | 0.860435372 |
| node_pheno18 | childhood_BMI | 0.000 | 0.0314 | -0.062 | 0.061 | 9.91e-01 | 0.996646331 |
| node_pheno19 | childhood_BMI | 0.020 | 0.0321 | -0.043 | 0.082 | 5.42e-01 | 0.853067207 |
| node_pheno2 | childhood_BMI | 0.007 | 0.0321 | -0.056 | 0.070 | 8.19e-01 | 0.958241885 |
| node_pheno20 | childhood_BMI | 0.033 | 0.0374 | -0.040 | 0.106 | 3.79e-01 | 0.802264449 |
| node_pheno21 | childhood_BMI | 0.026 | 0.0299 | -0.033 | 0.084 | 3.85e-01 | 0.802264449 |
| node_pheno22 | childhood_BMI | 0.009 | 0.0299 | -0.050 | 0.067 | 7.68e-01 | 0.958241885 |
| node_pheno23 | childhood_BMI | 0.009 | 0.0326 | -0.055 | 0.073 | 7.88e-01 | 0.958241885 |
| node_pheno24 | childhood_BMI | 0.048 | 0.0308 | -0.012 | 0.109 | 1.16e-01 | 0.52711383 |
| node_pheno25 | childhood_BMI | 0.011 | 0.0306 | -0.049 | 0.071 | 7.09e-01 | 0.947533776 |
| node_pheno26 | childhood_BMI | 0.018 | 0.0256 | -0.032 | 0.068 | 4.90e-01 | 0.828836593 |
| node_pheno27 | childhood_BMI | -0.023 | 0.0318 | -0.086 | 0.039 | 4.66e-01 | 0.816273304 |
| node_pheno28 | childhood_BMI | 0.015 | 0.0293 | -0.043 | 0.072 | 6.11e-01 | 0.880763225 |
| node_pheno29 | childhood_BMI | 0.076 | 0.0289 | 0.019 | 0.133 | 8.50e-03 | 0.162442985 |
| node_pheno3 | childhood_BMI | -0.045 | 0.0299 | -0.104 | 0.014 | 1.31e-01 | 0.533949597 |
| node_pheno30 | childhood_BMI | 0.033 | 0.0310 | -0.028 | 0.093 | 2.94e-01 | 0.746547599 |
| node_pheno31 | childhood_BMI | 0.022 | 0.0282 | -0.033 | 0.077 | 4.37e-01 | 0.813746745 |
| node_pheno32 | childhood_BMI | 0.017 | 0.0302 | -0.042 | 0.076 | 5.80e-01 | 0.862833896 |
| node_pheno33 | childhood_BMI | 0.038 | 0.0262 | -0.013 | 0.090 | 1.44e-01 | 0.564634214 |
| node_pheno34 | childhood_BMI | 0.034 | 0.0255 | -0.016 | 0.084 | 1.80e-01 | 0.623850644 |
| node_pheno35 | childhood_BMI | 0.056 | 0.0322 | -0.007 | 0.119 | 8.32e-02 | 0.486924804 |
| node_pheno36 | childhood_BMI | 0.040 | 0.0319 | -0.022 | 0.103 | 2.09e-01 | 0.665094404 |
| node_pheno37 | childhood_BMI | 0.016 | 0.0300 | -0.043 | 0.075 | 5.91e-01 | 0.871612318 |
| node_pheno38 | childhood_BMI | 0.037 | 0.0286 | -0.019 | 0.093 | 1.98e-01 | 0.655313302 |
| node_pheno39 | childhood_BMI | 0.012 | 0.0287 | -0.045 | 0.068 | 6.85e-01 | 0.924814508 |
| node_pheno4 | childhood_BMI | 0.010 | 0.0317 | -0.052 | 0.072 | 7.61e-01 | 0.958241885 |
| node_pheno40 | childhood_BMI | 0.042 | 0.0355 | -0.028 | 0.111 | 2.40e-01 | 0.706744706 |
| node_pheno41 | childhood_BMI | 0.046 | 0.0315 | -0.016 | 0.107 | 1.48e-01 | 0.564634214 |
| node_pheno42 | childhood_BMI | -0.009 | 0.0307 | -0.069 | 0.051 | 7.69e-01 | 0.958241885 |
| node_pheno43 | childhood_BMI | 0.036 | 0.0336 | -0.030 | 0.101 | 2.90e-01 | 0.746547599 |
| node_pheno44 | childhood_BMI | 0.033 | 0.0246 | -0.016 | 0.081 | 1.87e-01 | 0.643261304 |
| node_pheno45 | childhood_BMI | 0.005 | 0.0323 | -0.058 | 0.069 | 8.71e-01 | 0.958241885 |
| node_pheno46 | childhood_BMI | 0.055 | 0.0327 | -0.010 | 0.119 | 9.51e-02 | 0.486924804 |
| node_pheno47 | childhood_BMI | 0.033 | 0.0317 | -0.029 | 0.095 | 2.95e-01 | 0.746547599 |
| node_pheno48 | childhood_BMI | 0.037 | 0.0334 | -0.028 | 0.102 | 2.68e-01 | 0.743509995 |
| node_pheno49 | childhood_BMI | -0.003 | 0.0269 | -0.055 | 0.050 | 9.22e-01 | 0.970919907 |
| node_pheno5 | childhood_BMI | 0.034 | 0.0337 | -0.032 | 0.100 | 3.17e-01 | 0.754858317 |
| node_pheno50 | childhood_BMI | 0.045 | 0.0314 | -0.017 | 0.106 | 1.53e-01 | 0.567943724 |
| node_pheno51 | childhood_BMI | 0.059 | 0.0310 | -0.002 | 0.120 | 5.74e-02 | 0.414007535 |
| node_pheno52 | childhood_BMI | 0.029 | 0.0281 | -0.026 | 0.084 | 2.97e-01 | 0.746547599 |
| node_pheno53 | childhood_BMI | 0.049 | 0.0320 | -0.013 | 0.112 | 1.22e-01 | 0.530912634 |
| node_pheno54 | childhood_BMI | 0.009 | 0.0301 | -0.050 | 0.068 | 7.76e-01 | 0.958241885 |
| node_pheno55 | childhood_BMI | 0.002 | 0.0317 | -0.060 | 0.064 | 9.42e-01 | 0.975128003 |
| node_pheno56 | childhood_BMI | 0.005 | 0.0355 | -0.064 | 0.075 | 8.83e-01 | 0.960570209 |
| node_pheno57 | childhood_BMI | 0.055 | 0.0322 | -0.008 | 0.118 | 8.70e-02 | 0.486924804 |
| node_pheno58 | childhood_BMI | 0.031 | 0.0302 | -0.028 | 0.090 | 3.06e-01 | 0.75404701 |
| node_pheno59 | childhood_BMI | 0.021 | 0.0261 | -0.030 | 0.073 | 4.12e-01 | 0.813746745 |
| node_pheno60 | childhood_BMI | -0.006 | 0.0331 | -0.071 | 0.059 | 8.51e-01 | 0.958241885 |
| node_pheno61 | childhood_BMI | 0.009 | 0.0314 | -0.052 | 0.071 | 7.69e-01 | 0.958241885 |
| node_pheno62 | childhood_BMI | 0.062 | 0.0396 | -0.015 | 0.140 | 1.15e-01 | 0.52711383 |
| node_pheno63 | childhood_BMI | 0.051 | 0.0296 | -0.007 | 0.109 | 8.71e-02 | 0.486924804 |
| node_pheno64 | childhood_BMI | 0.044 | 0.0244 | -0.003 | 0.092 | 6.92e-02 | 0.471779995 |
| node_pheno65 | childhood_BMI | 0.017 | 0.0301 | -0.042 | 0.076 | 5.66e-01 | 0.853067207 |
| node_pheno66 | childhood_BMI | 0.063 | 0.0321 | 0.000 | 0.125 | 5.11e-02 | 0.390400558 |
| node_pheno67 | childhood_BMI | 0.049 | 0.0358 | -0.021 | 0.119 | 1.72e-01 | 0.606572815 |
| node_pheno68 | childhood_BMI | 0.024 | 0.0297 | -0.034 | 0.082 | 4.16e-01 | 0.813746745 |
| node_pheno69 | childhood_BMI | 0.083 | 0.0366 | 0.011 | 0.154 | 2.42e-02 | 0.274978068 |
| node_pheno7 | childhood_BMI | 0.007 | 0.0317 | -0.055 | 0.070 | 8.13e-01 | 0.958241885 |
| node_pheno70 | childhood_BMI | 0.028 | 0.0315 | -0.034 | 0.089 | 3.79e-01 | 0.802264449 |
| node_pheno71 | childhood_BMI | 0.003 | 0.0289 | -0.054 | 0.059 | 9.30e-01 | 0.970919907 |
| node_pheno72 | childhood_BMI | 0.057 | 0.0262 | 0.005 | 0.108 | 3.01e-02 | 0.280836432 |
| node_pheno73 | childhood_BMI | 0.035 | 0.0258 | -0.015 | 0.086 | 1.70e-01 | 0.605477177 |
| node_pheno74 | childhood_BMI | 0.029 | 0.0293 | -0.028 | 0.087 | 3.20e-01 | 0.754858317 |
| node_pheno75 | childhood_BMI | 0.027 | 0.0306 | -0.033 | 0.087 | 3.81e-01 | 0.802264449 |
| node_pheno76 | childhood_BMI | 0.031 | 0.0283 | -0.025 | 0.086 | 2.77e-01 | 0.74494082 |
| node_pheno8 | childhood_BMI | 0.021 | 0.0351 | -0.048 | 0.090 | 5.55e-01 | 0.853067207 |
| node_pheno9 | childhood_BMI | 0.017 | 0.0281 | -0.038 | 0.072 | 5.45e-01 | 0.853067207 |
| edge_pheno1020 | adulthood_BMI | 0.077 | 0.0241 | 0.030 | 0.125 | 1.30e-03 | 0.076392839 |
| edge_pheno288 | adulthood_BMI | -0.074 | 0.0241 | -0.121 | -0.026 | 2.27e-03 | 0.076392839 |
| node_pheno29 | adulthood_BMI | 0.074 | 0.0242 | 0.027 | 0.122 | 2.22e-03 | 0.076392839 |
| node_pheno36 | adulthood_BMI | 0.078 | 0.0242 | 0.030 | 0.125 | 1.34e-03 | 0.076392839 |
| node_pheno50 | adulthood_BMI | 0.082 | 0.0244 | 0.034 | 0.130 | 7.88e-04 | 0.076392839 |
| node_pheno52 | adulthood_BMI | 0.078 | 0.0249 | 0.029 | 0.127 | 1.74e-03 | 0.076392839 |
| node_pheno58 | adulthood_BMI | 0.079 | 0.0257 | 0.029 | 0.130 | 2.10e-03 | 0.076392839 |
| node_pheno64 | adulthood_BMI | 0.082 | 0.0231 | 0.037 | 0.128 | 3.62e-04 | 0.076392839 |
| node_pheno73 | adulthood_BMI | 0.072 | 0.0231 | 0.026 | 0.117 | 1.91e-03 | 0.076392839 |
| node_pheno76 | adulthood_BMI | 0.069 | 0.0221 | 0.026 | 0.112 | 1.80e-03 | 0.076392839 |
| node_pheno66 | adulthood_BMI | 0.074 | 0.0248 | 0.025 | 0.122 | 2.86e-03 | 0.083988848 |
| node_pheno23 | adulthood_BMI | 0.073 | 0.0255 | 0.024 | 0.123 | 3.94e-03 | 0.10021925 |
| node_pheno61 | adulthood_BMI | 0.069 | 0.0240 | 0.022 | 0.117 | 3.86e-03 | 0.10021925 |
| node_pheno72 | adulthood_BMI | 0.061 | 0.0218 | 0.018 | 0.103 | 5.23e-03 | 0.117409809 |
| node_pheno62 | adulthood_BMI | 0.068 | 0.0248 | 0.019 | 0.116 | 6.18e-03 | 0.131133062 |
| node_pheno67 | adulthood_BMI | 0.065 | 0.0247 | 0.017 | 0.114 | 8.06e-03 | 0.162126599 |
| node_pheno25 | adulthood_BMI | 0.064 | 0.0246 | 0.015 | 0.112 | 9.67e-03 | 0.175856171 |
| node_pheno63 | adulthood_BMI | 0.063 | 0.0247 | 0.014 | 0.111 | 1.11e-02 | 0.183722413 |
| node_pheno28 | adulthood_BMI | 0.060 | 0.0243 | 0.013 | 0.108 | 1.29e-02 | 0.189626828 |
| node_pheno32 | adulthood_BMI | 0.060 | 0.0240 | 0.013 | 0.107 | 1.22e-02 | 0.189626828 |
| node_pheno68 | adulthood_BMI | 0.057 | 0.0236 | 0.011 | 0.103 | 1.55e-02 | 0.219428119 |
| node_pheno51 | adulthood_BMI | 0.062 | 0.0259 | 0.011 | 0.113 | 1.65e-02 | 0.225182517 |
| edge_pheno1273 | adulthood_BMI | 0.057 | 0.0256 | 0.007 | 0.107 | 2.57e-02 | 0.274978068 |
| edge_pheno695 | adulthood_BMI | 0.056 | 0.0249 | 0.007 | 0.105 | 2.42e-02 | 0.274978068 |
| node_pheno33 | adulthood_BMI | 0.058 | 0.0258 | 0.007 | 0.108 | 2.59e-02 | 0.274978068 |
| node_pheno48 | adulthood_BMI | 0.058 | 0.0258 | 0.008 | 0.109 | 2.40e-02 | 0.274978068 |
| node_pheno59 | adulthood_BMI | 0.050 | 0.0223 | 0.006 | 0.094 | 2.48e-02 | 0.274978068 |
| node_pheno34 | adulthood_BMI | 0.055 | 0.0251 | 0.006 | 0.105 | 2.69e-02 | 0.277517191 |
| node_pheno56 | adulthood_BMI | 0.057 | 0.0257 | 0.006 | 0.107 | 2.78e-02 | 0.279783957 |
| node_pheno24 | adulthood_BMI | 0.054 | 0.0250 | 0.005 | 0.103 | 2.97e-02 | 0.280836432 |
| node_pheno26 | adulthood_BMI | 0.050 | 0.0228 | 0.005 | 0.095 | 2.91e-02 | 0.280836432 |
| node_pheno69 | adulthood_BMI | 0.052 | 0.0246 | 0.004 | 0.100 | 3.53e-02 | 0.31372883 |
| node_pheno75 | adulthood_BMI | 0.046 | 0.0218 | 0.003 | 0.089 | 3.52e-02 | 0.31372883 |
| node_pheno35 | adulthood_BMI | 0.050 | 0.0242 | 0.002 | 0.097 | 3.91e-02 | 0.339604399 |
| edge_pheno1013 | adulthood_BMI | -0.051 | 0.0247 | -0.099 | -0.002 | 4.01e-02 | 0.340431927 |
| node_pheno14 | adulthood_BMI | 0.052 | 0.0261 | 0.001 | 0.103 | 4.65e-02 | 0.378292571 |
| node_pheno65 | adulthood_BMI | 0.050 | 0.0257 | 0.000 | 0.101 | 5.10e-02 | 0.390400558 |
| node_pheno31 | adulthood_BMI | 0.048 | 0.0255 | -0.001 | 0.098 | 5.73e-02 | 0.414007535 |
| edge_pheno1311 | adulthood_BMI | 0.054 | 0.0294 | -0.004 | 0.111 | 6.84e-02 | 0.471779995 |
| edge_pheno1225 | adulthood_BMI | 0.044 | 0.0258 | -0.006 | 0.095 | 8.64e-02 | 0.486924804 |
| edge_pheno286 | adulthood_BMI | -0.043 | 0.0244 | -0.091 | 0.005 | 7.75e-02 | 0.486924804 |
| edge_pheno789 | adulthood_BMI | -0.041 | 0.0241 | -0.088 | 0.006 | 9.00e-02 | 0.486924804 |
| edge_pheno810 | adulthood_BMI | -0.042 | 0.0243 | -0.089 | 0.006 | 8.58e-02 | 0.486924804 |
| node_pheno12 | adulthood_BMI | 0.044 | 0.0255 | -0.006 | 0.094 | 8.25e-02 | 0.486924804 |
| node_pheno38 | adulthood_BMI | 0.044 | 0.0251 | -0.005 | 0.093 | 8.07e-02 | 0.486924804 |
| node_pheno39 | adulthood_BMI | 0.042 | 0.0249 | -0.007 | 0.091 | 9.38e-02 | 0.486924804 |
| node_pheno57 | adulthood_BMI | 0.042 | 0.0239 | -0.005 | 0.088 | 8.16e-02 | 0.486924804 |
| node_pheno71 | adulthood_BMI | 0.042 | 0.0237 | -0.004 | 0.089 | 7.59e-02 | 0.486924804 |
| edge_pheno801 | adulthood_BMI | 0.042 | 0.0259 | -0.008 | 0.093 | 1.01e-01 | 0.494334426 |
| edge_pheno777 | adulthood_BMI | -0.043 | 0.0268 | -0.095 | 0.010 | 1.10e-01 | 0.519359524 |
| node_pheno41 | adulthood_BMI | 0.042 | 0.0263 | -0.010 | 0.093 | 1.12e-01 | 0.521810883 |
| edge_pheno1141 | adulthood_BMI | -0.041 | 0.0269 | -0.094 | 0.011 | 1.25e-01 | 0.530912634 |
| edge_pheno1319 | adulthood_BMI | 0.041 | 0.0264 | -0.011 | 0.092 | 1.24e-01 | 0.530912634 |
| edge_pheno599 | adulthood_BMI | -0.039 | 0.0257 | -0.090 | 0.011 | 1.27e-01 | 0.530912634 |
| edge_pheno815 | adulthood_BMI | 0.041 | 0.0266 | -0.012 | 0.093 | 1.28e-01 | 0.530912634 |
| edge_pheno956 | adulthood_BMI | -0.042 | 0.0270 | -0.095 | 0.011 | 1.20e-01 | 0.530912634 |
| node_pheno60 | adulthood_BMI | 0.038 | 0.0246 | -0.011 | 0.086 | 1.26e-01 | 0.530912634 |
| node_pheno7 | adulthood_BMI | 0.040 | 0.0270 | -0.013 | 0.093 | 1.35e-01 | 0.53810511 |
| edge_pheno1041 | adulthood_BMI | 0.037 | 0.0252 | -0.013 | 0.086 | 1.47e-01 | 0.564634214 |
| edge_pheno135 | adulthood_BMI | -0.035 | 0.0244 | -0.083 | 0.013 | 1.52e-01 | 0.567943724 |
| edge_pheno698 | adulthood_BMI | -0.035 | 0.0248 | -0.084 | 0.013 | 1.52e-01 | 0.567943724 |
| node_pheno46 | adulthood_BMI | 0.037 | 0.0266 | -0.015 | 0.089 | 1.61e-01 | 0.587251108 |
| node_pheno43 | adulthood_BMI | 0.036 | 0.0260 | -0.015 | 0.087 | 1.68e-01 | 0.605477177 |
| edge_pheno1359 | adulthood_BMI | -0.036 | 0.0262 | -0.087 | 0.016 | 1.73e-01 | 0.606572815 |
| node_pheno54 | adulthood_BMI | 0.032 | 0.0248 | -0.016 | 0.081 | 1.92e-01 | 0.647891165 |
| edge_pheno816 | adulthood_BMI | -0.031 | 0.0243 | -0.079 | 0.017 | 2.01e-01 | 0.655313302 |
| node_pheno53 | adulthood_BMI | 0.034 | 0.0263 | -0.018 | 0.085 | 2.02e-01 | 0.655313302 |
| node_pheno70 | adulthood_BMI | 0.030 | 0.0238 | -0.016 | 0.077 | 1.99e-01 | 0.655313302 |
| node_pheno37 | adulthood_BMI | 0.032 | 0.0256 | -0.018 | 0.082 | 2.07e-01 | 0.66475667 |
| edge_pheno1696 | adulthood_BMI | 0.032 | 0.0255 | -0.018 | 0.082 | 2.15e-01 | 0.666251865 |
| edge_pheno932 | adulthood_BMI | -0.033 | 0.0265 | -0.085 | 0.019 | 2.14e-01 | 0.666251865 |
| node_pheno74 | adulthood_BMI | 0.031 | 0.0251 | -0.018 | 0.080 | 2.16e-01 | 0.666251865 |
| node_pheno40 | adulthood_BMI | 0.030 | 0.0244 | -0.018 | 0.078 | 2.24e-01 | 0.685966292 |
| edge_pheno101 | adulthood_BMI | -0.030 | 0.0251 | -0.079 | 0.019 | 2.35e-01 | 0.706744706 |
| node_pheno16 | adulthood_BMI | 0.029 | 0.0249 | -0.019 | 0.078 | 2.38e-01 | 0.706744706 |
| node_pheno55 | adulthood_BMI | 0.030 | 0.0253 | -0.020 | 0.080 | 2.37e-01 | 0.706744706 |
| edge_pheno812 | adulthood_BMI | -0.031 | 0.0268 | -0.083 | 0.022 | 2.49e-01 | 0.715628738 |
| edge_pheno103 | adulthood_BMI | -0.027 | 0.0249 | -0.076 | 0.022 | 2.73e-01 | 0.743509995 |
| edge_pheno1142 | adulthood_BMI | -0.029 | 0.0260 | -0.080 | 0.022 | 2.70e-01 | 0.743509995 |
| node_pheno10 | adulthood_BMI | 0.028 | 0.0259 | -0.022 | 0.079 | 2.74e-01 | 0.743509995 |
| node_pheno21 | adulthood_BMI | 0.028 | 0.0258 | -0.022 | 0.079 | 2.72e-01 | 0.743509995 |
| node_pheno45 | adulthood_BMI | 0.026 | 0.0237 | -0.020 | 0.073 | 2.66e-01 | 0.743509995 |
| edge_pheno1221 | adulthood_BMI | 0.028 | 0.0272 | -0.025 | 0.081 | 3.00e-01 | 0.746547599 |
| edge_pheno1382 | adulthood_BMI | -0.030 | 0.0275 | -0.083 | 0.024 | 2.80e-01 | 0.746547599 |
| edge_pheno683 | adulthood_BMI | -0.027 | 0.0251 | -0.076 | 0.022 | 2.83e-01 | 0.746547599 |
| edge_pheno867 | adulthood_BMI | -0.027 | 0.0260 | -0.078 | 0.024 | 2.95e-01 | 0.746547599 |
| edge_pheno87 | adulthood_BMI | 0.026 | 0.0254 | -0.024 | 0.076 | 3.01e-01 | 0.746547599 |
| node_pheno42 | adulthood_BMI | 0.026 | 0.0253 | -0.023 | 0.076 | 3.00e-01 | 0.746547599 |
| edge_pheno1175 | adulthood_BMI | 0.024 | 0.0244 | -0.024 | 0.072 | 3.20e-01 | 0.754858317 |
| edge_pheno606 | adulthood_BMI | 0.026 | 0.0253 | -0.024 | 0.075 | 3.11e-01 | 0.754858317 |
| node_pheno13 | adulthood_BMI | -0.027 | 0.0268 | -0.079 | 0.026 | 3.17e-01 | 0.754858317 |
| edge_pheno1171 | adulthood_BMI | 0.026 | 0.0266 | -0.026 | 0.078 | 3.33e-01 | 0.763375435 |
| edge_pheno461 | adulthood_BMI | -0.023 | 0.0244 | -0.071 | 0.024 | 3.36e-01 | 0.764373318 |
| edge_pheno55 | adulthood_BMI | 0.023 | 0.0248 | -0.026 | 0.072 | 3.53e-01 | 0.797546912 |
| edge_pheno1189 | adulthood_BMI | 0.024 | 0.0264 | -0.028 | 0.076 | 3.64e-01 | 0.800194823 |
| edge_pheno1257 | adulthood_BMI | 0.023 | 0.0248 | -0.026 | 0.071 | 3.64e-01 | 0.800194823 |
| edge_pheno1269 | adulthood_BMI | -0.024 | 0.0262 | -0.075 | 0.027 | 3.60e-01 | 0.800194823 |
| edge_pheno558 | adulthood_BMI | -0.023 | 0.0247 | -0.071 | 0.026 | 3.57e-01 | 0.800194823 |
| edge_pheno1317 | adulthood_BMI | -0.028 | 0.0315 | -0.090 | 0.034 | 3.76e-01 | 0.802264449 |
| edge_pheno447 | adulthood_BMI | -0.070 | 0.0793 | -0.226 | 0.085 | 3.76e-01 | 0.802264449 |
| node_pheno17 | adulthood_BMI | 0.023 | 0.0256 | -0.027 | 0.073 | 3.70e-01 | 0.802264449 |
| node_pheno18 | adulthood_BMI | 0.022 | 0.0250 | -0.027 | 0.071 | 3.86e-01 | 0.802264449 |
| edge_pheno965 | adulthood_BMI | 0.021 | 0.0243 | -0.027 | 0.069 | 3.89e-01 | 0.803521931 |
| edge_pheno1022 | adulthood_BMI | 0.019 | 0.0243 | -0.029 | 0.066 | 4.40e-01 | 0.813746745 |
| edge_pheno1126 | adulthood_BMI | -0.020 | 0.0251 | -0.070 | 0.029 | 4.16e-01 | 0.813746745 |
| edge_pheno1134 | adulthood_BMI | 0.022 | 0.0271 | -0.031 | 0.075 | 4.12e-01 | 0.813746745 |
| edge_pheno1698 | adulthood_BMI | 0.025 | 0.0299 | -0.034 | 0.084 | 4.05e-01 | 0.813746745 |
| edge_pheno636 | adulthood_BMI | 0.022 | 0.0263 | -0.030 | 0.073 | 4.09e-01 | 0.813746745 |
| edge_pheno869 | adulthood_BMI | 0.022 | 0.0280 | -0.033 | 0.077 | 4.26e-01 | 0.813746745 |
| node_pheno22 | adulthood_BMI | 0.020 | 0.0246 | -0.028 | 0.069 | 4.07e-01 | 0.813746745 |
| node_pheno30 | adulthood_BMI | 0.018 | 0.0239 | -0.028 | 0.065 | 4.40e-01 | 0.813746745 |
| node_pheno4 | adulthood_BMI | 0.019 | 0.0250 | -0.030 | 0.068 | 4.44e-01 | 0.813746745 |
| node_pheno44 | adulthood_BMI | 0.020 | 0.0235 | -0.026 | 0.066 | 4.02e-01 | 0.813746745 |
| edge_pheno389 | adulthood_BMI | -0.020 | 0.0260 | -0.071 | 0.031 | 4.48e-01 | 0.815139891 |
| edge_pheno102 | adulthood_BMI | -0.019 | 0.0258 | -0.070 | 0.031 | 4.59e-01 | 0.816249798 |
| edge_pheno1301 | adulthood_BMI | 0.020 | 0.0265 | -0.032 | 0.072 | 4.59e-01 | 0.816249798 |
| edge_pheno132 | adulthood_BMI | 0.018 | 0.0250 | -0.031 | 0.067 | 4.61e-01 | 0.816249798 |
| node_pheno15 | adulthood_BMI | 0.020 | 0.0267 | -0.032 | 0.072 | 4.54e-01 | 0.816249798 |
| edge_pheno1699 | adulthood_BMI | -0.019 | 0.0260 | -0.070 | 0.032 | 4.64e-01 | 0.816273304 |
| edge_pheno942 | adulthood_BMI | -0.020 | 0.0273 | -0.073 | 0.034 | 4.72e-01 | 0.819447892 |
| edge_pheno1296 | adulthood_BMI | -0.017 | 0.0256 | -0.067 | 0.033 | 5.06e-01 | 0.828836593 |
| edge_pheno1302 | adulthood_BMI | -0.018 | 0.0266 | -0.070 | 0.034 | 4.91e-01 | 0.828836593 |
| edge_pheno574 | adulthood_BMI | -0.017 | 0.0245 | -0.065 | 0.032 | 5.00e-01 | 0.828836593 |
| edge_pheno601 | adulthood_BMI | 0.018 | 0.0257 | -0.032 | 0.068 | 4.86e-01 | 0.828836593 |
| edge_pheno824 | adulthood_BMI | -0.018 | 0.0260 | -0.069 | 0.033 | 4.89e-01 | 0.828836593 |
| node_pheno2 | adulthood_BMI | 0.017 | 0.0255 | -0.033 | 0.067 | 5.01e-01 | 0.828836593 |
| node_pheno9 | adulthood_BMI | 0.017 | 0.0264 | -0.034 | 0.069 | 5.09e-01 | 0.830623671 |
| edge_pheno1059 | adulthood_BMI | -0.017 | 0.0259 | -0.068 | 0.034 | 5.15e-01 | 0.834490969 |
| edge_pheno449 | adulthood_BMI | -0.055 | 0.0879 | -0.228 | 0.117 | 5.29e-01 | 0.843109619 |
| edge_pheno1183 | adulthood_BMI | 0.015 | 0.0262 | -0.036 | 0.067 | 5.55e-01 | 0.853067207 |
| edge_pheno1293 | adulthood_BMI | 0.015 | 0.0264 | -0.036 | 0.067 | 5.63e-01 | 0.853067207 |
| edge_pheno249 | adulthood_BMI | 0.015 | 0.0254 | -0.035 | 0.065 | 5.61e-01 | 0.853067207 |
| edge_pheno716 | adulthood_BMI | -0.014 | 0.0250 | -0.063 | 0.035 | 5.69e-01 | 0.853067207 |
| node_pheno20 | adulthood_BMI | 0.015 | 0.0249 | -0.034 | 0.063 | 5.56e-01 | 0.853067207 |
| node_pheno8 | adulthood_BMI | 0.014 | 0.0245 | -0.034 | 0.062 | 5.63e-01 | 0.853067207 |
| edge_pheno624 | adulthood_BMI | 0.013 | 0.0247 | -0.035 | 0.062 | 5.90e-01 | 0.871612318 |
| edge_pheno681 | adulthood_BMI | 0.014 | 0.0255 | -0.036 | 0.064 | 5.93e-01 | 0.871612318 |
| edge_pheno1250 | adulthood_BMI | 0.014 | 0.0261 | -0.037 | 0.065 | 5.98e-01 | 0.875393052 |
| edge_pheno1167 | adulthood_BMI | -0.014 | 0.0270 | -0.067 | 0.039 | 6.09e-01 | 0.880763225 |
| edge_pheno491 | adulthood_BMI | -0.013 | 0.0264 | -0.065 | 0.038 | 6.10e-01 | 0.880763225 |
| edge_pheno1256 | adulthood_BMI | 0.013 | 0.0257 | -0.037 | 0.063 | 6.15e-01 | 0.88297052 |
| edge_pheno1161 | adulthood_BMI | -0.013 | 0.0270 | -0.066 | 0.040 | 6.28e-01 | 0.898899735 |
| edge_pheno147 | adulthood_BMI | -0.012 | 0.0254 | -0.062 | 0.038 | 6.39e-01 | 0.900920809 |
| edge_pheno903 | adulthood_BMI | 0.011 | 0.0257 | -0.039 | 0.062 | 6.57e-01 | 0.922053355 |
| edge_pheno1276 | adulthood_BMI | -0.011 | 0.0249 | -0.060 | 0.038 | 6.66e-01 | 0.92292935 |
| edge_pheno1300 | adulthood_BMI | 0.011 | 0.0258 | -0.040 | 0.061 | 6.71e-01 | 0.92292935 |
| edge_pheno1328 | adulthood_BMI | 0.011 | 0.0264 | -0.041 | 0.063 | 6.74e-01 | 0.92292935 |
| edge_pheno262 | adulthood_BMI | -0.010 | 0.0249 | -0.059 | 0.038 | 6.74e-01 | 0.92292935 |
| edge_pheno794 | adulthood_BMI | -0.010 | 0.0236 | -0.057 | 0.036 | 6.63e-01 | 0.92292935 |
| node_pheno5 | adulthood_BMI | 0.011 | 0.0274 | -0.043 | 0.065 | 6.85e-01 | 0.924814508 |
| edge_pheno639 | adulthood_BMI | -0.009 | 0.0238 | -0.055 | 0.038 | 7.18e-01 | 0.955786893 |
| edge_pheno1184 | adulthood_BMI | 0.009 | 0.0260 | -0.042 | 0.060 | 7.27e-01 | 0.956764954 |
| edge_pheno590 | adulthood_BMI | -0.009 | 0.0268 | -0.062 | 0.043 | 7.31e-01 | 0.956764954 |
| edge_pheno1122 | adulthood_BMI | -0.006 | 0.0293 | -0.063 | 0.052 | 8.40e-01 | 0.958241885 |
| edge_pheno1137 | adulthood_BMI | -0.008 | 0.0276 | -0.062 | 0.046 | 7.63e-01 | 0.958241885 |
| edge_pheno1205 | adulthood_BMI | 0.004 | 0.0235 | -0.042 | 0.050 | 8.64e-01 | 0.958241885 |
| edge_pheno1270 | adulthood_BMI | 0.006 | 0.0265 | -0.046 | 0.058 | 8.10e-01 | 0.958241885 |
| edge_pheno1309 | adulthood_BMI | 0.006 | 0.0271 | -0.047 | 0.059 | 8.27e-01 | 0.958241885 |
| edge_pheno1322 | adulthood_BMI | -0.007 | 0.0259 | -0.058 | 0.044 | 7.93e-01 | 0.958241885 |
| edge_pheno151 | adulthood_BMI | 0.007 | 0.0246 | -0.041 | 0.055 | 7.70e-01 | 0.958241885 |
| edge_pheno1697 | adulthood_BMI | -0.005 | 0.0270 | -0.058 | 0.048 | 8.44e-01 | 0.958241885 |
| edge_pheno537 | adulthood_BMI | 0.008 | 0.0265 | -0.044 | 0.060 | 7.60e-01 | 0.958241885 |
| edge_pheno58 | adulthood_BMI | 0.004 | 0.0246 | -0.044 | 0.053 | 8.57e-01 | 0.958241885 |
| edge_pheno593 | adulthood_BMI | -0.004 | 0.0266 | -0.056 | 0.048 | 8.75e-01 | 0.958241885 |
| edge_pheno609 | adulthood_BMI | 0.008 | 0.0261 | -0.043 | 0.059 | 7.52e-01 | 0.958241885 |
| edge_pheno65 | adulthood_BMI | -0.005 | 0.0246 | -0.053 | 0.044 | 8.54e-01 | 0.958241885 |
| edge_pheno674 | adulthood_BMI | -0.004 | 0.0253 | -0.054 | 0.045 | 8.64e-01 | 0.958241885 |
| edge_pheno705 | adulthood_BMI | 0.007 | 0.0252 | -0.042 | 0.057 | 7.70e-01 | 0.958241885 |
| edge_pheno899 | adulthood_BMI | -0.004 | 0.0252 | -0.054 | 0.045 | 8.69e-01 | 0.958241885 |
| edge_pheno908 | adulthood_BMI | -0.006 | 0.0254 | -0.056 | 0.044 | 8.08e-01 | 0.958241885 |
| node_pheno1 | adulthood_BMI | 0.007 | 0.0266 | -0.045 | 0.059 | 7.82e-01 | 0.958241885 |
| node_pheno11 | adulthood_BMI | -0.004 | 0.0257 | -0.054 | 0.046 | 8.75e-01 | 0.958241885 |
| node_pheno19 | adulthood_BMI | 0.008 | 0.0250 | -0.041 | 0.057 | 7.36e-01 | 0.958241885 |
| node_pheno47 | adulthood_BMI | 0.005 | 0.0248 | -0.044 | 0.053 | 8.45e-01 | 0.958241885 |
| node_pheno49 | adulthood_BMI | -0.007 | 0.0272 | -0.061 | 0.046 | 7.93e-01 | 0.958241885 |
| edge_pheno303 | adulthood_BMI | 0.004 | 0.0252 | -0.046 | 0.053 | 8.82e-01 | 0.960570209 |
| edge_pheno253 | adulthood_BMI | -0.003 | 0.0256 | -0.054 | 0.047 | 8.98e-01 | 0.969768565 |
| edge_pheno767 | adulthood_BMI | -0.003 | 0.0248 | -0.052 | 0.045 | 8.99e-01 | 0.969768565 |
| edge_pheno1211 | adulthood_BMI | -0.003 | 0.0253 | -0.052 | 0.047 | 9.17e-01 | 0.970919907 |
| edge_pheno1325 | adulthood_BMI | -0.003 | 0.0253 | -0.053 | 0.047 | 9.04e-01 | 0.970919907 |
| edge_pheno146 | adulthood_BMI | -0.002 | 0.0242 | -0.050 | 0.045 | 9.24e-01 | 0.970919907 |
| edge_pheno66 | adulthood_BMI | 0.003 | 0.0245 | -0.046 | 0.051 | 9.18e-01 | 0.970919907 |
| edge_pheno882 | adulthood_BMI | 0.002 | 0.0242 | -0.045 | 0.050 | 9.21e-01 | 0.970919907 |
| node_pheno27 | adulthood_BMI | -0.003 | 0.0258 | -0.053 | 0.048 | 9.20e-01 | 0.970919907 |
| node_pheno3 | adulthood_BMI | 0.003 | 0.0261 | -0.048 | 0.054 | 9.08e-01 | 0.970919907 |
| edge_pheno621 | adulthood_BMI | -0.002 | 0.0254 | -0.052 | 0.048 | 9.36e-01 | 0.973845911 |
| edge_pheno460 | adulthood_BMI | 0.002 | 0.0252 | -0.048 | 0.051 | 9.43e-01 | 0.975128003 |
| edge_pheno1701 | adulthood_BMI | 0.002 | 0.0301 | -0.057 | 0.061 | 9.59e-01 | 0.976467254 |
| edge_pheno918 | adulthood_BMI | 0.002 | 0.0238 | -0.045 | 0.048 | 9.49e-01 | 0.976467254 |
| edge_pheno597 | adulthood_BMI | -0.001 | 0.0269 | -0.053 | 0.052 | 9.82e-01 | 0.992440173 |
| edge_pheno405 | adulthood_BMI | 0.000 | 0.0263 | -0.052 | 0.051 | 9.92e-01 | 0.996646331 |

**Table S4. Associations of genetically predicted life-course body weight with rsfMRI traits in univariable Mendelian randomization with traditional sensitivity methods.**

| **outcome** | **exposure** | **method** | **beta** | **se** | **lower 95%CI** | **upper 95%CI** | **p-value** |
| --- | --- | --- | --- | --- | --- | --- | --- |
| edge_pheno101 | birth weight | MR Egger | -0.010 | 0.102 | -0.210 | 0.190 | 9.20e-01 |
| edge_pheno101 | birth weight | Weighted median | 0.024 | 0.047 | -0.069 | 0.117 | 6.10e-01 |
| edge_pheno101 | birth weight | Simple mode | 0.033 | 0.122 | -0.207 | 0.272 | 7.88e-01 |
| edge_pheno101 | birth weight | Weighted mode | 0.062 | 0.104 | -0.142 | 0.266 | 5.52e-01 |
| edge_pheno1013 | birth weight | MR Egger | -0.169 | 0.102 | -0.369 | 0.031 | 1.00e-01 |
| edge_pheno1013 | birth weight | Weighted median | 0.017 | 0.051 | -0.083 | 0.117 | 7.35e-01 |
| edge_pheno1013 | birth weight | Simple mode | 0.089 | 0.148 | -0.202 | 0.380 | 5.52e-01 |
| edge_pheno1013 | birth weight | Weighted mode | 0.070 | 0.125 | -0.174 | 0.314 | 5.74e-01 |
| edge_pheno102 | birth weight | MR Egger | 0.080 | 0.105 | -0.126 | 0.285 | 4.49e-01 |
| edge_pheno102 | birth weight | Weighted median | 0.031 | 0.047 | -0.061 | 0.124 | 5.04e-01 |
| edge_pheno102 | birth weight | Simple mode | 0.031 | 0.133 | -0.231 | 0.292 | 8.19e-01 |
| edge_pheno102 | birth weight | Weighted mode | 0.094 | 0.097 | -0.097 | 0.285 | 3.36e-01 |
| edge_pheno1020 | birth weight | MR Egger | 0.039 | 0.101 | -0.159 | 0.236 | 7.02e-01 |
| edge_pheno1020 | birth weight | Weighted median | -0.028 | 0.048 | -0.123 | 0.067 | 5.63e-01 |
| edge_pheno1020 | birth weight | Simple mode | -0.018 | 0.115 | -0.243 | 0.206 | 8.75e-01 |
| edge_pheno1020 | birth weight | Weighted mode | -0.040 | 0.098 | -0.233 | 0.153 | 6.82e-01 |
| edge_pheno1022 | birth weight | MR Egger | 0.022 | 0.101 | -0.176 | 0.219 | 8.31e-01 |
| edge_pheno1022 | birth weight | Weighted median | 0.047 | 0.050 | -0.050 | 0.145 | 3.43e-01 |
| edge_pheno1022 | birth weight | Simple mode | 0.126 | 0.133 | -0.134 | 0.386 | 3.43e-01 |
| edge_pheno1022 | birth weight | Weighted mode | 0.094 | 0.112 | -0.126 | 0.313 | 4.05e-01 |
| edge_pheno103 | birth weight | MR Egger | 0.001 | 0.114 | -0.223 | 0.224 | 9.95e-01 |
| edge_pheno103 | birth weight | Weighted median | 0.070 | 0.049 | -0.025 | 0.165 | 1.48e-01 |
| edge_pheno103 | birth weight | Simple mode | 0.144 | 0.131 | -0.112 | 0.400 | 2.74e-01 |
| edge_pheno103 | birth weight | Weighted mode | 0.158 | 0.097 | -0.032 | 0.347 | 1.05e-01 |
| edge_pheno1041 | birth weight | MR Egger | 0.088 | 0.117 | -0.141 | 0.317 | 4.53e-01 |
| edge_pheno1041 | birth weight | Weighted median | -0.007 | 0.051 | -0.106 | 0.093 | 8.97e-01 |
| edge_pheno1041 | birth weight | Simple mode | 0.055 | 0.139 | -0.218 | 0.328 | 6.92e-01 |
| edge_pheno1041 | birth weight | Weighted mode | 0.075 | 0.105 | -0.131 | 0.280 | 4.78e-01 |
| edge_pheno1059 | birth weight | MR Egger | -0.053 | 0.116 | -0.281 | 0.174 | 6.47e-01 |
| edge_pheno1059 | birth weight | Weighted median | 0.048 | 0.050 | -0.050 | 0.147 | 3.39e-01 |
| edge_pheno1059 | birth weight | Simple mode | 0.127 | 0.131 | -0.130 | 0.385 | 3.34e-01 |
| edge_pheno1059 | birth weight | Weighted mode | 0.067 | 0.090 | -0.109 | 0.243 | 4.58e-01 |
| edge_pheno1122 | birth weight | MR Egger | -0.054 | 0.114 | -0.278 | 0.169 | 6.35e-01 |
| edge_pheno1122 | birth weight | Weighted median | -0.049 | 0.050 | -0.147 | 0.048 | 3.22e-01 |
| edge_pheno1122 | birth weight | Simple mode | -0.059 | 0.123 | -0.301 | 0.183 | 6.32e-01 |
| edge_pheno1122 | birth weight | Weighted mode | -0.086 | 0.095 | -0.272 | 0.100 | 3.67e-01 |
| edge_pheno1126 | birth weight | MR Egger | -0.196 | 0.105 | -0.403 | 0.010 | 6.49e-02 |
| edge_pheno1126 | birth weight | Weighted median | -0.023 | 0.051 | -0.124 | 0.077 | 6.51e-01 |
| edge_pheno1126 | birth weight | Simple mode | -0.028 | 0.138 | -0.299 | 0.242 | 8.38e-01 |
| edge_pheno1126 | birth weight | Weighted mode | -0.043 | 0.127 | -0.292 | 0.206 | 7.35e-01 |
| edge_pheno1134 | birth weight | MR Egger | -0.348 | 0.116 | -0.576 | -0.120 | 3.37e-03 |
| edge_pheno1134 | birth weight | Weighted median | -0.126 | 0.050 | -0.225 | -0.028 | 1.19e-02 |
| edge_pheno1134 | birth weight | Simple mode | -0.120 | 0.126 | -0.368 | 0.128 | 3.45e-01 |
| edge_pheno1134 | birth weight | Weighted mode | -0.087 | 0.104 | -0.291 | 0.117 | 4.05e-01 |
| edge_pheno1137 | birth weight | MR Egger | 0.065 | 0.113 | -0.157 | 0.287 | 5.67e-01 |
| edge_pheno1137 | birth weight | Weighted median | 0.023 | 0.050 | -0.074 | 0.120 | 6.41e-01 |
| edge_pheno1137 | birth weight | Simple mode | 0.003 | 0.137 | -0.265 | 0.272 | 9.81e-01 |
| edge_pheno1137 | birth weight | Weighted mode | -0.042 | 0.107 | -0.251 | 0.167 | 6.94e-01 |
| edge_pheno1141 | birth weight | MR Egger | -0.010 | 0.107 | -0.219 | 0.199 | 9.23e-01 |
| edge_pheno1141 | birth weight | Weighted median | 0.013 | 0.047 | -0.079 | 0.105 | 7.79e-01 |
| edge_pheno1141 | birth weight | Simple mode | 0.077 | 0.130 | -0.179 | 0.333 | 5.55e-01 |
| edge_pheno1141 | birth weight | Weighted mode | 0.060 | 0.108 | -0.152 | 0.273 | 5.79e-01 |
| edge_pheno1142 | birth weight | MR Egger | -0.158 | 0.101 | -0.356 | 0.041 | 1.22e-01 |
| edge_pheno1142 | birth weight | Weighted median | 0.031 | 0.047 | -0.061 | 0.123 | 5.12e-01 |
| edge_pheno1142 | birth weight | Simple mode | 0.165 | 0.135 | -0.099 | 0.429 | 2.24e-01 |
| edge_pheno1142 | birth weight | Weighted mode | 0.124 | 0.124 | -0.119 | 0.367 | 3.20e-01 |
| edge_pheno1161 | birth weight | MR Egger | 0.069 | 0.119 | -0.165 | 0.303 | 5.64e-01 |
| edge_pheno1161 | birth weight | Weighted median | 0.058 | 0.050 | -0.041 | 0.157 | 2.51e-01 |
| edge_pheno1161 | birth weight | Simple mode | -0.073 | 0.136 | -0.340 | 0.193 | 5.91e-01 |
| edge_pheno1161 | birth weight | Weighted mode | 0.024 | 0.105 | -0.181 | 0.230 | 8.18e-01 |
| edge_pheno1167 | birth weight | MR Egger | 0.033 | 0.119 | -0.201 | 0.266 | 7.84e-01 |
| edge_pheno1167 | birth weight | Weighted median | 0.028 | 0.053 | -0.075 | 0.131 | 5.92e-01 |
| edge_pheno1167 | birth weight | Simple mode | -0.118 | 0.146 | -0.403 | 0.168 | 4.20e-01 |
| edge_pheno1167 | birth weight | Weighted mode | -0.009 | 0.109 | -0.223 | 0.205 | 9.36e-01 |
| edge_pheno1171 | birth weight | MR Egger | 0.092 | 0.114 | -0.131 | 0.316 | 4.18e-01 |
| edge_pheno1171 | birth weight | Weighted median | -0.004 | 0.051 | -0.104 | 0.096 | 9.34e-01 |
| edge_pheno1171 | birth weight | Simple mode | -0.053 | 0.134 | -0.315 | 0.209 | 6.91e-01 |
| edge_pheno1171 | birth weight | Weighted mode | -0.022 | 0.106 | -0.230 | 0.186 | 8.36e-01 |
| edge_pheno1175 | birth weight | MR Egger | 0.081 | 0.119 | -0.153 | 0.315 | 4.98e-01 |
| edge_pheno1175 | birth weight | Weighted median | 0.045 | 0.050 | -0.053 | 0.142 | 3.68e-01 |
| edge_pheno1175 | birth weight | Simple mode | 0.259 | 0.159 | -0.052 | 0.570 | 1.06e-01 |
| edge_pheno1175 | birth weight | Weighted mode | 0.224 | 0.122 | -0.014 | 0.463 | 6.77e-02 |
| edge_pheno1183 | birth weight | MR Egger | -0.110 | 0.100 | -0.307 | 0.086 | 2.73e-01 |
| edge_pheno1183 | birth weight | Weighted median | -0.093 | 0.050 | -0.191 | 0.004 | 6.14e-02 |
| edge_pheno1183 | birth weight | Simple mode | -0.092 | 0.128 | -0.343 | 0.158 | 4.73e-01 |
| edge_pheno1183 | birth weight | Weighted mode | -0.121 | 0.092 | -0.301 | 0.059 | 1.91e-01 |
| edge_pheno1184 | birth weight | MR Egger | -0.197 | 0.121 | -0.435 | 0.040 | 1.06e-01 |
| edge_pheno1184 | birth weight | Weighted median | 0.021 | 0.051 | -0.078 | 0.120 | 6.74e-01 |
| edge_pheno1184 | birth weight | Simple mode | 0.101 | 0.145 | -0.183 | 0.385 | 4.87e-01 |
| edge_pheno1184 | birth weight | Weighted mode | 0.094 | 0.133 | -0.168 | 0.355 | 4.83e-01 |
| edge_pheno1189 | birth weight | MR Egger | -0.137 | 0.112 | -0.356 | 0.082 | 2.22e-01 |
| edge_pheno1189 | birth weight | Weighted median | -0.052 | 0.048 | -0.145 | 0.041 | 2.77e-01 |
| edge_pheno1189 | birth weight | Simple mode | 0.021 | 0.125 | -0.225 | 0.266 | 8.70e-01 |
| edge_pheno1189 | birth weight | Weighted mode | 0.042 | 0.119 | -0.192 | 0.276 | 7.28e-01 |
| edge_pheno1205 | birth weight | MR Egger | -0.109 | 0.106 | -0.316 | 0.098 | 3.04e-01 |
| edge_pheno1205 | birth weight | Weighted median | -0.057 | 0.048 | -0.151 | 0.037 | 2.32e-01 |
| edge_pheno1205 | birth weight | Simple mode | -0.112 | 0.127 | -0.360 | 0.137 | 3.80e-01 |
| edge_pheno1205 | birth weight | Weighted mode | -0.047 | 0.090 | -0.223 | 0.128 | 5.99e-01 |
| edge_pheno1211 | birth weight | MR Egger | -0.208 | 0.111 | -0.425 | 0.009 | 6.25e-02 |
| edge_pheno1211 | birth weight | Weighted median | -0.053 | 0.051 | -0.153 | 0.047 | 2.98e-01 |
| edge_pheno1211 | birth weight | Simple mode | 0.061 | 0.129 | -0.192 | 0.314 | 6.39e-01 |
| edge_pheno1211 | birth weight | Weighted mode | -0.052 | 0.095 | -0.237 | 0.134 | 5.87e-01 |
| edge_pheno1221 | birth weight | MR Egger | -0.033 | 0.114 | -0.257 | 0.191 | 7.75e-01 |
| edge_pheno1221 | birth weight | Weighted median | -0.005 | 0.052 | -0.107 | 0.097 | 9.19e-01 |
| edge_pheno1221 | birth weight | Simple mode | -0.295 | 0.160 | -0.609 | 0.019 | 6.80e-02 |
| edge_pheno1221 | birth weight | Weighted mode | 0.173 | 0.161 | -0.143 | 0.489 | 2.87e-01 |
| edge_pheno1225 | birth weight | MR Egger | 0.017 | 0.104 | -0.186 | 0.221 | 8.67e-01 |
| edge_pheno1225 | birth weight | Weighted median | 0.021 | 0.047 | -0.070 | 0.112 | 6.52e-01 |
| edge_pheno1225 | birth weight | Simple mode | -0.054 | 0.119 | -0.286 | 0.179 | 6.53e-01 |
| edge_pheno1225 | birth weight | Weighted mode | -0.019 | 0.102 | -0.219 | 0.180 | 8.49e-01 |
| edge_pheno1250 | birth weight | MR Egger | -0.006 | 0.113 | -0.228 | 0.216 | 9.59e-01 |
| edge_pheno1250 | birth weight | Weighted median | -0.052 | 0.049 | -0.148 | 0.045 | 2.93e-01 |
| edge_pheno1250 | birth weight | Simple mode | 0.001 | 0.137 | -0.267 | 0.269 | 9.95e-01 |
| edge_pheno1250 | birth weight | Weighted mode | -0.067 | 0.111 | -0.284 | 0.151 | 5.49e-01 |
| edge_pheno1256 | birth weight | MR Egger | -0.063 | 0.106 | -0.271 | 0.145 | 5.53e-01 |
| edge_pheno1256 | birth weight | Weighted median | -0.063 | 0.049 | -0.159 | 0.033 | 2.01e-01 |
| edge_pheno1256 | birth weight | Simple mode | -0.063 | 0.128 | -0.314 | 0.187 | 6.21e-01 |
| edge_pheno1256 | birth weight | Weighted mode | -0.100 | 0.098 | -0.292 | 0.091 | 3.07e-01 |
| edge_pheno1257 | birth weight | MR Egger | -0.158 | 0.100 | -0.355 | 0.038 | 1.17e-01 |
| edge_pheno1257 | birth weight | Weighted median | -0.052 | 0.050 | -0.149 | 0.045 | 2.94e-01 |
| edge_pheno1257 | birth weight | Simple mode | -0.108 | 0.128 | -0.358 | 0.142 | 4.00e-01 |
| edge_pheno1257 | birth weight | Weighted mode | -0.051 | 0.101 | -0.248 | 0.146 | 6.11e-01 |
| edge_pheno1269 | birth weight | MR Egger | -0.005 | 0.100 | -0.201 | 0.191 | 9.60e-01 |
| edge_pheno1269 | birth weight | Weighted median | -0.014 | 0.051 | -0.114 | 0.085 | 7.76e-01 |
| edge_pheno1269 | birth weight | Simple mode | 0.126 | 0.129 | -0.127 | 0.379 | 3.31e-01 |
| edge_pheno1269 | birth weight | Weighted mode | -0.029 | 0.103 | -0.231 | 0.173 | 7.81e-01 |
| edge_pheno1270 | birth weight | MR Egger | 0.061 | 0.112 | -0.158 | 0.280 | 5.85e-01 |
| edge_pheno1270 | birth weight | Weighted median | -0.024 | 0.049 | -0.120 | 0.073 | 6.30e-01 |
| edge_pheno1270 | birth weight | Simple mode | -0.010 | 0.134 | -0.272 | 0.252 | 9.38e-01 |
| edge_pheno1270 | birth weight | Weighted mode | 0.021 | 0.093 | -0.161 | 0.204 | 8.19e-01 |
| edge_pheno1273 | birth weight | MR Egger | 0.085 | 0.112 | -0.134 | 0.304 | 4.49e-01 |
| edge_pheno1273 | birth weight | Weighted median | 0.042 | 0.050 | -0.057 | 0.140 | 4.09e-01 |
| edge_pheno1273 | birth weight | Simple mode | 0.099 | 0.137 | -0.170 | 0.368 | 4.72e-01 |
| edge_pheno1273 | birth weight | Weighted mode | 0.080 | 0.113 | -0.142 | 0.302 | 4.81e-01 |
| edge_pheno1276 | birth weight | MR Egger | -0.030 | 0.111 | -0.247 | 0.188 | 7.89e-01 |
| edge_pheno1276 | birth weight | Weighted median | -0.057 | 0.048 | -0.152 | 0.038 | 2.37e-01 |
| edge_pheno1276 | birth weight | Simple mode | -0.055 | 0.146 | -0.342 | 0.232 | 7.07e-01 |
| edge_pheno1276 | birth weight | Weighted mode | -0.225 | 0.118 | -0.456 | 0.006 | 5.85e-02 |
| edge_pheno1293 | birth weight | MR Egger | -0.043 | 0.123 | -0.283 | 0.198 | 7.28e-01 |
| edge_pheno1293 | birth weight | Weighted median | -0.023 | 0.051 | -0.123 | 0.078 | 6.59e-01 |
| edge_pheno1293 | birth weight | Simple mode | 0.169 | 0.175 | -0.175 | 0.513 | 3.37e-01 |
| edge_pheno1293 | birth weight | Weighted mode | -0.215 | 0.114 | -0.439 | 0.010 | 6.30e-02 |
| edge_pheno1296 | birth weight | MR Egger | 0.025 | 0.108 | -0.186 | 0.236 | 8.20e-01 |
| edge_pheno1296 | birth weight | Weighted median | 0.004 | 0.048 | -0.090 | 0.098 | 9.36e-01 |
| edge_pheno1296 | birth weight | Simple mode | -0.020 | 0.117 | -0.249 | 0.208 | 8.62e-01 |
| edge_pheno1296 | birth weight | Weighted mode | 0.003 | 0.087 | -0.167 | 0.173 | 9.75e-01 |
| edge_pheno1300 | birth weight | MR Egger | 0.030 | 0.116 | -0.197 | 0.256 | 7.97e-01 |
| edge_pheno1300 | birth weight | Weighted median | 0.000 | 0.050 | -0.097 | 0.097 | 9.94e-01 |
| edge_pheno1300 | birth weight | Simple mode | -0.083 | 0.133 | -0.344 | 0.179 | 5.35e-01 |
| edge_pheno1300 | birth weight | Weighted mode | -0.026 | 0.098 | -0.218 | 0.166 | 7.90e-01 |
| edge_pheno1301 | birth weight | MR Egger | 0.177 | 0.102 | -0.024 | 0.377 | 8.70e-02 |
| edge_pheno1301 | birth weight | Weighted median | 0.053 | 0.048 | -0.041 | 0.147 | 2.69e-01 |
| edge_pheno1301 | birth weight | Simple mode | 0.063 | 0.124 | -0.180 | 0.305 | 6.14e-01 |
| edge_pheno1301 | birth weight | Weighted mode | 0.084 | 0.101 | -0.115 | 0.282 | 4.10e-01 |
| edge_pheno1302 | birth weight | MR Egger | -0.082 | 0.101 | -0.281 | 0.117 | 4.23e-01 |
| edge_pheno1302 | birth weight | Weighted median | -0.011 | 0.049 | -0.107 | 0.086 | 8.30e-01 |
| edge_pheno1302 | birth weight | Simple mode | 0.038 | 0.118 | -0.193 | 0.269 | 7.49e-01 |
| edge_pheno1302 | birth weight | Weighted mode | 0.004 | 0.096 | -0.183 | 0.192 | 9.65e-01 |
| edge_pheno1309 | birth weight | MR Egger | 0.009 | 0.113 | -0.212 | 0.230 | 9.37e-01 |
| edge_pheno1309 | birth weight | Weighted median | -0.077 | 0.047 | -0.169 | 0.016 | 1.04e-01 |
| edge_pheno1309 | birth weight | Simple mode | -0.204 | 0.131 | -0.461 | 0.053 | 1.23e-01 |
| edge_pheno1309 | birth weight | Weighted mode | -0.197 | 0.110 | -0.413 | 0.019 | 7.65e-02 |
| edge_pheno1311 | birth weight | MR Egger | -0.048 | 0.114 | -0.271 | 0.176 | 6.77e-01 |
| edge_pheno1311 | birth weight | Weighted median | 0.067 | 0.058 | -0.047 | 0.181 | 2.47e-01 |
| edge_pheno1311 | birth weight | Simple mode | 0.048 | 0.146 | -0.238 | 0.335 | 7.41e-01 |
| edge_pheno1311 | birth weight | Weighted mode | 0.080 | 0.127 | -0.170 | 0.330 | 5.34e-01 |
| edge_pheno1317 | birth weight | MR Egger | -0.079 | 0.133 | -0.340 | 0.181 | 5.51e-01 |
| edge_pheno1317 | birth weight | Weighted median | -0.015 | 0.057 | -0.127 | 0.098 | 7.98e-01 |
| edge_pheno1317 | birth weight | Simple mode | 0.078 | 0.129 | -0.175 | 0.331 | 5.47e-01 |
| edge_pheno1317 | birth weight | Weighted mode | -0.015 | 0.095 | -0.201 | 0.171 | 8.74e-01 |
| edge_pheno1319 | birth weight | MR Egger | -0.098 | 0.100 | -0.294 | 0.098 | 3.30e-01 |
| edge_pheno1319 | birth weight | Weighted median | 0.016 | 0.046 | -0.074 | 0.106 | 7.27e-01 |
| edge_pheno1319 | birth weight | Simple mode | 0.087 | 0.126 | -0.159 | 0.334 | 4.88e-01 |
| edge_pheno1319 | birth weight | Weighted mode | 0.016 | 0.091 | -0.163 | 0.194 | 8.64e-01 |
| edge_pheno132 | birth weight | MR Egger | 0.057 | 0.108 | -0.155 | 0.270 | 6.00e-01 |
| edge_pheno132 | birth weight | Weighted median | 0.012 | 0.048 | -0.083 | 0.107 | 8.01e-01 |
| edge_pheno132 | birth weight | Simple mode | 0.162 | 0.135 | -0.103 | 0.427 | 2.34e-01 |
| edge_pheno132 | birth weight | Weighted mode | 0.079 | 0.118 | -0.153 | 0.310 | 5.07e-01 |
| edge_pheno1322 | birth weight | MR Egger | 0.062 | 0.105 | -0.143 | 0.268 | 5.53e-01 |
| edge_pheno1322 | birth weight | Weighted median | 0.011 | 0.047 | -0.080 | 0.103 | 8.09e-01 |
| edge_pheno1322 | birth weight | Simple mode | 0.012 | 0.124 | -0.231 | 0.255 | 9.24e-01 |
| edge_pheno1322 | birth weight | Weighted mode | 0.023 | 0.114 | -0.201 | 0.247 | 8.42e-01 |
| edge_pheno1325 | birth weight | MR Egger | 0.002 | 0.107 | -0.208 | 0.212 | 9.86e-01 |
| edge_pheno1325 | birth weight | Weighted median | -0.058 | 0.048 | -0.151 | 0.035 | 2.24e-01 |
| edge_pheno1325 | birth weight | Simple mode | -0.136 | 0.131 | -0.392 | 0.120 | 3.01e-01 |
| edge_pheno1325 | birth weight | Weighted mode | -0.112 | 0.096 | -0.300 | 0.076 | 2.44e-01 |
| edge_pheno1328 | birth weight | MR Egger | -0.045 | 0.118 | -0.276 | 0.186 | 7.06e-01 |
| edge_pheno1328 | birth weight | Weighted median | -0.099 | 0.051 | -0.200 | 0.001 | 5.32e-02 |
| edge_pheno1328 | birth weight | Simple mode | -0.152 | 0.151 | -0.447 | 0.143 | 3.14e-01 |
| edge_pheno1328 | birth weight | Weighted mode | -0.146 | 0.113 | -0.367 | 0.074 | 1.96e-01 |
| edge_pheno135 | birth weight | MR Egger | -0.103 | 0.104 | -0.308 | 0.101 | 3.25e-01 |
| edge_pheno135 | birth weight | Weighted median | 0.015 | 0.049 | -0.081 | 0.111 | 7.59e-01 |
| edge_pheno135 | birth weight | Simple mode | -0.018 | 0.128 | -0.268 | 0.232 | 8.87e-01 |
| edge_pheno135 | birth weight | Weighted mode | 0.013 | 0.105 | -0.193 | 0.218 | 9.03e-01 |
| edge_pheno1359 | birth weight | MR Egger | 0.055 | 0.116 | -0.172 | 0.283 | 6.34e-01 |
| edge_pheno1359 | birth weight | Weighted median | 0.020 | 0.051 | -0.081 | 0.121 | 6.96e-01 |
| edge_pheno1359 | birth weight | Simple mode | -0.059 | 0.142 | -0.337 | 0.219 | 6.79e-01 |
| edge_pheno1359 | birth weight | Weighted mode | -0.028 | 0.111 | -0.247 | 0.190 | 8.00e-01 |
| edge_pheno1382 | birth weight | MR Egger | -0.080 | 0.110 | -0.295 | 0.135 | 4.66e-01 |
| edge_pheno1382 | birth weight | Weighted median | -0.071 | 0.050 | -0.168 | 0.026 | 1.52e-01 |
| edge_pheno1382 | birth weight | Simple mode | -0.113 | 0.139 | -0.384 | 0.159 | 4.18e-01 |
| edge_pheno1382 | birth weight | Weighted mode | -0.119 | 0.103 | -0.320 | 0.082 | 2.49e-01 |
| edge_pheno146 | birth weight | MR Egger | 0.002 | 0.106 | -0.205 | 0.209 | 9.83e-01 |
| edge_pheno146 | birth weight | Weighted median | 0.053 | 0.049 | -0.042 | 0.149 | 2.74e-01 |
| edge_pheno146 | birth weight | Simple mode | 0.116 | 0.128 | -0.135 | 0.368 | 3.67e-01 |
| edge_pheno146 | birth weight | Weighted mode | 0.116 | 0.104 | -0.087 | 0.319 | 2.65e-01 |
| edge_pheno147 | birth weight | MR Egger | 0.078 | 0.103 | -0.124 | 0.279 | 4.51e-01 |
| edge_pheno147 | birth weight | Weighted median | 0.011 | 0.048 | -0.083 | 0.105 | 8.19e-01 |
| edge_pheno147 | birth weight | Simple mode | -0.042 | 0.123 | -0.284 | 0.200 | 7.37e-01 |
| edge_pheno147 | birth weight | Weighted mode | -0.004 | 0.099 | -0.197 | 0.190 | 9.69e-01 |
| edge_pheno151 | birth weight | MR Egger | 0.023 | 0.105 | -0.184 | 0.229 | 8.30e-01 |
| edge_pheno151 | birth weight | Weighted median | -0.032 | 0.048 | -0.126 | 0.062 | 5.03e-01 |
| edge_pheno151 | birth weight | Simple mode | -0.101 | 0.132 | -0.360 | 0.158 | 4.46e-01 |
| edge_pheno151 | birth weight | Weighted mode | -0.038 | 0.090 | -0.215 | 0.139 | 6.76e-01 |
| edge_pheno1696 | birth weight | MR Egger | 0.057 | 0.113 | -0.165 | 0.278 | 6.18e-01 |
| edge_pheno1696 | birth weight | Weighted median | -0.068 | 0.050 | -0.165 | 0.030 | 1.73e-01 |
| edge_pheno1696 | birth weight | Simple mode | -0.087 | 0.122 | -0.325 | 0.152 | 4.78e-01 |
| edge_pheno1696 | birth weight | Weighted mode | -0.129 | 0.101 | -0.327 | 0.068 | 2.02e-01 |
| edge_pheno1697 | birth weight | MR Egger | -0.046 | 0.129 | -0.299 | 0.206 | 7.20e-01 |
| edge_pheno1697 | birth weight | Weighted median | -0.037 | 0.050 | -0.136 | 0.061 | 4.57e-01 |
| edge_pheno1697 | birth weight | Simple mode | -0.045 | 0.128 | -0.296 | 0.206 | 7.24e-01 |
| edge_pheno1697 | birth weight | Weighted mode | -0.061 | 0.105 | -0.266 | 0.144 | 5.61e-01 |
| edge_pheno1698 | birth weight | MR Egger | -0.031 | 0.121 | -0.268 | 0.206 | 7.96e-01 |
| edge_pheno1698 | birth weight | Weighted median | -0.034 | 0.051 | -0.133 | 0.065 | 5.01e-01 |
| edge_pheno1698 | birth weight | Simple mode | -0.029 | 0.112 | -0.249 | 0.191 | 7.97e-01 |
| edge_pheno1698 | birth weight | Weighted mode | -0.053 | 0.098 | -0.246 | 0.140 | 5.90e-01 |
| edge_pheno1699 | birth weight | MR Egger | -0.071 | 0.122 | -0.310 | 0.168 | 5.62e-01 |
| edge_pheno1699 | birth weight | Weighted median | -0.001 | 0.046 | -0.090 | 0.088 | 9.81e-01 |
| edge_pheno1699 | birth weight | Simple mode | 0.010 | 0.117 | -0.219 | 0.239 | 9.32e-01 |
| edge_pheno1699 | birth weight | Weighted mode | 0.025 | 0.091 | -0.154 | 0.203 | 7.87e-01 |
| edge_pheno1701 | birth weight | MR Egger | 0.124 | 0.122 | -0.115 | 0.363 | 3.12e-01 |
| edge_pheno1701 | birth weight | Weighted median | 0.068 | 0.053 | -0.035 | 0.171 | 1.96e-01 |
| edge_pheno1701 | birth weight | Simple mode | 0.011 | 0.137 | -0.258 | 0.279 | 9.39e-01 |
| edge_pheno1701 | birth weight | Weighted mode | 0.032 | 0.104 | -0.172 | 0.235 | 7.61e-01 |
| edge_pheno249 | birth weight | MR Egger | -0.001 | 0.112 | -0.220 | 0.218 | 9.96e-01 |
| edge_pheno249 | birth weight | Weighted median | 0.076 | 0.048 | -0.018 | 0.169 | 1.12e-01 |
| edge_pheno249 | birth weight | Simple mode | 0.157 | 0.129 | -0.097 | 0.410 | 2.29e-01 |
| edge_pheno249 | birth weight | Weighted mode | 0.139 | 0.091 | -0.039 | 0.318 | 1.28e-01 |
| edge_pheno253 | birth weight | MR Egger | 0.003 | 0.101 | -0.194 | 0.201 | 9.72e-01 |
| edge_pheno253 | birth weight | Weighted median | 0.041 | 0.049 | -0.054 | 0.137 | 3.94e-01 |
| edge_pheno253 | birth weight | Simple mode | 0.035 | 0.133 | -0.225 | 0.295 | 7.91e-01 |
| edge_pheno253 | birth weight | Weighted mode | 0.024 | 0.117 | -0.206 | 0.254 | 8.37e-01 |
| edge_pheno262 | birth weight | MR Egger | 0.071 | 0.100 | -0.124 | 0.266 | 4.76e-01 |
| edge_pheno262 | birth weight | Weighted median | 0.023 | 0.046 | -0.068 | 0.113 | 6.26e-01 |
| edge_pheno262 | birth weight | Simple mode | 0.053 | 0.113 | -0.168 | 0.274 | 6.40e-01 |
| edge_pheno262 | birth weight | Weighted mode | 0.053 | 0.095 | -0.133 | 0.239 | 5.79e-01 |
| edge_pheno286 | birth weight | MR Egger | -0.006 | 0.104 | -0.210 | 0.198 | 9.55e-01 |
| edge_pheno286 | birth weight | Weighted median | -0.033 | 0.048 | -0.128 | 0.062 | 4.94e-01 |
| edge_pheno286 | birth weight | Simple mode | -0.084 | 0.123 | -0.326 | 0.158 | 4.99e-01 |
| edge_pheno286 | birth weight | Weighted mode | -0.041 | 0.097 | -0.231 | 0.148 | 6.69e-01 |
| edge_pheno288 | birth weight | MR Egger | -0.064 | 0.101 | -0.261 | 0.133 | 5.28e-01 |
| edge_pheno288 | birth weight | Weighted median | 0.007 | 0.048 | -0.088 | 0.102 | 8.87e-01 |
| edge_pheno288 | birth weight | Simple mode | -0.041 | 0.119 | -0.274 | 0.193 | 7.33e-01 |
| edge_pheno288 | birth weight | Weighted mode | -0.036 | 0.090 | -0.213 | 0.141 | 6.92e-01 |
| edge_pheno303 | birth weight | MR Egger | -0.163 | 0.109 | -0.378 | 0.051 | 1.38e-01 |
| edge_pheno303 | birth weight | Weighted median | -0.045 | 0.050 | -0.143 | 0.054 | 3.76e-01 |
| edge_pheno303 | birth weight | Simple mode | 0.018 | 0.160 | -0.295 | 0.332 | 9.09e-01 |
| edge_pheno303 | birth weight | Weighted mode | -0.105 | 0.112 | -0.324 | 0.115 | 3.52e-01 |
| edge_pheno389 | birth weight | MR Egger | 0.145 | 0.105 | -0.060 | 0.351 | 1.69e-01 |
| edge_pheno389 | birth weight | Weighted median | 0.020 | 0.050 | -0.079 | 0.118 | 6.97e-01 |
| edge_pheno389 | birth weight | Simple mode | -0.029 | 0.139 | -0.301 | 0.242 | 8.32e-01 |
| edge_pheno389 | birth weight | Weighted mode | -0.058 | 0.127 | -0.306 | 0.190 | 6.46e-01 |
| edge_pheno405 | birth weight | MR Egger | -0.016 | 0.099 | -0.209 | 0.178 | 8.75e-01 |
| edge_pheno405 | birth weight | Weighted median | -0.040 | 0.048 | -0.134 | 0.054 | 4.03e-01 |
| edge_pheno405 | birth weight | Simple mode | -0.034 | 0.123 | -0.276 | 0.208 | 7.82e-01 |
| edge_pheno405 | birth weight | Weighted mode | -0.055 | 0.086 | -0.224 | 0.114 | 5.27e-01 |
| edge_pheno447 | birth weight | MR Egger | 0.337 | 0.484 | -0.611 | 1.285 | 5.08e-01 |
| edge_pheno447 | birth weight | Weighted median | 0.273 | 0.163 | -0.046 | 0.592 | 9.40e-02 |
| edge_pheno447 | birth weight | Simple mode | 0.372 | 0.240 | -0.098 | 0.842 | 1.59e-01 |
| edge_pheno447 | birth weight | Weighted mode | 0.331 | 0.246 | -0.151 | 0.814 | 2.15e-01 |
| edge_pheno449 | birth weight | MR Egger | -0.141 | 0.518 | -1.156 | 0.875 | 7.97e-01 |
| edge_pheno449 | birth weight | Weighted median | -0.201 | 0.176 | -0.547 | 0.144 | 2.53e-01 |
| edge_pheno449 | birth weight | Simple mode | -0.249 | 0.254 | -0.746 | 0.248 | 3.63e-01 |
| edge_pheno449 | birth weight | Weighted mode | -0.242 | 0.234 | -0.700 | 0.216 | 3.40e-01 |
| edge_pheno460 | birth weight | MR Egger | 0.146 | 0.113 | -0.076 | 0.367 | 2.00e-01 |
| edge_pheno460 | birth weight | Weighted median | 0.051 | 0.050 | -0.047 | 0.149 | 3.08e-01 |
| edge_pheno460 | birth weight | Simple mode | 0.168 | 0.126 | -0.079 | 0.415 | 1.85e-01 |
| edge_pheno460 | birth weight | Weighted mode | 0.124 | 0.103 | -0.077 | 0.325 | 2.30e-01 |
| edge_pheno461 | birth weight | MR Egger | -0.095 | 0.113 | -0.316 | 0.125 | 3.98e-01 |
| edge_pheno461 | birth weight | Weighted median | 0.011 | 0.051 | -0.089 | 0.111 | 8.30e-01 |
| edge_pheno461 | birth weight | Simple mode | 0.088 | 0.154 | -0.213 | 0.389 | 5.68e-01 |
| edge_pheno461 | birth weight | Weighted mode | 0.094 | 0.181 | -0.260 | 0.448 | 6.04e-01 |
| edge_pheno491 | birth weight | MR Egger | 0.087 | 0.103 | -0.114 | 0.289 | 3.98e-01 |
| edge_pheno491 | birth weight | Weighted median | 0.044 | 0.049 | -0.052 | 0.140 | 3.69e-01 |
| edge_pheno491 | birth weight | Simple mode | 0.039 | 0.120 | -0.197 | 0.275 | 7.49e-01 |
| edge_pheno491 | birth weight | Weighted mode | 0.039 | 0.099 | -0.155 | 0.232 | 6.97e-01 |
| edge_pheno537 | birth weight | MR Egger | -0.109 | 0.111 | -0.326 | 0.108 | 3.29e-01 |
| edge_pheno537 | birth weight | Weighted median | -0.019 | 0.047 | -0.111 | 0.073 | 6.91e-01 |
| edge_pheno537 | birth weight | Simple mode | 0.034 | 0.128 | -0.216 | 0.284 | 7.92e-01 |
| edge_pheno537 | birth weight | Weighted mode | 0.040 | 0.099 | -0.154 | 0.234 | 6.87e-01 |
| edge_pheno55 | birth weight | MR Egger | 0.015 | 0.114 | -0.208 | 0.237 | 8.97e-01 |
| edge_pheno55 | birth weight | Weighted median | 0.004 | 0.051 | -0.096 | 0.105 | 9.31e-01 |
| edge_pheno55 | birth weight | Simple mode | 0.003 | 0.149 | -0.290 | 0.296 | 9.84e-01 |
| edge_pheno55 | birth weight | Weighted mode | -0.074 | 0.135 | -0.339 | 0.191 | 5.84e-01 |
| edge_pheno558 | birth weight | MR Egger | 0.040 | 0.104 | -0.165 | 0.245 | 7.02e-01 |
| edge_pheno558 | birth weight | Weighted median | 0.028 | 0.050 | -0.070 | 0.127 | 5.71e-01 |
| edge_pheno558 | birth weight | Simple mode | 0.082 | 0.130 | -0.173 | 0.336 | 5.30e-01 |
| edge_pheno558 | birth weight | Weighted mode | 0.027 | 0.118 | -0.204 | 0.259 | 8.18e-01 |
| edge_pheno574 | birth weight | MR Egger | -0.016 | 0.100 | -0.211 | 0.180 | 8.75e-01 |
| edge_pheno574 | birth weight | Weighted median | -0.028 | 0.048 | -0.123 | 0.067 | 5.62e-01 |
| edge_pheno574 | birth weight | Simple mode | -0.065 | 0.128 | -0.316 | 0.185 | 6.10e-01 |
| edge_pheno574 | birth weight | Weighted mode | -0.111 | 0.106 | -0.319 | 0.097 | 2.98e-01 |
| edge_pheno58 | birth weight | MR Egger | 0.027 | 0.096 | -0.162 | 0.216 | 7.78e-01 |
| edge_pheno58 | birth weight | Weighted median | 0.036 | 0.044 | -0.051 | 0.123 | 4.12e-01 |
| edge_pheno58 | birth weight | Simple mode | -0.064 | 0.109 | -0.278 | 0.150 | 5.59e-01 |
| edge_pheno58 | birth weight | Weighted mode | 0.014 | 0.091 | -0.163 | 0.192 | 8.74e-01 |
| edge_pheno590 | birth weight | MR Egger | -0.039 | 0.111 | -0.257 | 0.178 | 7.24e-01 |
| edge_pheno590 | birth weight | Weighted median | -0.006 | 0.050 | -0.103 | 0.092 | 9.09e-01 |
| edge_pheno590 | birth weight | Simple mode | 0.046 | 0.126 | -0.201 | 0.293 | 7.16e-01 |
| edge_pheno590 | birth weight | Weighted mode | 0.004 | 0.093 | -0.179 | 0.186 | 9.68e-01 |
| edge_pheno593 | birth weight | MR Egger | -0.079 | 0.105 | -0.285 | 0.128 | 4.56e-01 |
| edge_pheno593 | birth weight | Weighted median | -0.093 | 0.049 | -0.188 | 0.003 | 5.64e-02 |
| edge_pheno593 | birth weight | Simple mode | -0.069 | 0.116 | -0.297 | 0.159 | 5.54e-01 |
| edge_pheno593 | birth weight | Weighted mode | -0.093 | 0.094 | -0.277 | 0.091 | 3.24e-01 |
| edge_pheno597 | birth weight | MR Egger | -0.045 | 0.119 | -0.278 | 0.189 | 7.09e-01 |
| edge_pheno597 | birth weight | Weighted median | -0.070 | 0.048 | -0.164 | 0.025 | 1.49e-01 |
| edge_pheno597 | birth weight | Simple mode | -0.127 | 0.111 | -0.344 | 0.090 | 2.52e-01 |
| edge_pheno597 | birth weight | Weighted mode | -0.086 | 0.085 | -0.252 | 0.080 | 3.12e-01 |
| edge_pheno599 | birth weight | MR Egger | -0.150 | 0.103 | -0.352 | 0.052 | 1.47e-01 |
| edge_pheno599 | birth weight | Weighted median | 0.001 | 0.048 | -0.093 | 0.095 | 9.81e-01 |
| edge_pheno599 | birth weight | Simple mode | 0.042 | 0.145 | -0.243 | 0.326 | 7.75e-01 |
| edge_pheno599 | birth weight | Weighted mode | -0.052 | 0.098 | -0.244 | 0.139 | 5.94e-01 |
| edge_pheno601 | birth weight | MR Egger | -0.033 | 0.101 | -0.231 | 0.164 | 7.42e-01 |
| edge_pheno601 | birth weight | Weighted median | -0.060 | 0.047 | -0.152 | 0.033 | 2.09e-01 |
| edge_pheno601 | birth weight | Simple mode | -0.092 | 0.125 | -0.337 | 0.154 | 4.67e-01 |
| edge_pheno601 | birth weight | Weighted mode | -0.092 | 0.100 | -0.288 | 0.105 | 3.63e-01 |
| edge_pheno606 | birth weight | MR Egger | 0.146 | 0.102 | -0.054 | 0.347 | 1.55e-01 |
| edge_pheno606 | birth weight | Weighted median | 0.010 | 0.050 | -0.088 | 0.107 | 8.48e-01 |
| edge_pheno606 | birth weight | Simple mode | -0.076 | 0.126 | -0.324 | 0.171 | 5.47e-01 |
| edge_pheno606 | birth weight | Weighted mode | 0.025 | 0.109 | -0.190 | 0.239 | 8.23e-01 |
| edge_pheno609 | birth weight | MR Egger | -0.072 | 0.110 | -0.288 | 0.144 | 5.15e-01 |
| edge_pheno609 | birth weight | Weighted median | 0.007 | 0.049 | -0.088 | 0.102 | 8.86e-01 |
| edge_pheno609 | birth weight | Simple mode | -0.007 | 0.137 | -0.276 | 0.262 | 9.60e-01 |
| edge_pheno609 | birth weight | Weighted mode | 0.060 | 0.113 | -0.162 | 0.282 | 5.97e-01 |
| edge_pheno621 | birth weight | MR Egger | 0.054 | 0.105 | -0.152 | 0.260 | 6.08e-01 |
| edge_pheno621 | birth weight | Weighted median | 0.017 | 0.053 | -0.087 | 0.120 | 7.52e-01 |
| edge_pheno621 | birth weight | Simple mode | -0.032 | 0.145 | -0.317 | 0.253 | 8.27e-01 |
| edge_pheno621 | birth weight | Weighted mode | -0.009 | 0.125 | -0.254 | 0.235 | 9.41e-01 |
| edge_pheno624 | birth weight | MR Egger | 0.001 | 0.102 | -0.200 | 0.201 | 9.95e-01 |
| edge_pheno624 | birth weight | Weighted median | 0.085 | 0.046 | -0.006 | 0.176 | 6.65e-02 |
| edge_pheno624 | birth weight | Simple mode | 0.077 | 0.110 | -0.140 | 0.294 | 4.87e-01 |
| edge_pheno624 | birth weight | Weighted mode | 0.099 | 0.079 | -0.056 | 0.254 | 2.13e-01 |
| edge_pheno636 | birth weight | MR Egger | -0.011 | 0.100 | -0.208 | 0.186 | 9.09e-01 |
| edge_pheno636 | birth weight | Weighted median | -0.057 | 0.050 | -0.155 | 0.041 | 2.52e-01 |
| edge_pheno636 | birth weight | Simple mode | -0.040 | 0.121 | -0.277 | 0.196 | 7.40e-01 |
| edge_pheno636 | birth weight | Weighted mode | -0.065 | 0.100 | -0.262 | 0.132 | 5.19e-01 |
| edge_pheno639 | birth weight | MR Egger | 0.059 | 0.105 | -0.147 | 0.264 | 5.75e-01 |
| edge_pheno639 | birth weight | Weighted median | 0.013 | 0.049 | -0.083 | 0.108 | 7.93e-01 |
| edge_pheno639 | birth weight | Simple mode | 0.002 | 0.124 | -0.241 | 0.244 | 9.90e-01 |
| edge_pheno639 | birth weight | Weighted mode | 0.008 | 0.091 | -0.170 | 0.186 | 9.27e-01 |
| edge_pheno65 | birth weight | MR Egger | 0.018 | 0.115 | -0.208 | 0.244 | 8.76e-01 |
| edge_pheno65 | birth weight | Weighted median | -0.095 | 0.049 | -0.191 | 0.001 | 5.29e-02 |
| edge_pheno65 | birth weight | Simple mode | -0.096 | 0.129 | -0.350 | 0.157 | 4.57e-01 |
| edge_pheno65 | birth weight | Weighted mode | -0.116 | 0.108 | -0.328 | 0.096 | 2.87e-01 |
| edge_pheno66 | birth weight | MR Egger | -0.023 | 0.111 | -0.242 | 0.195 | 8.35e-01 |
| edge_pheno66 | birth weight | Weighted median | -0.028 | 0.050 | -0.126 | 0.069 | 5.70e-01 |
| edge_pheno66 | birth weight | Simple mode | -0.070 | 0.127 | -0.319 | 0.178 | 5.79e-01 |
| edge_pheno66 | birth weight | Weighted mode | -0.120 | 0.096 | -0.308 | 0.067 | 2.11e-01 |
| edge_pheno674 | birth weight | MR Egger | 0.200 | 0.105 | -0.005 | 0.406 | 5.82e-02 |
| edge_pheno674 | birth weight | Weighted median | 0.143 | 0.047 | 0.050 | 0.236 | 2.50e-03 |
| edge_pheno674 | birth weight | Simple mode | 0.164 | 0.123 | -0.077 | 0.406 | 1.85e-01 |
| edge_pheno674 | birth weight | Weighted mode | 0.153 | 0.095 | -0.033 | 0.339 | 1.09e-01 |
| edge_pheno681 | birth weight | MR Egger | 0.065 | 0.116 | -0.161 | 0.292 | 5.74e-01 |
| edge_pheno681 | birth weight | Weighted median | 0.038 | 0.048 | -0.056 | 0.133 | 4.26e-01 |
| edge_pheno681 | birth weight | Simple mode | 0.040 | 0.124 | -0.203 | 0.282 | 7.49e-01 |
| edge_pheno681 | birth weight | Weighted mode | 0.027 | 0.097 | -0.163 | 0.217 | 7.80e-01 |
| edge_pheno683 | birth weight | MR Egger | -0.115 | 0.113 | -0.335 | 0.106 | 3.11e-01 |
| edge_pheno683 | birth weight | Weighted median | 0.034 | 0.049 | -0.063 | 0.131 | 4.91e-01 |
| edge_pheno683 | birth weight | Simple mode | 0.026 | 0.129 | -0.227 | 0.278 | 8.43e-01 |
| edge_pheno683 | birth weight | Weighted mode | 0.050 | 0.108 | -0.162 | 0.261 | 6.47e-01 |
| edge_pheno695 | birth weight | MR Egger | -0.066 | 0.105 | -0.271 | 0.140 | 5.33e-01 |
| edge_pheno695 | birth weight | Weighted median | 0.062 | 0.049 | -0.034 | 0.157 | 2.05e-01 |
| edge_pheno695 | birth weight | Simple mode | 0.122 | 0.136 | -0.144 | 0.387 | 3.71e-01 |
| edge_pheno695 | birth weight | Weighted mode | 0.105 | 0.116 | -0.123 | 0.332 | 3.69e-01 |
| edge_pheno698 | birth weight | MR Egger | -0.012 | 0.102 | -0.213 | 0.188 | 9.04e-01 |
| edge_pheno698 | birth weight | Weighted median | -0.021 | 0.049 | -0.117 | 0.075 | 6.67e-01 |
| edge_pheno698 | birth weight | Simple mode | -0.039 | 0.130 | -0.294 | 0.216 | 7.66e-01 |
| edge_pheno698 | birth weight | Weighted mode | -0.015 | 0.105 | -0.220 | 0.191 | 8.89e-01 |
| edge_pheno705 | birth weight | MR Egger | 0.032 | 0.112 | -0.188 | 0.252 | 7.75e-01 |
| edge_pheno705 | birth weight | Weighted median | 0.081 | 0.050 | -0.017 | 0.178 | 1.04e-01 |
| edge_pheno705 | birth weight | Simple mode | 0.135 | 0.126 | -0.113 | 0.383 | 2.87e-01 |
| edge_pheno705 | birth weight | Weighted mode | 0.081 | 0.097 | -0.109 | 0.271 | 4.03e-01 |
| edge_pheno716 | birth weight | MR Egger | 0.073 | 0.105 | -0.132 | 0.279 | 4.85e-01 |
| edge_pheno716 | birth weight | Weighted median | -0.012 | 0.048 | -0.106 | 0.082 | 8.02e-01 |
| edge_pheno716 | birth weight | Simple mode | -0.130 | 0.150 | -0.425 | 0.164 | 3.88e-01 |
| edge_pheno716 | birth weight | Weighted mode | -0.119 | 0.141 | -0.395 | 0.158 | 4.02e-01 |
| edge_pheno767 | birth weight | MR Egger | 0.155 | 0.110 | -0.062 | 0.371 | 1.64e-01 |
| edge_pheno767 | birth weight | Weighted median | 0.093 | 0.052 | -0.009 | 0.195 | 7.34e-02 |
| edge_pheno767 | birth weight | Simple mode | 0.135 | 0.150 | -0.159 | 0.430 | 3.69e-01 |
| edge_pheno767 | birth weight | Weighted mode | 0.147 | 0.091 | -0.032 | 0.325 | 1.09e-01 |
| edge_pheno777 | birth weight | MR Egger | -0.066 | 0.110 | -0.282 | 0.149 | 5.47e-01 |
| edge_pheno777 | birth weight | Weighted median | 0.029 | 0.050 | -0.070 | 0.128 | 5.64e-01 |
| edge_pheno777 | birth weight | Simple mode | 0.030 | 0.136 | -0.236 | 0.297 | 8.23e-01 |
| edge_pheno777 | birth weight | Weighted mode | 0.024 | 0.114 | -0.198 | 0.247 | 8.32e-01 |
| edge_pheno789 | birth weight | MR Egger | 0.020 | 0.105 | -0.185 | 0.225 | 8.50e-01 |
| edge_pheno789 | birth weight | Weighted median | 0.064 | 0.045 | -0.025 | 0.152 | 1.58e-01 |
| edge_pheno789 | birth weight | Simple mode | 0.126 | 0.133 | -0.134 | 0.386 | 3.45e-01 |
| edge_pheno789 | birth weight | Weighted mode | 0.147 | 0.116 | -0.080 | 0.374 | 2.06e-01 |
| edge_pheno794 | birth weight | MR Egger | -0.021 | 0.102 | -0.221 | 0.179 | 8.38e-01 |
| edge_pheno794 | birth weight | Weighted median | 0.024 | 0.048 | -0.071 | 0.118 | 6.27e-01 |
| edge_pheno794 | birth weight | Simple mode | 0.049 | 0.137 | -0.220 | 0.318 | 7.21e-01 |
| edge_pheno794 | birth weight | Weighted mode | 0.067 | 0.110 | -0.148 | 0.281 | 5.44e-01 |
| edge_pheno801 | birth weight | MR Egger | 0.025 | 0.103 | -0.177 | 0.226 | 8.11e-01 |
| edge_pheno801 | birth weight | Weighted median | -0.020 | 0.048 | -0.114 | 0.073 | 6.71e-01 |
| edge_pheno801 | birth weight | Simple mode | -0.051 | 0.130 | -0.307 | 0.204 | 6.95e-01 |
| edge_pheno801 | birth weight | Weighted mode | -0.030 | 0.102 | -0.229 | 0.169 | 7.67e-01 |
| edge_pheno810 | birth weight | MR Egger | -0.021 | 0.107 | -0.231 | 0.189 | 8.44e-01 |
| edge_pheno810 | birth weight | Weighted median | 0.023 | 0.049 | -0.073 | 0.118 | 6.40e-01 |
| edge_pheno810 | birth weight | Simple mode | -0.035 | 0.129 | -0.287 | 0.217 | 7.86e-01 |
| edge_pheno810 | birth weight | Weighted mode | -0.012 | 0.100 | -0.208 | 0.184 | 9.04e-01 |
| edge_pheno812 | birth weight | MR Egger | -0.045 | 0.104 | -0.249 | 0.159 | 6.70e-01 |
| edge_pheno812 | birth weight | Weighted median | 0.029 | 0.049 | -0.068 | 0.127 | 5.51e-01 |
| edge_pheno812 | birth weight | Simple mode | -0.101 | 0.140 | -0.374 | 0.173 | 4.71e-01 |
| edge_pheno812 | birth weight | Weighted mode | 0.066 | 0.114 | -0.158 | 0.289 | 5.66e-01 |
| edge_pheno815 | birth weight | MR Egger | 0.232 | 0.124 | -0.011 | 0.474 | 6.32e-02 |
| edge_pheno815 | birth weight | Weighted median | 0.101 | 0.051 | 0.000 | 0.201 | 4.90e-02 |
| edge_pheno815 | birth weight | Simple mode | -0.001 | 0.135 | -0.266 | 0.264 | 9.94e-01 |
| edge_pheno815 | birth weight | Weighted mode | 0.215 | 0.125 | -0.029 | 0.460 | 8.66e-02 |
| edge_pheno816 | birth weight | MR Egger | -0.031 | 0.121 | -0.269 | 0.207 | 7.99e-01 |
| edge_pheno816 | birth weight | Weighted median | -0.040 | 0.048 | -0.134 | 0.055 | 4.12e-01 |
| edge_pheno816 | birth weight | Simple mode | -0.056 | 0.126 | -0.303 | 0.190 | 6.57e-01 |
| edge_pheno816 | birth weight | Weighted mode | -0.063 | 0.081 | -0.221 | 0.095 | 4.38e-01 |
| edge_pheno824 | birth weight | MR Egger | 0.031 | 0.115 | -0.195 | 0.257 | 7.87e-01 |
| edge_pheno824 | birth weight | Weighted median | 0.035 | 0.050 | -0.063 | 0.133 | 4.84e-01 |
| edge_pheno824 | birth weight | Simple mode | 0.080 | 0.113 | -0.142 | 0.303 | 4.79e-01 |
| edge_pheno824 | birth weight | Weighted mode | 0.080 | 0.078 | -0.072 | 0.233 | 3.03e-01 |
| edge_pheno867 | birth weight | MR Egger | 0.130 | 0.109 | -0.084 | 0.345 | 2.35e-01 |
| edge_pheno867 | birth weight | Weighted median | 0.020 | 0.052 | -0.081 | 0.121 | 6.99e-01 |
| edge_pheno867 | birth weight | Simple mode | 0.020 | 0.119 | -0.213 | 0.254 | 8.65e-01 |
| edge_pheno867 | birth weight | Weighted mode | 0.038 | 0.089 | -0.136 | 0.212 | 6.70e-01 |
| edge_pheno869 | birth weight | MR Egger | 0.145 | 0.122 | -0.093 | 0.384 | 2.35e-01 |
| edge_pheno869 | birth weight | Weighted median | 0.000 | 0.050 | -0.097 | 0.098 | 9.93e-01 |
| edge_pheno869 | birth weight | Simple mode | -0.009 | 0.131 | -0.266 | 0.248 | 9.46e-01 |
| edge_pheno869 | birth weight | Weighted mode | -0.061 | 0.115 | -0.286 | 0.164 | 5.94e-01 |
| edge_pheno87 | birth weight | MR Egger | -0.142 | 0.117 | -0.373 | 0.088 | 2.27e-01 |
| edge_pheno87 | birth weight | Weighted median | -0.044 | 0.050 | -0.142 | 0.055 | 3.84e-01 |
| edge_pheno87 | birth weight | Simple mode | 0.008 | 0.144 | -0.275 | 0.290 | 9.57e-01 |
| edge_pheno87 | birth weight | Weighted mode | -0.051 | 0.122 | -0.290 | 0.188 | 6.78e-01 |
| edge_pheno882 | birth weight | MR Egger | 0.057 | 0.105 | -0.150 | 0.264 | 5.89e-01 |
| edge_pheno882 | birth weight | Weighted median | 0.031 | 0.050 | -0.067 | 0.129 | 5.34e-01 |
| edge_pheno882 | birth weight | Simple mode | 0.144 | 0.115 | -0.081 | 0.368 | 2.12e-01 |
| edge_pheno882 | birth weight | Weighted mode | 0.107 | 0.091 | -0.072 | 0.286 | 2.43e-01 |
| edge_pheno899 | birth weight | MR Egger | 0.132 | 0.125 | -0.113 | 0.378 | 2.92e-01 |
| edge_pheno899 | birth weight | Weighted median | 0.006 | 0.051 | -0.095 | 0.107 | 9.04e-01 |
| edge_pheno899 | birth weight | Simple mode | 0.188 | 0.160 | -0.125 | 0.502 | 2.41e-01 |
| edge_pheno899 | birth weight | Weighted mode | 0.181 | 0.140 | -0.094 | 0.456 | 2.00e-01 |
| edge_pheno903 | birth weight | MR Egger | 0.054 | 0.111 | -0.162 | 0.271 | 6.24e-01 |
| edge_pheno903 | birth weight | Weighted median | -0.051 | 0.050 | -0.149 | 0.047 | 3.05e-01 |
| edge_pheno903 | birth weight | Simple mode | -0.021 | 0.136 | -0.287 | 0.246 | 8.80e-01 |
| edge_pheno903 | birth weight | Weighted mode | -0.014 | 0.105 | -0.220 | 0.191 | 8.91e-01 |
| edge_pheno908 | birth weight | MR Egger | -0.004 | 0.107 | -0.214 | 0.206 | 9.69e-01 |
| edge_pheno908 | birth weight | Weighted median | 0.006 | 0.050 | -0.092 | 0.103 | 9.11e-01 |
| edge_pheno908 | birth weight | Simple mode | 0.113 | 0.126 | -0.134 | 0.359 | 3.72e-01 |
| edge_pheno908 | birth weight | Weighted mode | 0.086 | 0.099 | -0.108 | 0.279 | 3.88e-01 |
| edge_pheno918 | birth weight | MR Egger | 0.019 | 0.113 | -0.203 | 0.241 | 8.69e-01 |
| edge_pheno918 | birth weight | Weighted median | -0.053 | 0.047 | -0.145 | 0.038 | 2.51e-01 |
| edge_pheno918 | birth weight | Simple mode | -0.099 | 0.137 | -0.369 | 0.170 | 4.73e-01 |
| edge_pheno918 | birth weight | Weighted mode | -0.110 | 0.108 | -0.321 | 0.100 | 3.07e-01 |
| edge_pheno932 | birth weight | MR Egger | -0.003 | 0.107 | -0.212 | 0.205 | 9.74e-01 |
| edge_pheno932 | birth weight | Weighted median | -0.058 | 0.048 | -0.152 | 0.035 | 2.19e-01 |
| edge_pheno932 | birth weight | Simple mode | -0.047 | 0.131 | -0.305 | 0.210 | 7.19e-01 |
| edge_pheno932 | birth weight | Weighted mode | -0.047 | 0.091 | -0.225 | 0.130 | 6.02e-01 |
| edge_pheno942 | birth weight | MR Egger | -0.127 | 0.114 | -0.351 | 0.097 | 2.70e-01 |
| edge_pheno942 | birth weight | Weighted median | 0.007 | 0.047 | -0.086 | 0.100 | 8.81e-01 |
| edge_pheno942 | birth weight | Simple mode | -0.092 | 0.121 | -0.329 | 0.145 | 4.48e-01 |
| edge_pheno942 | birth weight | Weighted mode | -0.100 | 0.103 | -0.301 | 0.101 | 3.32e-01 |
| edge_pheno956 | birth weight | MR Egger | -0.064 | 0.111 | -0.282 | 0.154 | 5.65e-01 |
| edge_pheno956 | birth weight | Weighted median | -0.062 | 0.048 | -0.156 | 0.033 | 2.03e-01 |
| edge_pheno956 | birth weight | Simple mode | 0.016 | 0.127 | -0.234 | 0.266 | 9.00e-01 |
| edge_pheno956 | birth weight | Weighted mode | -0.022 | 0.098 | -0.214 | 0.170 | 8.22e-01 |
| edge_pheno965 | birth weight | MR Egger | 0.076 | 0.105 | -0.131 | 0.282 | 4.74e-01 |
| edge_pheno965 | birth weight | Weighted median | -0.061 | 0.051 | -0.161 | 0.040 | 2.35e-01 |
| edge_pheno965 | birth weight | Simple mode | -0.260 | 0.133 | -0.522 | 0.001 | 5.31e-02 |
| edge_pheno965 | birth weight | Weighted mode | -0.149 | 0.099 | -0.342 | 0.045 | 1.35e-01 |
| node_pheno1 | birth weight | MR Egger | -0.112 | 0.116 | -0.339 | 0.114 | 3.34e-01 |
| node_pheno1 | birth weight | Weighted median | 0.064 | 0.051 | -0.037 | 0.164 | 2.13e-01 |
| node_pheno1 | birth weight | Simple mode | 0.079 | 0.114 | -0.145 | 0.302 | 4.92e-01 |
| node_pheno1 | birth weight | Weighted mode | 0.073 | 0.090 | -0.103 | 0.249 | 4.16e-01 |
| node_pheno10 | birth weight | MR Egger | -0.039 | 0.105 | -0.245 | 0.166 | 7.08e-01 |
| node_pheno10 | birth weight | Weighted median | -0.056 | 0.048 | -0.151 | 0.038 | 2.44e-01 |
| node_pheno10 | birth weight | Simple mode | 0.042 | 0.130 | -0.214 | 0.297 | 7.51e-01 |
| node_pheno10 | birth weight | Weighted mode | -0.032 | 0.104 | -0.236 | 0.172 | 7.60e-01 |
| node_pheno11 | birth weight | MR Egger | 0.031 | 0.099 | -0.164 | 0.225 | 7.58e-01 |
| node_pheno11 | birth weight | Weighted median | -0.044 | 0.047 | -0.135 | 0.047 | 3.41e-01 |
| node_pheno11 | birth weight | Simple mode | -0.233 | 0.127 | -0.483 | 0.016 | 6.89e-02 |
| node_pheno11 | birth weight | Weighted mode | 0.101 | 0.110 | -0.114 | 0.316 | 3.58e-01 |
| node_pheno12 | birth weight | MR Egger | -0.049 | 0.109 | -0.263 | 0.166 | 6.58e-01 |
| node_pheno12 | birth weight | Weighted median | -0.055 | 0.049 | -0.151 | 0.040 | 2.56e-01 |
| node_pheno12 | birth weight | Simple mode | -0.010 | 0.116 | -0.237 | 0.217 | 9.31e-01 |
| node_pheno12 | birth weight | Weighted mode | -0.036 | 0.088 | -0.208 | 0.136 | 6.84e-01 |
| node_pheno13 | birth weight | MR Egger | -0.098 | 0.111 | -0.315 | 0.120 | 3.80e-01 |
| node_pheno13 | birth weight | Weighted median | -0.013 | 0.049 | -0.108 | 0.082 | 7.90e-01 |
| node_pheno13 | birth weight | Simple mode | 0.006 | 0.120 | -0.229 | 0.242 | 9.57e-01 |
| node_pheno13 | birth weight | Weighted mode | 0.006 | 0.085 | -0.160 | 0.172 | 9.40e-01 |
| node_pheno14 | birth weight | MR Egger | -0.051 | 0.104 | -0.254 | 0.153 | 6.27e-01 |
| node_pheno14 | birth weight | Weighted median | 0.030 | 0.049 | -0.065 | 0.126 | 5.33e-01 |
| node_pheno14 | birth weight | Simple mode | 0.064 | 0.125 | -0.182 | 0.310 | 6.11e-01 |
| node_pheno14 | birth weight | Weighted mode | 0.075 | 0.096 | -0.112 | 0.262 | 4.35e-01 |
| node_pheno15 | birth weight | MR Egger | -0.142 | 0.097 | -0.333 | 0.049 | 1.47e-01 |
| node_pheno15 | birth weight | Weighted median | -0.064 | 0.048 | -0.158 | 0.030 | 1.82e-01 |
| node_pheno15 | birth weight | Simple mode | -0.032 | 0.114 | -0.256 | 0.193 | 7.83e-01 |
| node_pheno15 | birth weight | Weighted mode | -0.068 | 0.079 | -0.222 | 0.087 | 3.91e-01 |
| node_pheno16 | birth weight | MR Egger | -0.075 | 0.096 | -0.264 | 0.114 | 4.38e-01 |
| node_pheno16 | birth weight | Weighted median | -0.042 | 0.047 | -0.134 | 0.050 | 3.68e-01 |
| node_pheno16 | birth weight | Simple mode | -0.052 | 0.135 | -0.316 | 0.213 | 7.02e-01 |
| node_pheno16 | birth weight | Weighted mode | -0.006 | 0.097 | -0.196 | 0.184 | 9.49e-01 |
| node_pheno17 | birth weight | MR Egger | -0.071 | 0.105 | -0.278 | 0.136 | 5.02e-01 |
| node_pheno17 | birth weight | Weighted median | -0.052 | 0.046 | -0.141 | 0.038 | 2.60e-01 |
| node_pheno17 | birth weight | Simple mode | -0.112 | 0.120 | -0.348 | 0.123 | 3.52e-01 |
| node_pheno17 | birth weight | Weighted mode | -0.078 | 0.085 | -0.244 | 0.089 | 3.62e-01 |
| node_pheno18 | birth weight | MR Egger | -0.108 | 0.108 | -0.320 | 0.103 | 3.16e-01 |
| node_pheno18 | birth weight | Weighted median | -0.072 | 0.050 | -0.169 | 0.026 | 1.50e-01 |
| node_pheno18 | birth weight | Simple mode | 0.116 | 0.131 | -0.140 | 0.373 | 3.76e-01 |
| node_pheno18 | birth weight | Weighted mode | -0.080 | 0.098 | -0.271 | 0.112 | 4.16e-01 |
| node_pheno19 | birth weight | MR Egger | 0.064 | 0.097 | -0.125 | 0.254 | 5.06e-01 |
| node_pheno19 | birth weight | Weighted median | -0.052 | 0.048 | -0.146 | 0.043 | 2.85e-01 |
| node_pheno19 | birth weight | Simple mode | -0.065 | 0.122 | -0.304 | 0.174 | 5.94e-01 |
| node_pheno19 | birth weight | Weighted mode | -0.072 | 0.096 | -0.260 | 0.115 | 4.52e-01 |
| node_pheno2 | birth weight | MR Egger | -0.170 | 0.098 | -0.362 | 0.021 | 8.36e-02 |
| node_pheno2 | birth weight | Weighted median | -0.096 | 0.047 | -0.188 | -0.004 | 4.16e-02 |
| node_pheno2 | birth weight | Simple mode | -0.095 | 0.134 | -0.357 | 0.168 | 4.80e-01 |
| node_pheno2 | birth weight | Weighted mode | -0.125 | 0.085 | -0.292 | 0.042 | 1.45e-01 |
| node_pheno20 | birth weight | MR Egger | 0.062 | 0.105 | -0.142 | 0.267 | 5.52e-01 |
| node_pheno20 | birth weight | Weighted median | -0.053 | 0.049 | -0.150 | 0.044 | 2.86e-01 |
| node_pheno20 | birth weight | Simple mode | 0.036 | 0.138 | -0.234 | 0.307 | 7.92e-01 |
| node_pheno20 | birth weight | Weighted mode | 0.043 | 0.087 | -0.128 | 0.213 | 6.26e-01 |
| node_pheno21 | birth weight | MR Egger | -0.212 | 0.103 | -0.414 | -0.010 | 4.19e-02 |
| node_pheno21 | birth weight | Weighted median | -0.034 | 0.047 | -0.127 | 0.059 | 4.72e-01 |
| node_pheno21 | birth weight | Simple mode | -0.070 | 0.113 | -0.292 | 0.151 | 5.36e-01 |
| node_pheno21 | birth weight | Weighted mode | -0.055 | 0.095 | -0.242 | 0.132 | 5.67e-01 |
| node_pheno22 | birth weight | MR Egger | -0.060 | 0.104 | -0.263 | 0.143 | 5.65e-01 |
| node_pheno22 | birth weight | Weighted median | -0.062 | 0.046 | -0.152 | 0.027 | 1.72e-01 |
| node_pheno22 | birth weight | Simple mode | -0.007 | 0.122 | -0.246 | 0.232 | 9.53e-01 |
| node_pheno22 | birth weight | Weighted mode | -0.049 | 0.087 | -0.219 | 0.120 | 5.70e-01 |
| node_pheno23 | birth weight | MR Egger | -0.073 | 0.115 | -0.298 | 0.153 | 5.28e-01 |
| node_pheno23 | birth weight | Weighted median | 0.004 | 0.048 | -0.091 | 0.099 | 9.34e-01 |
| node_pheno23 | birth weight | Simple mode | 0.112 | 0.143 | -0.169 | 0.392 | 4.38e-01 |
| node_pheno23 | birth weight | Weighted mode | 0.104 | 0.116 | -0.124 | 0.332 | 3.72e-01 |
| node_pheno24 | birth weight | MR Egger | -0.051 | 0.107 | -0.260 | 0.159 | 6.38e-01 |
| node_pheno24 | birth weight | Weighted median | -0.027 | 0.047 | -0.119 | 0.065 | 5.63e-01 |
| node_pheno24 | birth weight | Simple mode | -0.023 | 0.116 | -0.249 | 0.204 | 8.44e-01 |
| node_pheno24 | birth weight | Weighted mode | -0.023 | 0.087 | -0.193 | 0.147 | 7.93e-01 |
| node_pheno25 | birth weight | MR Egger | -0.186 | 0.108 | -0.399 | 0.026 | 8.86e-02 |
| node_pheno25 | birth weight | Weighted median | -0.093 | 0.049 | -0.188 | 0.002 | 5.51e-02 |
| node_pheno25 | birth weight | Simple mode | -0.081 | 0.119 | -0.314 | 0.152 | 4.99e-01 |
| node_pheno25 | birth weight | Weighted mode | -0.075 | 0.092 | -0.255 | 0.105 | 4.14e-01 |
| node_pheno26 | birth weight | MR Egger | -0.215 | 0.097 | -0.405 | -0.025 | 2.83e-02 |
| node_pheno26 | birth weight | Weighted median | -0.072 | 0.044 | -0.158 | 0.015 | 1.04e-01 |
| node_pheno26 | birth weight | Simple mode | -0.025 | 0.117 | -0.255 | 0.205 | 8.30e-01 |
| node_pheno26 | birth weight | Weighted mode | -0.047 | 0.098 | -0.238 | 0.145 | 6.34e-01 |
| node_pheno27 | birth weight | MR Egger | -0.070 | 0.110 | -0.285 | 0.145 | 5.26e-01 |
| node_pheno27 | birth weight | Weighted median | -0.019 | 0.049 | -0.116 | 0.078 | 6.95e-01 |
| node_pheno27 | birth weight | Simple mode | 0.151 | 0.139 | -0.122 | 0.423 | 2.81e-01 |
| node_pheno27 | birth weight | Weighted mode | 0.133 | 0.122 | -0.106 | 0.371 | 2.78e-01 |
| node_pheno28 | birth weight | MR Egger | -0.039 | 0.096 | -0.229 | 0.150 | 6.83e-01 |
| node_pheno28 | birth weight | Weighted median | 0.071 | 0.047 | -0.022 | 0.164 | 1.36e-01 |
| node_pheno28 | birth weight | Simple mode | 0.099 | 0.128 | -0.152 | 0.349 | 4.41e-01 |
| node_pheno28 | birth weight | Weighted mode | 0.113 | 0.095 | -0.073 | 0.298 | 2.36e-01 |
| node_pheno29 | birth weight | MR Egger | 0.041 | 0.104 | -0.163 | 0.245 | 6.96e-01 |
| node_pheno29 | birth weight | Weighted median | -0.032 | 0.048 | -0.126 | 0.061 | 4.98e-01 |
| node_pheno29 | birth weight | Simple mode | -0.055 | 0.118 | -0.287 | 0.176 | 6.39e-01 |
| node_pheno29 | birth weight | Weighted mode | -0.061 | 0.089 | -0.236 | 0.114 | 4.95e-01 |
| node_pheno3 | birth weight | MR Egger | -0.175 | 0.120 | -0.411 | 0.061 | 1.49e-01 |
| node_pheno3 | birth weight | Weighted median | -0.004 | 0.051 | -0.104 | 0.097 | 9.43e-01 |
| node_pheno3 | birth weight | Simple mode | 0.098 | 0.127 | -0.150 | 0.347 | 4.40e-01 |
| node_pheno3 | birth weight | Weighted mode | 0.068 | 0.105 | -0.138 | 0.273 | 5.19e-01 |
| node_pheno30 | birth weight | MR Egger | -0.030 | 0.112 | -0.250 | 0.189 | 7.88e-01 |
| node_pheno30 | birth weight | Weighted median | -0.004 | 0.047 | -0.096 | 0.089 | 9.41e-01 |
| node_pheno30 | birth weight | Simple mode | 0.037 | 0.120 | -0.197 | 0.272 | 7.56e-01 |
| node_pheno30 | birth weight | Weighted mode | 0.020 | 0.091 | -0.157 | 0.198 | 8.22e-01 |
| node_pheno31 | birth weight | MR Egger | -0.016 | 0.098 | -0.208 | 0.176 | 8.74e-01 |
| node_pheno31 | birth weight | Weighted median | 0.025 | 0.046 | -0.065 | 0.115 | 5.85e-01 |
| node_pheno31 | birth weight | Simple mode | 0.026 | 0.111 | -0.192 | 0.243 | 8.18e-01 |
| node_pheno31 | birth weight | Weighted mode | 0.080 | 0.093 | -0.101 | 0.262 | 3.88e-01 |
| node_pheno32 | birth weight | MR Egger | -0.108 | 0.103 | -0.309 | 0.094 | 2.97e-01 |
| node_pheno32 | birth weight | Weighted median | -0.019 | 0.048 | -0.113 | 0.075 | 6.92e-01 |
| node_pheno32 | birth weight | Simple mode | -0.013 | 0.126 | -0.259 | 0.233 | 9.18e-01 |
| node_pheno32 | birth weight | Weighted mode | -0.002 | 0.106 | -0.209 | 0.206 | 9.87e-01 |
| node_pheno33 | birth weight | MR Egger | -0.132 | 0.108 | -0.344 | 0.080 | 2.24e-01 |
| node_pheno33 | birth weight | Weighted median | -0.039 | 0.046 | -0.129 | 0.052 | 4.01e-01 |
| node_pheno33 | birth weight | Simple mode | -0.067 | 0.121 | -0.304 | 0.170 | 5.81e-01 |
| node_pheno33 | birth weight | Weighted mode | -0.045 | 0.091 | -0.223 | 0.133 | 6.20e-01 |
| node_pheno34 | birth weight | MR Egger | -0.052 | 0.114 | -0.276 | 0.172 | 6.50e-01 |
| node_pheno34 | birth weight | Weighted median | 0.036 | 0.048 | -0.057 | 0.130 | 4.47e-01 |
| node_pheno34 | birth weight | Simple mode | 0.036 | 0.119 | -0.199 | 0.270 | 7.67e-01 |
| node_pheno34 | birth weight | Weighted mode | 0.006 | 0.091 | -0.173 | 0.184 | 9.51e-01 |
| node_pheno35 | birth weight | MR Egger | 0.027 | 0.093 | -0.156 | 0.211 | 7.69e-01 |
| node_pheno35 | birth weight | Weighted median | -0.017 | 0.046 | -0.108 | 0.073 | 7.06e-01 |
| node_pheno35 | birth weight | Simple mode | 0.020 | 0.109 | -0.194 | 0.233 | 8.57e-01 |
| node_pheno35 | birth weight | Weighted mode | -0.037 | 0.088 | -0.210 | 0.136 | 6.77e-01 |
| node_pheno36 | birth weight | MR Egger | -0.033 | 0.101 | -0.231 | 0.164 | 7.41e-01 |
| node_pheno36 | birth weight | Weighted median | -0.057 | 0.046 | -0.147 | 0.033 | 2.12e-01 |
| node_pheno36 | birth weight | Simple mode | -0.106 | 0.121 | -0.343 | 0.131 | 3.83e-01 |
| node_pheno36 | birth weight | Weighted mode | -0.067 | 0.096 | -0.255 | 0.120 | 4.82e-01 |
| node_pheno37 | birth weight | MR Egger | 0.080 | 0.096 | -0.108 | 0.268 | 4.09e-01 |
| node_pheno37 | birth weight | Weighted median | -0.036 | 0.047 | -0.127 | 0.055 | 4.37e-01 |
| node_pheno37 | birth weight | Simple mode | -0.106 | 0.113 | -0.328 | 0.116 | 3.50e-01 |
| node_pheno37 | birth weight | Weighted mode | -0.053 | 0.091 | -0.232 | 0.126 | 5.59e-01 |
| node_pheno38 | birth weight | MR Egger | -0.127 | 0.101 | -0.325 | 0.070 | 2.09e-01 |
| node_pheno38 | birth weight | Weighted median | -0.075 | 0.045 | -0.163 | 0.014 | 9.75e-02 |
| node_pheno38 | birth weight | Simple mode | -0.076 | 0.113 | -0.298 | 0.146 | 5.04e-01 |
| node_pheno38 | birth weight | Weighted mode | -0.061 | 0.096 | -0.250 | 0.128 | 5.29e-01 |
| node_pheno39 | birth weight | MR Egger | -0.016 | 0.112 | -0.237 | 0.204 | 8.84e-01 |
| node_pheno39 | birth weight | Weighted median | -0.039 | 0.047 | -0.132 | 0.053 | 4.06e-01 |
| node_pheno39 | birth weight | Simple mode | -0.217 | 0.124 | -0.460 | 0.026 | 8.25e-02 |
| node_pheno39 | birth weight | Weighted mode | 0.079 | 0.090 | -0.098 | 0.255 | 3.82e-01 |
| node_pheno4 | birth weight | MR Egger | -0.129 | 0.103 | -0.331 | 0.073 | 2.12e-01 |
| node_pheno4 | birth weight | Weighted median | -0.033 | 0.048 | -0.127 | 0.062 | 4.95e-01 |
| node_pheno4 | birth weight | Simple mode | 0.043 | 0.115 | -0.182 | 0.268 | 7.07e-01 |
| node_pheno4 | birth weight | Weighted mode | -0.009 | 0.092 | -0.188 | 0.171 | 9.26e-01 |
| node_pheno40 | birth weight | MR Egger | 0.103 | 0.106 | -0.105 | 0.310 | 3.34e-01 |
| node_pheno40 | birth weight | Weighted median | -0.088 | 0.045 | -0.176 | 0.000 | 4.93e-02 |
| node_pheno40 | birth weight | Simple mode | -0.138 | 0.130 | -0.392 | 0.116 | 2.90e-01 |
| node_pheno40 | birth weight | Weighted mode | 0.059 | 0.105 | -0.146 | 0.265 | 5.73e-01 |
| node_pheno41 | birth weight | MR Egger | -0.083 | 0.114 | -0.307 | 0.141 | 4.70e-01 |
| node_pheno41 | birth weight | Weighted median | -0.103 | 0.050 | -0.201 | -0.006 | 3.76e-02 |
| node_pheno41 | birth weight | Simple mode | -0.138 | 0.147 | -0.426 | 0.151 | 3.51e-01 |
| node_pheno41 | birth weight | Weighted mode | 0.003 | 0.146 | -0.283 | 0.289 | 9.85e-01 |
| node_pheno42 | birth weight | MR Egger | -0.067 | 0.117 | -0.297 | 0.163 | 5.70e-01 |
| node_pheno42 | birth weight | Weighted median | 0.007 | 0.047 | -0.086 | 0.100 | 8.89e-01 |
| node_pheno42 | birth weight | Simple mode | 0.058 | 0.142 | -0.221 | 0.337 | 6.85e-01 |
| node_pheno42 | birth weight | Weighted mode | 0.070 | 0.113 | -0.151 | 0.292 | 5.36e-01 |
| node_pheno43 | birth weight | MR Egger | -0.113 | 0.108 | -0.325 | 0.099 | 3.00e-01 |
| node_pheno43 | birth weight | Weighted median | -0.056 | 0.048 | -0.150 | 0.038 | 2.46e-01 |
| node_pheno43 | birth weight | Simple mode | -0.273 | 0.146 | -0.559 | 0.014 | 6.43e-02 |
| node_pheno43 | birth weight | Weighted mode | -0.243 | 0.138 | -0.514 | 0.028 | 8.10e-02 |
| node_pheno44 | birth weight | MR Egger | -0.080 | 0.094 | -0.264 | 0.105 | 3.99e-01 |
| node_pheno44 | birth weight | Weighted median | -0.049 | 0.045 | -0.137 | 0.039 | 2.76e-01 |
| node_pheno44 | birth weight | Simple mode | -0.148 | 0.113 | -0.370 | 0.074 | 1.93e-01 |
| node_pheno44 | birth weight | Weighted mode | -0.057 | 0.085 | -0.224 | 0.111 | 5.08e-01 |
| node_pheno45 | birth weight | MR Egger | 0.038 | 0.111 | -0.179 | 0.254 | 7.35e-01 |
| node_pheno45 | birth weight | Weighted median | -0.039 | 0.047 | -0.132 | 0.053 | 4.04e-01 |
| node_pheno45 | birth weight | Simple mode | 0.085 | 0.138 | -0.186 | 0.356 | 5.38e-01 |
| node_pheno45 | birth weight | Weighted mode | 0.021 | 0.144 | -0.261 | 0.303 | 8.84e-01 |
| node_pheno46 | birth weight | MR Egger | -0.043 | 0.126 | -0.291 | 0.204 | 7.33e-01 |
| node_pheno46 | birth weight | Weighted median | -0.017 | 0.051 | -0.116 | 0.083 | 7.42e-01 |
| node_pheno46 | birth weight | Simple mode | -0.320 | 0.165 | -0.643 | 0.004 | 5.49e-02 |
| node_pheno46 | birth weight | Weighted mode | 0.115 | 0.139 | -0.157 | 0.387 | 4.10e-01 |
| node_pheno47 | birth weight | MR Egger | -0.101 | 0.106 | -0.310 | 0.107 | 3.42e-01 |
| node_pheno47 | birth weight | Weighted median | -0.047 | 0.048 | -0.140 | 0.046 | 3.20e-01 |
| node_pheno47 | birth weight | Simple mode | -0.072 | 0.120 | -0.307 | 0.164 | 5.52e-01 |
| node_pheno47 | birth weight | Weighted mode | -0.031 | 0.102 | -0.230 | 0.168 | 7.62e-01 |
| node_pheno48 | birth weight | MR Egger | -0.030 | 0.117 | -0.260 | 0.200 | 7.96e-01 |
| node_pheno48 | birth weight | Weighted median | -0.035 | 0.048 | -0.130 | 0.060 | 4.69e-01 |
| node_pheno48 | birth weight | Simple mode | -0.053 | 0.128 | -0.305 | 0.198 | 6.80e-01 |
| node_pheno48 | birth weight | Weighted mode | -0.035 | 0.094 | -0.219 | 0.149 | 7.12e-01 |
| node_pheno49 | birth weight | MR Egger | -0.080 | 0.109 | -0.293 | 0.134 | 4.66e-01 |
| node_pheno49 | birth weight | Weighted median | -0.019 | 0.046 | -0.110 | 0.071 | 6.75e-01 |
| node_pheno49 | birth weight | Simple mode | -0.026 | 0.121 | -0.264 | 0.213 | 8.34e-01 |
| node_pheno49 | birth weight | Weighted mode | -0.003 | 0.089 | -0.178 | 0.171 | 9.71e-01 |
| node_pheno5 | birth weight | MR Egger | -0.099 | 0.098 | -0.292 | 0.093 | 3.14e-01 |
| node_pheno5 | birth weight | Weighted median | -0.008 | 0.046 | -0.099 | 0.082 | 8.59e-01 |
| node_pheno5 | birth weight | Simple mode | 0.120 | 0.123 | -0.122 | 0.362 | 3.33e-01 |
| node_pheno5 | birth weight | Weighted mode | 0.083 | 0.094 | -0.101 | 0.267 | 3.80e-01 |
| node_pheno50 | birth weight | MR Egger | -0.090 | 0.095 | -0.276 | 0.096 | 3.45e-01 |
| node_pheno50 | birth weight | Weighted median | -0.036 | 0.044 | -0.122 | 0.049 | 4.05e-01 |
| node_pheno50 | birth weight | Simple mode | 0.040 | 0.123 | -0.202 | 0.281 | 7.47e-01 |
| node_pheno50 | birth weight | Weighted mode | 0.008 | 0.088 | -0.165 | 0.180 | 9.29e-01 |
| node_pheno51 | birth weight | MR Egger | 0.025 | 0.107 | -0.184 | 0.234 | 8.16e-01 |
| node_pheno51 | birth weight | Weighted median | -0.004 | 0.049 | -0.099 | 0.091 | 9.34e-01 |
| node_pheno51 | birth weight | Simple mode | 0.008 | 0.131 | -0.248 | 0.265 | 9.49e-01 |
| node_pheno51 | birth weight | Weighted mode | 0.003 | 0.103 | -0.199 | 0.204 | 9.78e-01 |
| node_pheno52 | birth weight | MR Egger | -0.229 | 0.102 | -0.428 | -0.030 | 2.62e-02 |
| node_pheno52 | birth weight | Weighted median | -0.100 | 0.046 | -0.190 | -0.010 | 2.86e-02 |
| node_pheno52 | birth weight | Simple mode | -0.136 | 0.124 | -0.378 | 0.107 | 2.75e-01 |
| node_pheno52 | birth weight | Weighted mode | -0.166 | 0.097 | -0.357 | 0.024 | 8.97e-02 |
| node_pheno53 | birth weight | MR Egger | -0.149 | 0.113 | -0.370 | 0.072 | 1.88e-01 |
| node_pheno53 | birth weight | Weighted median | -0.084 | 0.048 | -0.179 | 0.011 | 8.18e-02 |
| node_pheno53 | birth weight | Simple mode | -0.043 | 0.119 | -0.277 | 0.190 | 7.18e-01 |
| node_pheno53 | birth weight | Weighted mode | -0.096 | 0.088 | -0.268 | 0.076 | 2.76e-01 |
| node_pheno54 | birth weight | MR Egger | -0.235 | 0.102 | -0.434 | -0.036 | 2.21e-02 |
| node_pheno54 | birth weight | Weighted median | -0.033 | 0.048 | -0.127 | 0.061 | 4.90e-01 |
| node_pheno54 | birth weight | Simple mode | 0.122 | 0.126 | -0.125 | 0.369 | 3.34e-01 |
| node_pheno54 | birth weight | Weighted mode | 0.095 | 0.106 | -0.113 | 0.302 | 3.72e-01 |
| node_pheno55 | birth weight | MR Egger | 0.072 | 0.109 | -0.142 | 0.285 | 5.13e-01 |
| node_pheno55 | birth weight | Weighted median | -0.040 | 0.047 | -0.132 | 0.052 | 3.94e-01 |
| node_pheno55 | birth weight | Simple mode | 0.009 | 0.114 | -0.213 | 0.232 | 9.35e-01 |
| node_pheno55 | birth weight | Weighted mode | 0.021 | 0.091 | -0.157 | 0.198 | 8.20e-01 |
| node_pheno56 | birth weight | MR Egger | -0.017 | 0.123 | -0.259 | 0.224 | 8.88e-01 |
| node_pheno56 | birth weight | Weighted median | -0.013 | 0.052 | -0.114 | 0.088 | 8.03e-01 |
| node_pheno56 | birth weight | Simple mode | 0.013 | 0.130 | -0.242 | 0.269 | 9.18e-01 |
| node_pheno56 | birth weight | Weighted mode | 0.027 | 0.111 | -0.191 | 0.246 | 8.07e-01 |
| node_pheno57 | birth weight | MR Egger | -0.129 | 0.097 | -0.319 | 0.061 | 1.87e-01 |
| node_pheno57 | birth weight | Weighted median | -0.009 | 0.048 | -0.102 | 0.084 | 8.54e-01 |
| node_pheno57 | birth weight | Simple mode | 0.036 | 0.116 | -0.192 | 0.263 | 7.60e-01 |
| node_pheno57 | birth weight | Weighted mode | 0.021 | 0.091 | -0.157 | 0.199 | 8.19e-01 |
| node_pheno58 | birth weight | MR Egger | -0.052 | 0.102 | -0.253 | 0.149 | 6.12e-01 |
| node_pheno58 | birth weight | Weighted median | -0.075 | 0.046 | -0.165 | 0.015 | 1.03e-01 |
| node_pheno58 | birth weight | Simple mode | -0.037 | 0.128 | -0.287 | 0.213 | 7.70e-01 |
| node_pheno58 | birth weight | Weighted mode | -0.158 | 0.120 | -0.393 | 0.078 | 1.91e-01 |
| node_pheno59 | birth weight | MR Egger | -0.095 | 0.097 | -0.285 | 0.095 | 3.29e-01 |
| node_pheno59 | birth weight | Weighted median | -0.057 | 0.040 | -0.136 | 0.022 | 1.54e-01 |
| node_pheno59 | birth weight | Simple mode | -0.045 | 0.105 | -0.250 | 0.160 | 6.68e-01 |
| node_pheno59 | birth weight | Weighted mode | -0.040 | 0.075 | -0.188 | 0.108 | 5.99e-01 |
| node_pheno60 | birth weight | MR Egger | -0.062 | 0.115 | -0.287 | 0.162 | 5.87e-01 |
| node_pheno60 | birth weight | Weighted median | -0.056 | 0.046 | -0.146 | 0.035 | 2.27e-01 |
| node_pheno60 | birth weight | Simple mode | 0.048 | 0.122 | -0.192 | 0.287 | 6.96e-01 |
| node_pheno60 | birth weight | Weighted mode | 0.079 | 0.109 | -0.135 | 0.292 | 4.72e-01 |
| node_pheno61 | birth weight | MR Egger | -0.028 | 0.116 | -0.255 | 0.198 | 8.06e-01 |
| node_pheno61 | birth weight | Weighted median | 0.051 | 0.047 | -0.041 | 0.143 | 2.77e-01 |
| node_pheno61 | birth weight | Simple mode | 0.111 | 0.136 | -0.155 | 0.377 | 4.15e-01 |
| node_pheno61 | birth weight | Weighted mode | 0.123 | 0.098 | -0.068 | 0.315 | 2.10e-01 |
| node_pheno62 | birth weight | MR Egger | -0.099 | 0.104 | -0.303 | 0.106 | 3.45e-01 |
| node_pheno62 | birth weight | Weighted median | -0.005 | 0.047 | -0.097 | 0.086 | 9.10e-01 |
| node_pheno62 | birth weight | Simple mode | -0.029 | 0.133 | -0.290 | 0.231 | 8.26e-01 |
| node_pheno62 | birth weight | Weighted mode | -0.024 | 0.098 | -0.215 | 0.167 | 8.07e-01 |
| node_pheno63 | birth weight | MR Egger | -0.022 | 0.100 | -0.218 | 0.174 | 8.26e-01 |
| node_pheno63 | birth weight | Weighted median | -0.002 | 0.047 | -0.093 | 0.090 | 9.70e-01 |
| node_pheno63 | birth weight | Simple mode | 0.041 | 0.129 | -0.211 | 0.294 | 7.48e-01 |
| node_pheno63 | birth weight | Weighted mode | 0.027 | 0.094 | -0.157 | 0.211 | 7.76e-01 |
| node_pheno64 | birth weight | MR Egger | -0.093 | 0.107 | -0.303 | 0.117 | 3.89e-01 |
| node_pheno64 | birth weight | Weighted median | -0.037 | 0.046 | -0.127 | 0.054 | 4.26e-01 |
| node_pheno64 | birth weight | Simple mode | -0.064 | 0.122 | -0.302 | 0.175 | 6.02e-01 |
| node_pheno64 | birth weight | Weighted mode | -0.046 | 0.088 | -0.219 | 0.127 | 6.02e-01 |
| node_pheno65 | birth weight | MR Egger | -0.198 | 0.103 | -0.400 | 0.005 | 5.77e-02 |
| node_pheno65 | birth weight | Weighted median | -0.031 | 0.047 | -0.122 | 0.060 | 5.03e-01 |
| node_pheno65 | birth weight | Simple mode | 0.019 | 0.129 | -0.234 | 0.273 | 8.82e-01 |
| node_pheno65 | birth weight | Weighted mode | -0.030 | 0.106 | -0.237 | 0.178 | 7.79e-01 |
| node_pheno66 | birth weight | MR Egger | 0.023 | 0.105 | -0.182 | 0.229 | 8.25e-01 |
| node_pheno66 | birth weight | Weighted median | -0.032 | 0.048 | -0.126 | 0.062 | 5.05e-01 |
| node_pheno66 | birth weight | Simple mode | -0.056 | 0.127 | -0.306 | 0.193 | 6.59e-01 |
| node_pheno66 | birth weight | Weighted mode | 0.020 | 0.095 | -0.165 | 0.205 | 8.32e-01 |
| node_pheno67 | birth weight | MR Egger | -0.181 | 0.112 | -0.400 | 0.037 | 1.07e-01 |
| node_pheno67 | birth weight | Weighted median | -0.132 | 0.048 | -0.225 | -0.039 | 5.61e-03 |
| node_pheno67 | birth weight | Simple mode | -0.160 | 0.121 | -0.397 | 0.078 | 1.90e-01 |
| node_pheno67 | birth weight | Weighted mode | -0.129 | 0.091 | -0.308 | 0.049 | 1.57e-01 |
| node_pheno68 | birth weight | MR Egger | 0.014 | 0.102 | -0.186 | 0.214 | 8.93e-01 |
| node_pheno68 | birth weight | Weighted median | 0.066 | 0.047 | -0.027 | 0.159 | 1.62e-01 |
| node_pheno68 | birth weight | Simple mode | 0.131 | 0.111 | -0.086 | 0.348 | 2.38e-01 |
| node_pheno68 | birth weight | Weighted mode | 0.106 | 0.079 | -0.049 | 0.260 | 1.83e-01 |
| node_pheno69 | birth weight | MR Egger | -0.121 | 0.120 | -0.355 | 0.114 | 3.15e-01 |
| node_pheno69 | birth weight | Weighted median | -0.079 | 0.047 | -0.172 | 0.014 | 9.49e-02 |
| node_pheno69 | birth weight | Simple mode | -0.047 | 0.124 | -0.291 | 0.197 | 7.05e-01 |
| node_pheno69 | birth weight | Weighted mode | -0.075 | 0.092 | -0.255 | 0.105 | 4.15e-01 |
| node_pheno7 | birth weight | MR Egger | -0.047 | 0.114 | -0.269 | 0.176 | 6.82e-01 |
| node_pheno7 | birth weight | Weighted median | 0.066 | 0.049 | -0.031 | 0.162 | 1.83e-01 |
| node_pheno7 | birth weight | Simple mode | 0.181 | 0.133 | -0.079 | 0.441 | 1.75e-01 |
| node_pheno7 | birth weight | Weighted mode | 0.130 | 0.105 | -0.075 | 0.335 | 2.15e-01 |
| node_pheno70 | birth weight | MR Egger | -0.028 | 0.106 | -0.237 | 0.180 | 7.89e-01 |
| node_pheno70 | birth weight | Weighted median | -0.008 | 0.046 | -0.097 | 0.082 | 8.67e-01 |
| node_pheno70 | birth weight | Simple mode | 0.187 | 0.126 | -0.060 | 0.435 | 1.41e-01 |
| node_pheno70 | birth weight | Weighted mode | -0.010 | 0.092 | -0.190 | 0.170 | 9.15e-01 |
| node_pheno71 | birth weight | MR Egger | -0.005 | 0.100 | -0.202 | 0.192 | 9.57e-01 |
| node_pheno71 | birth weight | Weighted median | 0.013 | 0.044 | -0.074 | 0.100 | 7.71e-01 |
| node_pheno71 | birth weight | Simple mode | 0.043 | 0.114 | -0.180 | 0.265 | 7.09e-01 |
| node_pheno71 | birth weight | Weighted mode | 0.061 | 0.093 | -0.121 | 0.242 | 5.14e-01 |
| node_pheno72 | birth weight | MR Egger | -0.165 | 0.104 | -0.369 | 0.040 | 1.17e-01 |
| node_pheno72 | birth weight | Weighted median | -0.071 | 0.040 | -0.149 | 0.008 | 7.89e-02 |
| node_pheno72 | birth weight | Simple mode | -0.071 | 0.113 | -0.291 | 0.150 | 5.32e-01 |
| node_pheno72 | birth weight | Weighted mode | -0.113 | 0.081 | -0.272 | 0.046 | 1.65e-01 |
| node_pheno73 | birth weight | MR Egger | -0.045 | 0.095 | -0.232 | 0.141 | 6.33e-01 |
| node_pheno73 | birth weight | Weighted median | -0.062 | 0.046 | -0.152 | 0.029 | 1.81e-01 |
| node_pheno73 | birth weight | Simple mode | -0.024 | 0.110 | -0.239 | 0.192 | 8.31e-01 |
| node_pheno73 | birth weight | Weighted mode | -0.080 | 0.092 | -0.260 | 0.100 | 3.87e-01 |
| node_pheno74 | birth weight | MR Egger | -0.211 | 0.108 | -0.423 | 0.002 | 5.43e-02 |
| node_pheno74 | birth weight | Weighted median | 0.006 | 0.049 | -0.090 | 0.102 | 9.05e-01 |
| node_pheno74 | birth weight | Simple mode | 0.103 | 0.137 | -0.166 | 0.371 | 4.56e-01 |
| node_pheno74 | birth weight | Weighted mode | 0.090 | 0.109 | -0.124 | 0.303 | 4.11e-01 |
| node_pheno75 | birth weight | MR Egger | -0.132 | 0.097 | -0.323 | 0.059 | 1.79e-01 |
| node_pheno75 | birth weight | Weighted median | -0.035 | 0.043 | -0.120 | 0.050 | 4.19e-01 |
| node_pheno75 | birth weight | Simple mode | -0.129 | 0.116 | -0.357 | 0.098 | 2.67e-01 |
| node_pheno75 | birth weight | Weighted mode | 0.000 | 0.087 | -0.169 | 0.170 | 9.97e-01 |
| node_pheno76 | birth weight | MR Egger | 0.005 | 0.091 | -0.173 | 0.184 | 9.54e-01 |
| node_pheno76 | birth weight | Weighted median | 0.009 | 0.040 | -0.069 | 0.087 | 8.22e-01 |
| node_pheno76 | birth weight | Simple mode | -0.073 | 0.117 | -0.303 | 0.156 | 5.34e-01 |
| node_pheno76 | birth weight | Weighted mode | -0.069 | 0.111 | -0.285 | 0.148 | 5.36e-01 |
| node_pheno8 | birth weight | MR Egger | 0.033 | 0.095 | -0.154 | 0.220 | 7.32e-01 |
| node_pheno8 | birth weight | Weighted median | -0.005 | 0.047 | -0.097 | 0.087 | 9.13e-01 |
| node_pheno8 | birth weight | Simple mode | 0.043 | 0.103 | -0.159 | 0.245 | 6.75e-01 |
| node_pheno8 | birth weight | Weighted mode | 0.034 | 0.090 | -0.143 | 0.211 | 7.10e-01 |
| node_pheno9 | birth weight | MR Egger | -0.208 | 0.102 | -0.408 | -0.009 | 4.32e-02 |
| node_pheno9 | birth weight | Weighted median | -0.052 | 0.050 | -0.151 | 0.047 | 3.01e-01 |
| node_pheno9 | birth weight | Simple mode | -0.043 | 0.123 | -0.283 | 0.197 | 7.26e-01 |
| node_pheno9 | birth weight | Weighted mode | -0.065 | 0.092 | -0.246 | 0.116 | 4.82e-01 |
| edge_pheno101 | childhood_BMI | MR Egger | -0.025 | 0.100 | -0.222 | 0.172 | 8.06e-01 |
| edge_pheno101 | childhood_BMI | Weighted median | -0.051 | 0.042 | -0.134 | 0.032 | 2.27e-01 |
| edge_pheno101 | childhood_BMI | Simple mode | -0.070 | 0.075 | -0.217 | 0.078 | 3.60e-01 |
| edge_pheno101 | childhood_BMI | Weighted mode | -0.066 | 0.056 | -0.176 | 0.043 | 2.44e-01 |
| edge_pheno1013 | childhood_BMI | MR Egger | 0.059 | 0.090 | -0.118 | 0.236 | 5.18e-01 |
| edge_pheno1013 | childhood_BMI | Weighted median | 0.022 | 0.040 | -0.057 | 0.101 | 5.85e-01 |
| edge_pheno1013 | childhood_BMI | Simple mode | 0.024 | 0.071 | -0.116 | 0.164 | 7.36e-01 |
| edge_pheno1013 | childhood_BMI | Weighted mode | 0.036 | 0.058 | -0.076 | 0.149 | 5.31e-01 |
| edge_pheno102 | childhood_BMI | MR Egger | -0.016 | 0.080 | -0.174 | 0.141 | 8.40e-01 |
| edge_pheno102 | childhood_BMI | Weighted median | -0.062 | 0.041 | -0.143 | 0.019 | 1.31e-01 |
| edge_pheno102 | childhood_BMI | Simple mode | -0.090 | 0.086 | -0.258 | 0.079 | 3.06e-01 |
| edge_pheno102 | childhood_BMI | Weighted mode | -0.078 | 0.067 | -0.209 | 0.053 | 2.50e-01 |
| edge_pheno1020 | childhood_BMI | MR Egger | -0.065 | 0.080 | -0.222 | 0.091 | 4.20e-01 |
| edge_pheno1020 | childhood_BMI | Weighted median | 0.015 | 0.038 | -0.060 | 0.090 | 6.91e-01 |
| edge_pheno1020 | childhood_BMI | Simple mode | -0.045 | 0.080 | -0.201 | 0.112 | 5.80e-01 |
| edge_pheno1020 | childhood_BMI | Weighted mode | 0.001 | 0.064 | -0.124 | 0.126 | 9.91e-01 |
| edge_pheno1022 | childhood_BMI | MR Egger | -0.051 | 0.087 | -0.221 | 0.119 | 5.60e-01 |
| edge_pheno1022 | childhood_BMI | Weighted median | -0.001 | 0.039 | -0.078 | 0.076 | 9.83e-01 |
| edge_pheno1022 | childhood_BMI | Simple mode | 0.082 | 0.082 | -0.079 | 0.243 | 3.25e-01 |
| edge_pheno1022 | childhood_BMI | Weighted mode | 0.035 | 0.075 | -0.111 | 0.181 | 6.41e-01 |
| edge_pheno103 | childhood_BMI | MR Egger | -0.088 | 0.078 | -0.241 | 0.065 | 2.66e-01 |
| edge_pheno103 | childhood_BMI | Weighted median | -0.078 | 0.037 | -0.150 | -0.006 | 3.35e-02 |
| edge_pheno103 | childhood_BMI | Simple mode | -0.046 | 0.073 | -0.188 | 0.097 | 5.32e-01 |
| edge_pheno103 | childhood_BMI | Weighted mode | -0.068 | 0.052 | -0.169 | 0.033 | 1.98e-01 |
| edge_pheno1041 | childhood_BMI | MR Egger | 0.015 | 0.079 | -0.141 | 0.170 | 8.53e-01 |
| edge_pheno1041 | childhood_BMI | Weighted median | -0.002 | 0.039 | -0.078 | 0.074 | 9.57e-01 |
| edge_pheno1041 | childhood_BMI | Simple mode | -0.026 | 0.071 | -0.166 | 0.113 | 7.16e-01 |
| edge_pheno1041 | childhood_BMI | Weighted mode | -0.030 | 0.058 | -0.143 | 0.083 | 6.03e-01 |
| edge_pheno1059 | childhood_BMI | MR Egger | 0.087 | 0.079 | -0.069 | 0.243 | 2.81e-01 |
| edge_pheno1059 | childhood_BMI | Weighted median | -0.020 | 0.040 | -0.099 | 0.059 | 6.19e-01 |
| edge_pheno1059 | childhood_BMI | Simple mode | 0.049 | 0.076 | -0.101 | 0.198 | 5.28e-01 |
| edge_pheno1059 | childhood_BMI | Weighted mode | 0.044 | 0.062 | -0.077 | 0.165 | 4.80e-01 |
| edge_pheno1122 | childhood_BMI | MR Egger | -0.045 | 0.102 | -0.244 | 0.154 | 6.62e-01 |
| edge_pheno1122 | childhood_BMI | Weighted median | -0.016 | 0.042 | -0.097 | 0.065 | 7.01e-01 |
| edge_pheno1122 | childhood_BMI | Simple mode | -0.010 | 0.082 | -0.171 | 0.151 | 8.99e-01 |
| edge_pheno1122 | childhood_BMI | Weighted mode | 0.008 | 0.061 | -0.111 | 0.128 | 8.94e-01 |
| edge_pheno1126 | childhood_BMI | MR Egger | -0.112 | 0.081 | -0.270 | 0.047 | 1.76e-01 |
| edge_pheno1126 | childhood_BMI | Weighted median | -0.088 | 0.039 | -0.165 | -0.011 | 2.53e-02 |
| edge_pheno1126 | childhood_BMI | Simple mode | -0.110 | 0.085 | -0.277 | 0.057 | 2.05e-01 |
| edge_pheno1126 | childhood_BMI | Weighted mode | -0.127 | 0.074 | -0.271 | 0.018 | 9.51e-02 |
| edge_pheno1134 | childhood_BMI | MR Egger | -0.039 | 0.081 | -0.197 | 0.119 | 6.34e-01 |
| edge_pheno1134 | childhood_BMI | Weighted median | -0.021 | 0.039 | -0.099 | 0.056 | 5.89e-01 |
| edge_pheno1134 | childhood_BMI | Simple mode | 0.033 | 0.073 | -0.109 | 0.175 | 6.51e-01 |
| edge_pheno1134 | childhood_BMI | Weighted mode | -0.013 | 0.067 | -0.145 | 0.119 | 8.47e-01 |
| edge_pheno1137 | childhood_BMI | MR Egger | -0.083 | 0.079 | -0.238 | 0.072 | 3.01e-01 |
| edge_pheno1137 | childhood_BMI | Weighted median | -0.043 | 0.039 | -0.119 | 0.033 | 2.69e-01 |
| edge_pheno1137 | childhood_BMI | Simple mode | -0.038 | 0.076 | -0.186 | 0.111 | 6.22e-01 |
| edge_pheno1137 | childhood_BMI | Weighted mode | -0.051 | 0.070 | -0.189 | 0.087 | 4.73e-01 |
| edge_pheno1141 | childhood_BMI | MR Egger | -0.073 | 0.079 | -0.227 | 0.082 | 3.63e-01 |
| edge_pheno1141 | childhood_BMI | Weighted median | -0.064 | 0.040 | -0.141 | 0.014 | 1.07e-01 |
| edge_pheno1141 | childhood_BMI | Simple mode | -0.052 | 0.067 | -0.182 | 0.078 | 4.40e-01 |
| edge_pheno1141 | childhood_BMI | Weighted mode | -0.058 | 0.054 | -0.164 | 0.048 | 2.92e-01 |
| edge_pheno1142 | childhood_BMI | MR Egger | 0.028 | 0.080 | -0.128 | 0.184 | 7.25e-01 |
| edge_pheno1142 | childhood_BMI | Weighted median | -0.050 | 0.041 | -0.131 | 0.030 | 2.20e-01 |
| edge_pheno1142 | childhood_BMI | Simple mode | -0.030 | 0.087 | -0.200 | 0.139 | 7.28e-01 |
| edge_pheno1142 | childhood_BMI | Weighted mode | -0.089 | 0.072 | -0.230 | 0.052 | 2.22e-01 |
| edge_pheno1161 | childhood_BMI | MR Egger | 0.053 | 0.096 | -0.136 | 0.242 | 5.84e-01 |
| edge_pheno1161 | childhood_BMI | Weighted median | 0.016 | 0.039 | -0.060 | 0.092 | 6.76e-01 |
| edge_pheno1161 | childhood_BMI | Simple mode | 0.036 | 0.071 | -0.104 | 0.176 | 6.15e-01 |
| edge_pheno1161 | childhood_BMI | Weighted mode | 0.027 | 0.056 | -0.083 | 0.137 | 6.39e-01 |
| edge_pheno1167 | childhood_BMI | MR Egger | -0.163 | 0.088 | -0.336 | 0.011 | 7.53e-02 |
| edge_pheno1167 | childhood_BMI | Weighted median | -0.057 | 0.042 | -0.140 | 0.026 | 1.80e-01 |
| edge_pheno1167 | childhood_BMI | Simple mode | -0.090 | 0.078 | -0.243 | 0.063 | 2.58e-01 |
| edge_pheno1167 | childhood_BMI | Weighted mode | -0.075 | 0.072 | -0.215 | 0.066 | 3.05e-01 |
| edge_pheno1171 | childhood_BMI | MR Egger | 0.068 | 0.078 | -0.085 | 0.221 | 3.88e-01 |
| edge_pheno1171 | childhood_BMI | Weighted median | 0.031 | 0.038 | -0.044 | 0.106 | 4.18e-01 |
| edge_pheno1171 | childhood_BMI | Simple mode | 0.049 | 0.064 | -0.076 | 0.174 | 4.46e-01 |
| edge_pheno1171 | childhood_BMI | Weighted mode | 0.043 | 0.056 | -0.067 | 0.153 | 4.48e-01 |
| edge_pheno1175 | childhood_BMI | MR Egger | 0.010 | 0.086 | -0.158 | 0.178 | 9.05e-01 |
| edge_pheno1175 | childhood_BMI | Weighted median | -0.001 | 0.038 | -0.077 | 0.074 | 9.70e-01 |
| edge_pheno1175 | childhood_BMI | Simple mode | -0.029 | 0.070 | -0.166 | 0.107 | 6.77e-01 |
| edge_pheno1175 | childhood_BMI | Weighted mode | 0.002 | 0.055 | -0.105 | 0.109 | 9.66e-01 |
| edge_pheno1183 | childhood_BMI | MR Egger | -0.107 | 0.081 | -0.266 | 0.051 | 1.94e-01 |
| edge_pheno1183 | childhood_BMI | Weighted median | 0.011 | 0.041 | -0.069 | 0.091 | 7.85e-01 |
| edge_pheno1183 | childhood_BMI | Simple mode | 0.051 | 0.085 | -0.117 | 0.218 | 5.56e-01 |
| edge_pheno1183 | childhood_BMI | Weighted mode | 0.006 | 0.069 | -0.129 | 0.142 | 9.28e-01 |
| edge_pheno1184 | childhood_BMI | MR Egger | -0.015 | 0.097 | -0.206 | 0.176 | 8.78e-01 |
| edge_pheno1184 | childhood_BMI | Weighted median | 0.000 | 0.040 | -0.078 | 0.078 | 9.98e-01 |
| edge_pheno1184 | childhood_BMI | Simple mode | 0.022 | 0.064 | -0.103 | 0.147 | 7.32e-01 |
| edge_pheno1184 | childhood_BMI | Weighted mode | 0.016 | 0.054 | -0.089 | 0.121 | 7.66e-01 |
| edge_pheno1189 | childhood_BMI | MR Egger | -0.046 | 0.097 | -0.236 | 0.143 | 6.36e-01 |
| edge_pheno1189 | childhood_BMI | Weighted median | -0.025 | 0.039 | -0.101 | 0.051 | 5.21e-01 |
| edge_pheno1189 | childhood_BMI | Simple mode | -0.001 | 0.077 | -0.153 | 0.150 | 9.87e-01 |
| edge_pheno1189 | childhood_BMI | Weighted mode | -0.026 | 0.064 | -0.151 | 0.099 | 6.84e-01 |
| edge_pheno1205 | childhood_BMI | MR Egger | 0.052 | 0.078 | -0.101 | 0.204 | 5.10e-01 |
| edge_pheno1205 | childhood_BMI | Weighted median | 0.017 | 0.039 | -0.060 | 0.094 | 6.63e-01 |
| edge_pheno1205 | childhood_BMI | Simple mode | 0.018 | 0.081 | -0.140 | 0.176 | 8.26e-01 |
| edge_pheno1205 | childhood_BMI | Weighted mode | 0.025 | 0.073 | -0.119 | 0.168 | 7.39e-01 |
| edge_pheno1211 | childhood_BMI | MR Egger | -0.001 | 0.083 | -0.164 | 0.161 | 9.89e-01 |
| edge_pheno1211 | childhood_BMI | Weighted median | 0.002 | 0.042 | -0.079 | 0.084 | 9.59e-01 |
| edge_pheno1211 | childhood_BMI | Simple mode | 0.035 | 0.076 | -0.113 | 0.184 | 6.43e-01 |
| edge_pheno1211 | childhood_BMI | Weighted mode | 0.040 | 0.061 | -0.080 | 0.160 | 5.18e-01 |
| edge_pheno1221 | childhood_BMI | MR Egger | 0.083 | 0.078 | -0.070 | 0.237 | 2.96e-01 |
| edge_pheno1221 | childhood_BMI | Weighted median | -0.003 | 0.039 | -0.080 | 0.075 | 9.43e-01 |
| edge_pheno1221 | childhood_BMI | Simple mode | -0.004 | 0.072 | -0.145 | 0.136 | 9.51e-01 |
| edge_pheno1221 | childhood_BMI | Weighted mode | -0.004 | 0.066 | -0.133 | 0.124 | 9.46e-01 |
| edge_pheno1225 | childhood_BMI | MR Egger | -0.121 | 0.092 | -0.302 | 0.059 | 1.98e-01 |
| edge_pheno1225 | childhood_BMI | Weighted median | -0.016 | 0.039 | -0.093 | 0.061 | 6.80e-01 |
| edge_pheno1225 | childhood_BMI | Simple mode | -0.002 | 0.071 | -0.142 | 0.138 | 9.73e-01 |
| edge_pheno1225 | childhood_BMI | Weighted mode | -0.010 | 0.049 | -0.107 | 0.086 | 8.34e-01 |
| edge_pheno1250 | childhood_BMI | MR Egger | -0.102 | 0.090 | -0.277 | 0.074 | 2.63e-01 |
| edge_pheno1250 | childhood_BMI | Weighted median | -0.034 | 0.038 | -0.109 | 0.041 | 3.70e-01 |
| edge_pheno1250 | childhood_BMI | Simple mode | -0.031 | 0.065 | -0.158 | 0.096 | 6.38e-01 |
| edge_pheno1250 | childhood_BMI | Weighted mode | -0.017 | 0.055 | -0.125 | 0.091 | 7.56e-01 |
| edge_pheno1256 | childhood_BMI | MR Egger | -0.077 | 0.090 | -0.253 | 0.099 | 3.96e-01 |
| edge_pheno1256 | childhood_BMI | Weighted median | -0.096 | 0.040 | -0.174 | -0.018 | 1.57e-02 |
| edge_pheno1256 | childhood_BMI | Simple mode | -0.104 | 0.075 | -0.250 | 0.042 | 1.73e-01 |
| edge_pheno1256 | childhood_BMI | Weighted mode | -0.109 | 0.057 | -0.222 | 0.003 | 6.50e-02 |
| edge_pheno1257 | childhood_BMI | MR Egger | -0.102 | 0.100 | -0.298 | 0.094 | 3.15e-01 |
| edge_pheno1257 | childhood_BMI | Weighted median | 0.010 | 0.043 | -0.074 | 0.094 | 8.22e-01 |
| edge_pheno1257 | childhood_BMI | Simple mode | 0.014 | 0.095 | -0.172 | 0.200 | 8.84e-01 |
| edge_pheno1257 | childhood_BMI | Weighted mode | 0.005 | 0.081 | -0.154 | 0.165 | 9.51e-01 |
| edge_pheno1269 | childhood_BMI | MR Egger | -0.138 | 0.085 | -0.305 | 0.030 | 1.16e-01 |
| edge_pheno1269 | childhood_BMI | Weighted median | -0.054 | 0.040 | -0.131 | 0.024 | 1.76e-01 |
| edge_pheno1269 | childhood_BMI | Simple mode | -0.029 | 0.075 | -0.176 | 0.118 | 7.03e-01 |
| edge_pheno1269 | childhood_BMI | Weighted mode | -0.062 | 0.061 | -0.181 | 0.057 | 3.14e-01 |
| edge_pheno1270 | childhood_BMI | MR Egger | -0.085 | 0.094 | -0.270 | 0.099 | 3.72e-01 |
| edge_pheno1270 | childhood_BMI | Weighted median | -0.012 | 0.039 | -0.088 | 0.063 | 7.50e-01 |
| edge_pheno1270 | childhood_BMI | Simple mode | -0.025 | 0.065 | -0.152 | 0.101 | 6.96e-01 |
| edge_pheno1270 | childhood_BMI | Weighted mode | -0.055 | 0.058 | -0.168 | 0.058 | 3.49e-01 |
| edge_pheno1273 | childhood_BMI | MR Egger | 0.132 | 0.089 | -0.042 | 0.306 | 1.46e-01 |
| edge_pheno1273 | childhood_BMI | Weighted median | 0.049 | 0.041 | -0.031 | 0.129 | 2.32e-01 |
| edge_pheno1273 | childhood_BMI | Simple mode | 0.158 | 0.078 | 0.005 | 0.311 | 5.09e-02 |
| edge_pheno1273 | childhood_BMI | Weighted mode | 0.145 | 0.075 | -0.003 | 0.292 | 6.30e-02 |
| edge_pheno1276 | childhood_BMI | MR Egger | -0.001 | 0.077 | -0.151 | 0.149 | 9.90e-01 |
| edge_pheno1276 | childhood_BMI | Weighted median | -0.032 | 0.037 | -0.103 | 0.040 | 3.88e-01 |
| edge_pheno1276 | childhood_BMI | Simple mode | -0.047 | 0.065 | -0.174 | 0.080 | 4.71e-01 |
| edge_pheno1276 | childhood_BMI | Weighted mode | -0.043 | 0.061 | -0.162 | 0.077 | 4.90e-01 |
| edge_pheno1293 | childhood_BMI | MR Egger | -0.127 | 0.087 | -0.297 | 0.044 | 1.54e-01 |
| edge_pheno1293 | childhood_BMI | Weighted median | 0.022 | 0.039 | -0.054 | 0.098 | 5.73e-01 |
| edge_pheno1293 | childhood_BMI | Simple mode | 0.029 | 0.070 | -0.107 | 0.166 | 6.76e-01 |
| edge_pheno1293 | childhood_BMI | Weighted mode | 0.026 | 0.055 | -0.082 | 0.135 | 6.36e-01 |
| edge_pheno1296 | childhood_BMI | MR Egger | -0.040 | 0.088 | -0.212 | 0.133 | 6.57e-01 |
| edge_pheno1296 | childhood_BMI | Weighted median | -0.038 | 0.040 | -0.118 | 0.041 | 3.40e-01 |
| edge_pheno1296 | childhood_BMI | Simple mode | -0.008 | 0.084 | -0.172 | 0.157 | 9.29e-01 |
| edge_pheno1296 | childhood_BMI | Weighted mode | -0.016 | 0.071 | -0.155 | 0.124 | 8.28e-01 |
| edge_pheno1300 | childhood_BMI | MR Egger | 0.041 | 0.082 | -0.120 | 0.202 | 6.19e-01 |
| edge_pheno1300 | childhood_BMI | Weighted median | 0.015 | 0.040 | -0.064 | 0.094 | 7.13e-01 |
| edge_pheno1300 | childhood_BMI | Simple mode | -0.005 | 0.066 | -0.134 | 0.124 | 9.40e-01 |
| edge_pheno1300 | childhood_BMI | Weighted mode | 0.018 | 0.059 | -0.097 | 0.133 | 7.59e-01 |
| edge_pheno1301 | childhood_BMI | MR Egger | -0.017 | 0.097 | -0.208 | 0.173 | 8.58e-01 |
| edge_pheno1301 | childhood_BMI | Weighted median | 0.001 | 0.039 | -0.075 | 0.078 | 9.75e-01 |
| edge_pheno1301 | childhood_BMI | Simple mode | 0.054 | 0.078 | -0.098 | 0.206 | 4.92e-01 |
| edge_pheno1301 | childhood_BMI | Weighted mode | 0.025 | 0.063 | -0.099 | 0.148 | 6.98e-01 |
| edge_pheno1302 | childhood_BMI | MR Egger | 0.086 | 0.079 | -0.068 | 0.240 | 2.84e-01 |
| edge_pheno1302 | childhood_BMI | Weighted median | 0.017 | 0.040 | -0.061 | 0.096 | 6.64e-01 |
| edge_pheno1302 | childhood_BMI | Simple mode | 0.043 | 0.076 | -0.107 | 0.192 | 5.80e-01 |
| edge_pheno1302 | childhood_BMI | Weighted mode | 0.041 | 0.061 | -0.079 | 0.160 | 5.10e-01 |
| edge_pheno1309 | childhood_BMI | MR Egger | -0.087 | 0.080 | -0.243 | 0.069 | 2.84e-01 |
| edge_pheno1309 | childhood_BMI | Weighted median | -0.005 | 0.041 | -0.086 | 0.076 | 9.07e-01 |
| edge_pheno1309 | childhood_BMI | Simple mode | -0.023 | 0.084 | -0.188 | 0.142 | 7.85e-01 |
| edge_pheno1309 | childhood_BMI | Weighted mode | -0.016 | 0.080 | -0.173 | 0.140 | 8.41e-01 |
| edge_pheno1311 | childhood_BMI | MR Egger | 0.023 | 0.091 | -0.156 | 0.201 | 8.06e-01 |
| edge_pheno1311 | childhood_BMI | Weighted median | -0.001 | 0.044 | -0.088 | 0.086 | 9.81e-01 |
| edge_pheno1311 | childhood_BMI | Simple mode | 0.000 | 0.084 | -0.166 | 0.165 | 9.97e-01 |
| edge_pheno1311 | childhood_BMI | Weighted mode | 0.004 | 0.066 | -0.126 | 0.134 | 9.55e-01 |
| edge_pheno1317 | childhood_BMI | MR Egger | -0.078 | 0.100 | -0.275 | 0.119 | 4.44e-01 |
| edge_pheno1317 | childhood_BMI | Weighted median | -0.079 | 0.047 | -0.172 | 0.014 | 9.46e-02 |
| edge_pheno1317 | childhood_BMI | Simple mode | -0.119 | 0.093 | -0.301 | 0.064 | 2.14e-01 |
| edge_pheno1317 | childhood_BMI | Weighted mode | -0.127 | 0.078 | -0.279 | 0.026 | 1.16e-01 |
| edge_pheno1319 | childhood_BMI | MR Egger | 0.276 | 0.094 | 0.092 | 0.461 | 6.07e-03 |
| edge_pheno1319 | childhood_BMI | Weighted median | 0.116 | 0.042 | 0.034 | 0.199 | 5.67e-03 |
| edge_pheno1319 | childhood_BMI | Simple mode | 0.113 | 0.092 | -0.067 | 0.293 | 2.29e-01 |
| edge_pheno1319 | childhood_BMI | Weighted mode | 0.159 | 0.069 | 0.024 | 0.294 | 2.71e-02 |
| edge_pheno132 | childhood_BMI | MR Egger | -0.016 | 0.079 | -0.172 | 0.140 | 8.39e-01 |
| edge_pheno132 | childhood_BMI | Weighted median | 0.003 | 0.039 | -0.073 | 0.080 | 9.33e-01 |
| edge_pheno132 | childhood_BMI | Simple mode | -0.013 | 0.080 | -0.170 | 0.144 | 8.75e-01 |
| edge_pheno132 | childhood_BMI | Weighted mode | -0.021 | 0.066 | -0.151 | 0.108 | 7.51e-01 |
| edge_pheno1322 | childhood_BMI | MR Egger | 0.122 | 0.079 | -0.033 | 0.278 | 1.33e-01 |
| edge_pheno1322 | childhood_BMI | Weighted median | 0.005 | 0.038 | -0.070 | 0.080 | 8.95e-01 |
| edge_pheno1322 | childhood_BMI | Simple mode | -0.016 | 0.067 | -0.147 | 0.115 | 8.09e-01 |
| edge_pheno1322 | childhood_BMI | Weighted mode | 0.006 | 0.053 | -0.098 | 0.110 | 9.12e-01 |
| edge_pheno1325 | childhood_BMI | MR Egger | -0.005 | 0.080 | -0.161 | 0.151 | 9.51e-01 |
| edge_pheno1325 | childhood_BMI | Weighted median | 0.000 | 0.039 | -0.076 | 0.076 | 9.94e-01 |
| edge_pheno1325 | childhood_BMI | Simple mode | -0.018 | 0.082 | -0.178 | 0.143 | 8.30e-01 |
| edge_pheno1325 | childhood_BMI | Weighted mode | 0.010 | 0.067 | -0.121 | 0.141 | 8.83e-01 |
| edge_pheno1328 | childhood_BMI | MR Egger | 0.004 | 0.080 | -0.152 | 0.160 | 9.56e-01 |
| edge_pheno1328 | childhood_BMI | Weighted median | 0.070 | 0.039 | -0.006 | 0.147 | 7.20e-02 |
| edge_pheno1328 | childhood_BMI | Simple mode | 0.076 | 0.080 | -0.081 | 0.234 | 3.48e-01 |
| edge_pheno1328 | childhood_BMI | Weighted mode | 0.084 | 0.057 | -0.027 | 0.195 | 1.48e-01 |
| edge_pheno135 | childhood_BMI | MR Egger | 0.056 | 0.079 | -0.099 | 0.211 | 4.85e-01 |
| edge_pheno135 | childhood_BMI | Weighted median | -0.003 | 0.041 | -0.083 | 0.077 | 9.47e-01 |
| edge_pheno135 | childhood_BMI | Simple mode | 0.008 | 0.077 | -0.142 | 0.158 | 9.20e-01 |
| edge_pheno135 | childhood_BMI | Weighted mode | 0.005 | 0.064 | -0.120 | 0.131 | 9.33e-01 |
| edge_pheno1359 | childhood_BMI | MR Egger | 0.053 | 0.079 | -0.102 | 0.207 | 5.07e-01 |
| edge_pheno1359 | childhood_BMI | Weighted median | 0.013 | 0.040 | -0.065 | 0.091 | 7.44e-01 |
| edge_pheno1359 | childhood_BMI | Simple mode | 0.013 | 0.080 | -0.143 | 0.170 | 8.69e-01 |
| edge_pheno1359 | childhood_BMI | Weighted mode | 0.033 | 0.069 | -0.103 | 0.169 | 6.37e-01 |
| edge_pheno1382 | childhood_BMI | MR Egger | 0.061 | 0.078 | -0.092 | 0.215 | 4.37e-01 |
| edge_pheno1382 | childhood_BMI | Weighted median | 0.076 | 0.038 | 0.002 | 0.150 | 4.28e-02 |
| edge_pheno1382 | childhood_BMI | Simple mode | 0.102 | 0.072 | -0.039 | 0.242 | 1.66e-01 |
| edge_pheno1382 | childhood_BMI | Weighted mode | 0.093 | 0.066 | -0.036 | 0.221 | 1.67e-01 |
| edge_pheno146 | childhood_BMI | MR Egger | -0.073 | 0.094 | -0.256 | 0.110 | 4.40e-01 |
| edge_pheno146 | childhood_BMI | Weighted median | 0.006 | 0.040 | -0.072 | 0.085 | 8.73e-01 |
| edge_pheno146 | childhood_BMI | Simple mode | 0.024 | 0.081 | -0.135 | 0.183 | 7.68e-01 |
| edge_pheno146 | childhood_BMI | Weighted mode | -0.004 | 0.061 | -0.124 | 0.115 | 9.45e-01 |
| edge_pheno147 | childhood_BMI | MR Egger | -0.094 | 0.080 | -0.250 | 0.063 | 2.49e-01 |
| edge_pheno147 | childhood_BMI | Weighted median | -0.071 | 0.038 | -0.145 | 0.004 | 6.29e-02 |
| edge_pheno147 | childhood_BMI | Simple mode | -0.047 | 0.070 | -0.185 | 0.091 | 5.10e-01 |
| edge_pheno147 | childhood_BMI | Weighted mode | -0.049 | 0.061 | -0.168 | 0.070 | 4.25e-01 |
| edge_pheno151 | childhood_BMI | MR Egger | 0.038 | 0.080 | -0.120 | 0.195 | 6.42e-01 |
| edge_pheno151 | childhood_BMI | Weighted median | 0.055 | 0.040 | -0.024 | 0.133 | 1.73e-01 |
| edge_pheno151 | childhood_BMI | Simple mode | 0.042 | 0.080 | -0.116 | 0.199 | 6.08e-01 |
| edge_pheno151 | childhood_BMI | Weighted mode | 0.050 | 0.066 | -0.079 | 0.179 | 4.51e-01 |
| edge_pheno1696 | childhood_BMI | MR Egger | 0.082 | 0.081 | -0.076 | 0.241 | 3.17e-01 |
| edge_pheno1696 | childhood_BMI | Weighted median | 0.040 | 0.040 | -0.039 | 0.119 | 3.17e-01 |
| edge_pheno1696 | childhood_BMI | Simple mode | 0.035 | 0.070 | -0.102 | 0.173 | 6.16e-01 |
| edge_pheno1696 | childhood_BMI | Weighted mode | 0.041 | 0.057 | -0.070 | 0.152 | 4.77e-01 |
| edge_pheno1697 | childhood_BMI | MR Egger | -0.004 | 0.077 | -0.156 | 0.147 | 9.56e-01 |
| edge_pheno1697 | childhood_BMI | Weighted median | -0.012 | 0.037 | -0.084 | 0.060 | 7.40e-01 |
| edge_pheno1697 | childhood_BMI | Simple mode | -0.014 | 0.062 | -0.136 | 0.108 | 8.26e-01 |
| edge_pheno1697 | childhood_BMI | Weighted mode | -0.018 | 0.052 | -0.121 | 0.084 | 7.30e-01 |
| edge_pheno1698 | childhood_BMI | MR Egger | -0.156 | 0.079 | -0.311 | -0.001 | 5.67e-02 |
| edge_pheno1698 | childhood_BMI | Weighted median | -0.002 | 0.038 | -0.076 | 0.073 | 9.66e-01 |
| edge_pheno1698 | childhood_BMI | Simple mode | 0.022 | 0.074 | -0.122 | 0.167 | 7.64e-01 |
| edge_pheno1698 | childhood_BMI | Weighted mode | -0.010 | 0.061 | -0.129 | 0.109 | 8.70e-01 |
| edge_pheno1699 | childhood_BMI | MR Egger | 0.004 | 0.086 | -0.165 | 0.174 | 9.60e-01 |
| edge_pheno1699 | childhood_BMI | Weighted median | 0.018 | 0.038 | -0.057 | 0.093 | 6.42e-01 |
| edge_pheno1699 | childhood_BMI | Simple mode | 0.047 | 0.071 | -0.092 | 0.186 | 5.11e-01 |
| edge_pheno1699 | childhood_BMI | Weighted mode | 0.012 | 0.053 | -0.092 | 0.117 | 8.18e-01 |
| edge_pheno1701 | childhood_BMI | MR Egger | -0.031 | 0.101 | -0.229 | 0.166 | 7.57e-01 |
| edge_pheno1701 | childhood_BMI | Weighted median | -0.026 | 0.039 | -0.103 | 0.051 | 5.11e-01 |
| edge_pheno1701 | childhood_BMI | Simple mode | 0.062 | 0.083 | -0.102 | 0.225 | 4.64e-01 |
| edge_pheno1701 | childhood_BMI | Weighted mode | -0.023 | 0.060 | -0.140 | 0.095 | 7.10e-01 |
| edge_pheno249 | childhood_BMI | MR Egger | 0.093 | 0.084 | -0.072 | 0.259 | 2.75e-01 |
| edge_pheno249 | childhood_BMI | Weighted median | 0.019 | 0.039 | -0.058 | 0.096 | 6.33e-01 |
| edge_pheno249 | childhood_BMI | Simple mode | -0.031 | 0.081 | -0.190 | 0.129 | 7.08e-01 |
| edge_pheno249 | childhood_BMI | Weighted mode | 0.018 | 0.067 | -0.113 | 0.150 | 7.85e-01 |
| edge_pheno253 | childhood_BMI | MR Egger | 0.112 | 0.080 | -0.044 | 0.269 | 1.69e-01 |
| edge_pheno253 | childhood_BMI | Weighted median | 0.037 | 0.039 | -0.039 | 0.114 | 3.41e-01 |
| edge_pheno253 | childhood_BMI | Simple mode | 0.036 | 0.074 | -0.110 | 0.182 | 6.31e-01 |
| edge_pheno253 | childhood_BMI | Weighted mode | 0.063 | 0.060 | -0.055 | 0.180 | 3.03e-01 |
| edge_pheno262 | childhood_BMI | MR Egger | -0.131 | 0.087 | -0.301 | 0.039 | 1.40e-01 |
| edge_pheno262 | childhood_BMI | Weighted median | 0.003 | 0.038 | -0.072 | 0.078 | 9.34e-01 |
| edge_pheno262 | childhood_BMI | Simple mode | 0.016 | 0.072 | -0.126 | 0.158 | 8.25e-01 |
| edge_pheno262 | childhood_BMI | Weighted mode | 0.009 | 0.057 | -0.102 | 0.120 | 8.72e-01 |
| edge_pheno286 | childhood_BMI | MR Egger | -0.080 | 0.095 | -0.266 | 0.106 | 4.07e-01 |
| edge_pheno286 | childhood_BMI | Weighted median | -0.041 | 0.041 | -0.122 | 0.040 | 3.25e-01 |
| edge_pheno286 | childhood_BMI | Simple mode | -0.020 | 0.075 | -0.168 | 0.128 | 7.95e-01 |
| edge_pheno286 | childhood_BMI | Weighted mode | -0.038 | 0.056 | -0.148 | 0.073 | 5.09e-01 |
| edge_pheno288 | childhood_BMI | MR Egger | -0.228 | 0.080 | -0.384 | -0.071 | 7.48e-03 |
| edge_pheno288 | childhood_BMI | Weighted median | 0.009 | 0.040 | -0.070 | 0.089 | 8.16e-01 |
| edge_pheno288 | childhood_BMI | Simple mode | 0.117 | 0.085 | -0.049 | 0.283 | 1.76e-01 |
| edge_pheno288 | childhood_BMI | Weighted mode | 0.016 | 0.069 | -0.120 | 0.152 | 8.20e-01 |
| edge_pheno303 | childhood_BMI | MR Egger | -0.009 | 0.085 | -0.175 | 0.156 | 9.13e-01 |
| edge_pheno303 | childhood_BMI | Weighted median | -0.027 | 0.040 | -0.106 | 0.051 | 4.95e-01 |
| edge_pheno303 | childhood_BMI | Simple mode | -0.007 | 0.079 | -0.162 | 0.149 | 9.34e-01 |
| edge_pheno303 | childhood_BMI | Weighted mode | -0.037 | 0.066 | -0.165 | 0.092 | 5.81e-01 |
| edge_pheno389 | childhood_BMI | MR Egger | -0.092 | 0.080 | -0.249 | 0.065 | 2.61e-01 |
| edge_pheno389 | childhood_BMI | Weighted median | -0.012 | 0.041 | -0.093 | 0.069 | 7.69e-01 |
| edge_pheno389 | childhood_BMI | Simple mode | -0.045 | 0.079 | -0.199 | 0.110 | 5.75e-01 |
| edge_pheno389 | childhood_BMI | Weighted mode | -0.006 | 0.060 | -0.123 | 0.112 | 9.27e-01 |
| edge_pheno405 | childhood_BMI | MR Egger | 0.227 | 0.087 | 0.056 | 0.399 | 1.37e-02 |
| edge_pheno405 | childhood_BMI | Weighted median | 0.073 | 0.041 | -0.007 | 0.153 | 7.40e-02 |
| edge_pheno405 | childhood_BMI | Simple mode | 0.070 | 0.080 | -0.087 | 0.227 | 3.90e-01 |
| edge_pheno405 | childhood_BMI | Weighted mode | 0.086 | 0.068 | -0.047 | 0.219 | 2.13e-01 |
| edge_pheno447 | childhood_BMI | MR Egger | 0.270 | 0.226 | -0.173 | 0.714 | 3.18e-01 |
| edge_pheno447 | childhood_BMI | Weighted median | -0.025 | 0.088 | -0.197 | 0.148 | 7.80e-01 |
| edge_pheno447 | childhood_BMI | Simple mode | -0.013 | 0.138 | -0.284 | 0.258 | 9.31e-01 |
| edge_pheno447 | childhood_BMI | Weighted mode | 0.042 | 0.118 | -0.189 | 0.273 | 7.39e-01 |
| edge_pheno449 | childhood_BMI | MR Egger | -0.127 | 0.265 | -0.646 | 0.391 | 6.63e-01 |
| edge_pheno449 | childhood_BMI | Weighted median | 0.013 | 0.087 | -0.157 | 0.183 | 8.82e-01 |
| edge_pheno449 | childhood_BMI | Simple mode | 0.114 | 0.152 | -0.185 | 0.413 | 4.96e-01 |
| edge_pheno449 | childhood_BMI | Weighted mode | 0.117 | 0.156 | -0.188 | 0.423 | 4.93e-01 |
| edge_pheno460 | childhood_BMI | MR Egger | 0.025 | 0.082 | -0.135 | 0.186 | 7.60e-01 |
| edge_pheno460 | childhood_BMI | Weighted median | 0.044 | 0.040 | -0.034 | 0.122 | 2.71e-01 |
| edge_pheno460 | childhood_BMI | Simple mode | 0.010 | 0.071 | -0.130 | 0.151 | 8.85e-01 |
| edge_pheno460 | childhood_BMI | Weighted mode | 0.044 | 0.064 | -0.081 | 0.169 | 4.97e-01 |
| edge_pheno461 | childhood_BMI | MR Egger | -0.060 | 0.080 | -0.217 | 0.096 | 4.54e-01 |
| edge_pheno461 | childhood_BMI | Weighted median | -0.047 | 0.039 | -0.124 | 0.030 | 2.35e-01 |
| edge_pheno461 | childhood_BMI | Simple mode | -0.046 | 0.073 | -0.189 | 0.098 | 5.35e-01 |
| edge_pheno461 | childhood_BMI | Weighted mode | -0.043 | 0.062 | -0.165 | 0.078 | 4.89e-01 |
| edge_pheno491 | childhood_BMI | MR Egger | -0.161 | 0.080 | -0.318 | -0.005 | 5.10e-02 |
| edge_pheno491 | childhood_BMI | Weighted median | 0.010 | 0.042 | -0.071 | 0.092 | 8.04e-01 |
| edge_pheno491 | childhood_BMI | Simple mode | 0.074 | 0.079 | -0.082 | 0.229 | 3.59e-01 |
| edge_pheno491 | childhood_BMI | Weighted mode | -0.078 | 0.076 | -0.227 | 0.070 | 3.09e-01 |
| edge_pheno537 | childhood_BMI | MR Egger | 0.004 | 0.077 | -0.148 | 0.156 | 9.57e-01 |
| edge_pheno537 | childhood_BMI | Weighted median | -0.056 | 0.039 | -0.133 | 0.021 | 1.54e-01 |
| edge_pheno537 | childhood_BMI | Simple mode | -0.111 | 0.082 | -0.271 | 0.049 | 1.84e-01 |
| edge_pheno537 | childhood_BMI | Weighted mode | -0.105 | 0.075 | -0.252 | 0.042 | 1.71e-01 |
| edge_pheno55 | childhood_BMI | MR Egger | 0.086 | 0.088 | -0.086 | 0.258 | 3.32e-01 |
| edge_pheno55 | childhood_BMI | Weighted median | 0.046 | 0.040 | -0.031 | 0.124 | 2.44e-01 |
| edge_pheno55 | childhood_BMI | Simple mode | 0.048 | 0.074 | -0.097 | 0.193 | 5.18e-01 |
| edge_pheno55 | childhood_BMI | Weighted mode | 0.077 | 0.061 | -0.041 | 0.196 | 2.10e-01 |
| edge_pheno558 | childhood_BMI | MR Egger | -0.149 | 0.087 | -0.320 | 0.022 | 9.65e-02 |
| edge_pheno558 | childhood_BMI | Weighted median | -0.048 | 0.041 | -0.129 | 0.032 | 2.41e-01 |
| edge_pheno558 | childhood_BMI | Simple mode | -0.061 | 0.092 | -0.241 | 0.119 | 5.09e-01 |
| edge_pheno558 | childhood_BMI | Weighted mode | -0.059 | 0.077 | -0.210 | 0.092 | 4.52e-01 |
| edge_pheno574 | childhood_BMI | MR Egger | -0.175 | 0.078 | -0.327 | -0.022 | 3.14e-02 |
| edge_pheno574 | childhood_BMI | Weighted median | -0.088 | 0.038 | -0.162 | -0.015 | 1.87e-02 |
| edge_pheno574 | childhood_BMI | Simple mode | -0.064 | 0.074 | -0.209 | 0.080 | 3.90e-01 |
| edge_pheno574 | childhood_BMI | Weighted mode | -0.077 | 0.061 | -0.197 | 0.042 | 2.12e-01 |
| edge_pheno58 | childhood_BMI | MR Egger | -0.012 | 0.080 | -0.169 | 0.144 | 8.80e-01 |
| edge_pheno58 | childhood_BMI | Weighted median | 0.006 | 0.039 | -0.070 | 0.082 | 8.78e-01 |
| edge_pheno58 | childhood_BMI | Simple mode | 0.009 | 0.070 | -0.128 | 0.145 | 9.04e-01 |
| edge_pheno58 | childhood_BMI | Weighted mode | 0.015 | 0.051 | -0.085 | 0.116 | 7.69e-01 |
| edge_pheno590 | childhood_BMI | MR Egger | 0.102 | 0.082 | -0.059 | 0.264 | 2.24e-01 |
| edge_pheno590 | childhood_BMI | Weighted median | 0.023 | 0.041 | -0.057 | 0.104 | 5.71e-01 |
| edge_pheno590 | childhood_BMI | Simple mode | 0.064 | 0.080 | -0.093 | 0.220 | 4.30e-01 |
| edge_pheno590 | childhood_BMI | Weighted mode | 0.064 | 0.067 | -0.068 | 0.196 | 3.50e-01 |
| edge_pheno593 | childhood_BMI | MR Egger | -0.076 | 0.079 | -0.231 | 0.079 | 3.43e-01 |
| edge_pheno593 | childhood_BMI | Weighted median | 0.031 | 0.040 | -0.048 | 0.110 | 4.42e-01 |
| edge_pheno593 | childhood_BMI | Simple mode | 0.096 | 0.079 | -0.059 | 0.250 | 2.33e-01 |
| edge_pheno593 | childhood_BMI | Weighted mode | 0.073 | 0.076 | -0.077 | 0.223 | 3.45e-01 |
| edge_pheno597 | childhood_BMI | MR Egger | -0.098 | 0.078 | -0.251 | 0.056 | 2.23e-01 |
| edge_pheno597 | childhood_BMI | Weighted median | 0.007 | 0.039 | -0.070 | 0.085 | 8.51e-01 |
| edge_pheno597 | childhood_BMI | Simple mode | -0.037 | 0.075 | -0.183 | 0.109 | 6.24e-01 |
| edge_pheno597 | childhood_BMI | Weighted mode | -0.037 | 0.066 | -0.167 | 0.093 | 5.80e-01 |
| edge_pheno599 | childhood_BMI | MR Egger | -0.066 | 0.080 | -0.222 | 0.090 | 4.14e-01 |
| edge_pheno599 | childhood_BMI | Weighted median | 0.018 | 0.042 | -0.064 | 0.100 | 6.68e-01 |
| edge_pheno599 | childhood_BMI | Simple mode | 0.048 | 0.082 | -0.113 | 0.209 | 5.62e-01 |
| edge_pheno599 | childhood_BMI | Weighted mode | 0.038 | 0.078 | -0.115 | 0.191 | 6.26e-01 |
| edge_pheno601 | childhood_BMI | MR Egger | 0.037 | 0.092 | -0.143 | 0.217 | 6.90e-01 |
| edge_pheno601 | childhood_BMI | Weighted median | 0.003 | 0.041 | -0.077 | 0.082 | 9.46e-01 |
| edge_pheno601 | childhood_BMI | Simple mode | 0.027 | 0.077 | -0.123 | 0.177 | 7.28e-01 |
| edge_pheno601 | childhood_BMI | Weighted mode | 0.006 | 0.068 | -0.127 | 0.140 | 9.26e-01 |
| edge_pheno606 | childhood_BMI | MR Egger | 0.067 | 0.084 | -0.098 | 0.232 | 4.33e-01 |
| edge_pheno606 | childhood_BMI | Weighted median | -0.012 | 0.043 | -0.097 | 0.072 | 7.77e-01 |
| edge_pheno606 | childhood_BMI | Simple mode | -0.015 | 0.084 | -0.180 | 0.150 | 8.56e-01 |
| edge_pheno606 | childhood_BMI | Weighted mode | -0.002 | 0.066 | -0.131 | 0.127 | 9.74e-01 |
| edge_pheno609 | childhood_BMI | MR Egger | -0.045 | 0.084 | -0.209 | 0.119 | 5.94e-01 |
| edge_pheno609 | childhood_BMI | Weighted median | -0.008 | 0.040 | -0.087 | 0.071 | 8.44e-01 |
| edge_pheno609 | childhood_BMI | Simple mode | -0.044 | 0.079 | -0.199 | 0.112 | 5.85e-01 |
| edge_pheno609 | childhood_BMI | Weighted mode | -0.046 | 0.061 | -0.166 | 0.073 | 4.54e-01 |
| edge_pheno621 | childhood_BMI | MR Egger | -0.106 | 0.082 | -0.267 | 0.055 | 2.07e-01 |
| edge_pheno621 | childhood_BMI | Weighted median | -0.018 | 0.039 | -0.095 | 0.059 | 6.41e-01 |
| edge_pheno621 | childhood_BMI | Simple mode | -0.020 | 0.082 | -0.182 | 0.141 | 8.08e-01 |
| edge_pheno621 | childhood_BMI | Weighted mode | -0.035 | 0.058 | -0.150 | 0.079 | 5.49e-01 |
| edge_pheno624 | childhood_BMI | MR Egger | -0.043 | 0.080 | -0.199 | 0.113 | 5.96e-01 |
| edge_pheno624 | childhood_BMI | Weighted median | -0.005 | 0.041 | -0.086 | 0.076 | 9.05e-01 |
| edge_pheno624 | childhood_BMI | Simple mode | 0.092 | 0.088 | -0.081 | 0.265 | 3.06e-01 |
| edge_pheno624 | childhood_BMI | Weighted mode | 0.103 | 0.078 | -0.050 | 0.257 | 1.96e-01 |
| edge_pheno636 | childhood_BMI | MR Egger | -0.040 | 0.093 | -0.222 | 0.141 | 6.65e-01 |
| edge_pheno636 | childhood_BMI | Weighted median | 0.015 | 0.040 | -0.063 | 0.092 | 7.12e-01 |
| edge_pheno636 | childhood_BMI | Simple mode | 0.011 | 0.068 | -0.123 | 0.144 | 8.77e-01 |
| edge_pheno636 | childhood_BMI | Weighted mode | 0.013 | 0.058 | -0.101 | 0.128 | 8.20e-01 |
| edge_pheno639 | childhood_BMI | MR Egger | -0.125 | 0.079 | -0.279 | 0.029 | 1.20e-01 |
| edge_pheno639 | childhood_BMI | Weighted median | -0.069 | 0.038 | -0.142 | 0.005 | 6.83e-02 |
| edge_pheno639 | childhood_BMI | Simple mode | -0.087 | 0.070 | -0.224 | 0.051 | 2.25e-01 |
| edge_pheno639 | childhood_BMI | Weighted mode | -0.082 | 0.051 | -0.182 | 0.019 | 1.19e-01 |
| edge_pheno65 | childhood_BMI | MR Egger | 0.054 | 0.079 | -0.101 | 0.210 | 4.98e-01 |
| edge_pheno65 | childhood_BMI | Weighted median | 0.039 | 0.039 | -0.036 | 0.115 | 3.08e-01 |
| edge_pheno65 | childhood_BMI | Simple mode | -0.003 | 0.069 | -0.138 | 0.133 | 9.71e-01 |
| edge_pheno65 | childhood_BMI | Weighted mode | 0.028 | 0.058 | -0.086 | 0.143 | 6.29e-01 |
| edge_pheno66 | childhood_BMI | MR Egger | 0.055 | 0.084 | -0.110 | 0.220 | 5.18e-01 |
| edge_pheno66 | childhood_BMI | Weighted median | 0.008 | 0.039 | -0.069 | 0.085 | 8.44e-01 |
| edge_pheno66 | childhood_BMI | Simple mode | 0.008 | 0.073 | -0.136 | 0.152 | 9.12e-01 |
| edge_pheno66 | childhood_BMI | Weighted mode | -0.005 | 0.063 | -0.128 | 0.119 | 9.40e-01 |
| edge_pheno674 | childhood_BMI | MR Egger | -0.020 | 0.082 | -0.180 | 0.140 | 8.09e-01 |
| edge_pheno674 | childhood_BMI | Weighted median | 0.031 | 0.041 | -0.050 | 0.111 | 4.54e-01 |
| edge_pheno674 | childhood_BMI | Simple mode | 0.053 | 0.088 | -0.120 | 0.226 | 5.55e-01 |
| edge_pheno674 | childhood_BMI | Weighted mode | 0.061 | 0.075 | -0.086 | 0.209 | 4.21e-01 |
| edge_pheno681 | childhood_BMI | MR Egger | 0.062 | 0.080 | -0.094 | 0.219 | 4.42e-01 |
| edge_pheno681 | childhood_BMI | Weighted median | 0.004 | 0.040 | -0.075 | 0.082 | 9.30e-01 |
| edge_pheno681 | childhood_BMI | Simple mode | -0.028 | 0.080 | -0.185 | 0.130 | 7.32e-01 |
| edge_pheno681 | childhood_BMI | Weighted mode | -0.018 | 0.065 | -0.144 | 0.109 | 7.88e-01 |
| edge_pheno683 | childhood_BMI | MR Egger | -0.027 | 0.080 | -0.183 | 0.130 | 7.40e-01 |
| edge_pheno683 | childhood_BMI | Weighted median | -0.011 | 0.038 | -0.084 | 0.063 | 7.79e-01 |
| edge_pheno683 | childhood_BMI | Simple mode | -0.011 | 0.066 | -0.140 | 0.117 | 8.66e-01 |
| edge_pheno683 | childhood_BMI | Weighted mode | -0.018 | 0.053 | -0.121 | 0.086 | 7.40e-01 |
| edge_pheno695 | childhood_BMI | MR Egger | -0.058 | 0.079 | -0.214 | 0.097 | 4.68e-01 |
| edge_pheno695 | childhood_BMI | Weighted median | 0.040 | 0.039 | -0.036 | 0.116 | 3.02e-01 |
| edge_pheno695 | childhood_BMI | Simple mode | 0.083 | 0.075 | -0.064 | 0.230 | 2.76e-01 |
| edge_pheno695 | childhood_BMI | Weighted mode | 0.072 | 0.061 | -0.049 | 0.192 | 2.50e-01 |
| edge_pheno698 | childhood_BMI | MR Egger | -0.127 | 0.084 | -0.292 | 0.038 | 1.40e-01 |
| edge_pheno698 | childhood_BMI | Weighted median | -0.081 | 0.040 | -0.160 | -0.002 | 4.56e-02 |
| edge_pheno698 | childhood_BMI | Simple mode | -0.184 | 0.077 | -0.336 | -0.033 | 2.24e-02 |
| edge_pheno698 | childhood_BMI | Weighted mode | -0.118 | 0.061 | -0.237 | 0.002 | 6.24e-02 |
| edge_pheno705 | childhood_BMI | MR Egger | 0.139 | 0.082 | -0.021 | 0.299 | 9.84e-02 |
| edge_pheno705 | childhood_BMI | Weighted median | 0.053 | 0.043 | -0.031 | 0.137 | 2.18e-01 |
| edge_pheno705 | childhood_BMI | Simple mode | 0.025 | 0.092 | -0.155 | 0.205 | 7.88e-01 |
| edge_pheno705 | childhood_BMI | Weighted mode | 0.107 | 0.079 | -0.048 | 0.262 | 1.84e-01 |
| edge_pheno716 | childhood_BMI | MR Egger | 0.037 | 0.080 | -0.119 | 0.193 | 6.47e-01 |
| edge_pheno716 | childhood_BMI | Weighted median | -0.066 | 0.041 | -0.147 | 0.015 | 1.12e-01 |
| edge_pheno716 | childhood_BMI | Simple mode | 0.042 | 0.083 | -0.121 | 0.205 | 6.16e-01 |
| edge_pheno716 | childhood_BMI | Weighted mode | -0.091 | 0.069 | -0.226 | 0.043 | 1.92e-01 |
| edge_pheno767 | childhood_BMI | MR Egger | 0.030 | 0.090 | -0.147 | 0.206 | 7.45e-01 |
| edge_pheno767 | childhood_BMI | Weighted median | 0.002 | 0.041 | -0.079 | 0.083 | 9.54e-01 |
| edge_pheno767 | childhood_BMI | Simple mode | -0.013 | 0.076 | -0.162 | 0.136 | 8.61e-01 |
| edge_pheno767 | childhood_BMI | Weighted mode | 0.003 | 0.062 | -0.118 | 0.124 | 9.58e-01 |
| edge_pheno777 | childhood_BMI | MR Egger | -0.081 | 0.090 | -0.257 | 0.096 | 3.78e-01 |
| edge_pheno777 | childhood_BMI | Weighted median | -0.030 | 0.041 | -0.110 | 0.051 | 4.71e-01 |
| edge_pheno777 | childhood_BMI | Simple mode | -0.016 | 0.079 | -0.171 | 0.139 | 8.39e-01 |
| edge_pheno777 | childhood_BMI | Weighted mode | -0.029 | 0.067 | -0.160 | 0.101 | 6.64e-01 |
| edge_pheno789 | childhood_BMI | MR Egger | 0.027 | 0.078 | -0.125 | 0.179 | 7.33e-01 |
| edge_pheno789 | childhood_BMI | Weighted median | 0.054 | 0.038 | -0.020 | 0.129 | 1.50e-01 |
| edge_pheno789 | childhood_BMI | Simple mode | 0.010 | 0.071 | -0.129 | 0.150 | 8.86e-01 |
| edge_pheno789 | childhood_BMI | Weighted mode | 0.032 | 0.063 | -0.092 | 0.156 | 6.20e-01 |
| edge_pheno794 | childhood_BMI | MR Egger | -0.124 | 0.078 | -0.277 | 0.029 | 1.23e-01 |
| edge_pheno794 | childhood_BMI | Weighted median | -0.064 | 0.038 | -0.138 | 0.011 | 9.35e-02 |
| edge_pheno794 | childhood_BMI | Simple mode | -0.081 | 0.069 | -0.217 | 0.055 | 2.53e-01 |
| edge_pheno794 | childhood_BMI | Weighted mode | -0.045 | 0.061 | -0.164 | 0.075 | 4.70e-01 |
| edge_pheno801 | childhood_BMI | MR Egger | 0.168 | 0.093 | -0.015 | 0.351 | 8.03e-02 |
| edge_pheno801 | childhood_BMI | Weighted median | 0.034 | 0.042 | -0.048 | 0.117 | 4.16e-01 |
| edge_pheno801 | childhood_BMI | Simple mode | 0.013 | 0.091 | -0.166 | 0.191 | 8.91e-01 |
| edge_pheno801 | childhood_BMI | Weighted mode | 0.027 | 0.085 | -0.140 | 0.194 | 7.53e-01 |
| edge_pheno810 | childhood_BMI | MR Egger | -0.075 | 0.077 | -0.225 | 0.075 | 3.34e-01 |
| edge_pheno810 | childhood_BMI | Weighted median | -0.025 | 0.036 | -0.097 | 0.046 | 4.88e-01 |
| edge_pheno810 | childhood_BMI | Simple mode | -0.054 | 0.066 | -0.182 | 0.075 | 4.17e-01 |
| edge_pheno810 | childhood_BMI | Weighted mode | -0.046 | 0.053 | -0.150 | 0.057 | 3.89e-01 |
| edge_pheno812 | childhood_BMI | MR Egger | -0.126 | 0.079 | -0.282 | 0.029 | 1.21e-01 |
| edge_pheno812 | childhood_BMI | Weighted median | -0.049 | 0.038 | -0.123 | 0.026 | 1.98e-01 |
| edge_pheno812 | childhood_BMI | Simple mode | -0.058 | 0.070 | -0.194 | 0.079 | 4.15e-01 |
| edge_pheno812 | childhood_BMI | Weighted mode | -0.064 | 0.054 | -0.170 | 0.042 | 2.45e-01 |
| edge_pheno815 | childhood_BMI | MR Egger | 0.022 | 0.079 | -0.133 | 0.176 | 7.84e-01 |
| edge_pheno815 | childhood_BMI | Weighted median | -0.008 | 0.040 | -0.087 | 0.071 | 8.48e-01 |
| edge_pheno815 | childhood_BMI | Simple mode | 0.019 | 0.080 | -0.137 | 0.176 | 8.10e-01 |
| edge_pheno815 | childhood_BMI | Weighted mode | 0.017 | 0.062 | -0.104 | 0.139 | 7.84e-01 |
| edge_pheno816 | childhood_BMI | MR Egger | 0.090 | 0.077 | -0.061 | 0.240 | 2.51e-01 |
| edge_pheno816 | childhood_BMI | Weighted median | -0.001 | 0.038 | -0.075 | 0.072 | 9.70e-01 |
| edge_pheno816 | childhood_BMI | Simple mode | -0.093 | 0.076 | -0.241 | 0.055 | 2.28e-01 |
| edge_pheno816 | childhood_BMI | Weighted mode | -0.084 | 0.067 | -0.215 | 0.047 | 2.18e-01 |
| edge_pheno824 | childhood_BMI | MR Egger | -0.034 | 0.096 | -0.222 | 0.155 | 7.28e-01 |
| edge_pheno824 | childhood_BMI | Weighted median | 0.065 | 0.040 | -0.014 | 0.143 | 1.09e-01 |
| edge_pheno824 | childhood_BMI | Simple mode | 0.086 | 0.089 | -0.089 | 0.261 | 3.42e-01 |
| edge_pheno824 | childhood_BMI | Weighted mode | 0.089 | 0.086 | -0.079 | 0.257 | 3.04e-01 |
| edge_pheno867 | childhood_BMI | MR Egger | 0.009 | 0.085 | -0.157 | 0.175 | 9.12e-01 |
| edge_pheno867 | childhood_BMI | Weighted median | 0.013 | 0.038 | -0.062 | 0.088 | 7.38e-01 |
| edge_pheno867 | childhood_BMI | Simple mode | 0.058 | 0.063 | -0.066 | 0.182 | 3.66e-01 |
| edge_pheno867 | childhood_BMI | Weighted mode | 0.040 | 0.058 | -0.074 | 0.154 | 4.99e-01 |
| edge_pheno869 | childhood_BMI | MR Egger | -0.030 | 0.103 | -0.233 | 0.172 | 7.72e-01 |
| edge_pheno869 | childhood_BMI | Weighted median | -0.040 | 0.042 | -0.122 | 0.043 | 3.45e-01 |
| edge_pheno869 | childhood_BMI | Simple mode | -0.029 | 0.092 | -0.209 | 0.152 | 7.57e-01 |
| edge_pheno869 | childhood_BMI | Weighted mode | -0.049 | 0.065 | -0.177 | 0.079 | 4.57e-01 |
| edge_pheno87 | childhood_BMI | MR Egger | 0.152 | 0.095 | -0.033 | 0.338 | 1.17e-01 |
| edge_pheno87 | childhood_BMI | Weighted median | -0.018 | 0.041 | -0.098 | 0.063 | 6.64e-01 |
| edge_pheno87 | childhood_BMI | Simple mode | -0.065 | 0.086 | -0.234 | 0.105 | 4.60e-01 |
| edge_pheno87 | childhood_BMI | Weighted mode | -0.029 | 0.069 | -0.164 | 0.107 | 6.80e-01 |
| edge_pheno882 | childhood_BMI | MR Egger | -0.105 | 0.091 | -0.284 | 0.074 | 2.57e-01 |
| edge_pheno882 | childhood_BMI | Weighted median | -0.022 | 0.042 | -0.104 | 0.060 | 6.01e-01 |
| edge_pheno882 | childhood_BMI | Simple mode | 0.100 | 0.083 | -0.062 | 0.262 | 2.35e-01 |
| edge_pheno882 | childhood_BMI | Weighted mode | -0.022 | 0.055 | -0.131 | 0.086 | 6.92e-01 |
| edge_pheno899 | childhood_BMI | MR Egger | 0.018 | 0.080 | -0.138 | 0.174 | 8.26e-01 |
| edge_pheno899 | childhood_BMI | Weighted median | 0.042 | 0.040 | -0.037 | 0.120 | 3.01e-01 |
| edge_pheno899 | childhood_BMI | Simple mode | 0.101 | 0.072 | -0.039 | 0.242 | 1.66e-01 |
| edge_pheno899 | childhood_BMI | Weighted mode | 0.075 | 0.066 | -0.054 | 0.204 | 2.61e-01 |
| edge_pheno903 | childhood_BMI | MR Egger | 0.063 | 0.079 | -0.092 | 0.218 | 4.30e-01 |
| edge_pheno903 | childhood_BMI | Weighted median | 0.018 | 0.037 | -0.054 | 0.091 | 6.24e-01 |
| edge_pheno903 | childhood_BMI | Simple mode | 0.031 | 0.070 | -0.106 | 0.168 | 6.61e-01 |
| edge_pheno903 | childhood_BMI | Weighted mode | 0.037 | 0.061 | -0.082 | 0.156 | 5.48e-01 |
| edge_pheno908 | childhood_BMI | MR Egger | -0.027 | 0.080 | -0.184 | 0.129 | 7.33e-01 |
| edge_pheno908 | childhood_BMI | Weighted median | 0.032 | 0.041 | -0.048 | 0.112 | 4.33e-01 |
| edge_pheno908 | childhood_BMI | Simple mode | -0.003 | 0.077 | -0.154 | 0.148 | 9.68e-01 |
| edge_pheno908 | childhood_BMI | Weighted mode | 0.010 | 0.068 | -0.124 | 0.144 | 8.85e-01 |
| edge_pheno918 | childhood_BMI | MR Egger | 0.003 | 0.082 | -0.158 | 0.165 | 9.70e-01 |
| edge_pheno918 | childhood_BMI | Weighted median | 0.003 | 0.039 | -0.074 | 0.080 | 9.42e-01 |
| edge_pheno918 | childhood_BMI | Simple mode | 0.032 | 0.074 | -0.113 | 0.178 | 6.65e-01 |
| edge_pheno918 | childhood_BMI | Weighted mode | -0.011 | 0.059 | -0.127 | 0.104 | 8.48e-01 |
| edge_pheno932 | childhood_BMI | MR Egger | 0.056 | 0.078 | -0.096 | 0.208 | 4.73e-01 |
| edge_pheno932 | childhood_BMI | Weighted median | 0.030 | 0.038 | -0.045 | 0.105 | 4.38e-01 |
| edge_pheno932 | childhood_BMI | Simple mode | -0.004 | 0.082 | -0.165 | 0.157 | 9.58e-01 |
| edge_pheno932 | childhood_BMI | Weighted mode | 0.041 | 0.072 | -0.100 | 0.182 | 5.75e-01 |
| edge_pheno942 | childhood_BMI | MR Egger | 0.131 | 0.078 | -0.023 | 0.284 | 1.06e-01 |
| edge_pheno942 | childhood_BMI | Weighted median | -0.018 | 0.039 | -0.095 | 0.059 | 6.45e-01 |
| edge_pheno942 | childhood_BMI | Simple mode | -0.063 | 0.077 | -0.214 | 0.089 | 4.23e-01 |
| edge_pheno942 | childhood_BMI | Weighted mode | -0.034 | 0.075 | -0.180 | 0.112 | 6.55e-01 |
| edge_pheno956 | childhood_BMI | MR Egger | 0.010 | 0.096 | -0.177 | 0.198 | 9.14e-01 |
| edge_pheno956 | childhood_BMI | Weighted median | -0.053 | 0.041 | -0.132 | 0.027 | 1.97e-01 |
| edge_pheno956 | childhood_BMI | Simple mode | -0.035 | 0.083 | -0.197 | 0.127 | 6.74e-01 |
| edge_pheno956 | childhood_BMI | Weighted mode | -0.044 | 0.059 | -0.160 | 0.073 | 4.66e-01 |
| edge_pheno965 | childhood_BMI | MR Egger | 0.057 | 0.083 | -0.106 | 0.220 | 4.94e-01 |
| edge_pheno965 | childhood_BMI | Weighted median | -0.012 | 0.041 | -0.093 | 0.069 | 7.69e-01 |
| edge_pheno965 | childhood_BMI | Simple mode | -0.071 | 0.081 | -0.229 | 0.087 | 3.86e-01 |
| edge_pheno965 | childhood_BMI | Weighted mode | -0.033 | 0.066 | -0.162 | 0.096 | 6.19e-01 |
| node_pheno1 | childhood_BMI | MR Egger | 0.162 | 0.087 | -0.009 | 0.332 | 7.20e-02 |
| node_pheno1 | childhood_BMI | Weighted median | 0.008 | 0.040 | -0.070 | 0.085 | 8.47e-01 |
| node_pheno1 | childhood_BMI | Simple mode | 0.009 | 0.074 | -0.136 | 0.154 | 9.04e-01 |
| node_pheno1 | childhood_BMI | Weighted mode | 0.006 | 0.064 | -0.119 | 0.131 | 9.24e-01 |
| node_pheno10 | childhood_BMI | MR Egger | 0.100 | 0.087 | -0.070 | 0.270 | 2.57e-01 |
| node_pheno10 | childhood_BMI | Weighted median | -0.021 | 0.040 | -0.099 | 0.056 | 5.89e-01 |
| node_pheno10 | childhood_BMI | Simple mode | -0.037 | 0.077 | -0.187 | 0.113 | 6.30e-01 |
| node_pheno10 | childhood_BMI | Weighted mode | -0.032 | 0.058 | -0.146 | 0.081 | 5.81e-01 |
| node_pheno11 | childhood_BMI | MR Egger | 0.001 | 0.090 | -0.175 | 0.177 | 9.92e-01 |
| node_pheno11 | childhood_BMI | Weighted median | -0.059 | 0.039 | -0.136 | 0.018 | 1.33e-01 |
| node_pheno11 | childhood_BMI | Simple mode | -0.091 | 0.075 | -0.237 | 0.055 | 2.31e-01 |
| node_pheno11 | childhood_BMI | Weighted mode | -0.078 | 0.062 | -0.200 | 0.043 | 2.16e-01 |
| node_pheno12 | childhood_BMI | MR Egger | 0.161 | 0.095 | -0.026 | 0.348 | 1.00e-01 |
| node_pheno12 | childhood_BMI | Weighted median | 0.057 | 0.039 | -0.020 | 0.133 | 1.48e-01 |
| node_pheno12 | childhood_BMI | Simple mode | 0.063 | 0.064 | -0.063 | 0.189 | 3.34e-01 |
| node_pheno12 | childhood_BMI | Weighted mode | 0.056 | 0.055 | -0.053 | 0.164 | 3.23e-01 |
| node_pheno13 | childhood_BMI | MR Egger | -0.003 | 0.078 | -0.156 | 0.150 | 9.68e-01 |
| node_pheno13 | childhood_BMI | Weighted median | -0.030 | 0.039 | -0.105 | 0.046 | 4.41e-01 |
| node_pheno13 | childhood_BMI | Simple mode | -0.005 | 0.064 | -0.132 | 0.121 | 9.34e-01 |
| node_pheno13 | childhood_BMI | Weighted mode | -0.059 | 0.059 | -0.175 | 0.057 | 3.28e-01 |
| node_pheno14 | childhood_BMI | MR Egger | 0.134 | 0.087 | -0.037 | 0.305 | 1.34e-01 |
| node_pheno14 | childhood_BMI | Weighted median | 0.019 | 0.040 | -0.059 | 0.097 | 6.29e-01 |
| node_pheno14 | childhood_BMI | Simple mode | 0.054 | 0.074 | -0.092 | 0.200 | 4.72e-01 |
| node_pheno14 | childhood_BMI | Weighted mode | 0.009 | 0.065 | -0.119 | 0.136 | 8.96e-01 |
| node_pheno15 | childhood_BMI | MR Egger | 0.070 | 0.090 | -0.106 | 0.246 | 4.40e-01 |
| node_pheno15 | childhood_BMI | Weighted median | 0.024 | 0.038 | -0.051 | 0.099 | 5.26e-01 |
| node_pheno15 | childhood_BMI | Simple mode | -0.005 | 0.071 | -0.144 | 0.134 | 9.45e-01 |
| node_pheno15 | childhood_BMI | Weighted mode | 0.012 | 0.053 | -0.091 | 0.116 | 8.19e-01 |
| node_pheno16 | childhood_BMI | MR Egger | 0.088 | 0.086 | -0.080 | 0.257 | 3.11e-01 |
| node_pheno16 | childhood_BMI | Weighted median | 0.061 | 0.037 | -0.013 | 0.134 | 1.04e-01 |
| node_pheno16 | childhood_BMI | Simple mode | 0.052 | 0.080 | -0.105 | 0.209 | 5.21e-01 |
| node_pheno16 | childhood_BMI | Weighted mode | 0.055 | 0.070 | -0.082 | 0.191 | 4.36e-01 |
| node_pheno17 | childhood_BMI | MR Egger | 0.063 | 0.097 | -0.126 | 0.253 | 5.17e-01 |
| node_pheno17 | childhood_BMI | Weighted median | 0.016 | 0.041 | -0.065 | 0.097 | 6.95e-01 |
| node_pheno17 | childhood_BMI | Simple mode | 0.026 | 0.074 | -0.118 | 0.170 | 7.24e-01 |
| node_pheno17 | childhood_BMI | Weighted mode | 0.032 | 0.071 | -0.108 | 0.171 | 6.57e-01 |
| node_pheno18 | childhood_BMI | MR Egger | 0.091 | 0.091 | -0.086 | 0.269 | 3.21e-01 |
| node_pheno18 | childhood_BMI | Weighted median | -0.004 | 0.040 | -0.081 | 0.074 | 9.23e-01 |
| node_pheno18 | childhood_BMI | Simple mode | -0.003 | 0.073 | -0.145 | 0.140 | 9.72e-01 |
| node_pheno18 | childhood_BMI | Weighted mode | 0.000 | 0.074 | -0.145 | 0.146 | 9.98e-01 |
| node_pheno19 | childhood_BMI | MR Egger | 0.069 | 0.094 | -0.115 | 0.253 | 4.67e-01 |
| node_pheno19 | childhood_BMI | Weighted median | 0.022 | 0.042 | -0.060 | 0.103 | 6.06e-01 |
| node_pheno19 | childhood_BMI | Simple mode | 0.109 | 0.091 | -0.069 | 0.286 | 2.37e-01 |
| node_pheno19 | childhood_BMI | Weighted mode | -0.051 | 0.069 | -0.187 | 0.085 | 4.66e-01 |
| node_pheno2 | childhood_BMI | MR Egger | 0.120 | 0.092 | -0.060 | 0.301 | 2.01e-01 |
| node_pheno2 | childhood_BMI | Weighted median | -0.022 | 0.042 | -0.104 | 0.059 | 5.91e-01 |
| node_pheno2 | childhood_BMI | Simple mode | 0.083 | 0.107 | -0.127 | 0.293 | 4.43e-01 |
| node_pheno2 | childhood_BMI | Weighted mode | -0.130 | 0.105 | -0.336 | 0.076 | 2.23e-01 |
| node_pheno20 | childhood_BMI | MR Egger | 0.155 | 0.108 | -0.056 | 0.366 | 1.59e-01 |
| node_pheno20 | childhood_BMI | Weighted median | 0.066 | 0.041 | -0.014 | 0.146 | 1.04e-01 |
| node_pheno20 | childhood_BMI | Simple mode | 0.058 | 0.074 | -0.086 | 0.203 | 4.35e-01 |
| node_pheno20 | childhood_BMI | Weighted mode | 0.079 | 0.074 | -0.066 | 0.224 | 2.92e-01 |
| node_pheno21 | childhood_BMI | MR Egger | 0.141 | 0.085 | -0.026 | 0.308 | 1.08e-01 |
| node_pheno21 | childhood_BMI | Weighted median | 0.058 | 0.040 | -0.021 | 0.137 | 1.49e-01 |
| node_pheno21 | childhood_BMI | Simple mode | 0.077 | 0.078 | -0.075 | 0.229 | 3.29e-01 |
| node_pheno21 | childhood_BMI | Weighted mode | 0.071 | 0.061 | -0.048 | 0.191 | 2.50e-01 |
| node_pheno22 | childhood_BMI | MR Egger | 0.052 | 0.087 | -0.119 | 0.223 | 5.56e-01 |
| node_pheno22 | childhood_BMI | Weighted median | -0.010 | 0.040 | -0.088 | 0.068 | 7.94e-01 |
| node_pheno22 | childhood_BMI | Simple mode | -0.144 | 0.100 | -0.340 | 0.052 | 1.58e-01 |
| node_pheno22 | childhood_BMI | Weighted mode | -0.090 | 0.092 | -0.270 | 0.089 | 3.31e-01 |
| node_pheno23 | childhood_BMI | MR Egger | 0.023 | 0.096 | -0.165 | 0.211 | 8.14e-01 |
| node_pheno23 | childhood_BMI | Weighted median | -0.026 | 0.037 | -0.098 | 0.047 | 4.88e-01 |
| node_pheno23 | childhood_BMI | Simple mode | 0.049 | 0.066 | -0.082 | 0.179 | 4.70e-01 |
| node_pheno23 | childhood_BMI | Weighted mode | -0.066 | 0.060 | -0.183 | 0.051 | 2.75e-01 |
| node_pheno24 | childhood_BMI | MR Egger | 0.072 | 0.090 | -0.106 | 0.249 | 4.34e-01 |
| node_pheno24 | childhood_BMI | Weighted median | 0.021 | 0.040 | -0.056 | 0.099 | 5.90e-01 |
| node_pheno24 | childhood_BMI | Simple mode | 0.000 | 0.070 | -0.137 | 0.136 | 9.97e-01 |
| node_pheno24 | childhood_BMI | Weighted mode | 0.011 | 0.061 | -0.109 | 0.131 | 8.58e-01 |
| node_pheno25 | childhood_BMI | MR Egger | 0.105 | 0.088 | -0.068 | 0.278 | 2.43e-01 |
| node_pheno25 | childhood_BMI | Weighted median | -0.013 | 0.038 | -0.088 | 0.062 | 7.33e-01 |
| node_pheno25 | childhood_BMI | Simple mode | -0.025 | 0.082 | -0.185 | 0.135 | 7.64e-01 |
| node_pheno25 | childhood_BMI | Weighted mode | -0.059 | 0.070 | -0.196 | 0.078 | 4.03e-01 |
| node_pheno26 | childhood_BMI | MR Egger | 0.126 | 0.072 | -0.016 | 0.268 | 9.05e-02 |
| node_pheno26 | childhood_BMI | Weighted median | 0.004 | 0.037 | -0.068 | 0.075 | 9.22e-01 |
| node_pheno26 | childhood_BMI | Simple mode | -0.012 | 0.067 | -0.143 | 0.119 | 8.63e-01 |
| node_pheno26 | childhood_BMI | Weighted mode | -0.014 | 0.059 | -0.129 | 0.101 | 8.13e-01 |
| node_pheno27 | childhood_BMI | MR Egger | -0.059 | 0.093 | -0.242 | 0.124 | 5.30e-01 |
| node_pheno27 | childhood_BMI | Weighted median | -0.041 | 0.038 | -0.116 | 0.035 | 2.90e-01 |
| node_pheno27 | childhood_BMI | Simple mode | -0.040 | 0.077 | -0.190 | 0.111 | 6.10e-01 |
| node_pheno27 | childhood_BMI | Weighted mode | -0.052 | 0.058 | -0.164 | 0.061 | 3.77e-01 |
| node_pheno28 | childhood_BMI | MR Egger | 0.064 | 0.086 | -0.104 | 0.233 | 4.59e-01 |
| node_pheno28 | childhood_BMI | Weighted median | 0.031 | 0.038 | -0.043 | 0.105 | 4.13e-01 |
| node_pheno28 | childhood_BMI | Simple mode | 0.027 | 0.067 | -0.104 | 0.157 | 6.92e-01 |
| node_pheno28 | childhood_BMI | Weighted mode | 0.033 | 0.060 | -0.084 | 0.150 | 5.82e-01 |
| node_pheno29 | childhood_BMI | MR Egger | 0.019 | 0.084 | -0.146 | 0.184 | 8.22e-01 |
| node_pheno29 | childhood_BMI | Weighted median | 0.067 | 0.039 | -0.010 | 0.144 | 8.81e-02 |
| node_pheno29 | childhood_BMI | Simple mode | 0.066 | 0.084 | -0.099 | 0.232 | 4.36e-01 |
| node_pheno29 | childhood_BMI | Weighted mode | 0.074 | 0.080 | -0.083 | 0.232 | 3.62e-01 |
| node_pheno3 | childhood_BMI | MR Egger | -0.004 | 0.088 | -0.176 | 0.168 | 9.63e-01 |
| node_pheno3 | childhood_BMI | Weighted median | -0.071 | 0.039 | -0.148 | 0.005 | 6.82e-02 |
| node_pheno3 | childhood_BMI | Simple mode | -0.098 | 0.070 | -0.236 | 0.040 | 1.73e-01 |
| node_pheno3 | childhood_BMI | Weighted mode | -0.083 | 0.061 | -0.203 | 0.038 | 1.87e-01 |
| node_pheno30 | childhood_BMI | MR Egger | 0.080 | 0.091 | -0.098 | 0.257 | 3.87e-01 |
| node_pheno30 | childhood_BMI | Weighted median | 0.034 | 0.038 | -0.040 | 0.109 | 3.67e-01 |
| node_pheno30 | childhood_BMI | Simple mode | 0.051 | 0.067 | -0.081 | 0.183 | 4.52e-01 |
| node_pheno30 | childhood_BMI | Weighted mode | 0.036 | 0.052 | -0.067 | 0.138 | 5.03e-01 |
| node_pheno31 | childhood_BMI | MR Egger | 0.057 | 0.083 | -0.105 | 0.219 | 4.97e-01 |
| node_pheno31 | childhood_BMI | Weighted median | -0.006 | 0.039 | -0.083 | 0.071 | 8.78e-01 |
| node_pheno31 | childhood_BMI | Simple mode | -0.045 | 0.080 | -0.202 | 0.112 | 5.79e-01 |
| node_pheno31 | childhood_BMI | Weighted mode | -0.039 | 0.071 | -0.179 | 0.101 | 5.89e-01 |
| node_pheno32 | childhood_BMI | MR Egger | -0.038 | 0.088 | -0.211 | 0.134 | 6.65e-01 |
| node_pheno32 | childhood_BMI | Weighted median | -0.006 | 0.036 | -0.077 | 0.065 | 8.73e-01 |
| node_pheno32 | childhood_BMI | Simple mode | -0.006 | 0.074 | -0.152 | 0.139 | 9.33e-01 |
| node_pheno32 | childhood_BMI | Weighted mode | 0.000 | 0.065 | -0.128 | 0.128 | 9.97e-01 |
| node_pheno33 | childhood_BMI | MR Egger | 0.166 | 0.076 | 0.018 | 0.315 | 3.55e-02 |
| node_pheno33 | childhood_BMI | Weighted median | 0.022 | 0.037 | -0.052 | 0.095 | 5.60e-01 |
| node_pheno33 | childhood_BMI | Simple mode | -0.064 | 0.073 | -0.206 | 0.078 | 3.84e-01 |
| node_pheno33 | childhood_BMI | Weighted mode | 0.019 | 0.056 | -0.091 | 0.128 | 7.39e-01 |
| node_pheno34 | childhood_BMI | MR Egger | 0.049 | 0.075 | -0.098 | 0.196 | 5.21e-01 |
| node_pheno34 | childhood_BMI | Weighted median | 0.027 | 0.037 | -0.046 | 0.099 | 4.73e-01 |
| node_pheno34 | childhood_BMI | Simple mode | 0.017 | 0.067 | -0.114 | 0.148 | 8.04e-01 |
| node_pheno34 | childhood_BMI | Weighted mode | 0.025 | 0.056 | -0.084 | 0.135 | 6.52e-01 |
| node_pheno35 | childhood_BMI | MR Egger | 0.111 | 0.094 | -0.073 | 0.296 | 2.45e-01 |
| node_pheno35 | childhood_BMI | Weighted median | 0.037 | 0.040 | -0.042 | 0.115 | 3.58e-01 |
| node_pheno35 | childhood_BMI | Simple mode | 0.032 | 0.078 | -0.120 | 0.184 | 6.82e-01 |
| node_pheno35 | childhood_BMI | Weighted mode | -0.006 | 0.073 | -0.149 | 0.138 | 9.36e-01 |
| node_pheno36 | childhood_BMI | MR Egger | 0.124 | 0.093 | -0.058 | 0.305 | 1.90e-01 |
| node_pheno36 | childhood_BMI | Weighted median | 0.016 | 0.037 | -0.056 | 0.089 | 6.56e-01 |
| node_pheno36 | childhood_BMI | Simple mode | -0.005 | 0.082 | -0.165 | 0.155 | 9.52e-01 |
| node_pheno36 | childhood_BMI | Weighted mode | 0.050 | 0.065 | -0.078 | 0.178 | 4.49e-01 |
| node_pheno37 | childhood_BMI | MR Egger | 0.033 | 0.088 | -0.140 | 0.206 | 7.13e-01 |
| node_pheno37 | childhood_BMI | Weighted median | -0.032 | 0.040 | -0.109 | 0.046 | 4.22e-01 |
| node_pheno37 | childhood_BMI | Simple mode | -0.080 | 0.088 | -0.254 | 0.093 | 3.71e-01 |
| node_pheno37 | childhood_BMI | Weighted mode | -0.070 | 0.065 | -0.197 | 0.057 | 2.89e-01 |
| node_pheno38 | childhood_BMI | MR Egger | 0.036 | 0.084 | -0.129 | 0.201 | 6.73e-01 |
| node_pheno38 | childhood_BMI | Weighted median | 0.062 | 0.037 | -0.010 | 0.134 | 8.96e-02 |
| node_pheno38 | childhood_BMI | Simple mode | 0.087 | 0.067 | -0.043 | 0.218 | 1.98e-01 |
| node_pheno38 | childhood_BMI | Weighted mode | 0.073 | 0.056 | -0.036 | 0.182 | 1.98e-01 |
| node_pheno39 | childhood_BMI | MR Egger | 0.000 | 0.084 | -0.165 | 0.165 | 9.99e-01 |
| node_pheno39 | childhood_BMI | Weighted median | 0.009 | 0.040 | -0.069 | 0.087 | 8.21e-01 |
| node_pheno39 | childhood_BMI | Simple mode | 0.022 | 0.075 | -0.125 | 0.169 | 7.69e-01 |
| node_pheno39 | childhood_BMI | Weighted mode | 0.015 | 0.061 | -0.106 | 0.135 | 8.13e-01 |
| node_pheno4 | childhood_BMI | MR Egger | 0.150 | 0.089 | -0.026 | 0.325 | 1.04e-01 |
| node_pheno4 | childhood_BMI | Weighted median | -0.024 | 0.040 | -0.102 | 0.054 | 5.41e-01 |
| node_pheno4 | childhood_BMI | Simple mode | -0.031 | 0.085 | -0.198 | 0.137 | 7.21e-01 |
| node_pheno4 | childhood_BMI | Weighted mode | 0.000 | 0.073 | -0.142 | 0.143 | 9.95e-01 |
| node_pheno40 | childhood_BMI | MR Egger | 0.130 | 0.103 | -0.071 | 0.332 | 2.14e-01 |
| node_pheno40 | childhood_BMI | Weighted median | 0.029 | 0.039 | -0.047 | 0.106 | 4.54e-01 |
| node_pheno40 | childhood_BMI | Simple mode | 0.024 | 0.065 | -0.103 | 0.151 | 7.16e-01 |
| node_pheno40 | childhood_BMI | Weighted mode | 0.020 | 0.062 | -0.102 | 0.143 | 7.45e-01 |
| node_pheno41 | childhood_BMI | MR Egger | 0.123 | 0.091 | -0.057 | 0.302 | 1.89e-01 |
| node_pheno41 | childhood_BMI | Weighted median | 0.001 | 0.039 | -0.076 | 0.079 | 9.72e-01 |
| node_pheno41 | childhood_BMI | Simple mode | -0.048 | 0.072 | -0.189 | 0.093 | 5.06e-01 |
| node_pheno41 | childhood_BMI | Weighted mode | -0.028 | 0.055 | -0.135 | 0.080 | 6.18e-01 |
| node_pheno42 | childhood_BMI | MR Egger | 0.073 | 0.089 | -0.102 | 0.247 | 4.21e-01 |
| node_pheno42 | childhood_BMI | Weighted median | 0.019 | 0.039 | -0.057 | 0.094 | 6.25e-01 |
| node_pheno42 | childhood_BMI | Simple mode | 0.034 | 0.068 | -0.099 | 0.166 | 6.23e-01 |
| node_pheno42 | childhood_BMI | Weighted mode | 0.036 | 0.070 | -0.101 | 0.173 | 6.07e-01 |
| node_pheno43 | childhood_BMI | MR Egger | 0.107 | 0.098 | -0.085 | 0.298 | 2.83e-01 |
| node_pheno43 | childhood_BMI | Weighted median | -0.011 | 0.040 | -0.090 | 0.068 | 7.82e-01 |
| node_pheno43 | childhood_BMI | Simple mode | -0.008 | 0.080 | -0.165 | 0.150 | 9.26e-01 |
| node_pheno43 | childhood_BMI | Weighted mode | -0.020 | 0.058 | -0.133 | 0.093 | 7.35e-01 |
| node_pheno44 | childhood_BMI | MR Egger | 0.067 | 0.071 | -0.073 | 0.207 | 3.56e-01 |
| node_pheno44 | childhood_BMI | Weighted median | 0.021 | 0.034 | -0.046 | 0.089 | 5.32e-01 |
| node_pheno44 | childhood_BMI | Simple mode | -0.032 | 0.062 | -0.153 | 0.089 | 6.08e-01 |
| node_pheno44 | childhood_BMI | Weighted mode | 0.004 | 0.053 | -0.100 | 0.108 | 9.38e-01 |
| node_pheno45 | childhood_BMI | MR Egger | 0.017 | 0.095 | -0.169 | 0.204 | 8.57e-01 |
| node_pheno45 | childhood_BMI | Weighted median | -0.005 | 0.037 | -0.077 | 0.068 | 8.99e-01 |
| node_pheno45 | childhood_BMI | Simple mode | -0.023 | 0.064 | -0.149 | 0.103 | 7.26e-01 |
| node_pheno45 | childhood_BMI | Weighted mode | 0.001 | 0.056 | -0.109 | 0.111 | 9.85e-01 |
| node_pheno46 | childhood_BMI | MR Egger | 0.102 | 0.096 | -0.085 | 0.290 | 2.93e-01 |
| node_pheno46 | childhood_BMI | Weighted median | 0.035 | 0.039 | -0.042 | 0.111 | 3.77e-01 |
| node_pheno46 | childhood_BMI | Simple mode | -0.064 | 0.088 | -0.237 | 0.110 | 4.76e-01 |
| node_pheno46 | childhood_BMI | Weighted mode | -0.045 | 0.068 | -0.177 | 0.088 | 5.12e-01 |
| node_pheno47 | childhood_BMI | MR Egger | 0.113 | 0.092 | -0.067 | 0.293 | 2.27e-01 |
| node_pheno47 | childhood_BMI | Weighted median | -0.007 | 0.038 | -0.082 | 0.067 | 8.44e-01 |
| node_pheno47 | childhood_BMI | Simple mode | -0.016 | 0.065 | -0.143 | 0.111 | 8.01e-01 |
| node_pheno47 | childhood_BMI | Weighted mode | -0.019 | 0.050 | -0.118 | 0.079 | 7.03e-01 |
| node_pheno48 | childhood_BMI | MR Egger | 0.087 | 0.098 | -0.105 | 0.278 | 3.81e-01 |
| node_pheno48 | childhood_BMI | Weighted median | 0.013 | 0.041 | -0.068 | 0.094 | 7.52e-01 |
| node_pheno48 | childhood_BMI | Simple mode | 0.022 | 0.076 | -0.127 | 0.171 | 7.77e-01 |
| node_pheno48 | childhood_BMI | Weighted mode | 0.003 | 0.058 | -0.111 | 0.116 | 9.65e-01 |
| node_pheno49 | childhood_BMI | MR Egger | 0.002 | 0.079 | -0.153 | 0.157 | 9.78e-01 |
| node_pheno49 | childhood_BMI | Weighted median | -0.040 | 0.039 | -0.116 | 0.036 | 3.02e-01 |
| node_pheno49 | childhood_BMI | Simple mode | -0.028 | 0.067 | -0.158 | 0.103 | 6.81e-01 |
| node_pheno49 | childhood_BMI | Weighted mode | -0.048 | 0.051 | -0.149 | 0.053 | 3.57e-01 |
| node_pheno5 | childhood_BMI | MR Egger | 0.233 | 0.092 | 0.053 | 0.413 | 1.62e-02 |
| node_pheno5 | childhood_BMI | Weighted median | 0.004 | 0.038 | -0.071 | 0.079 | 9.14e-01 |
| node_pheno5 | childhood_BMI | Simple mode | -0.051 | 0.069 | -0.187 | 0.084 | 4.62e-01 |
| node_pheno5 | childhood_BMI | Weighted mode | -0.035 | 0.063 | -0.159 | 0.089 | 5.79e-01 |
| node_pheno50 | childhood_BMI | MR Egger | 0.003 | 0.092 | -0.177 | 0.184 | 9.70e-01 |
| node_pheno50 | childhood_BMI | Weighted median | 0.022 | 0.038 | -0.052 | 0.097 | 5.57e-01 |
| node_pheno50 | childhood_BMI | Simple mode | 0.002 | 0.072 | -0.140 | 0.143 | 9.82e-01 |
| node_pheno50 | childhood_BMI | Weighted mode | 0.016 | 0.064 | -0.109 | 0.141 | 8.04e-01 |
| node_pheno51 | childhood_BMI | MR Egger | 0.174 | 0.089 | 0.000 | 0.348 | 5.79e-02 |
| node_pheno51 | childhood_BMI | Weighted median | 0.042 | 0.038 | -0.033 | 0.117 | 2.74e-01 |
| node_pheno51 | childhood_BMI | Simple mode | -0.053 | 0.076 | -0.202 | 0.095 | 4.87e-01 |
| node_pheno51 | childhood_BMI | Weighted mode | 0.066 | 0.059 | -0.051 | 0.182 | 2.78e-01 |
| node_pheno52 | childhood_BMI | MR Egger | 0.079 | 0.082 | -0.081 | 0.240 | 3.41e-01 |
| node_pheno52 | childhood_BMI | Weighted median | 0.035 | 0.039 | -0.043 | 0.112 | 3.79e-01 |
| node_pheno52 | childhood_BMI | Simple mode | 0.043 | 0.077 | -0.109 | 0.194 | 5.84e-01 |
| node_pheno52 | childhood_BMI | Weighted mode | 0.065 | 0.075 | -0.082 | 0.211 | 3.94e-01 |
| node_pheno53 | childhood_BMI | MR Egger | 0.112 | 0.093 | -0.072 | 0.295 | 2.41e-01 |
| node_pheno53 | childhood_BMI | Weighted median | 0.051 | 0.040 | -0.027 | 0.129 | 2.00e-01 |
| node_pheno53 | childhood_BMI | Simple mode | 0.102 | 0.071 | -0.037 | 0.240 | 1.58e-01 |
| node_pheno53 | childhood_BMI | Weighted mode | 0.055 | 0.056 | -0.054 | 0.165 | 3.28e-01 |
| node_pheno54 | childhood_BMI | MR Egger | 0.064 | 0.088 | -0.108 | 0.237 | 4.72e-01 |
| node_pheno54 | childhood_BMI | Weighted median | 0.024 | 0.040 | -0.055 | 0.103 | 5.55e-01 |
| node_pheno54 | childhood_BMI | Simple mode | -0.045 | 0.075 | -0.192 | 0.102 | 5.54e-01 |
| node_pheno54 | childhood_BMI | Weighted mode | 0.049 | 0.066 | -0.081 | 0.178 | 4.65e-01 |
| node_pheno55 | childhood_BMI | MR Egger | 0.077 | 0.092 | -0.103 | 0.258 | 4.08e-01 |
| node_pheno55 | childhood_BMI | Weighted median | -0.006 | 0.036 | -0.077 | 0.065 | 8.65e-01 |
| node_pheno55 | childhood_BMI | Simple mode | -0.017 | 0.059 | -0.132 | 0.099 | 7.77e-01 |
| node_pheno55 | childhood_BMI | Weighted mode | -0.011 | 0.052 | -0.112 | 0.091 | 8.36e-01 |
| node_pheno56 | childhood_BMI | MR Egger | 0.097 | 0.103 | -0.105 | 0.299 | 3.54e-01 |
| node_pheno56 | childhood_BMI | Weighted median | -0.016 | 0.040 | -0.094 | 0.063 | 6.98e-01 |
| node_pheno56 | childhood_BMI | Simple mode | -0.013 | 0.075 | -0.160 | 0.134 | 8.64e-01 |
| node_pheno56 | childhood_BMI | Weighted mode | -0.016 | 0.055 | -0.124 | 0.092 | 7.70e-01 |
| node_pheno57 | childhood_BMI | MR Egger | 0.096 | 0.095 | -0.089 | 0.282 | 3.15e-01 |
| node_pheno57 | childhood_BMI | Weighted median | 0.068 | 0.040 | -0.011 | 0.147 | 8.99e-02 |
| node_pheno57 | childhood_BMI | Simple mode | 0.115 | 0.084 | -0.050 | 0.281 | 1.81e-01 |
| node_pheno57 | childhood_BMI | Weighted mode | 0.099 | 0.075 | -0.047 | 0.246 | 1.93e-01 |
| node_pheno58 | childhood_BMI | MR Egger | 0.056 | 0.089 | -0.117 | 0.230 | 5.29e-01 |
| node_pheno58 | childhood_BMI | Weighted median | 0.016 | 0.040 | -0.062 | 0.094 | 6.80e-01 |
| node_pheno58 | childhood_BMI | Simple mode | 0.002 | 0.076 | -0.147 | 0.151 | 9.83e-01 |
| node_pheno58 | childhood_BMI | Weighted mode | -0.004 | 0.055 | -0.111 | 0.103 | 9.47e-01 |
| node_pheno59 | childhood_BMI | MR Egger | 0.103 | 0.075 | -0.045 | 0.250 | 1.82e-01 |
| node_pheno59 | childhood_BMI | Weighted median | 0.003 | 0.036 | -0.067 | 0.073 | 9.24e-01 |
| node_pheno59 | childhood_BMI | Simple mode | -0.095 | 0.083 | -0.258 | 0.068 | 2.62e-01 |
| node_pheno59 | childhood_BMI | Weighted mode | 0.107 | 0.066 | -0.022 | 0.237 | 1.12e-01 |
| node_pheno60 | childhood_BMI | MR Egger | -0.006 | 0.097 | -0.197 | 0.184 | 9.49e-01 |
| node_pheno60 | childhood_BMI | Weighted median | 0.001 | 0.038 | -0.073 | 0.075 | 9.73e-01 |
| node_pheno60 | childhood_BMI | Simple mode | 0.041 | 0.072 | -0.100 | 0.181 | 5.73e-01 |
| node_pheno60 | childhood_BMI | Weighted mode | -0.013 | 0.052 | -0.116 | 0.090 | 8.06e-01 |
| node_pheno61 | childhood_BMI | MR Egger | 0.066 | 0.092 | -0.114 | 0.245 | 4.80e-01 |
| node_pheno61 | childhood_BMI | Weighted median | 0.032 | 0.040 | -0.045 | 0.110 | 4.14e-01 |
| node_pheno61 | childhood_BMI | Simple mode | 0.050 | 0.077 | -0.100 | 0.200 | 5.17e-01 |
| node_pheno61 | childhood_BMI | Weighted mode | 0.047 | 0.067 | -0.083 | 0.178 | 4.84e-01 |
| node_pheno62 | childhood_BMI | MR Egger | 0.179 | 0.114 | -0.045 | 0.403 | 1.28e-01 |
| node_pheno62 | childhood_BMI | Weighted median | 0.059 | 0.044 | -0.026 | 0.145 | 1.76e-01 |
| node_pheno62 | childhood_BMI | Simple mode | 0.160 | 0.106 | -0.047 | 0.368 | 1.39e-01 |
| node_pheno62 | childhood_BMI | Weighted mode | 0.139 | 0.100 | -0.057 | 0.336 | 1.74e-01 |
| node_pheno63 | childhood_BMI | MR Egger | -0.007 | 0.086 | -0.176 | 0.162 | 9.36e-01 |
| node_pheno63 | childhood_BMI | Weighted median | 0.045 | 0.039 | -0.032 | 0.122 | 2.54e-01 |
| node_pheno63 | childhood_BMI | Simple mode | 0.102 | 0.078 | -0.052 | 0.255 | 2.02e-01 |
| node_pheno63 | childhood_BMI | Weighted mode | 0.055 | 0.070 | -0.082 | 0.192 | 4.35e-01 |
| node_pheno64 | childhood_BMI | MR Egger | 0.019 | 0.071 | -0.120 | 0.158 | 7.88e-01 |
| node_pheno64 | childhood_BMI | Weighted median | 0.031 | 0.035 | -0.039 | 0.101 | 3.82e-01 |
| node_pheno64 | childhood_BMI | Simple mode | 0.081 | 0.073 | -0.063 | 0.224 | 2.78e-01 |
| node_pheno64 | childhood_BMI | Weighted mode | -0.004 | 0.065 | -0.130 | 0.123 | 9.55e-01 |
| node_pheno65 | childhood_BMI | MR Egger | 0.072 | 0.088 | -0.100 | 0.245 | 4.17e-01 |
| node_pheno65 | childhood_BMI | Weighted median | 0.067 | 0.040 | -0.012 | 0.146 | 9.80e-02 |
| node_pheno65 | childhood_BMI | Simple mode | 0.121 | 0.095 | -0.066 | 0.308 | 2.12e-01 |
| node_pheno65 | childhood_BMI | Weighted mode | 0.138 | 0.073 | -0.005 | 0.282 | 6.69e-02 |
| node_pheno66 | childhood_BMI | MR Egger | 0.000 | 0.094 | -0.183 | 0.183 | 9.99e-01 |
| node_pheno66 | childhood_BMI | Weighted median | 0.091 | 0.041 | 0.011 | 0.171 | 2.52e-02 |
| node_pheno66 | childhood_BMI | Simple mode | 0.109 | 0.085 | -0.057 | 0.275 | 2.06e-01 |
| node_pheno66 | childhood_BMI | Weighted mode | 0.097 | 0.078 | -0.056 | 0.251 | 2.23e-01 |
| node_pheno67 | childhood_BMI | MR Egger | 0.212 | 0.101 | 0.014 | 0.410 | 4.37e-02 |
| node_pheno67 | childhood_BMI | Weighted median | 0.044 | 0.039 | -0.032 | 0.120 | 2.56e-01 |
| node_pheno67 | childhood_BMI | Simple mode | 0.069 | 0.077 | -0.082 | 0.221 | 3.77e-01 |
| node_pheno67 | childhood_BMI | Weighted mode | 0.018 | 0.081 | -0.141 | 0.177 | 8.26e-01 |
| node_pheno68 | childhood_BMI | MR Egger | 0.028 | 0.087 | -0.143 | 0.200 | 7.50e-01 |
| node_pheno68 | childhood_BMI | Weighted median | 0.012 | 0.040 | -0.065 | 0.090 | 7.60e-01 |
| node_pheno68 | childhood_BMI | Simple mode | 0.019 | 0.084 | -0.146 | 0.185 | 8.19e-01 |
| node_pheno68 | childhood_BMI | Weighted mode | -0.035 | 0.083 | -0.197 | 0.127 | 6.76e-01 |
| node_pheno69 | childhood_BMI | MR Egger | 0.086 | 0.108 | -0.125 | 0.297 | 4.28e-01 |
| node_pheno69 | childhood_BMI | Weighted median | 0.058 | 0.040 | -0.020 | 0.137 | 1.46e-01 |
| node_pheno69 | childhood_BMI | Simple mode | 0.059 | 0.073 | -0.084 | 0.203 | 4.24e-01 |
| node_pheno69 | childhood_BMI | Weighted mode | 0.049 | 0.060 | -0.069 | 0.168 | 4.20e-01 |
| node_pheno7 | childhood_BMI | MR Egger | 0.027 | 0.093 | -0.156 | 0.209 | 7.76e-01 |
| node_pheno7 | childhood_BMI | Weighted median | -0.017 | 0.043 | -0.101 | 0.067 | 6.86e-01 |
| node_pheno7 | childhood_BMI | Simple mode | -0.098 | 0.105 | -0.304 | 0.108 | 3.57e-01 |
| node_pheno7 | childhood_BMI | Weighted mode | -0.113 | 0.089 | -0.287 | 0.062 | 2.14e-01 |
| node_pheno70 | childhood_BMI | MR Egger | 0.050 | 0.092 | -0.131 | 0.231 | 5.92e-01 |
| node_pheno70 | childhood_BMI | Weighted median | 0.041 | 0.037 | -0.032 | 0.114 | 2.67e-01 |
| node_pheno70 | childhood_BMI | Simple mode | 0.080 | 0.068 | -0.054 | 0.214 | 2.51e-01 |
| node_pheno70 | childhood_BMI | Weighted mode | 0.080 | 0.063 | -0.044 | 0.203 | 2.14e-01 |
| node_pheno71 | childhood_BMI | MR Egger | 0.133 | 0.082 | -0.027 | 0.293 | 1.14e-01 |
| node_pheno71 | childhood_BMI | Weighted median | 0.007 | 0.038 | -0.068 | 0.081 | 8.63e-01 |
| node_pheno71 | childhood_BMI | Simple mode | 0.021 | 0.079 | -0.134 | 0.177 | 7.91e-01 |
| node_pheno71 | childhood_BMI | Weighted mode | 0.019 | 0.079 | -0.135 | 0.173 | 8.12e-01 |
| node_pheno72 | childhood_BMI | MR Egger | 0.055 | 0.077 | -0.096 | 0.206 | 4.82e-01 |
| node_pheno72 | childhood_BMI | Weighted median | 0.058 | 0.032 | -0.005 | 0.121 | 7.16e-02 |
| node_pheno72 | childhood_BMI | Simple mode | 0.090 | 0.061 | -0.030 | 0.210 | 1.51e-01 |
| node_pheno72 | childhood_BMI | Weighted mode | 0.037 | 0.052 | -0.064 | 0.138 | 4.76e-01 |
| node_pheno73 | childhood_BMI | MR Egger | 0.084 | 0.075 | -0.064 | 0.232 | 2.73e-01 |
| node_pheno73 | childhood_BMI | Weighted median | 0.040 | 0.034 | -0.027 | 0.108 | 2.39e-01 |
| node_pheno73 | childhood_BMI | Simple mode | 0.059 | 0.061 | -0.061 | 0.179 | 3.43e-01 |
| node_pheno73 | childhood_BMI | Weighted mode | 0.039 | 0.053 | -0.065 | 0.142 | 4.70e-01 |
| node_pheno74 | childhood_BMI | MR Egger | 0.036 | 0.086 | -0.133 | 0.205 | 6.76e-01 |
| node_pheno74 | childhood_BMI | Weighted median | 0.042 | 0.039 | -0.035 | 0.119 | 2.85e-01 |
| node_pheno74 | childhood_BMI | Simple mode | 0.102 | 0.072 | -0.038 | 0.243 | 1.63e-01 |
| node_pheno74 | childhood_BMI | Weighted mode | 0.038 | 0.062 | -0.084 | 0.160 | 5.42e-01 |
| node_pheno75 | childhood_BMI | MR Egger | 0.056 | 0.090 | -0.120 | 0.232 | 5.39e-01 |
| node_pheno75 | childhood_BMI | Weighted median | 0.006 | 0.038 | -0.069 | 0.082 | 8.67e-01 |
| node_pheno75 | childhood_BMI | Simple mode | -0.075 | 0.082 | -0.237 | 0.086 | 3.66e-01 |
| node_pheno75 | childhood_BMI | Weighted mode | -0.034 | 0.065 | -0.160 | 0.093 | 6.04e-01 |
| node_pheno76 | childhood_BMI | MR Egger | -0.042 | 0.082 | -0.203 | 0.118 | 6.09e-01 |
| node_pheno76 | childhood_BMI | Weighted median | 0.031 | 0.035 | -0.038 | 0.100 | 3.81e-01 |
| node_pheno76 | childhood_BMI | Simple mode | 0.148 | 0.090 | -0.029 | 0.325 | 1.10e-01 |
| node_pheno76 | childhood_BMI | Weighted mode | -0.063 | 0.066 | -0.192 | 0.065 | 3.43e-01 |
| node_pheno8 | childhood_BMI | MR Egger | 0.140 | 0.101 | -0.057 | 0.338 | 1.73e-01 |
| node_pheno8 | childhood_BMI | Weighted median | -0.004 | 0.039 | -0.081 | 0.074 | 9.29e-01 |
| node_pheno8 | childhood_BMI | Simple mode | 0.002 | 0.090 | -0.174 | 0.178 | 9.79e-01 |
| node_pheno8 | childhood_BMI | Weighted mode | -0.017 | 0.075 | -0.164 | 0.130 | 8.19e-01 |
| node_pheno9 | childhood_BMI | MR Egger | 0.195 | 0.078 | 0.042 | 0.348 | 1.77e-02 |
| node_pheno9 | childhood_BMI | Weighted median | 0.010 | 0.039 | -0.067 | 0.087 | 8.01e-01 |
| node_pheno9 | childhood_BMI | Simple mode | 0.025 | 0.065 | -0.103 | 0.153 | 7.03e-01 |
| node_pheno9 | childhood_BMI | Weighted mode | 0.018 | 0.055 | -0.089 | 0.125 | 7.44e-01 |
| edge_pheno101 | adulthood_BMI | MR Egger | -0.044 | 0.068 | -0.176 | 0.089 | 5.17e-01 |
| edge_pheno101 | adulthood_BMI | Weighted median | -0.011 | 0.040 | -0.089 | 0.067 | 7.88e-01 |
| edge_pheno101 | adulthood_BMI | Simple mode | 0.038 | 0.135 | -0.225 | 0.302 | 7.77e-01 |
| edge_pheno101 | adulthood_BMI | Weighted mode | -0.020 | 0.090 | -0.196 | 0.157 | 8.28e-01 |
| edge_pheno1013 | adulthood_BMI | MR Egger | -0.070 | 0.067 | -0.201 | 0.060 | 2.93e-01 |
| edge_pheno1013 | adulthood_BMI | Weighted median | -0.040 | 0.039 | -0.117 | 0.037 | 3.11e-01 |
| edge_pheno1013 | adulthood_BMI | Simple mode | -0.054 | 0.131 | -0.310 | 0.201 | 6.77e-01 |
| edge_pheno1013 | adulthood_BMI | Weighted mode | 0.003 | 0.086 | -0.167 | 0.172 | 9.75e-01 |
| edge_pheno102 | adulthood_BMI | MR Egger | 0.009 | 0.069 | -0.127 | 0.145 | 8.94e-01 |
| edge_pheno102 | adulthood_BMI | Weighted median | -0.012 | 0.040 | -0.090 | 0.067 | 7.75e-01 |
| edge_pheno102 | adulthood_BMI | Simple mode | -0.096 | 0.120 | -0.331 | 0.139 | 4.25e-01 |
| edge_pheno102 | adulthood_BMI | Weighted mode | -0.011 | 0.081 | -0.170 | 0.147 | 8.87e-01 |
| edge_pheno1020 | adulthood_BMI | MR Egger | 0.057 | 0.065 | -0.070 | 0.184 | 3.80e-01 |
| edge_pheno1020 | adulthood_BMI | Weighted median | 0.093 | 0.040 | 0.015 | 0.172 | 2.00e-02 |
| edge_pheno1020 | adulthood_BMI | Simple mode | 0.132 | 0.135 | -0.133 | 0.396 | 3.29e-01 |
| edge_pheno1020 | adulthood_BMI | Weighted mode | 0.146 | 0.094 | -0.038 | 0.329 | 1.21e-01 |
| edge_pheno1022 | adulthood_BMI | MR Egger | -0.049 | 0.065 | -0.177 | 0.079 | 4.52e-01 |
| edge_pheno1022 | adulthood_BMI | Weighted median | 0.007 | 0.040 | -0.072 | 0.086 | 8.54e-01 |
| edge_pheno1022 | adulthood_BMI | Simple mode | 0.004 | 0.126 | -0.243 | 0.251 | 9.74e-01 |
| edge_pheno1022 | adulthood_BMI | Weighted mode | -0.032 | 0.091 | -0.211 | 0.147 | 7.24e-01 |
| edge_pheno103 | adulthood_BMI | MR Egger | -0.061 | 0.067 | -0.193 | 0.070 | 3.62e-01 |
| edge_pheno103 | adulthood_BMI | Weighted median | -0.054 | 0.038 | -0.128 | 0.020 | 1.55e-01 |
| edge_pheno103 | adulthood_BMI | Simple mode | -0.045 | 0.119 | -0.279 | 0.188 | 7.03e-01 |
| edge_pheno103 | adulthood_BMI | Weighted mode | -0.083 | 0.073 | -0.226 | 0.060 | 2.56e-01 |
| edge_pheno1041 | adulthood_BMI | MR Egger | -0.006 | 0.068 | -0.139 | 0.127 | 9.26e-01 |
| edge_pheno1041 | adulthood_BMI | Weighted median | 0.020 | 0.039 | -0.056 | 0.097 | 6.05e-01 |
| edge_pheno1041 | adulthood_BMI | Simple mode | 0.001 | 0.132 | -0.257 | 0.260 | 9.92e-01 |
| edge_pheno1041 | adulthood_BMI | Weighted mode | 0.013 | 0.084 | -0.152 | 0.179 | 8.75e-01 |
| edge_pheno1059 | adulthood_BMI | MR Egger | 0.099 | 0.070 | -0.037 | 0.236 | 1.54e-01 |
| edge_pheno1059 | adulthood_BMI | Weighted median | 0.001 | 0.040 | -0.076 | 0.079 | 9.73e-01 |
| edge_pheno1059 | adulthood_BMI | Simple mode | -0.084 | 0.136 | -0.350 | 0.183 | 5.39e-01 |
| edge_pheno1059 | adulthood_BMI | Weighted mode | 0.049 | 0.082 | -0.112 | 0.211 | 5.47e-01 |
| edge_pheno1122 | adulthood_BMI | MR Egger | -0.120 | 0.079 | -0.275 | 0.034 | 1.27e-01 |
| edge_pheno1122 | adulthood_BMI | Weighted median | 0.001 | 0.042 | -0.080 | 0.083 | 9.77e-01 |
| edge_pheno1122 | adulthood_BMI | Simple mode | 0.217 | 0.138 | -0.052 | 0.487 | 1.15e-01 |
| edge_pheno1122 | adulthood_BMI | Weighted mode | 0.028 | 0.082 | -0.133 | 0.189 | 7.31e-01 |
| edge_pheno1126 | adulthood_BMI | MR Egger | -0.091 | 0.067 | -0.223 | 0.041 | 1.78e-01 |
| edge_pheno1126 | adulthood_BMI | Weighted median | -0.017 | 0.039 | -0.093 | 0.059 | 6.61e-01 |
| edge_pheno1126 | adulthood_BMI | Simple mode | 0.050 | 0.137 | -0.218 | 0.318 | 7.14e-01 |
| edge_pheno1126 | adulthood_BMI | Weighted mode | -0.062 | 0.086 | -0.230 | 0.105 | 4.67e-01 |
| edge_pheno1134 | adulthood_BMI | MR Egger | -0.037 | 0.073 | -0.180 | 0.106 | 6.11e-01 |
| edge_pheno1134 | adulthood_BMI | Weighted median | -0.014 | 0.040 | -0.092 | 0.064 | 7.33e-01 |
| edge_pheno1134 | adulthood_BMI | Simple mode | -0.103 | 0.131 | -0.361 | 0.154 | 4.32e-01 |
| edge_pheno1134 | adulthood_BMI | Weighted mode | -0.065 | 0.081 | -0.225 | 0.095 | 4.24e-01 |
| edge_pheno1137 | adulthood_BMI | MR Egger | -0.032 | 0.074 | -0.177 | 0.114 | 6.68e-01 |
| edge_pheno1137 | adulthood_BMI | Weighted median | -0.037 | 0.041 | -0.117 | 0.044 | 3.72e-01 |
| edge_pheno1137 | adulthood_BMI | Simple mode | 0.091 | 0.139 | -0.182 | 0.363 | 5.13e-01 |
| edge_pheno1137 | adulthood_BMI | Weighted mode | -0.112 | 0.083 | -0.275 | 0.051 | 1.79e-01 |
| edge_pheno1141 | adulthood_BMI | MR Egger | -0.101 | 0.072 | -0.242 | 0.041 | 1.65e-01 |
| edge_pheno1141 | adulthood_BMI | Weighted median | -0.051 | 0.039 | -0.128 | 0.025 | 1.88e-01 |
| edge_pheno1141 | adulthood_BMI | Simple mode | 0.017 | 0.129 | -0.235 | 0.269 | 8.97e-01 |
| edge_pheno1141 | adulthood_BMI | Weighted mode | -0.072 | 0.081 | -0.230 | 0.085 | 3.68e-01 |
| edge_pheno1142 | adulthood_BMI | MR Egger | -0.092 | 0.070 | -0.229 | 0.046 | 1.91e-01 |
| edge_pheno1142 | adulthood_BMI | Weighted median | -0.099 | 0.041 | -0.179 | -0.019 | 1.58e-02 |
| edge_pheno1142 | adulthood_BMI | Simple mode | -0.194 | 0.140 | -0.469 | 0.081 | 1.68e-01 |
| edge_pheno1142 | adulthood_BMI | Weighted mode | -0.204 | 0.092 | -0.385 | -0.024 | 2.69e-02 |
| edge_pheno1161 | adulthood_BMI | MR Egger | -0.016 | 0.073 | -0.158 | 0.126 | 8.26e-01 |
| edge_pheno1161 | adulthood_BMI | Weighted median | 0.048 | 0.038 | -0.027 | 0.123 | 2.11e-01 |
| edge_pheno1161 | adulthood_BMI | Simple mode | 0.132 | 0.115 | -0.093 | 0.357 | 2.50e-01 |
| edge_pheno1161 | adulthood_BMI | Weighted mode | 0.081 | 0.074 | -0.063 | 0.226 | 2.69e-01 |
| edge_pheno1167 | adulthood_BMI | MR Egger | -0.028 | 0.073 | -0.171 | 0.114 | 6.97e-01 |
| edge_pheno1167 | adulthood_BMI | Weighted median | -0.004 | 0.039 | -0.081 | 0.073 | 9.18e-01 |
| edge_pheno1167 | adulthood_BMI | Simple mode | -0.150 | 0.127 | -0.399 | 0.099 | 2.37e-01 |
| edge_pheno1167 | adulthood_BMI | Weighted mode | -0.081 | 0.106 | -0.290 | 0.128 | 4.48e-01 |
| edge_pheno1171 | adulthood_BMI | MR Egger | 0.052 | 0.072 | -0.088 | 0.193 | 4.66e-01 |
| edge_pheno1171 | adulthood_BMI | Weighted median | 0.041 | 0.040 | -0.036 | 0.119 | 2.95e-01 |
| edge_pheno1171 | adulthood_BMI | Simple mode | 0.100 | 0.126 | -0.146 | 0.347 | 4.25e-01 |
| edge_pheno1171 | adulthood_BMI | Weighted mode | 0.100 | 0.079 | -0.055 | 0.256 | 2.07e-01 |
| edge_pheno1175 | adulthood_BMI | MR Egger | 0.062 | 0.066 | -0.066 | 0.191 | 3.43e-01 |
| edge_pheno1175 | adulthood_BMI | Weighted median | 0.034 | 0.040 | -0.044 | 0.112 | 3.97e-01 |
| edge_pheno1175 | adulthood_BMI | Simple mode | -0.001 | 0.121 | -0.238 | 0.237 | 9.95e-01 |
| edge_pheno1175 | adulthood_BMI | Weighted mode | 0.069 | 0.071 | -0.071 | 0.208 | 3.34e-01 |
| edge_pheno1183 | adulthood_BMI | MR Egger | 0.022 | 0.070 | -0.116 | 0.160 | 7.52e-01 |
| edge_pheno1183 | adulthood_BMI | Weighted median | 0.011 | 0.040 | -0.067 | 0.088 | 7.83e-01 |
| edge_pheno1183 | adulthood_BMI | Simple mode | -0.049 | 0.127 | -0.299 | 0.200 | 6.97e-01 |
| edge_pheno1183 | adulthood_BMI | Weighted mode | 0.011 | 0.091 | -0.167 | 0.189 | 9.04e-01 |
| edge_pheno1184 | adulthood_BMI | MR Egger | 0.059 | 0.070 | -0.078 | 0.196 | 3.98e-01 |
| edge_pheno1184 | adulthood_BMI | Weighted median | 0.024 | 0.040 | -0.054 | 0.101 | 5.53e-01 |
| edge_pheno1184 | adulthood_BMI | Simple mode | 0.020 | 0.120 | -0.216 | 0.255 | 8.71e-01 |
| edge_pheno1184 | adulthood_BMI | Weighted mode | 0.031 | 0.078 | -0.121 | 0.184 | 6.88e-01 |
| edge_pheno1189 | adulthood_BMI | MR Egger | -0.030 | 0.071 | -0.169 | 0.109 | 6.71e-01 |
| edge_pheno1189 | adulthood_BMI | Weighted median | -0.009 | 0.041 | -0.089 | 0.071 | 8.27e-01 |
| edge_pheno1189 | adulthood_BMI | Simple mode | 0.025 | 0.142 | -0.252 | 0.303 | 8.59e-01 |
| edge_pheno1189 | adulthood_BMI | Weighted mode | -0.041 | 0.083 | -0.204 | 0.123 | 6.26e-01 |
| edge_pheno1205 | adulthood_BMI | MR Egger | 0.022 | 0.063 | -0.102 | 0.146 | 7.30e-01 |
| edge_pheno1205 | adulthood_BMI | Weighted median | 0.001 | 0.038 | -0.073 | 0.076 | 9.73e-01 |
| edge_pheno1205 | adulthood_BMI | Simple mode | 0.088 | 0.118 | -0.142 | 0.319 | 4.53e-01 |
| edge_pheno1205 | adulthood_BMI | Weighted mode | -0.024 | 0.084 | -0.188 | 0.140 | 7.75e-01 |
| edge_pheno1211 | adulthood_BMI | MR Egger | 0.028 | 0.068 | -0.106 | 0.161 | 6.85e-01 |
| edge_pheno1211 | adulthood_BMI | Weighted median | -0.010 | 0.039 | -0.086 | 0.067 | 8.01e-01 |
| edge_pheno1211 | adulthood_BMI | Simple mode | -0.097 | 0.143 | -0.377 | 0.183 | 4.96e-01 |
| edge_pheno1211 | adulthood_BMI | Weighted mode | -0.075 | 0.105 | -0.281 | 0.130 | 4.73e-01 |
| edge_pheno1221 | adulthood_BMI | MR Egger | -0.042 | 0.073 | -0.185 | 0.102 | 5.71e-01 |
| edge_pheno1221 | adulthood_BMI | Weighted median | -0.014 | 0.039 | -0.091 | 0.063 | 7.19e-01 |
| edge_pheno1221 | adulthood_BMI | Simple mode | -0.119 | 0.138 | -0.389 | 0.152 | 3.90e-01 |
| edge_pheno1221 | adulthood_BMI | Weighted mode | -0.068 | 0.088 | -0.241 | 0.104 | 4.37e-01 |
| edge_pheno1225 | adulthood_BMI | MR Egger | -0.022 | 0.070 | -0.159 | 0.114 | 7.47e-01 |
| edge_pheno1225 | adulthood_BMI | Weighted median | 0.011 | 0.041 | -0.069 | 0.092 | 7.82e-01 |
| edge_pheno1225 | adulthood_BMI | Simple mode | -0.077 | 0.132 | -0.336 | 0.182 | 5.61e-01 |
| edge_pheno1225 | adulthood_BMI | Weighted mode | -0.051 | 0.080 | -0.208 | 0.105 | 5.19e-01 |
| edge_pheno1250 | adulthood_BMI | MR Egger | 0.007 | 0.070 | -0.131 | 0.145 | 9.22e-01 |
| edge_pheno1250 | adulthood_BMI | Weighted median | -0.020 | 0.040 | -0.097 | 0.058 | 6.19e-01 |
| edge_pheno1250 | adulthood_BMI | Simple mode | -0.059 | 0.125 | -0.305 | 0.186 | 6.36e-01 |
| edge_pheno1250 | adulthood_BMI | Weighted mode | -0.046 | 0.082 | -0.206 | 0.114 | 5.74e-01 |
| edge_pheno1256 | adulthood_BMI | MR Egger | 0.113 | 0.069 | -0.023 | 0.248 | 1.04e-01 |
| edge_pheno1256 | adulthood_BMI | Weighted median | -0.019 | 0.038 | -0.094 | 0.056 | 6.20e-01 |
| edge_pheno1256 | adulthood_BMI | Simple mode | -0.061 | 0.151 | -0.357 | 0.234 | 6.84e-01 |
| edge_pheno1256 | adulthood_BMI | Weighted mode | -0.061 | 0.098 | -0.253 | 0.130 | 5.29e-01 |
| edge_pheno1257 | adulthood_BMI | MR Egger | -0.034 | 0.067 | -0.165 | 0.097 | 6.11e-01 |
| edge_pheno1257 | adulthood_BMI | Weighted median | 0.018 | 0.040 | -0.061 | 0.096 | 6.56e-01 |
| edge_pheno1257 | adulthood_BMI | Simple mode | 0.026 | 0.138 | -0.244 | 0.296 | 8.51e-01 |
| edge_pheno1257 | adulthood_BMI | Weighted mode | 0.038 | 0.091 | -0.141 | 0.217 | 6.79e-01 |
| edge_pheno1269 | adulthood_BMI | MR Egger | -0.109 | 0.071 | -0.247 | 0.029 | 1.22e-01 |
| edge_pheno1269 | adulthood_BMI | Weighted median | -0.069 | 0.039 | -0.144 | 0.007 | 7.41e-02 |
| edge_pheno1269 | adulthood_BMI | Simple mode | -0.008 | 0.124 | -0.252 | 0.235 | 9.46e-01 |
| edge_pheno1269 | adulthood_BMI | Weighted mode | -0.082 | 0.074 | -0.227 | 0.063 | 2.68e-01 |
| edge_pheno1270 | adulthood_BMI | MR Egger | 0.039 | 0.071 | -0.101 | 0.179 | 5.88e-01 |
| edge_pheno1270 | adulthood_BMI | Weighted median | -0.020 | 0.039 | -0.098 | 0.057 | 6.04e-01 |
| edge_pheno1270 | adulthood_BMI | Simple mode | -0.067 | 0.140 | -0.341 | 0.208 | 6.35e-01 |
| edge_pheno1270 | adulthood_BMI | Weighted mode | -0.105 | 0.102 | -0.304 | 0.095 | 3.05e-01 |
| edge_pheno1273 | adulthood_BMI | MR Egger | 0.067 | 0.069 | -0.068 | 0.201 | 3.34e-01 |
| edge_pheno1273 | adulthood_BMI | Weighted median | 0.055 | 0.040 | -0.023 | 0.133 | 1.64e-01 |
| edge_pheno1273 | adulthood_BMI | Simple mode | 0.055 | 0.122 | -0.184 | 0.295 | 6.51e-01 |
| edge_pheno1273 | adulthood_BMI | Weighted mode | 0.019 | 0.088 | -0.154 | 0.192 | 8.31e-01 |
| edge_pheno1276 | adulthood_BMI | MR Egger | 0.023 | 0.067 | -0.109 | 0.154 | 7.37e-01 |
| edge_pheno1276 | adulthood_BMI | Weighted median | -0.037 | 0.038 | -0.111 | 0.037 | 3.32e-01 |
| edge_pheno1276 | adulthood_BMI | Simple mode | -0.069 | 0.115 | -0.295 | 0.157 | 5.49e-01 |
| edge_pheno1276 | adulthood_BMI | Weighted mode | -0.032 | 0.069 | -0.167 | 0.104 | 6.44e-01 |
| edge_pheno1293 | adulthood_BMI | MR Egger | -0.123 | 0.071 | -0.261 | 0.016 | 8.37e-02 |
| edge_pheno1293 | adulthood_BMI | Weighted median | 0.047 | 0.040 | -0.032 | 0.126 | 2.47e-01 |
| edge_pheno1293 | adulthood_BMI | Simple mode | 0.078 | 0.123 | -0.163 | 0.320 | 5.24e-01 |
| edge_pheno1293 | adulthood_BMI | Weighted mode | 0.053 | 0.079 | -0.101 | 0.208 | 4.98e-01 |
| edge_pheno1296 | adulthood_BMI | MR Egger | -0.005 | 0.069 | -0.141 | 0.130 | 9.37e-01 |
| edge_pheno1296 | adulthood_BMI | Weighted median | -0.011 | 0.039 | -0.088 | 0.065 | 7.69e-01 |
| edge_pheno1296 | adulthood_BMI | Simple mode | -0.044 | 0.143 | -0.325 | 0.236 | 7.58e-01 |
| edge_pheno1296 | adulthood_BMI | Weighted mode | -0.086 | 0.086 | -0.254 | 0.081 | 3.13e-01 |
| edge_pheno1300 | adulthood_BMI | MR Egger | 0.155 | 0.069 | 0.019 | 0.290 | 2.54e-02 |
| edge_pheno1300 | adulthood_BMI | Weighted median | 0.073 | 0.040 | -0.004 | 0.151 | 6.46e-02 |
| edge_pheno1300 | adulthood_BMI | Simple mode | -0.010 | 0.133 | -0.271 | 0.251 | 9.40e-01 |
| edge_pheno1300 | adulthood_BMI | Weighted mode | 0.108 | 0.085 | -0.059 | 0.275 | 2.04e-01 |
| edge_pheno1301 | adulthood_BMI | MR Egger | -0.050 | 0.071 | -0.189 | 0.090 | 4.85e-01 |
| edge_pheno1301 | adulthood_BMI | Weighted median | -0.001 | 0.040 | -0.079 | 0.076 | 9.71e-01 |
| edge_pheno1301 | adulthood_BMI | Simple mode | 0.217 | 0.144 | -0.066 | 0.499 | 1.34e-01 |
| edge_pheno1301 | adulthood_BMI | Weighted mode | -0.169 | 0.093 | -0.351 | 0.014 | 7.05e-02 |
| edge_pheno1302 | adulthood_BMI | MR Egger | 0.078 | 0.071 | -0.062 | 0.218 | 2.75e-01 |
| edge_pheno1302 | adulthood_BMI | Weighted median | 0.018 | 0.040 | -0.060 | 0.097 | 6.46e-01 |
| edge_pheno1302 | adulthood_BMI | Simple mode | 0.050 | 0.126 | -0.196 | 0.297 | 6.89e-01 |
| edge_pheno1302 | adulthood_BMI | Weighted mode | 0.027 | 0.080 | -0.130 | 0.183 | 7.40e-01 |
| edge_pheno1309 | adulthood_BMI | MR Egger | -0.150 | 0.073 | -0.292 | -0.007 | 4.03e-02 |
| edge_pheno1309 | adulthood_BMI | Weighted median | 0.010 | 0.041 | -0.070 | 0.090 | 8.06e-01 |
| edge_pheno1309 | adulthood_BMI | Simple mode | 0.111 | 0.139 | -0.162 | 0.384 | 4.25e-01 |
| edge_pheno1309 | adulthood_BMI | Weighted mode | 0.006 | 0.086 | -0.162 | 0.174 | 9.47e-01 |
| edge_pheno1311 | adulthood_BMI | MR Egger | 0.129 | 0.080 | -0.028 | 0.286 | 1.08e-01 |
| edge_pheno1311 | adulthood_BMI | Weighted median | 0.067 | 0.047 | -0.024 | 0.159 | 1.50e-01 |
| edge_pheno1311 | adulthood_BMI | Simple mode | 0.156 | 0.140 | -0.119 | 0.430 | 2.67e-01 |
| edge_pheno1311 | adulthood_BMI | Weighted mode | 0.108 | 0.095 | -0.079 | 0.294 | 2.59e-01 |
| edge_pheno1317 | adulthood_BMI | MR Egger | -0.079 | 0.086 | -0.247 | 0.089 | 3.58e-01 |
| edge_pheno1317 | adulthood_BMI | Weighted median | -0.062 | 0.048 | -0.157 | 0.033 | 2.00e-01 |
| edge_pheno1317 | adulthood_BMI | Simple mode | -0.176 | 0.170 | -0.511 | 0.158 | 3.01e-01 |
| edge_pheno1317 | adulthood_BMI | Weighted mode | -0.227 | 0.133 | -0.488 | 0.034 | 8.91e-02 |
| edge_pheno1319 | adulthood_BMI | MR Egger | 0.185 | 0.071 | 0.046 | 0.324 | 9.34e-03 |
| edge_pheno1319 | adulthood_BMI | Weighted median | 0.068 | 0.040 | -0.012 | 0.147 | 9.40e-02 |
| edge_pheno1319 | adulthood_BMI | Simple mode | 0.033 | 0.137 | -0.236 | 0.301 | 8.12e-01 |
| edge_pheno1319 | adulthood_BMI | Weighted mode | 0.071 | 0.104 | -0.133 | 0.274 | 4.97e-01 |
| edge_pheno132 | adulthood_BMI | MR Egger | -0.034 | 0.067 | -0.166 | 0.098 | 6.15e-01 |
| edge_pheno132 | adulthood_BMI | Weighted median | -0.020 | 0.041 | -0.099 | 0.060 | 6.26e-01 |
| edge_pheno132 | adulthood_BMI | Simple mode | -0.130 | 0.113 | -0.351 | 0.091 | 2.50e-01 |
| edge_pheno132 | adulthood_BMI | Weighted mode | -0.073 | 0.078 | -0.225 | 0.080 | 3.51e-01 |
| edge_pheno1322 | adulthood_BMI | MR Egger | 0.031 | 0.070 | -0.106 | 0.168 | 6.57e-01 |
| edge_pheno1322 | adulthood_BMI | Weighted median | 0.015 | 0.040 | -0.064 | 0.094 | 7.16e-01 |
| edge_pheno1322 | adulthood_BMI | Simple mode | -0.006 | 0.134 | -0.268 | 0.256 | 9.64e-01 |
| edge_pheno1322 | adulthood_BMI | Weighted mode | 0.065 | 0.074 | -0.080 | 0.210 | 3.80e-01 |
| edge_pheno1325 | adulthood_BMI | MR Egger | -0.053 | 0.068 | -0.186 | 0.081 | 4.40e-01 |
| edge_pheno1325 | adulthood_BMI | Weighted median | -0.021 | 0.040 | -0.100 | 0.057 | 5.93e-01 |
| edge_pheno1325 | adulthood_BMI | Simple mode | -0.074 | 0.149 | -0.366 | 0.219 | 6.21e-01 |
| edge_pheno1325 | adulthood_BMI | Weighted mode | -0.088 | 0.100 | -0.284 | 0.107 | 3.76e-01 |
| edge_pheno1328 | adulthood_BMI | MR Egger | -0.127 | 0.071 | -0.266 | 0.012 | 7.30e-02 |
| edge_pheno1328 | adulthood_BMI | Weighted median | 0.003 | 0.039 | -0.073 | 0.079 | 9.35e-01 |
| edge_pheno1328 | adulthood_BMI | Simple mode | -0.107 | 0.132 | -0.365 | 0.152 | 4.19e-01 |
| edge_pheno1328 | adulthood_BMI | Weighted mode | 0.074 | 0.097 | -0.116 | 0.264 | 4.44e-01 |
| edge_pheno135 | adulthood_BMI | MR Egger | -0.049 | 0.066 | -0.178 | 0.080 | 4.57e-01 |
| edge_pheno135 | adulthood_BMI | Weighted median | -0.003 | 0.041 | -0.083 | 0.077 | 9.43e-01 |
| edge_pheno135 | adulthood_BMI | Simple mode | -0.207 | 0.129 | -0.460 | 0.046 | 1.10e-01 |
| edge_pheno135 | adulthood_BMI | Weighted mode | -0.012 | 0.085 | -0.179 | 0.156 | 8.92e-01 |
| edge_pheno1359 | adulthood_BMI | MR Egger | 0.021 | 0.071 | -0.117 | 0.160 | 7.62e-01 |
| edge_pheno1359 | adulthood_BMI | Weighted median | -0.001 | 0.039 | -0.077 | 0.075 | 9.82e-01 |
| edge_pheno1359 | adulthood_BMI | Simple mode | 0.046 | 0.135 | -0.219 | 0.311 | 7.34e-01 |
| edge_pheno1359 | adulthood_BMI | Weighted mode | 0.059 | 0.108 | -0.154 | 0.271 | 5.89e-01 |
| edge_pheno1382 | adulthood_BMI | MR Egger | -0.050 | 0.074 | -0.195 | 0.095 | 4.96e-01 |
| edge_pheno1382 | adulthood_BMI | Weighted median | -0.008 | 0.040 | -0.087 | 0.070 | 8.36e-01 |
| edge_pheno1382 | adulthood_BMI | Simple mode | 0.130 | 0.141 | -0.146 | 0.405 | 3.57e-01 |
| edge_pheno1382 | adulthood_BMI | Weighted mode | 0.076 | 0.075 | -0.071 | 0.224 | 3.11e-01 |
| edge_pheno146 | adulthood_BMI | MR Egger | -0.089 | 0.065 | -0.216 | 0.039 | 1.73e-01 |
| edge_pheno146 | adulthood_BMI | Weighted median | 0.012 | 0.038 | -0.063 | 0.087 | 7.57e-01 |
| edge_pheno146 | adulthood_BMI | Simple mode | 0.077 | 0.132 | -0.182 | 0.336 | 5.61e-01 |
| edge_pheno146 | adulthood_BMI | Weighted mode | -0.027 | 0.091 | -0.206 | 0.152 | 7.69e-01 |
| edge_pheno147 | adulthood_BMI | MR Egger | -0.106 | 0.068 | -0.241 | 0.028 | 1.20e-01 |
| edge_pheno147 | adulthood_BMI | Weighted median | -0.035 | 0.039 | -0.113 | 0.042 | 3.70e-01 |
| edge_pheno147 | adulthood_BMI | Simple mode | -0.019 | 0.136 | -0.284 | 0.247 | 8.91e-01 |
| edge_pheno147 | adulthood_BMI | Weighted mode | -0.106 | 0.075 | -0.252 | 0.040 | 1.56e-01 |
| edge_pheno151 | adulthood_BMI | MR Egger | 0.051 | 0.066 | -0.079 | 0.181 | 4.42e-01 |
| edge_pheno151 | adulthood_BMI | Weighted median | 0.032 | 0.040 | -0.047 | 0.111 | 4.26e-01 |
| edge_pheno151 | adulthood_BMI | Simple mode | 0.029 | 0.124 | -0.215 | 0.273 | 8.15e-01 |
| edge_pheno151 | adulthood_BMI | Weighted mode | 0.029 | 0.083 | -0.134 | 0.192 | 7.27e-01 |
| edge_pheno1696 | adulthood_BMI | MR Egger | 0.038 | 0.069 | -0.097 | 0.173 | 5.80e-01 |
| edge_pheno1696 | adulthood_BMI | Weighted median | 0.013 | 0.037 | -0.059 | 0.085 | 7.20e-01 |
| edge_pheno1696 | adulthood_BMI | Simple mode | -0.012 | 0.123 | -0.253 | 0.229 | 9.21e-01 |
| edge_pheno1696 | adulthood_BMI | Weighted mode | 0.015 | 0.079 | -0.140 | 0.169 | 8.52e-01 |
| edge_pheno1697 | adulthood_BMI | MR Egger | 0.050 | 0.073 | -0.093 | 0.192 | 4.93e-01 |
| edge_pheno1697 | adulthood_BMI | Weighted median | -0.012 | 0.038 | -0.087 | 0.063 | 7.49e-01 |
| edge_pheno1697 | adulthood_BMI | Simple mode | -0.058 | 0.124 | -0.300 | 0.184 | 6.39e-01 |
| edge_pheno1697 | adulthood_BMI | Weighted mode | 0.004 | 0.073 | -0.140 | 0.147 | 9.62e-01 |
| edge_pheno1698 | adulthood_BMI | MR Egger | -0.120 | 0.080 | -0.278 | 0.037 | 1.35e-01 |
| edge_pheno1698 | adulthood_BMI | Weighted median | 0.054 | 0.041 | -0.027 | 0.135 | 1.94e-01 |
| edge_pheno1698 | adulthood_BMI | Simple mode | 0.065 | 0.127 | -0.185 | 0.314 | 6.12e-01 |
| edge_pheno1698 | adulthood_BMI | Weighted mode | 0.034 | 0.085 | -0.131 | 0.200 | 6.84e-01 |
| edge_pheno1699 | adulthood_BMI | MR Egger | -0.031 | 0.070 | -0.168 | 0.107 | 6.62e-01 |
| edge_pheno1699 | adulthood_BMI | Weighted median | -0.020 | 0.036 | -0.090 | 0.051 | 5.88e-01 |
| edge_pheno1699 | adulthood_BMI | Simple mode | -0.068 | 0.119 | -0.301 | 0.165 | 5.67e-01 |
| edge_pheno1699 | adulthood_BMI | Weighted mode | -0.018 | 0.080 | -0.175 | 0.138 | 8.18e-01 |
| edge_pheno1701 | adulthood_BMI | MR Egger | 0.030 | 0.081 | -0.129 | 0.189 | 7.12e-01 |
| edge_pheno1701 | adulthood_BMI | Weighted median | -0.013 | 0.040 | -0.092 | 0.066 | 7.45e-01 |
| edge_pheno1701 | adulthood_BMI | Simple mode | 0.038 | 0.152 | -0.259 | 0.336 | 8.01e-01 |
| edge_pheno1701 | adulthood_BMI | Weighted mode | 0.001 | 0.092 | -0.179 | 0.181 | 9.94e-01 |
| edge_pheno249 | adulthood_BMI | MR Egger | 0.068 | 0.068 | -0.066 | 0.202 | 3.21e-01 |
| edge_pheno249 | adulthood_BMI | Weighted median | 0.008 | 0.040 | -0.070 | 0.085 | 8.44e-01 |
| edge_pheno249 | adulthood_BMI | Simple mode | -0.042 | 0.139 | -0.315 | 0.231 | 7.65e-01 |
| edge_pheno249 | adulthood_BMI | Weighted mode | 0.011 | 0.093 | -0.172 | 0.194 | 9.06e-01 |
| edge_pheno253 | adulthood_BMI | MR Egger | 0.157 | 0.069 | 0.022 | 0.292 | 2.27e-02 |
| edge_pheno253 | adulthood_BMI | Weighted median | 0.036 | 0.038 | -0.038 | 0.111 | 3.43e-01 |
| edge_pheno253 | adulthood_BMI | Simple mode | 0.053 | 0.135 | -0.211 | 0.318 | 6.93e-01 |
| edge_pheno253 | adulthood_BMI | Weighted mode | 0.087 | 0.084 | -0.078 | 0.253 | 3.00e-01 |
| edge_pheno262 | adulthood_BMI | MR Egger | -0.071 | 0.067 | -0.202 | 0.061 | 2.92e-01 |
| edge_pheno262 | adulthood_BMI | Weighted median | -0.027 | 0.039 | -0.103 | 0.049 | 4.90e-01 |
| edge_pheno262 | adulthood_BMI | Simple mode | -0.132 | 0.142 | -0.411 | 0.148 | 3.56e-01 |
| edge_pheno262 | adulthood_BMI | Weighted mode | -0.046 | 0.099 | -0.239 | 0.147 | 6.42e-01 |
| edge_pheno286 | adulthood_BMI | MR Egger | -0.115 | 0.066 | -0.244 | 0.014 | 7.99e-02 |
| edge_pheno286 | adulthood_BMI | Weighted median | -0.038 | 0.039 | -0.113 | 0.038 | 3.29e-01 |
| edge_pheno286 | adulthood_BMI | Simple mode | 0.003 | 0.124 | -0.240 | 0.246 | 9.79e-01 |
| edge_pheno286 | adulthood_BMI | Weighted mode | -0.071 | 0.081 | -0.229 | 0.087 | 3.79e-01 |
| edge_pheno288 | adulthood_BMI | MR Egger | 0.010 | 0.065 | -0.117 | 0.137 | 8.76e-01 |
| edge_pheno288 | adulthood_BMI | Weighted median | -0.037 | 0.038 | -0.111 | 0.038 | 3.32e-01 |
| edge_pheno288 | adulthood_BMI | Simple mode | -0.130 | 0.133 | -0.390 | 0.131 | 3.29e-01 |
| edge_pheno288 | adulthood_BMI | Weighted mode | -0.059 | 0.082 | -0.220 | 0.101 | 4.69e-01 |
| edge_pheno303 | adulthood_BMI | MR Egger | -0.068 | 0.068 | -0.201 | 0.065 | 3.17e-01 |
| edge_pheno303 | adulthood_BMI | Weighted median | -0.032 | 0.040 | -0.111 | 0.047 | 4.27e-01 |
| edge_pheno303 | adulthood_BMI | Simple mode | -0.017 | 0.132 | -0.275 | 0.241 | 8.99e-01 |
| edge_pheno303 | adulthood_BMI | Weighted mode | -0.050 | 0.077 | -0.201 | 0.101 | 5.17e-01 |
| edge_pheno389 | adulthood_BMI | MR Egger | -0.112 | 0.070 | -0.249 | 0.025 | 1.09e-01 |
| edge_pheno389 | adulthood_BMI | Weighted median | -0.019 | 0.040 | -0.097 | 0.060 | 6.38e-01 |
| edge_pheno389 | adulthood_BMI | Simple mode | -0.140 | 0.138 | -0.410 | 0.131 | 3.12e-01 |
| edge_pheno389 | adulthood_BMI | Weighted mode | -0.062 | 0.077 | -0.214 | 0.090 | 4.25e-01 |
| edge_pheno405 | adulthood_BMI | MR Egger | 0.115 | 0.071 | -0.023 | 0.254 | 1.03e-01 |
| edge_pheno405 | adulthood_BMI | Weighted median | 0.018 | 0.040 | -0.061 | 0.097 | 6.51e-01 |
| edge_pheno405 | adulthood_BMI | Simple mode | 0.052 | 0.135 | -0.213 | 0.317 | 7.00e-01 |
| edge_pheno405 | adulthood_BMI | Weighted mode | 0.052 | 0.112 | -0.168 | 0.273 | 6.43e-01 |
| edge_pheno447 | adulthood_BMI | MR Egger | 0.109 | 0.185 | -0.253 | 0.472 | 5.58e-01 |
| edge_pheno447 | adulthood_BMI | Weighted median | 0.003 | 0.118 | -0.228 | 0.235 | 9.79e-01 |
| edge_pheno447 | adulthood_BMI | Simple mode | -0.450 | 0.268 | -0.976 | 0.075 | 9.93e-02 |
| edge_pheno447 | adulthood_BMI | Weighted mode | 0.044 | 0.166 | -0.282 | 0.370 | 7.93e-01 |
| edge_pheno449 | adulthood_BMI | MR Egger | -0.206 | 0.206 | -0.608 | 0.197 | 3.23e-01 |
| edge_pheno449 | adulthood_BMI | Weighted median | -0.060 | 0.118 | -0.291 | 0.171 | 6.11e-01 |
| edge_pheno449 | adulthood_BMI | Simple mode | 0.196 | 0.243 | -0.280 | 0.671 | 4.24e-01 |
| edge_pheno449 | adulthood_BMI | Weighted mode | 0.019 | 0.173 | -0.321 | 0.358 | 9.15e-01 |
| edge_pheno460 | adulthood_BMI | MR Egger | 0.093 | 0.068 | -0.040 | 0.226 | 1.69e-01 |
| edge_pheno460 | adulthood_BMI | Weighted median | -0.001 | 0.040 | -0.079 | 0.077 | 9.79e-01 |
| edge_pheno460 | adulthood_BMI | Simple mode | -0.028 | 0.126 | -0.275 | 0.219 | 8.24e-01 |
| edge_pheno460 | adulthood_BMI | Weighted mode | -0.005 | 0.087 | -0.176 | 0.166 | 9.54e-01 |
| edge_pheno461 | adulthood_BMI | MR Egger | -0.056 | 0.066 | -0.185 | 0.073 | 3.98e-01 |
| edge_pheno461 | adulthood_BMI | Weighted median | -0.063 | 0.040 | -0.142 | 0.016 | 1.18e-01 |
| edge_pheno461 | adulthood_BMI | Simple mode | -0.128 | 0.122 | -0.368 | 0.112 | 2.96e-01 |
| edge_pheno461 | adulthood_BMI | Weighted mode | -0.073 | 0.081 | -0.232 | 0.087 | 3.72e-01 |
| edge_pheno491 | adulthood_BMI | MR Egger | -0.004 | 0.071 | -0.144 | 0.135 | 9.50e-01 |
| edge_pheno491 | adulthood_BMI | Weighted median | 0.031 | 0.038 | -0.045 | 0.106 | 4.25e-01 |
| edge_pheno491 | adulthood_BMI | Simple mode | 0.078 | 0.144 | -0.204 | 0.359 | 5.88e-01 |
| edge_pheno491 | adulthood_BMI | Weighted mode | 0.102 | 0.104 | -0.101 | 0.305 | 3.25e-01 |
| edge_pheno537 | adulthood_BMI | MR Egger | -0.020 | 0.071 | -0.160 | 0.120 | 7.79e-01 |
| edge_pheno537 | adulthood_BMI | Weighted median | -0.022 | 0.038 | -0.096 | 0.053 | 5.64e-01 |
| edge_pheno537 | adulthood_BMI | Simple mode | -0.064 | 0.117 | -0.294 | 0.165 | 5.84e-01 |
| edge_pheno537 | adulthood_BMI | Weighted mode | -0.091 | 0.076 | -0.239 | 0.058 | 2.31e-01 |
| edge_pheno55 | adulthood_BMI | MR Egger | -0.038 | 0.067 | -0.169 | 0.092 | 5.66e-01 |
| edge_pheno55 | adulthood_BMI | Weighted median | 0.005 | 0.039 | -0.072 | 0.082 | 8.99e-01 |
| edge_pheno55 | adulthood_BMI | Simple mode | 0.020 | 0.129 | -0.232 | 0.273 | 8.74e-01 |
| edge_pheno55 | adulthood_BMI | Weighted mode | 0.020 | 0.085 | -0.146 | 0.187 | 8.10e-01 |
| edge_pheno558 | adulthood_BMI | MR Egger | -0.084 | 0.067 | -0.214 | 0.047 | 2.09e-01 |
| edge_pheno558 | adulthood_BMI | Weighted median | -0.050 | 0.037 | -0.122 | 0.023 | 1.79e-01 |
| edge_pheno558 | adulthood_BMI | Simple mode | 0.124 | 0.127 | -0.125 | 0.374 | 3.29e-01 |
| edge_pheno558 | adulthood_BMI | Weighted mode | -0.127 | 0.097 | -0.317 | 0.064 | 1.93e-01 |
| edge_pheno574 | adulthood_BMI | MR Egger | -0.150 | 0.066 | -0.279 | -0.021 | 2.30e-02 |
| edge_pheno574 | adulthood_BMI | Weighted median | -0.046 | 0.038 | -0.121 | 0.029 | 2.29e-01 |
| edge_pheno574 | adulthood_BMI | Simple mode | -0.103 | 0.137 | -0.371 | 0.166 | 4.55e-01 |
| edge_pheno574 | adulthood_BMI | Weighted mode | -0.115 | 0.082 | -0.276 | 0.046 | 1.61e-01 |
| edge_pheno58 | adulthood_BMI | MR Egger | 0.030 | 0.066 | -0.099 | 0.160 | 6.46e-01 |
| edge_pheno58 | adulthood_BMI | Weighted median | 0.011 | 0.037 | -0.062 | 0.084 | 7.64e-01 |
| edge_pheno58 | adulthood_BMI | Simple mode | 0.070 | 0.124 | -0.172 | 0.312 | 5.70e-01 |
| edge_pheno58 | adulthood_BMI | Weighted mode | -0.042 | 0.078 | -0.194 | 0.110 | 5.90e-01 |
| edge_pheno590 | adulthood_BMI | MR Egger | 0.069 | 0.072 | -0.073 | 0.210 | 3.41e-01 |
| edge_pheno590 | adulthood_BMI | Weighted median | 0.019 | 0.039 | -0.058 | 0.097 | 6.24e-01 |
| edge_pheno590 | adulthood_BMI | Simple mode | 0.004 | 0.130 | -0.250 | 0.259 | 9.73e-01 |
| edge_pheno590 | adulthood_BMI | Weighted mode | 0.043 | 0.095 | -0.143 | 0.229 | 6.50e-01 |
| edge_pheno593 | adulthood_BMI | MR Egger | -0.138 | 0.071 | -0.278 | 0.001 | 5.29e-02 |
| edge_pheno593 | adulthood_BMI | Weighted median | -0.033 | 0.040 | -0.112 | 0.045 | 4.09e-01 |
| edge_pheno593 | adulthood_BMI | Simple mode | -0.083 | 0.137 | -0.352 | 0.185 | 5.44e-01 |
| edge_pheno593 | adulthood_BMI | Weighted mode | -0.045 | 0.091 | -0.224 | 0.135 | 6.25e-01 |
| edge_pheno597 | adulthood_BMI | MR Egger | -0.104 | 0.072 | -0.246 | 0.038 | 1.52e-01 |
| edge_pheno597 | adulthood_BMI | Weighted median | -0.006 | 0.040 | -0.084 | 0.073 | 8.84e-01 |
| edge_pheno597 | adulthood_BMI | Simple mode | 0.090 | 0.132 | -0.170 | 0.349 | 4.98e-01 |
| edge_pheno597 | adulthood_BMI | Weighted mode | 0.031 | 0.085 | -0.136 | 0.198 | 7.19e-01 |
| edge_pheno599 | adulthood_BMI | MR Egger | -0.097 | 0.069 | -0.233 | 0.039 | 1.62e-01 |
| edge_pheno599 | adulthood_BMI | Weighted median | -0.043 | 0.038 | -0.117 | 0.031 | 2.52e-01 |
| edge_pheno599 | adulthood_BMI | Simple mode | -0.046 | 0.132 | -0.303 | 0.212 | 7.29e-01 |
| edge_pheno599 | adulthood_BMI | Weighted mode | -0.058 | 0.089 | -0.232 | 0.117 | 5.19e-01 |
| edge_pheno601 | adulthood_BMI | MR Egger | -0.068 | 0.069 | -0.204 | 0.067 | 3.25e-01 |
| edge_pheno601 | adulthood_BMI | Weighted median | 0.014 | 0.041 | -0.066 | 0.094 | 7.27e-01 |
| edge_pheno601 | adulthood_BMI | Simple mode | 0.095 | 0.140 | -0.180 | 0.369 | 5.01e-01 |
| edge_pheno601 | adulthood_BMI | Weighted mode | 0.023 | 0.095 | -0.162 | 0.209 | 8.05e-01 |
| edge_pheno606 | adulthood_BMI | MR Egger | 0.041 | 0.068 | -0.093 | 0.174 | 5.50e-01 |
| edge_pheno606 | adulthood_BMI | Weighted median | 0.036 | 0.041 | -0.044 | 0.116 | 3.82e-01 |
| edge_pheno606 | adulthood_BMI | Simple mode | -0.039 | 0.141 | -0.315 | 0.237 | 7.81e-01 |
| edge_pheno606 | adulthood_BMI | Weighted mode | -0.039 | 0.088 | -0.211 | 0.133 | 6.57e-01 |
| edge_pheno609 | adulthood_BMI | MR Egger | -0.093 | 0.070 | -0.231 | 0.044 | 1.83e-01 |
| edge_pheno609 | adulthood_BMI | Weighted median | -0.014 | 0.040 | -0.092 | 0.064 | 7.18e-01 |
| edge_pheno609 | adulthood_BMI | Simple mode | -0.121 | 0.138 | -0.393 | 0.150 | 3.81e-01 |
| edge_pheno609 | adulthood_BMI | Weighted mode | -0.175 | 0.099 | -0.370 | 0.020 | 7.88e-02 |
| edge_pheno621 | adulthood_BMI | MR Egger | 0.050 | 0.068 | -0.084 | 0.184 | 4.66e-01 |
| edge_pheno621 | adulthood_BMI | Weighted median | 0.012 | 0.040 | -0.066 | 0.091 | 7.56e-01 |
| edge_pheno621 | adulthood_BMI | Simple mode | 0.068 | 0.124 | -0.174 | 0.311 | 5.82e-01 |
| edge_pheno621 | adulthood_BMI | Weighted mode | 0.024 | 0.088 | -0.148 | 0.196 | 7.85e-01 |
| edge_pheno624 | adulthood_BMI | MR Egger | 0.030 | 0.066 | -0.100 | 0.160 | 6.53e-01 |
| edge_pheno624 | adulthood_BMI | Weighted median | 0.009 | 0.039 | -0.068 | 0.087 | 8.12e-01 |
| edge_pheno624 | adulthood_BMI | Simple mode | -0.004 | 0.123 | -0.246 | 0.237 | 9.71e-01 |
| edge_pheno624 | adulthood_BMI | Weighted mode | -0.004 | 0.082 | -0.165 | 0.156 | 9.57e-01 |
| edge_pheno636 | adulthood_BMI | MR Egger | 0.007 | 0.071 | -0.131 | 0.146 | 9.17e-01 |
| edge_pheno636 | adulthood_BMI | Weighted median | 0.029 | 0.039 | -0.048 | 0.106 | 4.59e-01 |
| edge_pheno636 | adulthood_BMI | Simple mode | 0.033 | 0.128 | -0.218 | 0.284 | 7.97e-01 |
| edge_pheno636 | adulthood_BMI | Weighted mode | 0.021 | 0.082 | -0.140 | 0.182 | 8.03e-01 |
| edge_pheno639 | adulthood_BMI | MR Egger | -0.070 | 0.064 | -0.196 | 0.056 | 2.77e-01 |
| edge_pheno639 | adulthood_BMI | Weighted median | -0.016 | 0.041 | -0.096 | 0.064 | 6.89e-01 |
| edge_pheno639 | adulthood_BMI | Simple mode | 0.068 | 0.127 | -0.180 | 0.317 | 5.91e-01 |
| edge_pheno639 | adulthood_BMI | Weighted mode | -0.041 | 0.088 | -0.214 | 0.132 | 6.42e-01 |
| edge_pheno65 | adulthood_BMI | MR Egger | 0.046 | 0.066 | -0.084 | 0.176 | 4.87e-01 |
| edge_pheno65 | adulthood_BMI | Weighted median | -0.004 | 0.039 | -0.081 | 0.072 | 9.09e-01 |
| edge_pheno65 | adulthood_BMI | Simple mode | -0.080 | 0.124 | -0.323 | 0.162 | 5.17e-01 |
| edge_pheno65 | adulthood_BMI | Weighted mode | 0.007 | 0.083 | -0.155 | 0.169 | 9.34e-01 |
| edge_pheno66 | adulthood_BMI | MR Egger | -0.060 | 0.066 | -0.190 | 0.069 | 3.60e-01 |
| edge_pheno66 | adulthood_BMI | Weighted median | -0.004 | 0.039 | -0.081 | 0.073 | 9.12e-01 |
| edge_pheno66 | adulthood_BMI | Simple mode | -0.006 | 0.135 | -0.271 | 0.258 | 9.62e-01 |
| edge_pheno66 | adulthood_BMI | Weighted mode | -0.019 | 0.086 | -0.187 | 0.149 | 8.28e-01 |
| edge_pheno674 | adulthood_BMI | MR Egger | -0.031 | 0.068 | -0.165 | 0.102 | 6.49e-01 |
| edge_pheno674 | adulthood_BMI | Weighted median | -0.006 | 0.039 | -0.082 | 0.070 | 8.85e-01 |
| edge_pheno674 | adulthood_BMI | Simple mode | -0.012 | 0.143 | -0.293 | 0.269 | 9.33e-01 |
| edge_pheno674 | adulthood_BMI | Weighted mode | 0.011 | 0.105 | -0.195 | 0.216 | 9.20e-01 |
| edge_pheno681 | adulthood_BMI | MR Egger | -0.083 | 0.069 | -0.217 | 0.052 | 2.30e-01 |
| edge_pheno681 | adulthood_BMI | Weighted median | -0.002 | 0.038 | -0.077 | 0.074 | 9.63e-01 |
| edge_pheno681 | adulthood_BMI | Simple mode | 0.020 | 0.136 | -0.247 | 0.287 | 8.85e-01 |
| edge_pheno681 | adulthood_BMI | Weighted mode | -0.032 | 0.094 | -0.215 | 0.152 | 7.36e-01 |
| edge_pheno683 | adulthood_BMI | MR Egger | 0.027 | 0.067 | -0.105 | 0.159 | 6.92e-01 |
| edge_pheno683 | adulthood_BMI | Weighted median | -0.023 | 0.039 | -0.099 | 0.054 | 5.63e-01 |
| edge_pheno683 | adulthood_BMI | Simple mode | 0.057 | 0.125 | -0.189 | 0.303 | 6.49e-01 |
| edge_pheno683 | adulthood_BMI | Weighted mode | -0.015 | 0.082 | -0.175 | 0.145 | 8.57e-01 |
| edge_pheno695 | adulthood_BMI | MR Egger | 0.052 | 0.067 | -0.079 | 0.184 | 4.35e-01 |
| edge_pheno695 | adulthood_BMI | Weighted median | 0.054 | 0.038 | -0.020 | 0.128 | 1.53e-01 |
| edge_pheno695 | adulthood_BMI | Simple mode | -0.063 | 0.131 | -0.320 | 0.193 | 6.29e-01 |
| edge_pheno695 | adulthood_BMI | Weighted mode | 0.020 | 0.084 | -0.144 | 0.185 | 8.07e-01 |
| edge_pheno698 | adulthood_BMI | MR Egger | -0.079 | 0.067 | -0.210 | 0.052 | 2.37e-01 |
| edge_pheno698 | adulthood_BMI | Weighted median | -0.062 | 0.040 | -0.140 | 0.016 | 1.21e-01 |
| edge_pheno698 | adulthood_BMI | Simple mode | -0.093 | 0.136 | -0.360 | 0.174 | 4.95e-01 |
| edge_pheno698 | adulthood_BMI | Weighted mode | -0.134 | 0.079 | -0.289 | 0.022 | 9.25e-02 |
| edge_pheno705 | adulthood_BMI | MR Egger | 0.027 | 0.068 | -0.106 | 0.160 | 6.92e-01 |
| edge_pheno705 | adulthood_BMI | Weighted median | 0.006 | 0.038 | -0.070 | 0.081 | 8.85e-01 |
| edge_pheno705 | adulthood_BMI | Simple mode | 0.040 | 0.140 | -0.235 | 0.314 | 7.78e-01 |
| edge_pheno705 | adulthood_BMI | Weighted mode | 0.028 | 0.117 | -0.202 | 0.258 | 8.13e-01 |
| edge_pheno716 | adulthood_BMI | MR Egger | -0.104 | 0.067 | -0.236 | 0.028 | 1.23e-01 |
| edge_pheno716 | adulthood_BMI | Weighted median | -0.066 | 0.040 | -0.145 | 0.012 | 9.88e-02 |
| edge_pheno716 | adulthood_BMI | Simple mode | -0.046 | 0.144 | -0.328 | 0.235 | 7.48e-01 |
| edge_pheno716 | adulthood_BMI | Weighted mode | -0.117 | 0.090 | -0.293 | 0.059 | 1.94e-01 |
| edge_pheno767 | adulthood_BMI | MR Egger | 0.067 | 0.067 | -0.063 | 0.198 | 3.12e-01 |
| edge_pheno767 | adulthood_BMI | Weighted median | -0.024 | 0.040 | -0.102 | 0.054 | 5.49e-01 |
| edge_pheno767 | adulthood_BMI | Simple mode | -0.046 | 0.127 | -0.294 | 0.203 | 7.19e-01 |
| edge_pheno767 | adulthood_BMI | Weighted mode | 0.008 | 0.077 | -0.143 | 0.159 | 9.19e-01 |
| edge_pheno777 | adulthood_BMI | MR Egger | -0.073 | 0.072 | -0.214 | 0.069 | 3.16e-01 |
| edge_pheno777 | adulthood_BMI | Weighted median | -0.030 | 0.040 | -0.110 | 0.049 | 4.50e-01 |
| edge_pheno777 | adulthood_BMI | Simple mode | 0.003 | 0.129 | -0.249 | 0.255 | 9.81e-01 |
| edge_pheno777 | adulthood_BMI | Weighted mode | -0.023 | 0.106 | -0.231 | 0.185 | 8.29e-01 |
| edge_pheno789 | adulthood_BMI | MR Egger | 0.003 | 0.065 | -0.124 | 0.131 | 9.59e-01 |
| edge_pheno789 | adulthood_BMI | Weighted median | -0.032 | 0.039 | -0.108 | 0.045 | 4.17e-01 |
| edge_pheno789 | adulthood_BMI | Simple mode | -0.038 | 0.125 | -0.284 | 0.208 | 7.60e-01 |
| edge_pheno789 | adulthood_BMI | Weighted mode | 0.025 | 0.083 | -0.138 | 0.188 | 7.64e-01 |
| edge_pheno794 | adulthood_BMI | MR Egger | 0.028 | 0.064 | -0.097 | 0.152 | 6.65e-01 |
| edge_pheno794 | adulthood_BMI | Weighted median | 0.000 | 0.038 | -0.074 | 0.074 | 9.96e-01 |
| edge_pheno794 | adulthood_BMI | Simple mode | -0.045 | 0.127 | -0.293 | 0.203 | 7.25e-01 |
| edge_pheno794 | adulthood_BMI | Weighted mode | -0.022 | 0.080 | -0.179 | 0.135 | 7.82e-01 |
| edge_pheno801 | adulthood_BMI | MR Egger | 0.114 | 0.070 | -0.022 | 0.251 | 1.01e-01 |
| edge_pheno801 | adulthood_BMI | Weighted median | 0.063 | 0.038 | -0.012 | 0.137 | 9.97e-02 |
| edge_pheno801 | adulthood_BMI | Simple mode | 0.026 | 0.139 | -0.246 | 0.298 | 8.50e-01 |
| edge_pheno801 | adulthood_BMI | Weighted mode | -0.020 | 0.122 | -0.259 | 0.218 | 8.66e-01 |
| edge_pheno810 | adulthood_BMI | MR Egger | -0.096 | 0.065 | -0.224 | 0.032 | 1.43e-01 |
| edge_pheno810 | adulthood_BMI | Weighted median | -0.053 | 0.039 | -0.129 | 0.024 | 1.76e-01 |
| edge_pheno810 | adulthood_BMI | Simple mode | -0.112 | 0.122 | -0.351 | 0.127 | 3.59e-01 |
| edge_pheno810 | adulthood_BMI | Weighted mode | -0.058 | 0.077 | -0.210 | 0.094 | 4.53e-01 |
| edge_pheno812 | adulthood_BMI | MR Egger | -0.019 | 0.072 | -0.161 | 0.123 | 7.93e-01 |
| edge_pheno812 | adulthood_BMI | Weighted median | -0.021 | 0.042 | -0.103 | 0.061 | 6.17e-01 |
| edge_pheno812 | adulthood_BMI | Simple mode | 0.177 | 0.124 | -0.065 | 0.420 | 1.52e-01 |
| edge_pheno812 | adulthood_BMI | Weighted mode | -0.061 | 0.075 | -0.209 | 0.086 | 4.15e-01 |
| edge_pheno815 | adulthood_BMI | MR Egger | -0.026 | 0.072 | -0.166 | 0.115 | 7.19e-01 |
| edge_pheno815 | adulthood_BMI | Weighted median | 0.031 | 0.040 | -0.047 | 0.109 | 4.41e-01 |
| edge_pheno815 | adulthood_BMI | Simple mode | 0.056 | 0.137 | -0.211 | 0.324 | 6.80e-01 |
| edge_pheno815 | adulthood_BMI | Weighted mode | 0.015 | 0.091 | -0.163 | 0.192 | 8.72e-01 |
| edge_pheno816 | adulthood_BMI | MR Egger | -0.089 | 0.065 | -0.218 | 0.039 | 1.73e-01 |
| edge_pheno816 | adulthood_BMI | Weighted median | -0.033 | 0.038 | -0.106 | 0.041 | 3.87e-01 |
| edge_pheno816 | adulthood_BMI | Simple mode | 0.031 | 0.131 | -0.225 | 0.287 | 8.13e-01 |
| edge_pheno816 | adulthood_BMI | Weighted mode | -0.009 | 0.091 | -0.188 | 0.170 | 9.21e-01 |
| edge_pheno824 | adulthood_BMI | MR Egger | -0.051 | 0.070 | -0.188 | 0.087 | 4.70e-01 |
| edge_pheno824 | adulthood_BMI | Weighted median | -0.035 | 0.038 | -0.110 | 0.040 | 3.62e-01 |
| edge_pheno824 | adulthood_BMI | Simple mode | -0.153 | 0.136 | -0.420 | 0.114 | 2.63e-01 |
| edge_pheno824 | adulthood_BMI | Weighted mode | -0.114 | 0.083 | -0.276 | 0.049 | 1.71e-01 |
| edge_pheno867 | adulthood_BMI | MR Egger | 0.037 | 0.070 | -0.100 | 0.174 | 5.99e-01 |
| edge_pheno867 | adulthood_BMI | Weighted median | -0.034 | 0.038 | -0.108 | 0.040 | 3.71e-01 |
| edge_pheno867 | adulthood_BMI | Simple mode | -0.072 | 0.122 | -0.311 | 0.168 | 5.58e-01 |
| edge_pheno867 | adulthood_BMI | Weighted mode | -0.072 | 0.079 | -0.227 | 0.083 | 3.65e-01 |
| edge_pheno869 | adulthood_BMI | MR Egger | 0.065 | 0.075 | -0.083 | 0.213 | 3.87e-01 |
| edge_pheno869 | adulthood_BMI | Weighted median | 0.033 | 0.042 | -0.050 | 0.116 | 4.34e-01 |
| edge_pheno869 | adulthood_BMI | Simple mode | 0.105 | 0.146 | -0.180 | 0.390 | 4.71e-01 |
| edge_pheno869 | adulthood_BMI | Weighted mode | 0.056 | 0.089 | -0.120 | 0.231 | 5.33e-01 |
| edge_pheno87 | adulthood_BMI | MR Egger | -0.055 | 0.068 | -0.189 | 0.080 | 4.25e-01 |
| edge_pheno87 | adulthood_BMI | Weighted median | 0.021 | 0.041 | -0.059 | 0.101 | 6.08e-01 |
| edge_pheno87 | adulthood_BMI | Simple mode | 0.147 | 0.141 | -0.130 | 0.423 | 3.00e-01 |
| edge_pheno87 | adulthood_BMI | Weighted mode | 0.037 | 0.096 | -0.151 | 0.224 | 7.00e-01 |
| edge_pheno882 | adulthood_BMI | MR Egger | -0.078 | 0.065 | -0.205 | 0.050 | 2.35e-01 |
| edge_pheno882 | adulthood_BMI | Weighted median | -0.044 | 0.041 | -0.123 | 0.036 | 2.80e-01 |
| edge_pheno882 | adulthood_BMI | Simple mode | -0.059 | 0.129 | -0.312 | 0.194 | 6.47e-01 |
| edge_pheno882 | adulthood_BMI | Weighted mode | -0.071 | 0.082 | -0.232 | 0.089 | 3.85e-01 |
| edge_pheno899 | adulthood_BMI | MR Egger | -0.005 | 0.068 | -0.138 | 0.128 | 9.39e-01 |
| edge_pheno899 | adulthood_BMI | Weighted median | -0.006 | 0.041 | -0.085 | 0.074 | 8.89e-01 |
| edge_pheno899 | adulthood_BMI | Simple mode | -0.084 | 0.128 | -0.335 | 0.166 | 5.09e-01 |
| edge_pheno899 | adulthood_BMI | Weighted mode | -0.011 | 0.079 | -0.166 | 0.144 | 8.92e-01 |
| edge_pheno903 | adulthood_BMI | MR Egger | 0.046 | 0.069 | -0.090 | 0.181 | 5.09e-01 |
| edge_pheno903 | adulthood_BMI | Weighted median | 0.018 | 0.039 | -0.058 | 0.094 | 6.40e-01 |
| edge_pheno903 | adulthood_BMI | Simple mode | -0.008 | 0.120 | -0.242 | 0.227 | 9.48e-01 |
| edge_pheno903 | adulthood_BMI | Weighted mode | 0.041 | 0.091 | -0.138 | 0.219 | 6.54e-01 |
| edge_pheno908 | adulthood_BMI | MR Egger | -0.033 | 0.068 | -0.167 | 0.101 | 6.27e-01 |
| edge_pheno908 | adulthood_BMI | Weighted median | -0.006 | 0.039 | -0.082 | 0.070 | 8.75e-01 |
| edge_pheno908 | adulthood_BMI | Simple mode | 0.099 | 0.129 | -0.154 | 0.352 | 4.45e-01 |
| edge_pheno908 | adulthood_BMI | Weighted mode | 0.072 | 0.088 | -0.100 | 0.244 | 4.12e-01 |
| edge_pheno918 | adulthood_BMI | MR Egger | -0.042 | 0.064 | -0.167 | 0.084 | 5.15e-01 |
| edge_pheno918 | adulthood_BMI | Weighted median | 0.005 | 0.040 | -0.073 | 0.084 | 8.96e-01 |
| edge_pheno918 | adulthood_BMI | Simple mode | 0.001 | 0.127 | -0.249 | 0.251 | 9.95e-01 |
| edge_pheno918 | adulthood_BMI | Weighted mode | -0.012 | 0.083 | -0.174 | 0.151 | 8.87e-01 |
| edge_pheno932 | adulthood_BMI | MR Egger | 0.021 | 0.071 | -0.119 | 0.161 | 7.68e-01 |
| edge_pheno932 | adulthood_BMI | Weighted median | -0.031 | 0.039 | -0.107 | 0.046 | 4.29e-01 |
| edge_pheno932 | adulthood_BMI | Simple mode | -0.029 | 0.107 | -0.239 | 0.181 | 7.84e-01 |
| edge_pheno932 | adulthood_BMI | Weighted mode | -0.029 | 0.078 | -0.182 | 0.123 | 7.06e-01 |
| edge_pheno942 | adulthood_BMI | MR Egger | 0.031 | 0.073 | -0.113 | 0.175 | 6.75e-01 |
| edge_pheno942 | adulthood_BMI | Weighted median | -0.005 | 0.040 | -0.083 | 0.073 | 9.01e-01 |
| edge_pheno942 | adulthood_BMI | Simple mode | -0.017 | 0.144 | -0.300 | 0.265 | 9.05e-01 |
| edge_pheno942 | adulthood_BMI | Weighted mode | 0.025 | 0.114 | -0.199 | 0.249 | 8.29e-01 |
| edge_pheno956 | adulthood_BMI | MR Egger | 0.045 | 0.073 | -0.097 | 0.188 | 5.31e-01 |
| edge_pheno956 | adulthood_BMI | Weighted median | -0.029 | 0.042 | -0.111 | 0.052 | 4.80e-01 |
| edge_pheno956 | adulthood_BMI | Simple mode | -0.054 | 0.140 | -0.329 | 0.221 | 7.00e-01 |
| edge_pheno956 | adulthood_BMI | Weighted mode | -0.066 | 0.086 | -0.235 | 0.104 | 4.48e-01 |
| edge_pheno965 | adulthood_BMI | MR Egger | 0.074 | 0.066 | -0.054 | 0.203 | 2.58e-01 |
| edge_pheno965 | adulthood_BMI | Weighted median | 0.038 | 0.039 | -0.038 | 0.114 | 3.30e-01 |
| edge_pheno965 | adulthood_BMI | Simple mode | 0.135 | 0.135 | -0.130 | 0.400 | 3.18e-01 |
| edge_pheno965 | adulthood_BMI | Weighted mode | 0.110 | 0.086 | -0.058 | 0.279 | 2.01e-01 |
| node_pheno1 | adulthood_BMI | MR Egger | 0.097 | 0.072 | -0.044 | 0.237 | 1.77e-01 |
| node_pheno1 | adulthood_BMI | Weighted median | 0.030 | 0.040 | -0.049 | 0.110 | 4.53e-01 |
| node_pheno1 | adulthood_BMI | Simple mode | 0.142 | 0.151 | -0.154 | 0.438 | 3.47e-01 |
| node_pheno1 | adulthood_BMI | Weighted mode | 0.064 | 0.093 | -0.118 | 0.246 | 4.92e-01 |
| node_pheno10 | adulthood_BMI | MR Egger | 0.053 | 0.070 | -0.084 | 0.189 | 4.50e-01 |
| node_pheno10 | adulthood_BMI | Weighted median | -0.029 | 0.039 | -0.105 | 0.046 | 4.47e-01 |
| node_pheno10 | adulthood_BMI | Simple mode | -0.052 | 0.123 | -0.293 | 0.188 | 6.70e-01 |
| node_pheno10 | adulthood_BMI | Weighted mode | -0.065 | 0.072 | -0.207 | 0.076 | 3.64e-01 |
| node_pheno11 | adulthood_BMI | MR Egger | -0.022 | 0.069 | -0.158 | 0.113 | 7.49e-01 |
| node_pheno11 | adulthood_BMI | Weighted median | -0.070 | 0.040 | -0.148 | 0.008 | 7.95e-02 |
| node_pheno11 | adulthood_BMI | Simple mode | -0.025 | 0.133 | -0.286 | 0.236 | 8.49e-01 |
| node_pheno11 | adulthood_BMI | Weighted mode | -0.133 | 0.081 | -0.291 | 0.025 | 9.90e-02 |
| node_pheno12 | adulthood_BMI | MR Egger | 0.078 | 0.069 | -0.056 | 0.213 | 2.54e-01 |
| node_pheno12 | adulthood_BMI | Weighted median | 0.027 | 0.039 | -0.050 | 0.104 | 4.93e-01 |
| node_pheno12 | adulthood_BMI | Simple mode | 0.023 | 0.116 | -0.205 | 0.251 | 8.42e-01 |
| node_pheno12 | adulthood_BMI | Weighted mode | 0.009 | 0.075 | -0.139 | 0.157 | 9.03e-01 |
| node_pheno13 | adulthood_BMI | MR Egger | 0.039 | 0.072 | -0.103 | 0.180 | 5.93e-01 |
| node_pheno13 | adulthood_BMI | Weighted median | -0.051 | 0.040 | -0.128 | 0.027 | 2.02e-01 |
| node_pheno13 | adulthood_BMI | Simple mode | -0.088 | 0.140 | -0.363 | 0.186 | 5.28e-01 |
| node_pheno13 | adulthood_BMI | Weighted mode | -0.088 | 0.086 | -0.257 | 0.080 | 3.05e-01 |
| node_pheno14 | adulthood_BMI | MR Egger | 0.160 | 0.070 | 0.022 | 0.297 | 2.34e-02 |
| node_pheno14 | adulthood_BMI | Weighted median | 0.034 | 0.040 | -0.045 | 0.113 | 4.00e-01 |
| node_pheno14 | adulthood_BMI | Simple mode | 0.094 | 0.149 | -0.198 | 0.386 | 5.28e-01 |
| node_pheno14 | adulthood_BMI | Weighted mode | 0.032 | 0.095 | -0.155 | 0.218 | 7.40e-01 |
| node_pheno15 | adulthood_BMI | MR Egger | 0.096 | 0.072 | -0.044 | 0.237 | 1.80e-01 |
| node_pheno15 | adulthood_BMI | Weighted median | -0.012 | 0.039 | -0.088 | 0.064 | 7.56e-01 |
| node_pheno15 | adulthood_BMI | Simple mode | 0.106 | 0.129 | -0.148 | 0.359 | 4.14e-01 |
| node_pheno15 | adulthood_BMI | Weighted mode | 0.009 | 0.085 | -0.157 | 0.176 | 9.12e-01 |
| node_pheno16 | adulthood_BMI | MR Egger | 0.070 | 0.067 | -0.062 | 0.201 | 2.99e-01 |
| node_pheno16 | adulthood_BMI | Weighted median | 0.019 | 0.038 | -0.057 | 0.094 | 6.24e-01 |
| node_pheno16 | adulthood_BMI | Simple mode | -0.011 | 0.132 | -0.270 | 0.249 | 9.36e-01 |
| node_pheno16 | adulthood_BMI | Weighted mode | 0.024 | 0.094 | -0.160 | 0.208 | 7.98e-01 |
| node_pheno17 | adulthood_BMI | MR Egger | 0.083 | 0.069 | -0.052 | 0.218 | 2.29e-01 |
| node_pheno17 | adulthood_BMI | Weighted median | 0.011 | 0.040 | -0.067 | 0.089 | 7.88e-01 |
| node_pheno17 | adulthood_BMI | Simple mode | -0.026 | 0.144 | -0.309 | 0.256 | 8.55e-01 |
| node_pheno17 | adulthood_BMI | Weighted mode | 0.010 | 0.088 | -0.162 | 0.182 | 9.08e-01 |
| node_pheno18 | adulthood_BMI | MR Egger | 0.023 | 0.067 | -0.109 | 0.156 | 7.29e-01 |
| node_pheno18 | adulthood_BMI | Weighted median | 0.008 | 0.039 | -0.069 | 0.085 | 8.35e-01 |
| node_pheno18 | adulthood_BMI | Simple mode | 0.175 | 0.141 | -0.102 | 0.452 | 2.16e-01 |
| node_pheno18 | adulthood_BMI | Weighted mode | 0.020 | 0.093 | -0.162 | 0.202 | 8.27e-01 |
| node_pheno19 | adulthood_BMI | MR Egger | 0.039 | 0.067 | -0.093 | 0.172 | 5.58e-01 |
| node_pheno19 | adulthood_BMI | Weighted median | 0.041 | 0.039 | -0.036 | 0.118 | 2.96e-01 |
| node_pheno19 | adulthood_BMI | Simple mode | 0.074 | 0.126 | -0.173 | 0.321 | 5.58e-01 |
| node_pheno19 | adulthood_BMI | Weighted mode | 0.087 | 0.085 | -0.080 | 0.253 | 3.09e-01 |
| node_pheno2 | adulthood_BMI | MR Egger | -0.016 | 0.069 | -0.151 | 0.118 | 8.13e-01 |
| node_pheno2 | adulthood_BMI | Weighted median | -0.009 | 0.040 | -0.087 | 0.069 | 8.22e-01 |
| node_pheno2 | adulthood_BMI | Simple mode | 0.053 | 0.136 | -0.213 | 0.320 | 6.95e-01 |
| node_pheno2 | adulthood_BMI | Weighted mode | -0.025 | 0.097 | -0.216 | 0.165 | 7.94e-01 |
| node_pheno20 | adulthood_BMI | MR Egger | 0.037 | 0.067 | -0.095 | 0.168 | 5.84e-01 |
| node_pheno20 | adulthood_BMI | Weighted median | 0.054 | 0.039 | -0.022 | 0.130 | 1.66e-01 |
| node_pheno20 | adulthood_BMI | Simple mode | 0.124 | 0.132 | -0.135 | 0.382 | 3.49e-01 |
| node_pheno20 | adulthood_BMI | Weighted mode | 0.095 | 0.108 | -0.116 | 0.307 | 3.77e-01 |
| node_pheno21 | adulthood_BMI | MR Egger | 0.084 | 0.069 | -0.052 | 0.220 | 2.26e-01 |
| node_pheno21 | adulthood_BMI | Weighted median | 0.048 | 0.039 | -0.028 | 0.124 | 2.19e-01 |
| node_pheno21 | adulthood_BMI | Simple mode | 0.135 | 0.130 | -0.120 | 0.389 | 3.00e-01 |
| node_pheno21 | adulthood_BMI | Weighted mode | 0.063 | 0.076 | -0.086 | 0.213 | 4.07e-01 |
| node_pheno22 | adulthood_BMI | MR Egger | 0.014 | 0.066 | -0.116 | 0.144 | 8.31e-01 |
| node_pheno22 | adulthood_BMI | Weighted median | 0.028 | 0.038 | -0.047 | 0.103 | 4.60e-01 |
| node_pheno22 | adulthood_BMI | Simple mode | 0.146 | 0.123 | -0.096 | 0.388 | 2.37e-01 |
| node_pheno22 | adulthood_BMI | Weighted mode | 0.066 | 0.093 | -0.115 | 0.247 | 4.77e-01 |
| node_pheno23 | adulthood_BMI | MR Egger | 0.117 | 0.069 | -0.017 | 0.252 | 8.75e-02 |
| node_pheno23 | adulthood_BMI | Weighted median | 0.041 | 0.037 | -0.032 | 0.115 | 2.67e-01 |
| node_pheno23 | adulthood_BMI | Simple mode | 0.060 | 0.134 | -0.203 | 0.322 | 6.56e-01 |
| node_pheno23 | adulthood_BMI | Weighted mode | -0.022 | 0.080 | -0.179 | 0.136 | 7.86e-01 |
| node_pheno24 | adulthood_BMI | MR Egger | 0.078 | 0.067 | -0.054 | 0.210 | 2.47e-01 |
| node_pheno24 | adulthood_BMI | Weighted median | 0.059 | 0.037 | -0.014 | 0.131 | 1.11e-01 |
| node_pheno24 | adulthood_BMI | Simple mode | 0.148 | 0.133 | -0.113 | 0.409 | 2.66e-01 |
| node_pheno24 | adulthood_BMI | Weighted mode | 0.035 | 0.083 | -0.127 | 0.197 | 6.74e-01 |
| node_pheno25 | adulthood_BMI | MR Egger | 0.035 | 0.066 | -0.095 | 0.165 | 5.94e-01 |
| node_pheno25 | adulthood_BMI | Weighted median | 0.073 | 0.038 | -0.002 | 0.148 | 5.62e-02 |
| node_pheno25 | adulthood_BMI | Simple mode | 0.145 | 0.126 | -0.102 | 0.392 | 2.51e-01 |
| node_pheno25 | adulthood_BMI | Weighted mode | 0.081 | 0.084 | -0.083 | 0.245 | 3.33e-01 |
| node_pheno26 | adulthood_BMI | MR Egger | 0.069 | 0.062 | -0.052 | 0.189 | 2.66e-01 |
| node_pheno26 | adulthood_BMI | Weighted median | 0.046 | 0.035 | -0.023 | 0.114 | 1.93e-01 |
| node_pheno26 | adulthood_BMI | Simple mode | 0.070 | 0.115 | -0.156 | 0.296 | 5.45e-01 |
| node_pheno26 | adulthood_BMI | Weighted mode | 0.018 | 0.080 | -0.140 | 0.175 | 8.27e-01 |
| node_pheno27 | adulthood_BMI | MR Egger | 0.016 | 0.070 | -0.121 | 0.152 | 8.22e-01 |
| node_pheno27 | adulthood_BMI | Weighted median | -0.020 | 0.039 | -0.097 | 0.057 | 6.17e-01 |
| node_pheno27 | adulthood_BMI | Simple mode | -0.004 | 0.137 | -0.273 | 0.265 | 9.76e-01 |
| node_pheno27 | adulthood_BMI | Weighted mode | -0.079 | 0.083 | -0.241 | 0.084 | 3.42e-01 |
| node_pheno28 | adulthood_BMI | MR Egger | 0.145 | 0.065 | 0.017 | 0.273 | 2.64e-02 |
| node_pheno28 | adulthood_BMI | Weighted median | 0.059 | 0.038 | -0.015 | 0.132 | 1.17e-01 |
| node_pheno28 | adulthood_BMI | Simple mode | 0.147 | 0.113 | -0.075 | 0.369 | 1.95e-01 |
| node_pheno28 | adulthood_BMI | Weighted mode | 0.054 | 0.074 | -0.091 | 0.199 | 4.65e-01 |
| node_pheno29 | adulthood_BMI | MR Egger | 0.113 | 0.065 | -0.015 | 0.241 | 8.33e-02 |
| node_pheno29 | adulthood_BMI | Weighted median | 0.074 | 0.039 | -0.002 | 0.150 | 5.61e-02 |
| node_pheno29 | adulthood_BMI | Simple mode | 0.097 | 0.127 | -0.152 | 0.345 | 4.46e-01 |
| node_pheno29 | adulthood_BMI | Weighted mode | 0.074 | 0.083 | -0.088 | 0.237 | 3.70e-01 |
| node_pheno3 | adulthood_BMI | MR Egger | 0.065 | 0.070 | -0.073 | 0.203 | 3.53e-01 |
| node_pheno3 | adulthood_BMI | Weighted median | -0.025 | 0.041 | -0.105 | 0.055 | 5.37e-01 |
| node_pheno3 | adulthood_BMI | Simple mode | 0.017 | 0.142 | -0.261 | 0.296 | 9.02e-01 |
| node_pheno3 | adulthood_BMI | Weighted mode | -0.066 | 0.091 | -0.245 | 0.113 | 4.70e-01 |
| node_pheno30 | adulthood_BMI | MR Egger | 0.148 | 0.064 | 0.022 | 0.274 | 2.14e-02 |
| node_pheno30 | adulthood_BMI | Weighted median | 0.002 | 0.038 | -0.073 | 0.078 | 9.50e-01 |
| node_pheno30 | adulthood_BMI | Simple mode | -0.001 | 0.126 | -0.248 | 0.247 | 9.96e-01 |
| node_pheno30 | adulthood_BMI | Weighted mode | -0.001 | 0.081 | -0.159 | 0.158 | 9.94e-01 |
| node_pheno31 | adulthood_BMI | MR Egger | 0.104 | 0.069 | -0.031 | 0.238 | 1.31e-01 |
| node_pheno31 | adulthood_BMI | Weighted median | 0.052 | 0.037 | -0.020 | 0.123 | 1.59e-01 |
| node_pheno31 | adulthood_BMI | Simple mode | 0.368 | 0.146 | 0.083 | 0.654 | 1.17e-02 |
| node_pheno31 | adulthood_BMI | Weighted mode | -0.101 | 0.111 | -0.319 | 0.116 | 3.62e-01 |
| node_pheno32 | adulthood_BMI | MR Egger | 0.031 | 0.065 | -0.095 | 0.158 | 6.30e-01 |
| node_pheno32 | adulthood_BMI | Weighted median | 0.077 | 0.038 | 0.002 | 0.152 | 4.48e-02 |
| node_pheno32 | adulthood_BMI | Simple mode | 0.185 | 0.128 | -0.066 | 0.436 | 1.48e-01 |
| node_pheno32 | adulthood_BMI | Weighted mode | 0.090 | 0.101 | -0.108 | 0.288 | 3.72e-01 |
| node_pheno33 | adulthood_BMI | MR Egger | 0.069 | 0.070 | -0.068 | 0.205 | 3.23e-01 |
| node_pheno33 | adulthood_BMI | Weighted median | 0.030 | 0.039 | -0.045 | 0.106 | 4.31e-01 |
| node_pheno33 | adulthood_BMI | Simple mode | 0.046 | 0.126 | -0.200 | 0.293 | 7.12e-01 |
| node_pheno33 | adulthood_BMI | Weighted mode | 0.021 | 0.075 | -0.126 | 0.169 | 7.78e-01 |
| node_pheno34 | adulthood_BMI | MR Egger | 0.089 | 0.068 | -0.043 | 0.222 | 1.87e-01 |
| node_pheno34 | adulthood_BMI | Weighted median | 0.084 | 0.038 | 0.010 | 0.157 | 2.66e-02 |
| node_pheno34 | adulthood_BMI | Simple mode | 0.087 | 0.135 | -0.178 | 0.351 | 5.21e-01 |
| node_pheno34 | adulthood_BMI | Weighted mode | 0.060 | 0.082 | -0.100 | 0.220 | 4.61e-01 |
| node_pheno35 | adulthood_BMI | MR Egger | 0.037 | 0.065 | -0.091 | 0.164 | 5.74e-01 |
| node_pheno35 | adulthood_BMI | Weighted median | 0.064 | 0.035 | -0.006 | 0.133 | 7.21e-02 |
| node_pheno35 | adulthood_BMI | Simple mode | 0.110 | 0.114 | -0.114 | 0.335 | 3.35e-01 |
| node_pheno35 | adulthood_BMI | Weighted mode | 0.099 | 0.083 | -0.064 | 0.261 | 2.36e-01 |
| node_pheno36 | adulthood_BMI | MR Egger | 0.099 | 0.065 | -0.029 | 0.227 | 1.30e-01 |
| node_pheno36 | adulthood_BMI | Weighted median | 0.036 | 0.037 | -0.035 | 0.108 | 3.20e-01 |
| node_pheno36 | adulthood_BMI | Simple mode | 0.097 | 0.125 | -0.147 | 0.341 | 4.38e-01 |
| node_pheno36 | adulthood_BMI | Weighted mode | 0.009 | 0.079 | -0.145 | 0.163 | 9.06e-01 |
| node_pheno37 | adulthood_BMI | MR Egger | 0.031 | 0.069 | -0.105 | 0.166 | 6.58e-01 |
| node_pheno37 | adulthood_BMI | Weighted median | 0.054 | 0.038 | -0.021 | 0.129 | 1.55e-01 |
| node_pheno37 | adulthood_BMI | Simple mode | 0.121 | 0.131 | -0.137 | 0.379 | 3.58e-01 |
| node_pheno37 | adulthood_BMI | Weighted mode | 0.046 | 0.093 | -0.135 | 0.228 | 6.17e-01 |
| node_pheno38 | adulthood_BMI | MR Egger | 0.103 | 0.068 | -0.030 | 0.235 | 1.29e-01 |
| node_pheno38 | adulthood_BMI | Weighted median | 0.052 | 0.036 | -0.019 | 0.123 | 1.51e-01 |
| node_pheno38 | adulthood_BMI | Simple mode | 0.045 | 0.121 | -0.193 | 0.282 | 7.12e-01 |
| node_pheno38 | adulthood_BMI | Weighted mode | 0.045 | 0.084 | -0.119 | 0.209 | 5.93e-01 |
| node_pheno39 | adulthood_BMI | MR Egger | 0.069 | 0.067 | -0.062 | 0.201 | 3.02e-01 |
| node_pheno39 | adulthood_BMI | Weighted median | 0.070 | 0.039 | -0.006 | 0.146 | 7.00e-02 |
| node_pheno39 | adulthood_BMI | Simple mode | 0.181 | 0.132 | -0.077 | 0.439 | 1.69e-01 |
| node_pheno39 | adulthood_BMI | Weighted mode | 0.119 | 0.089 | -0.055 | 0.294 | 1.79e-01 |
| node_pheno4 | adulthood_BMI | MR Egger | 0.058 | 0.067 | -0.074 | 0.189 | 3.93e-01 |
| node_pheno4 | adulthood_BMI | Weighted median | -0.011 | 0.039 | -0.086 | 0.065 | 7.81e-01 |
| node_pheno4 | adulthood_BMI | Simple mode | -0.063 | 0.136 | -0.330 | 0.204 | 6.46e-01 |
| node_pheno4 | adulthood_BMI | Weighted mode | 0.001 | 0.092 | -0.179 | 0.181 | 9.90e-01 |
| node_pheno40 | adulthood_BMI | MR Egger | 0.073 | 0.066 | -0.056 | 0.202 | 2.66e-01 |
| node_pheno40 | adulthood_BMI | Weighted median | 0.000 | 0.037 | -0.072 | 0.072 | 9.99e-01 |
| node_pheno40 | adulthood_BMI | Simple mode | 0.018 | 0.126 | -0.228 | 0.264 | 8.86e-01 |
| node_pheno40 | adulthood_BMI | Weighted mode | -0.027 | 0.080 | -0.184 | 0.130 | 7.35e-01 |
| node_pheno41 | adulthood_BMI | MR Egger | 0.056 | 0.071 | -0.082 | 0.195 | 4.27e-01 |
| node_pheno41 | adulthood_BMI | Weighted median | 0.032 | 0.038 | -0.043 | 0.107 | 4.01e-01 |
| node_pheno41 | adulthood_BMI | Simple mode | 0.092 | 0.129 | -0.161 | 0.344 | 4.77e-01 |
| node_pheno41 | adulthood_BMI | Weighted mode | 0.032 | 0.084 | -0.132 | 0.196 | 6.98e-01 |
| node_pheno42 | adulthood_BMI | MR Egger | 0.074 | 0.068 | -0.059 | 0.208 | 2.75e-01 |
| node_pheno42 | adulthood_BMI | Weighted median | 0.023 | 0.037 | -0.049 | 0.094 | 5.38e-01 |
| node_pheno42 | adulthood_BMI | Simple mode | 0.094 | 0.130 | -0.161 | 0.348 | 4.71e-01 |
| node_pheno42 | adulthood_BMI | Weighted mode | 0.023 | 0.086 | -0.146 | 0.191 | 7.93e-01 |
| node_pheno43 | adulthood_BMI | MR Egger | 0.115 | 0.070 | -0.022 | 0.252 | 1.01e-01 |
| node_pheno43 | adulthood_BMI | Weighted median | -0.001 | 0.040 | -0.079 | 0.077 | 9.72e-01 |
| node_pheno43 | adulthood_BMI | Simple mode | 0.212 | 0.131 | -0.044 | 0.468 | 1.06e-01 |
| node_pheno43 | adulthood_BMI | Weighted mode | -0.023 | 0.088 | -0.196 | 0.149 | 7.90e-01 |
| node_pheno44 | adulthood_BMI | MR Egger | 0.071 | 0.063 | -0.053 | 0.195 | 2.65e-01 |
| node_pheno44 | adulthood_BMI | Weighted median | 0.025 | 0.035 | -0.044 | 0.095 | 4.73e-01 |
| node_pheno44 | adulthood_BMI | Simple mode | 0.050 | 0.124 | -0.194 | 0.294 | 6.89e-01 |
| node_pheno44 | adulthood_BMI | Weighted mode | 0.008 | 0.076 | -0.141 | 0.157 | 9.20e-01 |
| node_pheno45 | adulthood_BMI | MR Egger | 0.064 | 0.064 | -0.061 | 0.189 | 3.15e-01 |
| node_pheno45 | adulthood_BMI | Weighted median | -0.004 | 0.037 | -0.077 | 0.070 | 9.22e-01 |
| node_pheno45 | adulthood_BMI | Simple mode | -0.094 | 0.125 | -0.340 | 0.152 | 4.53e-01 |
| node_pheno45 | adulthood_BMI | Weighted mode | -0.116 | 0.082 | -0.277 | 0.045 | 1.57e-01 |
| node_pheno46 | adulthood_BMI | MR Egger | 0.096 | 0.072 | -0.044 | 0.236 | 1.81e-01 |
| node_pheno46 | adulthood_BMI | Weighted median | -0.006 | 0.040 | -0.084 | 0.073 | 8.87e-01 |
| node_pheno46 | adulthood_BMI | Simple mode | -0.120 | 0.141 | -0.397 | 0.157 | 3.96e-01 |
| node_pheno46 | adulthood_BMI | Weighted mode | -0.120 | 0.092 | -0.301 | 0.061 | 1.93e-01 |
| node_pheno47 | adulthood_BMI | MR Egger | 0.073 | 0.067 | -0.058 | 0.204 | 2.74e-01 |
| node_pheno47 | adulthood_BMI | Weighted median | -0.014 | 0.038 | -0.090 | 0.061 | 7.05e-01 |
| node_pheno47 | adulthood_BMI | Simple mode | -0.061 | 0.129 | -0.314 | 0.192 | 6.36e-01 |
| node_pheno47 | adulthood_BMI | Weighted mode | -0.013 | 0.078 | -0.166 | 0.141 | 8.73e-01 |
| node_pheno48 | adulthood_BMI | MR Egger | 0.128 | 0.069 | -0.008 | 0.264 | 6.54e-02 |
| node_pheno48 | adulthood_BMI | Weighted median | 0.029 | 0.039 | -0.046 | 0.105 | 4.49e-01 |
| node_pheno48 | adulthood_BMI | Simple mode | 0.046 | 0.135 | -0.218 | 0.310 | 7.34e-01 |
| node_pheno48 | adulthood_BMI | Weighted mode | 0.023 | 0.085 | -0.143 | 0.189 | 7.89e-01 |
| node_pheno49 | adulthood_BMI | MR Egger | 0.051 | 0.073 | -0.093 | 0.194 | 4.89e-01 |
| node_pheno49 | adulthood_BMI | Weighted median | -0.014 | 0.038 | -0.089 | 0.061 | 7.17e-01 |
| node_pheno49 | adulthood_BMI | Simple mode | -0.089 | 0.140 | -0.364 | 0.186 | 5.27e-01 |
| node_pheno49 | adulthood_BMI | Weighted mode | -0.020 | 0.086 | -0.189 | 0.148 | 8.12e-01 |
| node_pheno5 | adulthood_BMI | MR Egger | 0.139 | 0.074 | -0.005 | 0.284 | 5.89e-02 |
| node_pheno5 | adulthood_BMI | Weighted median | 0.020 | 0.042 | -0.063 | 0.103 | 6.31e-01 |
| node_pheno5 | adulthood_BMI | Simple mode | -0.032 | 0.136 | -0.299 | 0.236 | 8.17e-01 |
| node_pheno5 | adulthood_BMI | Weighted mode | 0.033 | 0.088 | -0.139 | 0.206 | 7.04e-01 |
| node_pheno50 | adulthood_BMI | MR Egger | 0.100 | 0.066 | -0.030 | 0.229 | 1.31e-01 |
| node_pheno50 | adulthood_BMI | Weighted median | 0.028 | 0.036 | -0.044 | 0.099 | 4.47e-01 |
| node_pheno50 | adulthood_BMI | Simple mode | -0.085 | 0.129 | -0.337 | 0.167 | 5.08e-01 |
| node_pheno50 | adulthood_BMI | Weighted mode | -0.052 | 0.084 | -0.217 | 0.112 | 5.33e-01 |
| node_pheno51 | adulthood_BMI | MR Egger | 0.056 | 0.070 | -0.080 | 0.193 | 4.19e-01 |
| node_pheno51 | adulthood_BMI | Weighted median | 0.077 | 0.040 | -0.001 | 0.155 | 5.32e-02 |
| node_pheno51 | adulthood_BMI | Simple mode | 0.150 | 0.125 | -0.095 | 0.394 | 2.31e-01 |
| node_pheno51 | adulthood_BMI | Weighted mode | 0.103 | 0.071 | -0.036 | 0.243 | 1.47e-01 |
| node_pheno52 | adulthood_BMI | MR Egger | 0.081 | 0.067 | -0.051 | 0.212 | 2.30e-01 |
| node_pheno52 | adulthood_BMI | Weighted median | 0.098 | 0.039 | 0.022 | 0.174 | 1.14e-02 |
| node_pheno52 | adulthood_BMI | Simple mode | 0.132 | 0.119 | -0.101 | 0.365 | 2.66e-01 |
| node_pheno52 | adulthood_BMI | Weighted mode | 0.119 | 0.088 | -0.052 | 0.291 | 1.74e-01 |
| node_pheno53 | adulthood_BMI | MR Egger | 0.090 | 0.071 | -0.049 | 0.229 | 2.05e-01 |
| node_pheno53 | adulthood_BMI | Weighted median | 0.025 | 0.039 | -0.051 | 0.102 | 5.22e-01 |
| node_pheno53 | adulthood_BMI | Simple mode | 0.101 | 0.125 | -0.144 | 0.346 | 4.20e-01 |
| node_pheno53 | adulthood_BMI | Weighted mode | 0.064 | 0.074 | -0.082 | 0.209 | 3.93e-01 |
| node_pheno54 | adulthood_BMI | MR Egger | 0.045 | 0.067 | -0.086 | 0.176 | 5.00e-01 |
| node_pheno54 | adulthood_BMI | Weighted median | 0.022 | 0.038 | -0.052 | 0.096 | 5.53e-01 |
| node_pheno54 | adulthood_BMI | Simple mode | -0.041 | 0.133 | -0.302 | 0.220 | 7.59e-01 |
| node_pheno54 | adulthood_BMI | Weighted mode | 0.001 | 0.095 | -0.186 | 0.188 | 9.95e-01 |
| node_pheno55 | adulthood_BMI | MR Egger | 0.099 | 0.068 | -0.035 | 0.232 | 1.49e-01 |
| node_pheno55 | adulthood_BMI | Weighted median | 0.020 | 0.037 | -0.053 | 0.094 | 5.87e-01 |
| node_pheno55 | adulthood_BMI | Simple mode | -0.057 | 0.134 | -0.319 | 0.204 | 6.68e-01 |
| node_pheno55 | adulthood_BMI | Weighted mode | -0.014 | 0.081 | -0.172 | 0.145 | 8.64e-01 |
| node_pheno56 | adulthood_BMI | MR Egger | 0.096 | 0.069 | -0.039 | 0.232 | 1.65e-01 |
| node_pheno56 | adulthood_BMI | Weighted median | 0.039 | 0.038 | -0.036 | 0.113 | 3.09e-01 |
| node_pheno56 | adulthood_BMI | Simple mode | 0.026 | 0.118 | -0.205 | 0.257 | 8.25e-01 |
| node_pheno56 | adulthood_BMI | Weighted mode | 0.015 | 0.068 | -0.118 | 0.147 | 8.29e-01 |
| node_pheno57 | adulthood_BMI | MR Egger | 0.064 | 0.064 | -0.063 | 0.190 | 3.23e-01 |
| node_pheno57 | adulthood_BMI | Weighted median | 0.043 | 0.036 | -0.027 | 0.114 | 2.30e-01 |
| node_pheno57 | adulthood_BMI | Simple mode | 0.240 | 0.142 | -0.040 | 0.519 | 9.32e-02 |
| node_pheno57 | adulthood_BMI | Weighted mode | -0.172 | 0.107 | -0.381 | 0.037 | 1.07e-01 |
| node_pheno58 | adulthood_BMI | MR Egger | 0.103 | 0.069 | -0.033 | 0.239 | 1.38e-01 |
| node_pheno58 | adulthood_BMI | Weighted median | 0.058 | 0.037 | -0.015 | 0.130 | 1.21e-01 |
| node_pheno58 | adulthood_BMI | Simple mode | 0.078 | 0.134 | -0.184 | 0.341 | 5.58e-01 |
| node_pheno58 | adulthood_BMI | Weighted mode | 0.043 | 0.086 | -0.126 | 0.213 | 6.17e-01 |
| node_pheno59 | adulthood_BMI | MR Egger | 0.064 | 0.060 | -0.054 | 0.182 | 2.87e-01 |
| node_pheno59 | adulthood_BMI | Weighted median | 0.069 | 0.034 | 0.002 | 0.135 | 4.26e-02 |
| node_pheno59 | adulthood_BMI | Simple mode | 0.162 | 0.117 | -0.068 | 0.393 | 1.67e-01 |
| node_pheno59 | adulthood_BMI | Weighted mode | 0.111 | 0.077 | -0.041 | 0.262 | 1.53e-01 |
| node_pheno60 | adulthood_BMI | MR Egger | 0.060 | 0.066 | -0.070 | 0.190 | 3.64e-01 |
| node_pheno60 | adulthood_BMI | Weighted median | 0.014 | 0.038 | -0.061 | 0.089 | 7.16e-01 |
| node_pheno60 | adulthood_BMI | Simple mode | 0.051 | 0.136 | -0.216 | 0.318 | 7.07e-01 |
| node_pheno60 | adulthood_BMI | Weighted mode | -0.026 | 0.080 | -0.184 | 0.131 | 7.42e-01 |
| node_pheno61 | adulthood_BMI | MR Egger | 0.090 | 0.065 | -0.037 | 0.216 | 1.67e-01 |
| node_pheno61 | adulthood_BMI | Weighted median | 0.092 | 0.039 | 0.016 | 0.169 | 1.83e-02 |
| node_pheno61 | adulthood_BMI | Simple mode | 0.163 | 0.118 | -0.068 | 0.394 | 1.68e-01 |
| node_pheno61 | adulthood_BMI | Weighted mode | 0.151 | 0.092 | -0.030 | 0.332 | 1.02e-01 |
| node_pheno62 | adulthood_BMI | MR Egger | 0.128 | 0.067 | -0.003 | 0.258 | 5.63e-02 |
| node_pheno62 | adulthood_BMI | Weighted median | 0.071 | 0.037 | -0.002 | 0.144 | 5.57e-02 |
| node_pheno62 | adulthood_BMI | Simple mode | 0.180 | 0.130 | -0.075 | 0.435 | 1.66e-01 |
| node_pheno62 | adulthood_BMI | Weighted mode | 0.124 | 0.108 | -0.087 | 0.336 | 2.51e-01 |
| node_pheno63 | adulthood_BMI | MR Egger | 0.062 | 0.067 | -0.069 | 0.192 | 3.53e-01 |
| node_pheno63 | adulthood_BMI | Weighted median | 0.047 | 0.038 | -0.028 | 0.121 | 2.19e-01 |
| node_pheno63 | adulthood_BMI | Simple mode | -0.036 | 0.129 | -0.289 | 0.217 | 7.81e-01 |
| node_pheno63 | adulthood_BMI | Weighted mode | 0.005 | 0.087 | -0.166 | 0.175 | 9.57e-01 |
| node_pheno64 | adulthood_BMI | MR Egger | 0.134 | 0.062 | 0.012 | 0.256 | 3.13e-02 |
| node_pheno64 | adulthood_BMI | Weighted median | 0.030 | 0.036 | -0.040 | 0.099 | 4.07e-01 |
| node_pheno64 | adulthood_BMI | Simple mode | -0.016 | 0.119 | -0.249 | 0.217 | 8.95e-01 |
| node_pheno64 | adulthood_BMI | Weighted mode | -0.016 | 0.074 | -0.161 | 0.129 | 8.32e-01 |
| node_pheno65 | adulthood_BMI | MR Egger | 0.071 | 0.069 | -0.064 | 0.207 | 3.03e-01 |
| node_pheno65 | adulthood_BMI | Weighted median | 0.057 | 0.036 | -0.015 | 0.128 | 1.19e-01 |
| node_pheno65 | adulthood_BMI | Simple mode | 0.249 | 0.149 | -0.043 | 0.541 | 9.48e-02 |
| node_pheno65 | adulthood_BMI | Weighted mode | 0.193 | 0.129 | -0.059 | 0.445 | 1.35e-01 |
| node_pheno66 | adulthood_BMI | MR Egger | 0.071 | 0.067 | -0.060 | 0.202 | 2.87e-01 |
| node_pheno66 | adulthood_BMI | Weighted median | -0.012 | 0.039 | -0.089 | 0.065 | 7.62e-01 |
| node_pheno66 | adulthood_BMI | Simple mode | -0.142 | 0.143 | -0.423 | 0.138 | 3.20e-01 |
| node_pheno66 | adulthood_BMI | Weighted mode | -0.119 | 0.095 | -0.305 | 0.067 | 2.11e-01 |
| node_pheno67 | adulthood_BMI | MR Egger | 0.108 | 0.067 | -0.022 | 0.238 | 1.05e-01 |
| node_pheno67 | adulthood_BMI | Weighted median | 0.033 | 0.039 | -0.043 | 0.109 | 3.93e-01 |
| node_pheno67 | adulthood_BMI | Simple mode | -0.044 | 0.129 | -0.297 | 0.209 | 7.34e-01 |
| node_pheno67 | adulthood_BMI | Weighted mode | 0.048 | 0.087 | -0.124 | 0.219 | 5.87e-01 |
| node_pheno68 | adulthood_BMI | MR Egger | 0.082 | 0.064 | -0.043 | 0.206 | 1.99e-01 |
| node_pheno68 | adulthood_BMI | Weighted median | 0.065 | 0.037 | -0.007 | 0.137 | 7.89e-02 |
| node_pheno68 | adulthood_BMI | Simple mode | 0.182 | 0.128 | -0.069 | 0.434 | 1.56e-01 |
| node_pheno68 | adulthood_BMI | Weighted mode | 0.216 | 0.101 | 0.018 | 0.415 | 3.29e-02 |
| node_pheno69 | adulthood_BMI | MR Egger | 0.118 | 0.066 | -0.012 | 0.248 | 7.57e-02 |
| node_pheno69 | adulthood_BMI | Weighted median | 0.047 | 0.038 | -0.028 | 0.122 | 2.22e-01 |
| node_pheno69 | adulthood_BMI | Simple mode | 0.187 | 0.119 | -0.046 | 0.419 | 1.16e-01 |
| node_pheno69 | adulthood_BMI | Weighted mode | 0.103 | 0.077 | -0.048 | 0.253 | 1.83e-01 |
| node_pheno7 | adulthood_BMI | MR Egger | 0.086 | 0.073 | -0.056 | 0.228 | 2.37e-01 |
| node_pheno7 | adulthood_BMI | Weighted median | 0.020 | 0.040 | -0.059 | 0.099 | 6.23e-01 |
| node_pheno7 | adulthood_BMI | Simple mode | 0.284 | 0.183 | -0.075 | 0.643 | 1.21e-01 |
| node_pheno7 | adulthood_BMI | Weighted mode | -0.262 | 0.173 | -0.602 | 0.078 | 1.31e-01 |
| node_pheno70 | adulthood_BMI | MR Egger | 0.097 | 0.064 | -0.029 | 0.222 | 1.31e-01 |
| node_pheno70 | adulthood_BMI | Weighted median | 0.013 | 0.036 | -0.058 | 0.083 | 7.27e-01 |
| node_pheno70 | adulthood_BMI | Simple mode | 0.105 | 0.140 | -0.170 | 0.380 | 4.54e-01 |
| node_pheno70 | adulthood_BMI | Weighted mode | -0.145 | 0.102 | -0.344 | 0.054 | 1.55e-01 |
| node_pheno71 | adulthood_BMI | MR Egger | 0.034 | 0.064 | -0.091 | 0.159 | 5.94e-01 |
| node_pheno71 | adulthood_BMI | Weighted median | 0.016 | 0.037 | -0.057 | 0.090 | 6.66e-01 |
| node_pheno71 | adulthood_BMI | Simple mode | 0.088 | 0.126 | -0.159 | 0.336 | 4.84e-01 |
| node_pheno71 | adulthood_BMI | Weighted mode | 0.010 | 0.092 | -0.171 | 0.192 | 9.11e-01 |
| node_pheno72 | adulthood_BMI | MR Egger | 0.101 | 0.059 | -0.014 | 0.215 | 8.65e-02 |
| node_pheno72 | adulthood_BMI | Weighted median | 0.049 | 0.034 | -0.017 | 0.115 | 1.48e-01 |
| node_pheno72 | adulthood_BMI | Simple mode | 0.088 | 0.105 | -0.118 | 0.294 | 4.03e-01 |
| node_pheno72 | adulthood_BMI | Weighted mode | 0.042 | 0.068 | -0.090 | 0.175 | 5.33e-01 |
| node_pheno73 | adulthood_BMI | MR Egger | 0.141 | 0.062 | 0.019 | 0.263 | 2.39e-02 |
| node_pheno73 | adulthood_BMI | Weighted median | 0.059 | 0.035 | -0.009 | 0.128 | 9.04e-02 |
| node_pheno73 | adulthood_BMI | Simple mode | 0.164 | 0.124 | -0.079 | 0.406 | 1.86e-01 |
| node_pheno73 | adulthood_BMI | Weighted mode | 0.022 | 0.071 | -0.118 | 0.161 | 7.60e-01 |
| node_pheno74 | adulthood_BMI | MR Egger | 0.118 | 0.067 | -0.014 | 0.250 | 8.06e-02 |
| node_pheno74 | adulthood_BMI | Weighted median | 0.012 | 0.039 | -0.064 | 0.089 | 7.54e-01 |
| node_pheno74 | adulthood_BMI | Simple mode | 0.033 | 0.131 | -0.224 | 0.291 | 7.99e-01 |
| node_pheno74 | adulthood_BMI | Weighted mode | -0.019 | 0.079 | -0.175 | 0.136 | 8.08e-01 |
| node_pheno75 | adulthood_BMI | MR Egger | 0.057 | 0.059 | -0.059 | 0.172 | 3.36e-01 |
| node_pheno75 | adulthood_BMI | Weighted median | 0.038 | 0.034 | -0.030 | 0.105 | 2.73e-01 |
| node_pheno75 | adulthood_BMI | Simple mode | 0.120 | 0.124 | -0.123 | 0.363 | 3.34e-01 |
| node_pheno75 | adulthood_BMI | Weighted mode | 0.069 | 0.077 | -0.082 | 0.221 | 3.70e-01 |
| node_pheno76 | adulthood_BMI | MR Egger | 0.133 | 0.060 | 0.016 | 0.250 | 2.59e-02 |
| node_pheno76 | adulthood_BMI | Weighted median | 0.091 | 0.034 | 0.025 | 0.157 | 6.73e-03 |
| node_pheno76 | adulthood_BMI | Simple mode | 0.105 | 0.132 | -0.154 | 0.364 | 4.28e-01 |
| node_pheno76 | adulthood_BMI | Weighted mode | 0.147 | 0.098 | -0.046 | 0.339 | 1.37e-01 |
| node_pheno8 | adulthood_BMI | MR Egger | 0.050 | 0.066 | -0.079 | 0.179 | 4.50e-01 |
| node_pheno8 | adulthood_BMI | Weighted median | -0.008 | 0.038 | -0.084 | 0.067 | 8.27e-01 |
| node_pheno8 | adulthood_BMI | Simple mode | 0.000 | 0.138 | -0.271 | 0.271 | 9.99e-01 |
| node_pheno8 | adulthood_BMI | Weighted mode | -0.011 | 0.101 | -0.210 | 0.187 | 9.12e-01 |
| node_pheno9 | adulthood_BMI | MR Egger | 0.039 | 0.071 | -0.101 | 0.179 | 5.83e-01 |
| node_pheno9 | adulthood_BMI | Weighted median | 0.037 | 0.040 | -0.041 | 0.115 | 3.56e-01 |
| node_pheno9 | adulthood_BMI | Simple mode | 0.041 | 0.126 | -0.205 | 0.288 | 7.42e-01 |
| node_pheno9 | adulthood_BMI | Weighted mode | 0.019 | 0.084 | -0.146 | 0.184 | 8.22e-01 |

**Table S5. Heterogeneity and pleiotropy test results from MR-Egger regression analysis.**

| **exposure** | **outcome** | **Heterogeneity test** | |  | **Pleiotropy test** | | |
| --- | --- | --- | --- | --- | --- | --- | --- |
|  |  | **Cochran's *Q*** | **p*-*value** |  | **intercept** | **se** | **p*-*value** |
| edge_pheno101 | birth weight | 126.2 | 3.32E-01 |  | 0.0008 | 0.0027 | 7.67E-01 |
| edge_pheno1013 | birth weight | 124.7 | 3.67E-01 |  | 0.0047 | 0.0027 | 7.91E-02 |
| edge_pheno102 | birth weight | 132.5 | 2.06E-01 |  | -0.0020 | 0.0027 | 4.72E-01 |
| edge_pheno1020 | birth weight | 102.9 | 8.67E-01 |  | -0.0015 | 0.0026 | 5.62E-01 |
| edge_pheno1022 | birth weight | 115.7 | 5.94E-01 |  | -0.0001 | 0.0026 | 9.59E-01 |
| edge_pheno103 | birth weight | 161.4 | 6.98E-03 |  | 0.0002 | 0.0030 | 9.43E-01 |
| edge_pheno1041 | birth weight | 163.8 | 4.89E-03 |  | -0.0028 | 0.0030 | 3.66E-01 |
| edge_pheno1059 | birth weight | 161.6 | 6.77E-03 |  | 0.0021 | 0.0030 | 4.89E-01 |
| edge_pheno1122 | birth weight | 157.1 | 1.31E-02 |  | 0.0007 | 0.0030 | 8.23E-01 |
| edge_pheno1126 | birth weight | 136.1 | 1.50E-01 |  | 0.0050 | 0.0027 | 7.09E-02 |
| edge_pheno1134 | birth weight | 169.2 | 2.08E-03 |  | 0.0060 | 0.0030 | 5.17E-02 |
| edge_pheno1137 | birth weight | 155.4 | 1.65E-02 |  | -0.0006 | 0.0030 | 8.49E-01 |
| edge_pheno1141 | birth weight | 139.2 | 1.11E-01 |  | -0.0002 | 0.0028 | 9.54E-01 |
| edge_pheno1142 | birth weight | 122.6 | 4.16E-01 |  | 0.0041 | 0.0026 | 1.19E-01 |
| edge_pheno1161 | birth weight | 179.7 | 3.45E-04 |  | -0.0006 | 0.0031 | 8.49E-01 |
| edge_pheno1167 | birth weight | 171.2 | 1.51E-03 |  | 0.0009 | 0.0031 | 7.71E-01 |
| edge_pheno1171 | birth weight | 151.1 | 2.49E-02 |  | -0.0030 | 0.0029 | 3.10E-01 |
| edge_pheno1175 | birth weight | 169.0 | 2.14E-03 |  | -0.0026 | 0.0031 | 4.13E-01 |
| edge_pheno1183 | birth weight | 120.3 | 4.76E-01 |  | 0.0011 | 0.0026 | 6.75E-01 |
| edge_pheno1184 | birth weight | 173.7 | 9.83E-04 |  | 0.0047 | 0.0032 | 1.36E-01 |
| edge_pheno1189 | birth weight | 150.2 | 3.25E-02 |  | 0.0023 | 0.0029 | 4.34E-01 |
| edge_pheno1205 | birth weight | 139.3 | 1.10E-01 |  | 0.0011 | 0.0028 | 6.94E-01 |
| edge_pheno1211 | birth weight | 147.3 | 4.60E-02 |  | 0.0041 | 0.0029 | 1.53E-01 |
| edge_pheno1221 | birth weight | 160.9 | 7.54E-03 |  | 0.0004 | 0.0030 | 8.96E-01 |
| edge_pheno1225 | birth weight | 130.9 | 2.33E-01 |  | 0.0000 | 0.0027 | 9.95E-01 |
| edge_pheno1250 | birth weight | 161.2 | 7.21E-03 |  | -0.0005 | 0.0030 | 8.55E-01 |
| edge_pheno1256 | birth weight | 139.4 | 1.09E-01 |  | 0.0008 | 0.0028 | 7.83E-01 |
| edge_pheno1257 | birth weight | 125.1 | 3.57E-01 |  | 0.0026 | 0.0026 | 3.26E-01 |
| edge_pheno1269 | birth weight | 121.9 | 4.34E-01 |  | 0.0005 | 0.0026 | 8.57E-01 |
| edge_pheno1270 | birth weight | 155.6 | 1.59E-02 |  | -0.0028 | 0.0029 | 3.39E-01 |
| edge_pheno1273 | birth weight | 151.9 | 2.61E-02 |  | -0.0014 | 0.0029 | 6.36E-01 |
| edge_pheno1276 | birth weight | 158.6 | 1.05E-02 |  | 0.0003 | 0.0029 | 9.08E-01 |
| edge_pheno1293 | birth weight | 178.8 | 4.01E-04 |  | 0.0014 | 0.0032 | 6.60E-01 |
| edge_pheno1296 | birth weight | 140.6 | 9.58E-02 |  | -0.0007 | 0.0028 | 8.06E-01 |
| edge_pheno1300 | birth weight | 158.9 | 1.01E-02 |  | -0.0011 | 0.0030 | 7.14E-01 |
| edge_pheno1301 | birth weight | 124.5 | 3.70E-01 |  | -0.0045 | 0.0027 | 9.16E-02 |
| edge_pheno1302 | birth weight | 125.9 | 3.38E-01 |  | 0.0020 | 0.0026 | 4.54E-01 |
| edge_pheno1309 | birth weight | 150.8 | 2.99E-02 |  | -0.0017 | 0.0029 | 5.73E-01 |
| edge_pheno1311 | birth weight | 71.2 | 7.49E-01 |  | 0.0015 | 0.0030 | 6.15E-01 |
| edge_pheno1317 | birth weight | 107.9 | 1.72E-02 |  | 0.0016 | 0.0035 | 6.56E-01 |
| edge_pheno1319 | birth weight | 95.0 | 9.55E-01 |  | 0.0033 | 0.0026 | 2.06E-01 |
| edge_pheno132 | birth weight | 140.8 | 9.40E-02 |  | -0.0015 | 0.0028 | 5.94E-01 |
| edge_pheno1322 | birth weight | 132.0 | 2.13E-01 |  | -0.0009 | 0.0027 | 7.39E-01 |
| edge_pheno1325 | birth weight | 136.9 | 1.39E-01 |  | 0.0010 | 0.0028 | 7.32E-01 |
| edge_pheno1328 | birth weight | 166.1 | 3.43E-03 |  | -0.0004 | 0.0031 | 9.08E-01 |
| edge_pheno135 | birth weight | 131.1 | 2.30E-01 |  | 0.0038 | 0.0027 | 1.70E-01 |
| edge_pheno1359 | birth weight | 165.3 | 3.90E-03 |  | -0.0005 | 0.0030 | 8.58E-01 |
| edge_pheno1382 | birth weight | 149.5 | 3.50E-02 |  | 0.0009 | 0.0029 | 7.59E-01 |
| edge_pheno146 | birth weight | 134.9 | 1.66E-01 |  | 0.0009 | 0.0028 | 7.45E-01 |
| edge_pheno147 | birth weight | 125.1 | 3.56E-01 |  | -0.0013 | 0.0027 | 6.40E-01 |
| edge_pheno151 | birth weight | 134.7 | 1.70E-01 |  | -0.0014 | 0.0027 | 6.04E-01 |
| edge_pheno1696 | birth weight | 157.5 | 1.23E-02 |  | -0.0021 | 0.0030 | 4.73E-01 |
| edge_pheno1697 | birth weight | 215.2 | 2.17E-07 |  | -0.0002 | 0.0034 | 9.60E-01 |
| edge_pheno1698 | birth weight | 177.1 | 5.48E-04 |  | 0.0003 | 0.0032 | 9.15E-01 |
| edge_pheno1699 | birth weight | 219.2 | 8.60E-08 |  | 0.0003 | 0.0032 | 9.35E-01 |
| edge_pheno1701 | birth weight | 187.1 | 8.68E-05 |  | -0.0009 | 0.0032 | 7.66E-01 |
| edge_pheno249 | birth weight | 150.0 | 3.29E-02 |  | 0.0000 | 0.0029 | 9.92E-01 |
| edge_pheno253 | birth weight | 114.1 | 6.34E-01 |  | 0.0005 | 0.0026 | 8.54E-01 |
| edge_pheno262 | birth weight | 95.2 | 9.54E-01 |  | -0.0010 | 0.0026 | 7.00E-01 |
| edge_pheno286 | birth weight | 134.8 | 1.69E-01 |  | -0.0006 | 0.0027 | 8.20E-01 |
| edge_pheno288 | birth weight | 96.7 | 9.42E-01 |  | 0.0029 | 0.0026 | 2.70E-01 |
| edge_pheno303 | birth weight | 147.2 | 4.64E-02 |  | 0.0038 | 0.0029 | 1.90E-01 |
| edge_pheno389 | birth weight | 129.4 | 2.63E-01 |  | -0.0026 | 0.0027 | 3.36E-01 |
| edge_pheno405 | birth weight | 99.1 | 9.18E-01 |  | -0.0004 | 0.0026 | 8.66E-01 |
| edge_pheno447 | birth weight | 7.6 | 3.68E-01 |  | -0.0050 | 0.0130 | 7.11E-01 |
| edge_pheno449 | birth weight | 2.2 | 8.18E-01 |  | -0.0012 | 0.0151 | 9.38E-01 |
| edge_pheno460 | birth weight | 153.3 | 2.17E-02 |  | -0.0038 | 0.0029 | 2.05E-01 |
| edge_pheno461 | birth weight | 152.4 | 2.43E-02 |  | 0.0026 | 0.0029 | 3.83E-01 |
| edge_pheno491 | birth weight | 126.5 | 3.25E-01 |  | -0.0011 | 0.0027 | 6.83E-01 |
| edge_pheno537 | birth weight | 155.0 | 1.73E-02 |  | 0.0011 | 0.0029 | 7.14E-01 |
| edge_pheno55 | birth weight | 153.4 | 2.16E-02 |  | 0.0002 | 0.0030 | 9.47E-01 |
| edge_pheno558 | birth weight | 134.3 | 1.76E-01 |  | -0.0007 | 0.0027 | 8.12E-01 |
| edge_pheno574 | birth weight | 123.9 | 3.85E-01 |  | 0.0008 | 0.0026 | 7.69E-01 |
| edge_pheno58 | birth weight | 115.2 | 6.06E-01 |  | -0.0001 | 0.0025 | 9.70E-01 |
| edge_pheno590 | birth weight | 148.4 | 4.02E-02 |  | 0.0017 | 0.0029 | 5.59E-01 |
| edge_pheno593 | birth weight | 133.9 | 1.82E-01 |  | 0.0008 | 0.0027 | 7.77E-01 |
| edge_pheno597 | birth weight | 174.8 | 8.14E-04 |  | 0.0001 | 0.0031 | 9.82E-01 |
| edge_pheno599 | birth weight | 126.8 | 3.17E-01 |  | 0.0046 | 0.0027 | 9.17E-02 |
| edge_pheno601 | birth weight | 124.3 | 3.75E-01 |  | 0.0001 | 0.0026 | 9.59E-01 |
| edge_pheno606 | birth weight | 126.1 | 3.33E-01 |  | -0.0043 | 0.0027 | 1.11E-01 |
| edge_pheno609 | birth weight | 148.2 | 4.14E-02 |  | 0.0015 | 0.0029 | 6.04E-01 |
| edge_pheno621 | birth weight | 131.4 | 2.25E-01 |  | -0.0009 | 0.0027 | 7.50E-01 |
| edge_pheno624 | birth weight | 125.1 | 3.56E-01 |  | 0.0006 | 0.0027 | 8.36E-01 |
| edge_pheno636 | birth weight | 118.9 | 5.11E-01 |  | -0.0004 | 0.0026 | 8.71E-01 |
| edge_pheno639 | birth weight | 134.6 | 1.71E-01 |  | -0.0014 | 0.0027 | 6.16E-01 |
| edge_pheno65 | birth weight | 160.3 | 8.19E-03 |  | -0.0021 | 0.0030 | 4.82E-01 |
| edge_pheno66 | birth weight | 152.5 | 2.41E-02 |  | 0.0013 | 0.0029 | 6.61E-01 |
| edge_pheno674 | birth weight | 133.1 | 1.96E-01 |  | -0.0029 | 0.0027 | 2.91E-01 |
| edge_pheno681 | birth weight | 158.0 | 1.14E-02 |  | -0.0008 | 0.0030 | 7.79E-01 |
| edge_pheno683 | birth weight | 150.7 | 3.03E-02 |  | 0.0040 | 0.0029 | 1.76E-01 |
| edge_pheno695 | birth weight | 132.6 | 2.03E-01 |  | 0.0026 | 0.0027 | 3.52E-01 |
| edge_pheno698 | birth weight | 126.5 | 3.24E-01 |  | 0.0003 | 0.0027 | 9.21E-01 |
| edge_pheno705 | birth weight | 149.7 | 3.44E-02 |  | 0.0002 | 0.0029 | 9.52E-01 |
| edge_pheno716 | birth weight | 131.8 | 2.17E-01 |  | -0.0019 | 0.0027 | 4.83E-01 |
| edge_pheno767 | birth weight | 146.3 | 5.17E-02 |  | -0.0037 | 0.0029 | 2.03E-01 |
| edge_pheno777 | birth weight | 147.8 | 4.31E-02 |  | 0.0024 | 0.0029 | 3.98E-01 |
| edge_pheno789 | birth weight | 137.2 | 1.35E-01 |  | 0.0002 | 0.0027 | 9.28E-01 |
| edge_pheno794 | birth weight | 128.7 | 2.78E-01 |  | -0.0007 | 0.0027 | 8.00E-01 |
| edge_pheno801 | birth weight | 126.3 | 3.30E-01 |  | -0.0010 | 0.0027 | 7.08E-01 |
| edge_pheno810 | birth weight | 148.2 | 4.14E-02 |  | 0.0011 | 0.0028 | 6.84E-01 |
| edge_pheno812 | birth weight | 130.3 | 2.45E-01 |  | 0.0006 | 0.0027 | 8.23E-01 |
| edge_pheno815 | birth weight | 186.8 | 9.21E-05 |  | -0.0046 | 0.0032 | 1.57E-01 |
| edge_pheno816 | birth weight | 189.8 | 5.14E-05 |  | -0.0009 | 0.0032 | 7.79E-01 |
| edge_pheno824 | birth weight | 167.2 | 2.90E-03 |  | -0.0015 | 0.0030 | 6.26E-01 |
| edge_pheno867 | birth weight | 145.7 | 5.53E-02 |  | -0.0042 | 0.0029 | 1.44E-01 |
| edge_pheno869 | birth weight | 179.4 | 3.64E-04 |  | -0.0031 | 0.0032 | 3.36E-01 |
| edge_pheno87 | birth weight | 164.4 | 4.49E-03 |  | 0.0028 | 0.0031 | 3.56E-01 |
| edge_pheno882 | birth weight | 131.3 | 2.26E-01 |  | -0.0007 | 0.0028 | 7.99E-01 |
| edge_pheno899 | birth weight | 187.3 | 8.28E-05 |  | -0.0047 | 0.0033 | 1.54E-01 |
| edge_pheno903 | birth weight | 148.5 | 3.97E-02 |  | -0.0031 | 0.0029 | 2.89E-01 |
| edge_pheno908 | birth weight | 136.7 | 1.41E-01 |  | 0.0002 | 0.0028 | 9.54E-01 |
| edge_pheno918 | birth weight | 160.5 | 7.98E-03 |  | 0.0001 | 0.0030 | 9.77E-01 |
| edge_pheno932 | birth weight | 142.5 | 7.88E-02 |  | -0.0015 | 0.0028 | 5.89E-01 |
| edge_pheno942 | birth weight | 160.8 | 7.62E-03 |  | 0.0045 | 0.0030 | 1.37E-01 |
| edge_pheno956 | birth weight | 148.9 | 3.79E-02 |  | 0.0002 | 0.0029 | 9.42E-01 |
| edge_pheno965 | birth weight | 132.1 | 2.13E-01 |  | -0.0032 | 0.0027 | 2.42E-01 |
| node_pheno1 | birth weight | 169.7 | 1.93E-03 |  | 0.0026 | 0.0030 | 3.93E-01 |
| node_pheno10 | birth weight | 137.0 | 1.37E-01 |  | -0.0009 | 0.0027 | 7.33E-01 |
| node_pheno11 | birth weight | 125.1 | 3.57E-01 |  | -0.0017 | 0.0026 | 5.19E-01 |
| node_pheno12 | birth weight | 148.9 | 3.78E-02 |  | -0.0003 | 0.0029 | 9.11E-01 |
| node_pheno13 | birth weight | 152.6 | 2.38E-02 |  | 0.0015 | 0.0029 | 5.99E-01 |
| node_pheno14 | birth weight | 141.1 | 9.12E-02 |  | 0.0002 | 0.0027 | 9.48E-01 |
| node_pheno15 | birth weight | 119.2 | 5.03E-01 |  | 0.0028 | 0.0025 | 2.70E-01 |
| node_pheno16 | birth weight | 125.1 | 3.56E-01 |  | 0.0006 | 0.0025 | 8.02E-01 |
| node_pheno17 | birth weight | 141.9 | 8.43E-02 |  | 0.0004 | 0.0028 | 8.77E-01 |
| node_pheno18 | birth weight | 154.5 | 1.85E-02 |  | 0.0011 | 0.0028 | 7.06E-01 |
| node_pheno19 | birth weight | 122.8 | 4.11E-01 |  | -0.0024 | 0.0025 | 3.36E-01 |
| node_pheno2 | birth weight | 120.8 | 4.63E-01 |  | 0.0033 | 0.0025 | 1.96E-01 |
| node_pheno20 | birth weight | 141.5 | 8.74E-02 |  | -0.0039 | 0.0027 | 1.55E-01 |
| node_pheno21 | birth weight | 133.9 | 1.82E-01 |  | 0.0048 | 0.0027 | 7.91E-02 |
| node_pheno22 | birth weight | 143.4 | 7.19E-02 |  | 0.0004 | 0.0027 | 8.70E-01 |
| node_pheno23 | birth weight | 187.8 | 7.50E-05 |  | 0.0004 | 0.0030 | 8.82E-01 |
| node_pheno24 | birth weight | 154.7 | 1.80E-02 |  | 0.0006 | 0.0028 | 8.29E-01 |
| node_pheno25 | birth weight | 158.3 | 1.09E-02 |  | 0.0032 | 0.0028 | 2.63E-01 |
| node_pheno26 | birth weight | 137.1 | 1.36E-01 |  | 0.0041 | 0.0025 | 1.09E-01 |
| node_pheno27 | birth weight | 150.3 | 3.20E-02 |  | 0.0004 | 0.0029 | 8.78E-01 |
| node_pheno28 | birth weight | 130.5 | 2.41E-01 |  | 0.0010 | 0.0025 | 6.93E-01 |
| node_pheno29 | birth weight | 144.3 | 6.49E-02 |  | -0.0015 | 0.0027 | 5.74E-01 |
| node_pheno3 | birth weight | 181.1 | 2.65E-04 |  | 0.0024 | 0.0031 | 4.41E-01 |
| node_pheno30 | birth weight | 167.5 | 2.75E-03 |  | -0.0005 | 0.0029 | 8.67E-01 |
| node_pheno31 | birth weight | 131.3 | 2.27E-01 |  | 0.0003 | 0.0026 | 9.20E-01 |
| node_pheno32 | birth weight | 144.4 | 6.39E-02 |  | 0.0013 | 0.0027 | 6.34E-01 |
| node_pheno33 | birth weight | 153.5 | 2.12E-02 |  | 0.0013 | 0.0028 | 6.54E-01 |
| node_pheno34 | birth weight | 182.5 | 2.04E-04 |  | 0.0017 | 0.0030 | 5.59E-01 |
| node_pheno35 | birth weight | 110.6 | 7.20E-01 |  | -0.0007 | 0.0024 | 7.71E-01 |
| node_pheno36 | birth weight | 145.7 | 5.51E-02 |  | -0.0006 | 0.0026 | 8.33E-01 |
| node_pheno37 | birth weight | 109.3 | 7.49E-01 |  | -0.0028 | 0.0025 | 2.63E-01 |
| node_pheno38 | birth weight | 146.3 | 5.16E-02 |  | 0.0020 | 0.0026 | 4.42E-01 |
| node_pheno39 | birth weight | 172.1 | 1.29E-03 |  | -0.0016 | 0.0029 | 5.90E-01 |
| node_pheno4 | birth weight | 136.7 | 1.42E-01 |  | 0.0023 | 0.0027 | 4.05E-01 |
| node_pheno40 | birth weight | 156.8 | 1.36E-02 |  | -0.0048 | 0.0028 | 8.63E-02 |
| node_pheno41 | birth weight | 160.2 | 8.36E-03 |  | -0.0001 | 0.0030 | 9.68E-01 |
| node_pheno42 | birth weight | 196.1 | 1.42E-05 |  | 0.0015 | 0.0031 | 6.36E-01 |
| node_pheno43 | birth weight | 156.3 | 1.44E-02 |  | 0.0015 | 0.0028 | 5.92E-01 |
| node_pheno44 | birth weight | 131.4 | 2.24E-01 |  | 0.0004 | 0.0025 | 8.74E-01 |
| node_pheno45 | birth weight | 169.4 | 2.02E-03 |  | -0.0032 | 0.0029 | 2.68E-01 |
| node_pheno46 | birth weight | 208.6 | 9.88E-07 |  | 0.0001 | 0.0033 | 9.82E-01 |
| node_pheno47 | birth weight | 148.2 | 4.14E-02 |  | 0.0012 | 0.0028 | 6.73E-01 |
| node_pheno48 | birth weight | 177.0 | 5.58E-04 |  | -0.0002 | 0.0031 | 9.46E-01 |
| node_pheno49 | birth weight | 152.5 | 2.42E-02 |  | 0.0006 | 0.0028 | 8.24E-01 |
| node_pheno5 | birth weight | 114.4 | 6.26E-01 |  | 0.0010 | 0.0026 | 7.09E-01 |
| node_pheno50 | birth weight | 125.0 | 3.59E-01 |  | 0.0011 | 0.0025 | 6.67E-01 |
| node_pheno51 | birth weight | 144.5 | 6.35E-02 |  | -0.0016 | 0.0028 | 5.69E-01 |
| node_pheno52 | birth weight | 145.9 | 5.43E-02 |  | 0.0038 | 0.0027 | 1.53E-01 |
| node_pheno53 | birth weight | 158.6 | 1.05E-02 |  | 0.0015 | 0.0029 | 6.20E-01 |
| node_pheno54 | birth weight | 136.4 | 1.46E-01 |  | 0.0055 | 0.0026 | 3.97E-02 |
| node_pheno55 | birth weight | 156.9 | 1.33E-02 |  | -0.0039 | 0.0028 | 1.76E-01 |
| node_pheno56 | birth weight | 200.0 | 6.31E-06 |  | -0.0010 | 0.0032 | 7.62E-01 |
| node_pheno57 | birth weight | 131.3 | 2.26E-01 |  | 0.0023 | 0.0025 | 3.71E-01 |
| node_pheno58 | birth weight | 140.1 | 1.02E-01 |  | -0.0001 | 0.0027 | 9.78E-01 |
| node_pheno59 | birth weight | 165.7 | 3.67E-03 |  | 0.0009 | 0.0025 | 7.28E-01 |
| node_pheno60 | birth weight | 179.4 | 3.64E-04 |  | -0.0009 | 0.0030 | 7.58E-01 |
| node_pheno61 | birth weight | 180.6 | 2.92E-04 |  | 0.0003 | 0.0030 | 9.11E-01 |
| node_pheno62 | birth weight | 140.3 | 9.96E-02 |  | 0.0030 | 0.0027 | 2.75E-01 |
| node_pheno63 | birth weight | 138.5 | 1.18E-01 |  | -0.0004 | 0.0026 | 8.69E-01 |
| node_pheno64 | birth weight | 174.1 | 9.26E-04 |  | 0.0016 | 0.0028 | 5.64E-01 |
| node_pheno65 | birth weight | 150.6 | 3.05E-02 |  | 0.0040 | 0.0027 | 1.35E-01 |
| node_pheno66 | birth weight | 151.1 | 2.86E-02 |  | -0.0017 | 0.0027 | 5.45E-01 |
| node_pheno67 | birth weight | 166.6 | 2.18E-03 |  | 0.0009 | 0.0029 | 7.48E-01 |
| node_pheno68 | birth weight | 153.5 | 2.13E-02 |  | 0.0000 | 0.0027 | 9.92E-01 |
| node_pheno69 | birth weight | 192.3 | 3.11E-05 |  | 0.0003 | 0.0031 | 9.14E-01 |
| node_pheno7 | birth weight | 160.3 | 8.22E-03 |  | 0.0013 | 0.0030 | 6.62E-01 |
| node_pheno70 | birth weight | 162.1 | 6.34E-03 |  | 0.0008 | 0.0028 | 7.77E-01 |
| node_pheno71 | birth weight | 149.4 | 3.58E-02 |  | -0.0003 | 0.0026 | 9.00E-01 |
| node_pheno72 | birth weight | 185.9 | 1.07E-04 |  | 0.0037 | 0.0027 | 1.82E-01 |
| node_pheno73 | birth weight | 153.0 | 2.25E-02 |  | -0.0002 | 0.0025 | 9.49E-01 |
| node_pheno74 | birth weight | 153.6 | 2.09E-02 |  | 0.0050 | 0.0028 | 7.92E-02 |
| node_pheno75 | birth weight | 150.2 | 3.24E-02 |  | 0.0027 | 0.0025 | 2.95E-01 |
| node_pheno76 | birth weight | 148.5 | 3.97E-02 |  | -0.0002 | 0.0024 | 9.48E-01 |
| node_pheno8 | birth weight | 104.2 | 8.48E-01 |  | -0.0011 | 0.0025 | 6.47E-01 |
| node_pheno9 | birth weight | 129.1 | 2.69E-01 |  | 0.0040 | 0.0027 | 1.38E-01 |
| edge_pheno101 | childhood_BMI | 53.3 | 1.42E-02 |  | -0.0020 | 0.0057 | 7.25E-01 |
| edge_pheno1013 | childhood_BMI | 42.5 | 1.25E-01 |  | -0.0037 | 0.0051 | 4.75E-01 |
| edge_pheno102 | childhood_BMI | 34.1 | 4.16E-01 |  | -0.0028 | 0.0045 | 5.42E-01 |
| edge_pheno1020 | childhood_BMI | 30.8 | 5.79E-01 |  | 0.0054 | 0.0045 | 2.40E-01 |
| edge_pheno1022 | childhood_BMI | 38.8 | 2.26E-01 |  | 0.0035 | 0.0049 | 4.81E-01 |
| edge_pheno103 | childhood_BMI | 29.7 | 6.30E-01 |  | 0.0024 | 0.0044 | 5.96E-01 |
| edge_pheno1041 | childhood_BMI | 28.5 | 6.91E-01 |  | -0.0001 | 0.0045 | 9.80E-01 |
| edge_pheno1059 | childhood_BMI | 26.5 | 7.82E-01 |  | -0.0080 | 0.0045 | 8.31E-02 |
| edge_pheno1122 | childhood_BMI | 54.4 | 1.10E-02 |  | -0.0014 | 0.0057 | 8.09E-01 |
| edge_pheno1126 | childhood_BMI | 35.0 | 3.75E-01 |  | 0.0042 | 0.0046 | 3.66E-01 |
| edge_pheno1134 | childhood_BMI | 35.6 | 3.46E-01 |  | 0.0013 | 0.0045 | 7.79E-01 |
| edge_pheno1137 | childhood_BMI | 30.3 | 6.05E-01 |  | 0.0030 | 0.0045 | 5.11E-01 |
| edge_pheno1141 | childhood_BMI | 30.7 | 5.80E-01 |  | -0.0003 | 0.0044 | 9.53E-01 |
| edge_pheno1142 | childhood_BMI | 30.2 | 6.05E-01 |  | -0.0028 | 0.0045 | 5.34E-01 |
| edge_pheno1161 | childhood_BMI | 51.0 | 2.37E-02 |  | -0.0044 | 0.0054 | 4.20E-01 |
| edge_pheno1167 | childhood_BMI | 41.1 | 1.57E-01 |  | 0.0086 | 0.0050 | 9.27E-02 |
| edge_pheno1171 | childhood_BMI | 22.0 | 9.27E-01 |  | -0.0033 | 0.0044 | 4.62E-01 |
| edge_pheno1175 | childhood_BMI | 38.1 | 2.50E-01 |  | 0.0000 | 0.0048 | 9.99E-01 |
| edge_pheno1183 | childhood_BMI | 34.2 | 4.10E-01 |  | 0.0074 | 0.0046 | 1.13E-01 |
| edge_pheno1184 | childhood_BMI | 49.0 | 3.64E-02 |  | 0.0013 | 0.0055 | 8.09E-01 |
| edge_pheno1189 | childhood_BMI | 49.2 | 3.47E-02 |  | 0.0023 | 0.0054 | 6.74E-01 |
| edge_pheno1205 | childhood_BMI | 28.3 | 7.00E-01 |  | -0.0030 | 0.0044 | 5.05E-01 |
| edge_pheno1211 | childhood_BMI | 36.1 | 3.27E-01 |  | -0.0004 | 0.0047 | 9.31E-01 |
| edge_pheno1221 | childhood_BMI | 30.7 | 5.81E-01 |  | -0.0050 | 0.0044 | 2.70E-01 |
| edge_pheno1225 | childhood_BMI | 44.9 | 8.17E-02 |  | 0.0079 | 0.0052 | 1.36E-01 |
| edge_pheno1250 | childhood_BMI | 43.9 | 9.81E-02 |  | 0.0055 | 0.0050 | 2.83E-01 |
| edge_pheno1256 | childhood_BMI | 43.5 | 1.05E-01 |  | 0.0015 | 0.0050 | 7.61E-01 |
| edge_pheno1257 | childhood_BMI | 54.1 | 1.17E-02 |  | 0.0057 | 0.0056 | 3.17E-01 |
| edge_pheno1269 | childhood_BMI | 38.8 | 2.24E-01 |  | 0.0065 | 0.0048 | 1.83E-01 |
| edge_pheno1270 | childhood_BMI | 48.2 | 4.28E-02 |  | 0.0054 | 0.0053 | 3.14E-01 |
| edge_pheno1273 | childhood_BMI | 41.6 | 1.44E-01 |  | -0.0053 | 0.0050 | 2.99E-01 |
| edge_pheno1276 | childhood_BMI | 26.0 | 8.01E-01 |  | 0.0003 | 0.0043 | 9.39E-01 |
| edge_pheno1293 | childhood_BMI | 39.1 | 2.16E-01 |  | 0.0090 | 0.0049 | 7.54E-02 |
| edge_pheno1296 | childhood_BMI | 41.1 | 1.56E-01 |  | 0.0003 | 0.0050 | 9.46E-01 |
| edge_pheno1300 | childhood_BMI | 35.0 | 3.72E-01 |  | -0.0018 | 0.0046 | 7.01E-01 |
| edge_pheno1301 | childhood_BMI | 48.9 | 3.71E-02 |  | -0.0005 | 0.0055 | 9.25E-01 |
| edge_pheno1302 | childhood_BMI | 25.3 | 8.29E-01 |  | -0.0061 | 0.0044 | 1.80E-01 |
| edge_pheno1309 | childhood_BMI | 29.6 | 6.37E-01 |  | 0.0049 | 0.0045 | 2.86E-01 |
| edge_pheno1311 | childhood_BMI | 17.0 | 8.07E-01 |  | -0.0031 | 0.0053 | 5.58E-01 |
| edge_pheno1317 | childhood_BMI | 28.7 | 1.89E-01 |  | 0.0032 | 0.0058 | 5.91E-01 |
| edge_pheno1319 | childhood_BMI | 46.5 | 5.96E-02 |  | -0.0131 | 0.0053 | 1.88E-02 |
| edge_pheno132 | childhood_BMI | 31.0 | 5.66E-01 |  | 0.0011 | 0.0045 | 8.14E-01 |
| edge_pheno1322 | childhood_BMI | 28.9 | 6.72E-01 |  | -0.0075 | 0.0045 | 1.02E-01 |
| edge_pheno1325 | childhood_BMI | 24.3 | 8.63E-01 |  | -0.0001 | 0.0045 | 9.81E-01 |
| edge_pheno1328 | childhood_BMI | 32.7 | 4.84E-01 |  | 0.0016 | 0.0045 | 7.15E-01 |
| edge_pheno135 | childhood_BMI | 26.9 | 7.62E-01 |  | -0.0050 | 0.0045 | 2.68E-01 |
| edge_pheno1359 | childhood_BMI | 32.9 | 4.73E-01 |  | -0.0020 | 0.0044 | 6.54E-01 |
| edge_pheno1382 | childhood_BMI | 25.6 | 8.18E-01 |  | -0.0022 | 0.0044 | 6.17E-01 |
| edge_pheno146 | childhood_BMI | 46.1 | 6.47E-02 |  | 0.0031 | 0.0053 | 5.55E-01 |
| edge_pheno147 | childhood_BMI | 24.6 | 8.54E-01 |  | 0.0014 | 0.0045 | 7.57E-01 |
| edge_pheno151 | childhood_BMI | 34.3 | 4.07E-01 |  | 0.0005 | 0.0045 | 9.18E-01 |
| edge_pheno1696 | childhood_BMI | 35.2 | 3.64E-01 |  | -0.0020 | 0.0046 | 6.66E-01 |
| edge_pheno1697 | childhood_BMI | 28.0 | 6.67E-01 |  | -0.0010 | 0.0044 | 8.14E-01 |
| edge_pheno1698 | childhood_BMI | 28.3 | 7.00E-01 |  | 0.0105 | 0.0045 | 2.49E-02 |
| edge_pheno1699 | childhood_BMI | 47.8 | 4.62E-02 |  | 0.0001 | 0.0049 | 9.89E-01 |
| edge_pheno1701 | childhood_BMI | 55.9 | 7.73E-03 |  | 0.0024 | 0.0057 | 6.76E-01 |
| edge_pheno249 | childhood_BMI | 37.2 | 2.83E-01 |  | -0.0044 | 0.0047 | 3.57E-01 |
| edge_pheno253 | childhood_BMI | 28.8 | 6.77E-01 |  | -0.0066 | 0.0045 | 1.53E-01 |
| edge_pheno262 | childhood_BMI | 39.8 | 1.93E-01 |  | 0.0071 | 0.0049 | 1.56E-01 |
| edge_pheno286 | childhood_BMI | 48.8 | 3.74E-02 |  | 0.0029 | 0.0054 | 5.91E-01 |
| edge_pheno288 | childhood_BMI | 24.0 | 8.74E-01 |  | 0.0140 | 0.0045 | 3.85E-03 |
| edge_pheno303 | childhood_BMI | 38.3 | 2.41E-01 |  | 0.0000 | 0.0048 | 9.98E-01 |
| edge_pheno389 | childhood_BMI | 30.9 | 5.74E-01 |  | 0.0028 | 0.0045 | 5.35E-01 |
| edge_pheno405 | childhood_BMI | 40.9 | 1.64E-01 |  | -0.0111 | 0.0049 | 3.03E-02 |
| edge_pheno447 | childhood_BMI | 2.0 | 5.68E-01 |  | -0.0207 | 0.0137 | 2.28E-01 |
| edge_pheno449 | childhood_BMI | 4.1 | 2.46E-01 |  | 0.0071 | 0.0160 | 6.88E-01 |
| edge_pheno460 | childhood_BMI | 35.1 | 3.68E-01 |  | -0.0002 | 0.0046 | 9.71E-01 |
| edge_pheno461 | childhood_BMI | 33.4 | 4.46E-01 |  | 0.0022 | 0.0045 | 6.24E-01 |
| edge_pheno491 | childhood_BMI | 27.1 | 7.57E-01 |  | 0.0096 | 0.0045 | 3.91E-02 |
| edge_pheno537 | childhood_BMI | 27.2 | 7.51E-01 |  | -0.0018 | 0.0044 | 6.78E-01 |
| edge_pheno55 | childhood_BMI | 39.8 | 1.92E-01 |  | -0.0025 | 0.0049 | 6.16E-01 |
| edge_pheno558 | childhood_BMI | 40.7 | 1.68E-01 |  | 0.0075 | 0.0049 | 1.38E-01 |
| edge_pheno574 | childhood_BMI | 33.0 | 4.68E-01 |  | 0.0050 | 0.0044 | 2.67E-01 |
| edge_pheno58 | childhood_BMI | 35.8 | 3.37E-01 |  | -0.0005 | 0.0045 | 9.17E-01 |
| edge_pheno590 | childhood_BMI | 35.5 | 3.49E-01 |  | -0.0057 | 0.0046 | 2.29E-01 |
| edge_pheno593 | childhood_BMI | 31.7 | 5.34E-01 |  | 0.0052 | 0.0045 | 2.48E-01 |
| edge_pheno597 | childhood_BMI | 29.2 | 6.56E-01 |  | 0.0062 | 0.0044 | 1.67E-01 |
| edge_pheno599 | childhood_BMI | 28.4 | 6.94E-01 |  | 0.0042 | 0.0045 | 3.51E-01 |
| edge_pheno601 | childhood_BMI | 45.0 | 7.93E-02 |  | -0.0017 | 0.0052 | 7.51E-01 |
| edge_pheno606 | childhood_BMI | 37.2 | 2.82E-01 |  | -0.0058 | 0.0047 | 2.27E-01 |
| edge_pheno609 | childhood_BMI | 37.0 | 2.91E-01 |  | 0.0017 | 0.0047 | 7.17E-01 |
| edge_pheno621 | childhood_BMI | 34.8 | 3.82E-01 |  | 0.0059 | 0.0046 | 2.10E-01 |
| edge_pheno624 | childhood_BMI | 30.0 | 6.16E-01 |  | 0.0025 | 0.0045 | 5.86E-01 |
| edge_pheno636 | childhood_BMI | 44.4 | 8.85E-02 |  | 0.0020 | 0.0052 | 7.03E-01 |
| edge_pheno639 | childhood_BMI | 23.4 | 8.91E-01 |  | 0.0052 | 0.0044 | 2.45E-01 |
| edge_pheno65 | childhood_BMI | 27.9 | 7.19E-01 |  | -0.0003 | 0.0045 | 9.49E-01 |
| edge_pheno66 | childhood_BMI | 37.9 | 2.57E-01 |  | -0.0029 | 0.0047 | 5.41E-01 |
| edge_pheno674 | childhood_BMI | 35.1 | 3.68E-01 |  | 0.0018 | 0.0046 | 7.00E-01 |
| edge_pheno681 | childhood_BMI | 28.7 | 6.82E-01 |  | 0.0000 | 0.0045 | 9.92E-01 |
| edge_pheno683 | childhood_BMI | 20.1 | 9.62E-01 |  | 0.0011 | 0.0045 | 8.06E-01 |
| edge_pheno695 | childhood_BMI | 24.3 | 8.65E-01 |  | 0.0049 | 0.0045 | 2.85E-01 |
| edge_pheno698 | childhood_BMI | 37.4 | 2.75E-01 |  | 0.0024 | 0.0047 | 6.16E-01 |
| edge_pheno705 | childhood_BMI | 34.6 | 3.91E-01 |  | -0.0072 | 0.0046 | 1.25E-01 |
| edge_pheno716 | childhood_BMI | 30.3 | 6.02E-01 |  | -0.0049 | 0.0045 | 2.85E-01 |
| edge_pheno767 | childhood_BMI | 42.4 | 1.26E-01 |  | -0.0032 | 0.0051 | 5.33E-01 |
| edge_pheno777 | childhood_BMI | 43.4 | 1.06E-01 |  | 0.0012 | 0.0051 | 8.22E-01 |
| edge_pheno789 | childhood_BMI | 30.7 | 5.84E-01 |  | 0.0015 | 0.0044 | 7.31E-01 |
| edge_pheno794 | childhood_BMI | 33.0 | 4.66E-01 |  | 0.0034 | 0.0044 | 4.45E-01 |
| edge_pheno801 | childhood_BMI | 45.5 | 7.28E-02 |  | -0.0087 | 0.0052 | 1.05E-01 |
| edge_pheno810 | childhood_BMI | 24.2 | 8.66E-01 |  | 0.0053 | 0.0043 | 2.31E-01 |
| edge_pheno812 | childhood_BMI | 26.2 | 7.92E-01 |  | 0.0076 | 0.0045 | 9.90E-02 |
| edge_pheno815 | childhood_BMI | 33.0 | 4.66E-01 |  | -0.0025 | 0.0044 | 5.79E-01 |
| edge_pheno816 | childhood_BMI | 26.6 | 7.77E-01 |  | -0.0054 | 0.0043 | 2.22E-01 |
| edge_pheno824 | childhood_BMI | 50.5 | 2.60E-02 |  | 0.0043 | 0.0054 | 4.28E-01 |
| edge_pheno867 | childhood_BMI | 38.0 | 2.52E-01 |  | -0.0007 | 0.0048 | 8.91E-01 |
| edge_pheno869 | childhood_BMI | 56.3 | 6.94E-03 |  | 0.0015 | 0.0058 | 8.03E-01 |
| edge_pheno87 | childhood_BMI | 46.5 | 5.98E-02 |  | -0.0088 | 0.0053 | 1.07E-01 |
| edge_pheno882 | childhood_BMI | 42.7 | 1.20E-01 |  | 0.0056 | 0.0051 | 2.85E-01 |
| edge_pheno899 | childhood_BMI | 31.6 | 5.37E-01 |  | 0.0006 | 0.0045 | 8.94E-01 |
| edge_pheno903 | childhood_BMI | 31.7 | 5.34E-01 |  | -0.0031 | 0.0044 | 4.92E-01 |
| edge_pheno908 | childhood_BMI | 29.8 | 6.25E-01 |  | 0.0033 | 0.0045 | 4.71E-01 |
| edge_pheno918 | childhood_BMI | 37.0 | 2.89E-01 |  | 0.0001 | 0.0046 | 9.82E-01 |
| edge_pheno932 | childhood_BMI | 27.4 | 7.40E-01 |  | -0.0021 | 0.0044 | 6.30E-01 |
| edge_pheno942 | childhood_BMI | 26.5 | 7.81E-01 |  | -0.0086 | 0.0044 | 6.02E-02 |
| edge_pheno956 | childhood_BMI | 48.0 | 4.44E-02 |  | -0.0029 | 0.0054 | 5.95E-01 |
| edge_pheno965 | childhood_BMI | 35.9 | 3.35E-01 |  | -0.0041 | 0.0047 | 3.85E-01 |
| node_pheno1 | childhood_BMI | 41.9 | 1.38E-01 |  | -0.0110 | 0.0049 | 3.12E-02 |
| node_pheno10 | childhood_BMI | 40.8 | 1.66E-01 |  | -0.0040 | 0.0049 | 4.21E-01 |
| node_pheno11 | childhood_BMI | 44.8 | 8.19E-02 |  | -0.0012 | 0.0051 | 8.09E-01 |
| node_pheno12 | childhood_BMI | 49.5 | 3.28E-02 |  | -0.0067 | 0.0054 | 2.24E-01 |
| node_pheno13 | childhood_BMI | 30.3 | 6.03E-01 |  | -0.0015 | 0.0044 | 7.28E-01 |
| node_pheno14 | childhood_BMI | 43.4 | 1.06E-01 |  | -0.0066 | 0.0049 | 1.85E-01 |
| node_pheno15 | childhood_BMI | 44.7 | 8.48E-02 |  | -0.0018 | 0.0051 | 7.19E-01 |
| node_pheno16 | childhood_BMI | 43.3 | 1.09E-01 |  | -0.0024 | 0.0048 | 6.25E-01 |
| node_pheno17 | childhood_BMI | 52.1 | 1.84E-02 |  | -0.0027 | 0.0054 | 6.24E-01 |
| node_pheno18 | childhood_BMI | 47.7 | 4.73E-02 |  | -0.0055 | 0.0051 | 2.89E-01 |
| node_pheno19 | childhood_BMI | 50.6 | 2.55E-02 |  | -0.0030 | 0.0053 | 5.78E-01 |
| node_pheno2 | childhood_BMI | 46.8 | 5.61E-02 |  | -0.0068 | 0.0052 | 2.01E-01 |
| node_pheno20 | childhood_BMI | 65.5 | 6.35E-04 |  | -0.0073 | 0.0061 | 2.35E-01 |
| node_pheno21 | childhood_BMI | 39.9 | 1.90E-01 |  | -0.0069 | 0.0048 | 1.61E-01 |
| node_pheno22 | childhood_BMI | 44.5 | 8.67E-02 |  | -0.0026 | 0.0049 | 6.02E-01 |
| node_pheno23 | childhood_BMI | 56.9 | 6.02E-03 |  | -0.0008 | 0.0054 | 8.78E-01 |
| node_pheno24 | childhood_BMI | 48.2 | 4.23E-02 |  | -0.0014 | 0.0051 | 7.87E-01 |
| node_pheno25 | childhood_BMI | 45.8 | 6.81E-02 |  | -0.0056 | 0.0050 | 2.67E-01 |
| node_pheno26 | childhood_BMI | 33.4 | 4.49E-01 |  | -0.0065 | 0.0041 | 1.20E-01 |
| node_pheno27 | childhood_BMI | 47.2 | 5.16E-02 |  | 0.0022 | 0.0052 | 6.83E-01 |
| node_pheno28 | childhood_BMI | 45.0 | 7.90E-02 |  | -0.0030 | 0.0048 | 5.44E-01 |
| node_pheno29 | childhood_BMI | 41.2 | 1.54E-01 |  | 0.0034 | 0.0047 | 4.77E-01 |
| node_pheno3 | childhood_BMI | 41.9 | 1.39E-01 |  | -0.0025 | 0.0049 | 6.21E-01 |
| node_pheno30 | childhood_BMI | 47.8 | 4.64E-02 |  | -0.0028 | 0.0051 | 5.85E-01 |
| node_pheno31 | childhood_BMI | 40.9 | 1.63E-01 |  | -0.0021 | 0.0047 | 6.56E-01 |
| node_pheno32 | childhood_BMI | 46.2 | 6.28E-02 |  | 0.0033 | 0.0050 | 5.10E-01 |
| node_pheno33 | childhood_BMI | 28.1 | 7.08E-01 |  | -0.0077 | 0.0043 | 8.13E-02 |
| node_pheno34 | childhood_BMI | 34.2 | 4.09E-01 |  | -0.0009 | 0.0042 | 8.39E-01 |
| node_pheno35 | childhood_BMI | 53.2 | 1.43E-02 |  | -0.0033 | 0.0053 | 5.34E-01 |
| node_pheno36 | childhood_BMI | 53.8 | 1.24E-02 |  | -0.0050 | 0.0052 | 3.42E-01 |
| node_pheno37 | childhood_BMI | 44.3 | 9.05E-02 |  | -0.0010 | 0.0050 | 8.42E-01 |
| node_pheno38 | childhood_BMI | 44.2 | 9.19E-02 |  | 0.0001 | 0.0047 | 9.91E-01 |
| node_pheno39 | childhood_BMI | 42.2 | 1.30E-01 |  | 0.0007 | 0.0047 | 8.85E-01 |
| node_pheno4 | childhood_BMI | 44.8 | 8.30E-02 |  | -0.0084 | 0.0050 | 1.05E-01 |
| node_pheno40 | childhood_BMI | 64.6 | 8.10E-04 |  | -0.0053 | 0.0058 | 3.65E-01 |
| node_pheno41 | childhood_BMI | 44.7 | 8.36E-02 |  | -0.0046 | 0.0051 | 3.75E-01 |
| node_pheno42 | childhood_BMI | 49.4 | 3.33E-02 |  | -0.0049 | 0.0050 | 3.36E-01 |
| node_pheno43 | childhood_BMI | 55.7 | 8.11E-03 |  | -0.0043 | 0.0055 | 4.43E-01 |
| node_pheno44 | childhood_BMI | 27.5 | 7.38E-01 |  | -0.0021 | 0.0040 | 6.12E-01 |
| node_pheno45 | childhood_BMI | 54.5 | 1.07E-02 |  | -0.0007 | 0.0054 | 8.94E-01 |
| node_pheno46 | childhood_BMI | 52.3 | 1.75E-02 |  | -0.0029 | 0.0054 | 5.98E-01 |
| node_pheno47 | childhood_BMI | 48.4 | 4.08E-02 |  | -0.0048 | 0.0052 | 3.60E-01 |
| node_pheno48 | childhood_BMI | 53.5 | 1.34E-02 |  | -0.0030 | 0.0055 | 5.91E-01 |
| node_pheno49 | childhood_BMI | 34.9 | 3.78E-01 |  | -0.0003 | 0.0044 | 9.48E-01 |
| node_pheno5 | childhood_BMI | 45.9 | 6.67E-02 |  | -0.0119 | 0.0052 | 2.74E-02 |
| node_pheno50 | childhood_BMI | 51.2 | 2.23E-02 |  | 0.0025 | 0.0052 | 6.35E-01 |
| node_pheno51 | childhood_BMI | 43.4 | 1.06E-01 |  | -0.0069 | 0.0050 | 1.75E-01 |
| node_pheno52 | childhood_BMI | 41.4 | 1.50E-01 |  | -0.0030 | 0.0046 | 5.20E-01 |
| node_pheno53 | childhood_BMI | 47.6 | 4.78E-02 |  | -0.0037 | 0.0053 | 4.84E-01 |
| node_pheno54 | childhood_BMI | 44.7 | 8.44E-02 |  | -0.0033 | 0.0050 | 5.07E-01 |
| node_pheno55 | childhood_BMI | 48.9 | 3.70E-02 |  | -0.0045 | 0.0052 | 3.93E-01 |
| node_pheno56 | childhood_BMI | 61.1 | 2.09E-03 |  | -0.0055 | 0.0058 | 3.50E-01 |
| node_pheno57 | childhood_BMI | 54.2 | 1.14E-02 |  | -0.0025 | 0.0053 | 6.45E-01 |
| node_pheno58 | childhood_BMI | 45.7 | 7.02E-02 |  | -0.0015 | 0.0050 | 7.61E-01 |
| node_pheno59 | childhood_BMI | 43.4 | 1.07E-01 |  | -0.0049 | 0.0042 | 2.59E-01 |
| node_pheno60 | childhood_BMI | 56.3 | 6.94E-03 |  | 0.0000 | 0.0055 | 9.99E-01 |
| node_pheno61 | childhood_BMI | 49.6 | 3.17E-02 |  | -0.0034 | 0.0052 | 5.17E-01 |
| node_pheno62 | childhood_BMI | 73.6 | 6.30E-05 |  | -0.0070 | 0.0064 | 2.86E-01 |
| node_pheno63 | childhood_BMI | 45.2 | 7.66E-02 |  | 0.0035 | 0.0049 | 4.82E-01 |
| node_pheno64 | childhood_BMI | 27.0 | 7.61E-01 |  | 0.0015 | 0.0040 | 7.06E-01 |
| node_pheno65 | childhood_BMI | 47.7 | 4.74E-02 |  | -0.0033 | 0.0050 | 5.10E-01 |
| node_pheno66 | childhood_BMI | 52.5 | 1.67E-02 |  | 0.0037 | 0.0053 | 4.82E-01 |
| node_pheno67 | childhood_BMI | 61.2 | 2.01E-03 |  | -0.0098 | 0.0057 | 9.53E-02 |
| node_pheno68 | childhood_BMI | 49.1 | 3.56E-02 |  | -0.0002 | 0.0049 | 9.62E-01 |
| node_pheno69 | childhood_BMI | 68.0 | 3.21E-04 |  | -0.0002 | 0.0061 | 9.69E-01 |
| node_pheno7 | childhood_BMI | 46.9 | 5.47E-02 |  | -0.0012 | 0.0052 | 8.27E-01 |
| node_pheno70 | childhood_BMI | 53.6 | 1.31E-02 |  | -0.0013 | 0.0052 | 7.98E-01 |
| node_pheno71 | childhood_BMI | 43.0 | 1.15E-01 |  | -0.0078 | 0.0046 | 9.89E-02 |
| node_pheno72 | childhood_BMI | 44.3 | 9.12E-02 |  | 0.0001 | 0.0043 | 9.77E-01 |
| node_pheno73 | childhood_BMI | 41.9 | 1.38E-01 |  | -0.0029 | 0.0042 | 4.98E-01 |
| node_pheno74 | childhood_BMI | 42.4 | 1.26E-01 |  | -0.0004 | 0.0049 | 9.30E-01 |
| node_pheno75 | childhood_BMI | 55.5 | 8.33E-03 |  | -0.0017 | 0.0051 | 7.34E-01 |
| node_pheno76 | childhood_BMI | 52.5 | 1.70E-02 |  | 0.0044 | 0.0046 | 3.49E-01 |
| node_pheno8 | childhood_BMI | 58.5 | 4.09E-03 |  | -0.0072 | 0.0057 | 2.15E-01 |
| node_pheno9 | childhood_BMI | 31.1 | 5.62E-01 |  | -0.0107 | 0.0044 | 2.09E-02 |
| edge_pheno101 | adulthood_BMI | 672.3 | 3.36E-02 |  | 0.0002 | 0.0010 | 8.23E-01 |
| edge_pheno1013 | adulthood_BMI | 646.9 | 1.27E-01 |  | 0.0003 | 0.0010 | 7.55E-01 |
| edge_pheno102 | adulthood_BMI | 709.2 | 2.53E-03 |  | -0.0005 | 0.0010 | 6.60E-01 |
| edge_pheno1020 | adulthood_BMI | 582.2 | 7.59E-01 |  | 0.0003 | 0.0010 | 7.33E-01 |
| edge_pheno1022 | adulthood_BMI | 612.3 | 4.33E-01 |  | 0.0011 | 0.0010 | 2.63E-01 |
| edge_pheno103 | adulthood_BMI | 681.3 | 1.92E-02 |  | 0.0005 | 0.0010 | 5.87E-01 |
| edge_pheno1041 | adulthood_BMI | 674.2 | 3.00E-02 |  | 0.0007 | 0.0010 | 4.96E-01 |
| edge_pheno1059 | adulthood_BMI | 708.2 | 2.75E-03 |  | -0.0018 | 0.0010 | 7.24E-02 |
| edge_pheno1122 | adulthood_BMI | 913.4 | 9.52E-15 |  | 0.0018 | 0.0012 | 1.18E-01 |
| edge_pheno1126 | adulthood_BMI | 678.9 | 2.25E-02 |  | 0.0011 | 0.0010 | 2.60E-01 |
| edge_pheno1134 | adulthood_BMI | 808.6 | 7.02E-08 |  | 0.0009 | 0.0011 | 3.81E-01 |
| edge_pheno1137 | adulthood_BMI | 809.9 | 5.92E-08 |  | 0.0004 | 0.0011 | 7.33E-01 |
| edge_pheno1141 | adulthood_BMI | 777.6 | 3.09E-06 |  | 0.0009 | 0.0011 | 3.76E-01 |
| edge_pheno1142 | adulthood_BMI | 714.3 | 1.67E-03 |  | 0.0010 | 0.0010 | 3.33E-01 |
| edge_pheno1161 | adulthood_BMI | 808.3 | 7.32E-08 |  | 0.0000 | 0.0011 | 9.66E-01 |
| edge_pheno1167 | adulthood_BMI | 776.8 | 3.40E-06 |  | 0.0002 | 0.0011 | 8.29E-01 |
| edge_pheno1171 | adulthood_BMI | 779.8 | 2.39E-06 |  | -0.0004 | 0.0011 | 6.90E-01 |
| edge_pheno1175 | adulthood_BMI | 622.2 | 3.26E-01 |  | -0.0006 | 0.0010 | 5.32E-01 |
| edge_pheno1183 | adulthood_BMI | 723.4 | 7.69E-04 |  | -0.0001 | 0.0010 | 9.17E-01 |
| edge_pheno1184 | adulthood_BMI | 703.2 | 4.07E-03 |  | -0.0008 | 0.0010 | 4.41E-01 |
| edge_pheno1189 | adulthood_BMI | 740.4 | 1.60E-04 |  | 0.0009 | 0.0010 | 4.12E-01 |
| edge_pheno1205 | adulthood_BMI | 588.1 | 7.02E-01 |  | -0.0003 | 0.0009 | 7.62E-01 |
| edge_pheno1211 | adulthood_BMI | 679.0 | 2.22E-02 |  | -0.0005 | 0.0010 | 6.32E-01 |
| edge_pheno1221 | adulthood_BMI | 802.0 | 1.64E-07 |  | 0.0011 | 0.0011 | 3.05E-01 |
| edge_pheno1225 | adulthood_BMI | 713.5 | 1.79E-03 |  | 0.0011 | 0.0010 | 3.01E-01 |
| edge_pheno1250 | adulthood_BMI | 754.2 | 4.02E-05 |  | 0.0001 | 0.0010 | 9.16E-01 |
| edge_pheno1256 | adulthood_BMI | 720.9 | 9.56E-04 |  | -0.0016 | 0.0010 | 1.21E-01 |
| edge_pheno1257 | adulthood_BMI | 677.0 | 2.53E-02 |  | 0.0009 | 0.0010 | 3.62E-01 |
| edge_pheno1269 | adulthood_BMI | 737.5 | 2.11E-04 |  | 0.0014 | 0.0010 | 1.94E-01 |
| edge_pheno1270 | adulthood_BMI | 773.5 | 4.93E-06 |  | -0.0005 | 0.0011 | 6.26E-01 |
| edge_pheno1273 | adulthood_BMI | 700.6 | 4.97E-03 |  | -0.0002 | 0.0010 | 8.81E-01 |
| edge_pheno1276 | adulthood_BMI | 703.1 | 4.09E-03 |  | -0.0005 | 0.0010 | 5.93E-01 |
| edge_pheno1293 | adulthood_BMI | 724.4 | 7.05E-04 |  | 0.0022 | 0.0010 | 3.63E-02 |
| edge_pheno1296 | adulthood_BMI | 704.0 | 3.81E-03 |  | -0.0002 | 0.0010 | 8.56E-01 |
| edge_pheno1300 | adulthood_BMI | 691.1 | 9.91E-03 |  | -0.0023 | 0.0010 | 2.53E-02 |
| edge_pheno1301 | adulthood_BMI | 734.9 | 2.70E-04 |  | 0.0011 | 0.0011 | 2.95E-01 |
| edge_pheno1302 | adulthood_BMI | 760.0 | 2.18E-05 |  | -0.0015 | 0.0011 | 1.47E-01 |
| edge_pheno1309 | adulthood_BMI | 765.8 | 1.17E-05 |  | 0.0025 | 0.0011 | 2.17E-02 |
| edge_pheno1311 | adulthood_BMI | 436.1 | 3.83E-01 |  | -0.0012 | 0.0012 | 3.11E-01 |
| edge_pheno1317 | adulthood_BMI | 507.5 | 2.95E-03 |  | 0.0008 | 0.0013 | 5.23E-01 |
| edge_pheno1319 | adulthood_BMI | 734.0 | 2.94E-04 |  | -0.0023 | 0.0010 | 2.87E-02 |
| edge_pheno132 | adulthood_BMI | 659.5 | 6.88E-02 |  | 0.0008 | 0.0010 | 4.03E-01 |
| edge_pheno1322 | adulthood_BMI | 713.4 | 1.80E-03 |  | -0.0006 | 0.0010 | 5.60E-01 |
| edge_pheno1325 | adulthood_BMI | 675.8 | 2.73E-02 |  | 0.0008 | 0.0010 | 4.33E-01 |
| edge_pheno1328 | adulthood_BMI | 731.0 | 3.87E-04 |  | 0.0022 | 0.0010 | 3.59E-02 |
| edge_pheno135 | adulthood_BMI | 634.3 | 2.15E-01 |  | 0.0002 | 0.0010 | 8.18E-01 |
| edge_pheno1359 | adulthood_BMI | 740.3 | 1.61E-04 |  | -0.0009 | 0.0010 | 3.84E-01 |
| edge_pheno1382 | adulthood_BMI | 826.4 | 6.51E-09 |  | 0.0003 | 0.0011 | 7.63E-01 |
| edge_pheno146 | adulthood_BMI | 621.1 | 3.37E-01 |  | 0.0014 | 0.0010 | 1.53E-01 |
| edge_pheno147 | adulthood_BMI | 675.9 | 2.71E-02 |  | 0.0015 | 0.0010 | 1.37E-01 |
| edge_pheno151 | adulthood_BMI | 651.8 | 1.01E-01 |  | -0.0007 | 0.0010 | 4.78E-01 |
| edge_pheno1696 | adulthood_BMI | 706.2 | 3.21E-03 |  | -0.0001 | 0.0010 | 9.19E-01 |
| edge_pheno1697 | adulthood_BMI | 832.2 | 2.94E-09 |  | -0.0009 | 0.0011 | 4.14E-01 |
| edge_pheno1698 | adulthood_BMI | 950.9 | 1.23E-17 |  | 0.0023 | 0.0012 | 5.21E-02 |
| edge_pheno1699 | adulthood_BMI | 880.5 | 2.15E-12 |  | 0.0002 | 0.0010 | 8.59E-01 |
| edge_pheno1701 | adulthood_BMI | 1006.4 | 2.82E-22 |  | -0.0005 | 0.0012 | 7.06E-01 |
| edge_pheno249 | adulthood_BMI | 683.8 | 1.63E-02 |  | -0.0008 | 0.0010 | 4.03E-01 |
| edge_pheno253 | adulthood_BMI | 679.6 | 2.14E-02 |  | -0.0025 | 0.0010 | 1.23E-02 |
| edge_pheno262 | adulthood_BMI | 662.1 | 6.00E-02 |  | 0.0010 | 0.0010 | 3.33E-01 |
| edge_pheno286 | adulthood_BMI | 651.0 | 1.05E-01 |  | 0.0011 | 0.0010 | 2.37E-01 |
| edge_pheno288 | adulthood_BMI | 605.5 | 5.10E-01 |  | -0.0013 | 0.0010 | 1.65E-01 |
| edge_pheno303 | adulthood_BMI | 688.0 | 1.23E-02 |  | 0.0011 | 0.0010 | 2.55E-01 |
| edge_pheno389 | adulthood_BMI | 698.7 | 5.73E-03 |  | 0.0015 | 0.0010 | 1.55E-01 |
| edge_pheno405 | adulthood_BMI | 746.7 | 8.65E-05 |  | -0.0018 | 0.0010 | 7.87E-02 |
| edge_pheno447 | adulthood_BMI | 52.5 | 3.40E-01 |  | -0.0031 | 0.0029 | 2.88E-01 |
| edge_pheno449 | adulthood_BMI | 60.1 | 7.91E-02 |  | 0.0027 | 0.0033 | 4.23E-01 |
| edge_pheno460 | adulthood_BMI | 672.1 | 3.40E-02 |  | -0.0015 | 0.0010 | 1.46E-01 |
| edge_pheno461 | adulthood_BMI | 633.3 | 2.22E-01 |  | 0.0005 | 0.0010 | 5.98E-01 |
| edge_pheno491 | adulthood_BMI | 731.5 | 3.70E-04 |  | -0.0001 | 0.0010 | 8.92E-01 |
| edge_pheno537 | adulthood_BMI | 785.1 | 1.30E-06 |  | 0.0004 | 0.0011 | 6.71E-01 |
| edge_pheno55 | adulthood_BMI | 643.6 | 1.47E-01 |  | 0.0010 | 0.0010 | 3.22E-01 |
| edge_pheno558 | adulthood_BMI | 664.5 | 5.27E-02 |  | 0.0010 | 0.0010 | 3.25E-01 |
| edge_pheno574 | adulthood_BMI | 658.8 | 7.14E-02 |  | 0.0021 | 0.0010 | 2.93E-02 |
| edge_pheno58 | adulthood_BMI | 687.7 | 1.26E-02 |  | -0.0004 | 0.0010 | 6.73E-01 |
| edge_pheno590 | adulthood_BMI | 757.9 | 2.73E-05 |  | -0.0012 | 0.0011 | 2.45E-01 |
| edge_pheno593 | adulthood_BMI | 748.9 | 6.90E-05 |  | 0.0021 | 0.0011 | 4.32E-02 |
| edge_pheno597 | adulthood_BMI | 784.2 | 1.44E-06 |  | 0.0016 | 0.0011 | 1.25E-01 |
| edge_pheno599 | adulthood_BMI | 700.0 | 5.21E-03 |  | 0.0009 | 0.0010 | 3.69E-01 |
| edge_pheno601 | adulthood_BMI | 712.0 | 2.03E-03 |  | 0.0014 | 0.0010 | 1.80E-01 |
| edge_pheno606 | adulthood_BMI | 679.3 | 2.19E-02 |  | -0.0002 | 0.0010 | 8.11E-01 |
| edge_pheno609 | adulthood_BMI | 725.7 | 6.29E-04 |  | 0.0016 | 0.0010 | 1.19E-01 |
| edge_pheno621 | adulthood_BMI | 672.3 | 3.37E-02 |  | -0.0008 | 0.0010 | 4.14E-01 |
| edge_pheno624 | adulthood_BMI | 641.5 | 1.61E-01 |  | -0.0003 | 0.0010 | 7.88E-01 |
| edge_pheno636 | adulthood_BMI | 725.5 | 6.41E-04 |  | 0.0002 | 0.0010 | 8.28E-01 |
| edge_pheno639 | adulthood_BMI | 613.9 | 4.14E-01 |  | 0.0010 | 0.0009 | 3.04E-01 |
| edge_pheno65 | adulthood_BMI | 640.6 | 1.67E-01 |  | -0.0008 | 0.0010 | 4.11E-01 |
| edge_pheno66 | adulthood_BMI | 649.3 | 1.14E-01 |  | 0.0010 | 0.0010 | 3.04E-01 |
| edge_pheno674 | adulthood_BMI | 683.9 | 1.62E-02 |  | 0.0004 | 0.0010 | 6.73E-01 |
| edge_pheno681 | adulthood_BMI | 678.2 | 2.34E-02 |  | 0.0015 | 0.0010 | 1.32E-01 |
| edge_pheno683 | adulthood_BMI | 658.9 | 7.12E-02 |  | -0.0009 | 0.0010 | 3.92E-01 |
| edge_pheno695 | adulthood_BMI | 659.4 | 6.91E-02 |  | 0.0001 | 0.0010 | 9.52E-01 |
| edge_pheno698 | adulthood_BMI | 655.0 | 8.64E-02 |  | 0.0007 | 0.0010 | 4.82E-01 |
| edge_pheno705 | adulthood_BMI | 669.1 | 4.07E-02 |  | -0.0003 | 0.0010 | 7.57E-01 |
| edge_pheno716 | adulthood_BMI | 658.2 | 7.36E-02 |  | 0.0014 | 0.0010 | 1.51E-01 |
| edge_pheno767 | adulthood_BMI | 648.1 | 1.20E-01 |  | -0.0011 | 0.0010 | 2.54E-01 |
| edge_pheno777 | adulthood_BMI | 778.2 | 2.89E-06 |  | 0.0005 | 0.0011 | 6.58E-01 |
| edge_pheno789 | adulthood_BMI | 645.7 | 1.34E-01 |  | -0.0007 | 0.0010 | 4.64E-01 |
| edge_pheno794 | adulthood_BMI | 610.3 | 4.55E-01 |  | -0.0006 | 0.0009 | 5.22E-01 |
| edge_pheno801 | adulthood_BMI | 706.6 | 3.13E-03 |  | -0.0011 | 0.0010 | 2.67E-01 |
| edge_pheno810 | adulthood_BMI | 671.6 | 3.51E-02 |  | 0.0009 | 0.0010 | 3.73E-01 |
| edge_pheno812 | adulthood_BMI | 764.3 | 1.37E-05 |  | -0.0002 | 0.0011 | 8.59E-01 |
| edge_pheno815 | adulthood_BMI | 763.5 | 1.49E-05 |  | 0.0011 | 0.0011 | 3.19E-01 |
| edge_pheno816 | adulthood_BMI | 671.5 | 3.53E-02 |  | 0.0009 | 0.0010 | 3.38E-01 |
| edge_pheno824 | adulthood_BMI | 750.0 | 6.19E-05 |  | 0.0005 | 0.0010 | 6.16E-01 |
| edge_pheno867 | adulthood_BMI | 725.3 | 6.52E-04 |  | -0.0010 | 0.0010 | 3.25E-01 |
| edge_pheno869 | adulthood_BMI | 837.9 | 1.32E-09 |  | -0.0007 | 0.0011 | 5.40E-01 |
| edge_pheno87 | adulthood_BMI | 678.0 | 2.37E-02 |  | 0.0013 | 0.0010 | 2.03E-01 |
| edge_pheno882 | adulthood_BMI | 610.6 | 4.52E-01 |  | 0.0013 | 0.0010 | 1.87E-01 |
| edge_pheno899 | adulthood_BMI | 670.0 | 3.84E-02 |  | 0.0000 | 0.0010 | 9.87E-01 |
| edge_pheno903 | adulthood_BMI | 706.7 | 3.09E-03 |  | -0.0005 | 0.0010 | 5.93E-01 |
| edge_pheno908 | adulthood_BMI | 677.6 | 2.43E-02 |  | 0.0004 | 0.0010 | 6.70E-01 |
| edge_pheno918 | adulthood_BMI | 624.1 | 3.06E-01 |  | 0.0007 | 0.0009 | 4.68E-01 |
| edge_pheno932 | adulthood_BMI | 777.8 | 3.03E-06 |  | -0.0009 | 0.0011 | 4.16E-01 |
| edge_pheno942 | adulthood_BMI | 808.6 | 6.99E-08 |  | -0.0008 | 0.0011 | 4.60E-01 |
| edge_pheno956 | adulthood_BMI | 771.4 | 6.25E-06 |  | -0.0014 | 0.0011 | 1.95E-01 |
| edge_pheno965 | adulthood_BMI | 622.0 | 3.28E-01 |  | -0.0008 | 0.0010 | 3.82E-01 |
| node_pheno1 | adulthood_BMI | 786.6 | 9.40E-07 |  | -0.0014 | 0.0011 | 1.79E-01 |
| node_pheno10 | adulthood_BMI | 736.9 | 2.25E-04 |  | -0.0004 | 0.0010 | 7.07E-01 |
| node_pheno11 | adulthood_BMI | 739.1 | 1.81E-04 |  | 0.0003 | 0.0010 | 7.78E-01 |
| node_pheno12 | adulthood_BMI | 714.0 | 1.71E-03 |  | -0.0005 | 0.0010 | 5.93E-01 |
| node_pheno13 | adulthood_BMI | 785.2 | 1.28E-06 |  | -0.0010 | 0.0011 | 3.30E-01 |
| node_pheno14 | adulthood_BMI | 785.7 | 1.20E-06 |  | -0.0017 | 0.0010 | 9.95E-02 |
| node_pheno15 | adulthood_BMI | 796.1 | 3.42E-07 |  | -0.0012 | 0.0011 | 2.53E-01 |
| node_pheno16 | adulthood_BMI | 733.6 | 2.77E-04 |  | -0.0006 | 0.0010 | 5.18E-01 |
| node_pheno17 | adulthood_BMI | 738.6 | 1.90E-04 |  | -0.0010 | 0.0010 | 3.48E-01 |
| node_pheno18 | adulthood_BMI | 737.0 | 2.22E-04 |  | 0.0000 | 0.0010 | 9.78E-01 |
| node_pheno19 | adulthood_BMI | 723.9 | 6.70E-04 |  | -0.0005 | 0.0010 | 6.20E-01 |
| node_pheno2 | adulthood_BMI | 723.6 | 6.84E-04 |  | 0.0005 | 0.0010 | 6.00E-01 |
| node_pheno20 | adulthood_BMI | 703.0 | 3.79E-03 |  | -0.0003 | 0.0010 | 7.23E-01 |
| node_pheno21 | adulthood_BMI | 733.9 | 2.68E-04 |  | -0.0009 | 0.0010 | 3.87E-01 |
| node_pheno22 | adulthood_BMI | 713.1 | 1.84E-03 |  | 0.0001 | 0.0010 | 9.19E-01 |
| node_pheno23 | adulthood_BMI | 811.5 | 4.78E-08 |  | -0.0007 | 0.0010 | 4.91E-01 |
| node_pheno24 | adulthood_BMI | 742.6 | 1.16E-04 |  | -0.0004 | 0.0010 | 7.04E-01 |
| node_pheno25 | adulthood_BMI | 716.4 | 1.29E-03 |  | 0.0004 | 0.0010 | 6.46E-01 |
| node_pheno26 | adulthood_BMI | 668.4 | 3.97E-02 |  | -0.0003 | 0.0009 | 7.44E-01 |
| node_pheno27 | adulthood_BMI | 733.6 | 3.07E-04 |  | -0.0003 | 0.0010 | 7.77E-01 |
| node_pheno28 | adulthood_BMI | 728.4 | 4.92E-04 |  | -0.0014 | 0.0010 | 1.61E-01 |
| node_pheno29 | adulthood_BMI | 688.7 | 1.18E-02 |  | -0.0006 | 0.0010 | 5.19E-01 |
| node_pheno3 | adulthood_BMI | 749.5 | 5.82E-05 |  | -0.0010 | 0.0010 | 3.40E-01 |
| node_pheno30 | adulthood_BMI | 667.4 | 4.48E-02 |  | -0.0021 | 0.0009 | 3.00E-02 |
| node_pheno31 | adulthood_BMI | 780.5 | 1.93E-06 |  | -0.0009 | 0.0010 | 3.86E-01 |
| node_pheno32 | adulthood_BMI | 689.0 | 1.07E-02 |  | 0.0005 | 0.0010 | 6.30E-01 |
| node_pheno33 | adulthood_BMI | 771.8 | 5.27E-06 |  | -0.0002 | 0.0010 | 8.62E-01 |
| node_pheno34 | adulthood_BMI | 769.5 | 6.84E-06 |  | -0.0005 | 0.0010 | 5.90E-01 |
| node_pheno35 | adulthood_BMI | 704.9 | 3.28E-03 |  | 0.0002 | 0.0010 | 8.27E-01 |
| node_pheno36 | adulthood_BMI | 746.8 | 8.51E-05 |  | -0.0003 | 0.0010 | 7.27E-01 |
| node_pheno37 | adulthood_BMI | 753.4 | 4.38E-05 |  | 0.0000 | 0.0010 | 9.78E-01 |
| node_pheno38 | adulthood_BMI | 797.9 | 2.71E-07 |  | -0.0009 | 0.0010 | 3.49E-01 |
| node_pheno39 | adulthood_BMI | 745.5 | 9.72E-05 |  | -0.0004 | 0.0010 | 6.58E-01 |
| node_pheno4 | adulthood_BMI | 708.1 | 2.78E-03 |  | -0.0006 | 0.0010 | 5.39E-01 |
| node_pheno40 | adulthood_BMI | 732.9 | 2.95E-04 |  | -0.0007 | 0.0010 | 4.76E-01 |
| node_pheno41 | adulthood_BMI | 748.3 | 7.36E-05 |  | -0.0002 | 0.0010 | 8.25E-01 |
| node_pheno42 | adulthood_BMI | 802.9 | 1.45E-07 |  | -0.0008 | 0.0010 | 4.46E-01 |
| node_pheno43 | adulthood_BMI | 793.2 | 4.85E-07 |  | -0.0013 | 0.0010 | 2.24E-01 |
| node_pheno44 | adulthood_BMI | 725.0 | 6.66E-04 |  | -0.0008 | 0.0009 | 3.86E-01 |
| node_pheno45 | adulthood_BMI | 681.5 | 1.77E-02 |  | -0.0006 | 0.0009 | 5.22E-01 |
| node_pheno46 | adulthood_BMI | 815.2 | 2.95E-08 |  | -0.0009 | 0.0011 | 3.78E-01 |
| node_pheno47 | adulthood_BMI | 708.9 | 2.38E-03 |  | -0.0011 | 0.0010 | 2.71E-01 |
| node_pheno48 | adulthood_BMI | 754.8 | 3.79E-05 |  | -0.0011 | 0.0010 | 2.78E-01 |
| node_pheno49 | adulthood_BMI | 838.2 | 1.26E-09 |  | -0.0009 | 0.0011 | 3.95E-01 |
| node_pheno5 | adulthood_BMI | 816.4 | 2.16E-08 |  | -0.0020 | 0.0011 | 6.12E-02 |
| node_pheno50 | adulthood_BMI | 733.7 | 3.03E-04 |  | -0.0003 | 0.0010 | 7.75E-01 |
| node_pheno51 | adulthood_BMI | 749.0 | 6.83E-05 |  | 0.0001 | 0.0010 | 9.31E-01 |
| node_pheno52 | adulthood_BMI | 769.1 | 7.16E-06 |  | 0.0000 | 0.0010 | 9.67E-01 |
| node_pheno53 | adulthood_BMI | 763.2 | 1.54E-05 |  | -0.0009 | 0.0010 | 3.93E-01 |
| node_pheno54 | adulthood_BMI | 721.0 | 9.45E-04 |  | -0.0002 | 0.0010 | 8.38E-01 |
| node_pheno55 | adulthood_BMI | 742.0 | 1.23E-04 |  | -0.0011 | 0.0010 | 2.79E-01 |
| node_pheno56 | adulthood_BMI | 767.1 | 1.01E-05 |  | -0.0006 | 0.0010 | 5.37E-01 |
| node_pheno57 | adulthood_BMI | 698.3 | 5.43E-03 |  | -0.0003 | 0.0009 | 7.13E-01 |
| node_pheno58 | adulthood_BMI | 777.0 | 2.91E-06 |  | -0.0004 | 0.0010 | 7.09E-01 |
| node_pheno59 | adulthood_BMI | 770.2 | 7.14E-06 |  | -0.0002 | 0.0009 | 8.02E-01 |
| node_pheno60 | adulthood_BMI | 723.3 | 7.08E-04 |  | -0.0004 | 0.0010 | 7.14E-01 |
| node_pheno61 | adulthood_BMI | 689.5 | 1.11E-02 |  | -0.0003 | 0.0010 | 7.39E-01 |
| node_pheno62 | adulthood_BMI | 697.3 | 6.37E-03 |  | -0.0009 | 0.0010 | 3.35E-01 |
| node_pheno63 | adulthood_BMI | 744.3 | 9.76E-05 |  | 0.0000 | 0.0010 | 9.89E-01 |
| node_pheno64 | adulthood_BMI | 712.1 | 2.01E-03 |  | -0.0008 | 0.0009 | 3.70E-01 |
| node_pheno65 | adulthood_BMI | 821.8 | 1.04E-08 |  | -0.0003 | 0.0010 | 7.42E-01 |
| node_pheno66 | adulthood_BMI | 743.1 | 1.10E-04 |  | 0.0000 | 0.0010 | 9.65E-01 |
| node_pheno67 | adulthood_BMI | 738.3 | 1.76E-04 |  | -0.0007 | 0.0010 | 4.92E-01 |
| node_pheno68 | adulthood_BMI | 717.8 | 1.14E-03 |  | -0.0004 | 0.0009 | 6.77E-01 |
| node_pheno69 | adulthood_BMI | 713.4 | 1.66E-03 |  | -0.0010 | 0.0010 | 2.83E-01 |
| node_pheno7 | adulthood_BMI | 792.4 | 4.67E-07 |  | -0.0007 | 0.0011 | 4.99E-01 |
| node_pheno70 | adulthood_BMI | 712.1 | 1.84E-03 |  | -0.0011 | 0.0009 | 2.65E-01 |
| node_pheno71 | adulthood_BMI | 736.2 | 2.39E-04 |  | 0.0001 | 0.0009 | 8.92E-01 |
| node_pheno72 | adulthood_BMI | 713.1 | 1.86E-03 |  | -0.0006 | 0.0009 | 4.65E-01 |
| node_pheno73 | adulthood_BMI | 797.5 | 2.85E-07 |  | -0.0011 | 0.0009 | 2.32E-01 |
| node_pheno74 | adulthood_BMI | 723.0 | 7.98E-04 |  | -0.0014 | 0.0010 | 1.65E-01 |
| node_pheno75 | adulthood_BMI | 662.0 | 6.04E-02 |  | -0.0002 | 0.0009 | 8.45E-01 |
| node_pheno76 | adulthood_BMI | 772.6 | 5.46E-06 |  | -0.0010 | 0.0009 | 2.48E-01 |
| node_pheno8 | adulthood_BMI | 692.1 | 8.55E-03 |  | -0.0006 | 0.0010 | 5.60E-01 |
| node_pheno9 | adulthood_BMI | 763.5 | 1.32E-05 |  | -0.0003 | 0.0010 | 7.44E-01 |

**Table S6. Screen the candidate proteomic mediators of the causal association between the life-course body weight and rsfMRI traits using two-step Mendelian randomization.**

| **exposure** | **mediator** | **outcome** | **exposure to mediator** | **se (exposure to mediator)** | **p (exposure to mediator)** | **mediator to outcome** | **se (mediator to outcome)** | **p (mediator to outcome)** | **indirect effect** | **se of indirect** | **proportion of mediation effect** | **se of mediation effect** | **lci_prop_mediated** | **uci_prop_mediated** | **indirect_Z** | **indirect_pval** |
| --- | --- | --- | --- | --- | --- | --- | --- | --- | --- | --- | --- | --- | --- | --- | --- | --- |
| birth weight | prot_a_1315_Hemoglobin_subunit_gamma_1 | node_pheno67 | -0.203 | 0.102 | 0.048 | 0.087 | 0.044 | 0.048 | -0.018 | 0.013 | 0.112 | 0.084 | -0.053 | 0.277 | -1.400 | 0.162 |
| birth weight | prot_a_1387_Heat_shock_70_kDa_protein_1_like | node_pheno67 | -0.268 | 0.098 | 0.006 | 0.033 | 0.016 | 0.036 | -0.009 | 0.005 | 0.055 | 0.036 | -0.015 | 0.125 | -1.662 | 0.097 |
| birth weight | prot_a_1526_Interleukin_36_alpha | node_pheno67 | -0.215 | 0.098 | 0.028 | 0.035 | 0.015 | 0.019 | -0.008 | 0.005 | 0.048 | 0.032 | -0.015 | 0.110 | -1.607 | 0.108 |
| birth weight | prot_a_1760_Ig_Kappa_chain_V_I_region_HK102_like | node_pheno67 | -0.296 | 0.115 | 0.010 | 0.143 | 0.047 | 0.002 | -0.042 | 0.022 | 0.267 | 0.150 | -0.028 | 0.562 | -1.960 | 0.050 |
| birth weight | prot_a_181_Activating_signal_cointegrator_1_complex_subunit_1 | node_pheno67 | -0.247 | 0.101 | 0.014 | 0.050 | 0.021 | 0.020 | -0.012 | 0.007 | 0.077 | 0.049 | -0.019 | 0.174 | -1.687 | 0.092 |
| birth weight | prot_a_1958_Metallothionein_1F | node_pheno67 | -0.317 | 0.099 | 0.001 | 0.037 | 0.015 | 0.015 | -0.012 | 0.006 | 0.074 | 0.042 | -0.009 | 0.157 | -1.936 | 0.053 |
| birth weight | prot_a_2071_Epididymal_secretory_protein_E1 | node_pheno67 | 0.259 | 0.098 | 0.008 | -0.015 | 0.007 | 0.024 | -0.004 | 0.002 | 0.025 | 0.016 | -0.006 | 0.056 | -1.719 | 0.086 |
| birth weight | prot_a_2074_Nuclear_pore_complex_interacting_protein_family_member_B3 | node_pheno67 | -0.238 | 0.100 | 0.017 | 0.047 | 0.019 | 0.012 | -0.011 | 0.007 | 0.071 | 0.045 | -0.016 | 0.159 | -1.725 | 0.085 |
| birth weight | prot_a_2133_Neurexophilin_3 | node_pheno67 | -0.294 | 0.112 | 0.009 | 0.147 | 0.049 | 0.002 | -0.043 | 0.022 | 0.273 | 0.126 | 0.026 | 0.520 | -1.981 | 0.048 |
| birth weight | prot_a_2519_Tumor_necrosis_factor_receptor_superfamily_member_19L | node_pheno67 | 0.243 | 0.102 | 0.017 | -0.068 | 0.029 | 0.019 | -0.016 | 0.010 | 0.104 | 0.067 | -0.027 | 0.235 | -1.674 | 0.094 |
| birth weight | prot_a_2536_Regulator_of_G_protein_signaling_19 | node_pheno67 | -0.242 | 0.098 | 0.014 | 0.060 | 0.023 | 0.008 | -0.015 | 0.008 | 0.092 | 0.055 | -0.017 | 0.200 | -1.803 | 0.071 |
| birth weight | prot_a_2744_SLAM_family_member_7 | node_pheno67 | -0.212 | 0.098 | 0.031 | 0.045 | 0.017 | 0.007 | -0.010 | 0.006 | 0.060 | 0.038 | -0.015 | 0.135 | -1.683 | 0.092 |
| birth weight | prot_a_300_Complement_component_1_Q_subcomponent_binding_protein_mitochondrial | node_pheno67 | -0.259 | 0.100 | 0.010 | 0.082 | 0.042 | 0.048 | -0.021 | 0.014 | 0.134 | 0.091 | -0.045 | 0.314 | -1.571 | 0.116 |
| birth weight | prot_a_319_Uncharacterized_protein_C6orf226 | node_pheno67 | 0.214 | 0.102 | 0.037 | -0.064 | 0.029 | 0.027 | -0.014 | 0.009 | 0.086 | 0.060 | -0.032 | 0.204 | -1.517 | 0.129 |
| birth weight | prot_a_472_M_phase_inducer_phosphatase_2 | node_pheno67 | -0.253 | 0.100 | 0.012 | 0.079 | 0.034 | 0.022 | -0.020 | 0.012 | 0.126 | 0.080 | -0.031 | 0.282 | -1.695 | 0.090 |
| birth weight | prot_a_541_Chitinase_3_like_protein_2 | node_pheno67 | -0.262 | 0.098 | 0.008 | 0.088 | 0.038 | 0.022 | -0.023 | 0.013 | 0.145 | 0.090 | -0.032 | 0.321 | -1.739 | 0.082 |
| birth weight | prot_a_564_Cytoskeleton_associated_protein_2 | node_pheno67 | -0.281 | 0.102 | 0.006 | 0.031 | 0.016 | 0.049 | -0.009 | 0.005 | 0.056 | 0.037 | -0.017 | 0.128 | -1.602 | 0.109 |
| birth weight | prot_a_586_Dual_specificity_protein_kinase_CLK2 | node_pheno67 | -0.235 | 0.098 | 0.016 | 0.031 | 0.012 | 0.012 | -0.007 | 0.004 | 0.046 | 0.029 | -0.010 | 0.102 | -1.740 | 0.082 |
| birth weight | prot_a_649_Carboxypeptidase_Z | node_pheno67 | 0.243 | 0.098 | 0.013 | -0.106 | 0.037 | 0.004 | -0.026 | 0.014 | 0.162 | 0.094 | -0.023 | 0.347 | -1.884 | 0.060 |
| childhood BMI | prot_a_13_Arf_GAP_with_coiled_coil_ANK_repeat_and_PH_domain_containing_protein_2 | edge_pheno698 | -0.236 | 0.102 | 0.021 | 0.074 | 0.034 | 0.030 | -0.018 | 0.011 | 0.240 | 0.184 | -0.121 | 0.600 | -1.580 | 0.114 |
| childhood BMI | prot_a_1757_LIM_and_cysteine_rich_domains_protein_1 | edge_pheno698 | 0.241 | 0.102 | 0.019 | -0.104 | 0.053 | 0.049 | -0.025 | 0.017 | 0.343 | 0.271 | -0.189 | 0.875 | -1.510 | 0.131 |
| childhood BMI | prot_a_3229_WAP_Kazal_immunoglobulin_Kunitz_and_NTR_domain_containing_protein_2 | edge_pheno698 | -0.302 | 0.125 | 0.016 | 0.023 | 0.010 | 0.027 | -0.007 | 0.004 | 0.093 | 0.070 | -0.044 | 0.230 | -1.627 | 0.104 |
| childhood BMI | prot_a_392_C_C_motif_chemokine_15 | edge_pheno698 | -0.224 | 0.102 | 0.029 | 0.019 | 0.007 | 0.005 | -0.004 | 0.002 | 0.059 | 0.042 | -0.025 | 0.142 | -1.725 | 0.084 |
| adulthood BMI | prot_a_3089_Tubulin_polymerization_promoting_protein_family_member_2 | node_pheno52 | 0.183 | 0.057 | 0.001 | 0.076 | 0.037 | 0.039 | 0.014 | 0.008 | 0.143 | 0.086 | -0.027 | 0.312 | 1.735 | 0.083 |
| adulthood BMI | prot_a_608_Contactin_4 | node_pheno52 | -0.113 | 0.056 | 0.042 | -0.049 | 0.017 | 0.005 | 0.006 | 0.003 | 0.056 | 0.036 | -0.014 | 0.127 | 1.648 | 0.099 |
| adulthood BMI | prot_a_990_Endothelial_cell_specific_molecule_1 | node_pheno52 | -0.210 | 0.056 | 0.000 | -0.065 | 0.032 | 0.043 | 0.014 | 0.008 | 0.139 | 0.082 | -0.022 | 0.301 | 1.781 | 0.075 |

**Table S7. Genetic correlations between investigated traits by linkage disequilibrium score regression.**

| **trait1** | **trait2** | ***r_g_*** | **standard error** | ***p* value** |
| --- | --- | --- | --- | --- |
| adulthood BMI | birth weight | 0.08 | 0.02 | 7.49E-04 |
| adulthood BMI | childhood BMI | 0.64 | 0.04 | 1.90E-57 |
| adulthood BMI | edge pheno1020 | 0.12 | 0.09 | 1.92E-01 |
| adulthood BMI | edge pheno1134 | 0.01 | 0.03 | 8.04E-01 |
| adulthood BMI | edge pheno288 | -0.07 | 0.05 | 1.64E-01 |
| adulthood BMI | edge pheno574 | -0.06 | 0.05 | 2.89E-01 |
| adulthood BMI | edge pheno698 | -0.13 | 0.07 | 6.00E-02 |
| adulthood BMI | node pheno29 | 0.06 | 0.05 | 2.22E-01 |
| adulthood BMI | node pheno36 | 0.08 | 0.04 | 3.64E-02 |
| adulthood BMI | node pheno50 | 0.05 | 0.04 | 1.94E-01 |
| adulthood BMI | node pheno52 | 0.09 | 0.04 | 4.85E-02 |
| adulthood BMI | node pheno58 | 0.05 | 0.04 | 2.09E-01 |
| adulthood BMI | node pheno64 | 0.11 | 0.04 | 4.02E-03 |
| adulthood BMI | node pheno66 | 0.07 | 0.04 | 6.68E-02 |
| adulthood BMI | node pheno67 | 0.07 | 0.04 | 8.01E-02 |
| adulthood BMI | node pheno73 | 0.09 | 0.04 | 2.58E-02 |
| adulthood BMI | node pheno76 | 0.12 | 0.04 | 6.07E-03 |
| birth weight | childhood BMI | 0.22 | 0.04 | 8.31E-08 |
| birth weight | edge pheno1020 | 0.12 | 0.15 | 3.96E-01 |
| birth weight | edge pheno1134 | -0.07 | 0.05 | 1.75E-01 |
| birth weight | edge pheno288 | 0.08 | 0.06 | 2.06E-01 |
| birth weight | edge pheno574 | 0.11 | 0.07 | 1.02E-01 |
| birth weight | edge pheno698 | -0.07 | 0.07 | 2.85E-01 |
| birth weight | node pheno29 | 0.02 | 0.07 | 7.78E-01 |
| birth weight | node pheno36 | 0.03 | 0.05 | 6.01E-01 |
| birth weight | node pheno50 | 0.06 | 0.06 | 2.77E-01 |
| birth weight | node pheno52 | 0.06 | 0.05 | 2.56E-01 |
| birth weight | node pheno58 | 0.03 | 0.05 | 5.79E-01 |
| birth weight | node pheno64 | -0.02 | 0.07 | 7.95E-01 |
| birth weight | node pheno66 | -0.03 | 0.06 | 6.22E-01 |
| birth weight | node pheno67 | -0.10 | 0.05 | 5.46E-02 |
| birth weight | node pheno73 | -0.01 | 0.06 | 9.14E-01 |
| birth weight | node pheno76 | 0.06 | 0.06 | 3.33E-01 |
| childhood BMI | edge pheno1020 | 0.04 | 0.18 | 8.12E-01 |
| childhood BMI | edge pheno1134 | -0.02 | 0.06 | 7.59E-01 |
| childhood BMI | edge pheno288 | -0.04 | 0.07 | 5.79E-01 |
| childhood BMI | edge pheno574 | -0.03 | 0.09 | 7.10E-01 |
| childhood BMI | edge pheno698 | -0.01 | 0.10 | 9.52E-01 |
| childhood BMI | node pheno29 | -0.03 | 0.08 | 6.52E-01 |
| childhood BMI | node pheno36 | 0.02 | 0.06 | 7.43E-01 |
| childhood BMI | node pheno50 | -0.01 | 0.06 | 8.83E-01 |
| childhood BMI | node pheno52 | 0.09 | 0.07 | 1.48E-01 |
| childhood BMI | node pheno58 | -0.01 | 0.07 | 8.91E-01 |
| childhood BMI | node pheno64 | 0.05 | 0.07 | 4.84E-01 |
| childhood BMI | node pheno66 | 0.02 | 0.07 | 7.39E-01 |
| childhood BMI | node pheno67 | 0.05 | 0.07 | 4.90E-01 |
| childhood BMI | node pheno73 | 0.04 | 0.08 | 5.58E-01 |
| childhood BMI | node pheno76 | 0.07 | 0.07 | 3.72E-01 |
| edge pheno1020 | edge pheno1134 | 0.02 | 0.20 | 9.41E-01 |
| edge pheno1020 | edge pheno288 | -0.16 | 0.24 | 5.03E-01 |
| edge pheno1020 | edge pheno574 | 0.14 | 0.30 | 6.51E-01 |
| edge pheno1020 | edge pheno698 | -1.04 | 0.39 | 8.28E-03 |
| edge pheno1020 | node pheno29 | 0.44 | 0.29 | 1.32E-01 |
| edge pheno1020 | node pheno36 | -0.06 | 0.24 | 8.14E-01 |
| edge pheno1020 | node pheno50 | 0.05 | 0.22 | 8.04E-01 |
| edge pheno1020 | node pheno52 | -0.40 | 0.22 | 7.31E-02 |
| edge pheno1020 | node pheno58 | 0.11 | 0.23 | 6.36E-01 |
| edge pheno1020 | node pheno64 | 0.31 | 0.27 | 2.56E-01 |
| edge pheno1020 | node pheno66 | -0.21 | 0.24 | 3.77E-01 |
| edge pheno1020 | node pheno67 | -0.32 | 0.20 | 1.01E-01 |
| edge pheno1020 | node pheno73 | -0.21 | 0.26 | 4.18E-01 |
| edge pheno1020 | node pheno76 | -0.22 | 0.25 | 3.68E-01 |
| edge pheno1134 | edge pheno288 | -0.27 | 0.08 | 5.88E-04 |
| edge pheno1134 | edge pheno574 | 0.09 | 0.11 | 4.10E-01 |
| edge pheno1134 | edge pheno698 | -0.07 | 0.11 | 5.18E-01 |
| edge pheno1134 | node pheno29 | 0.21 | 0.09 | 1.84E-02 |
| edge pheno1134 | node pheno36 | 0.19 | 0.07 | 1.13E-02 |
| edge pheno1134 | node pheno50 | -0.08 | 0.07 | 3.03E-01 |
| edge pheno1134 | node pheno52 | 0.20 | 0.07 | 4.16E-03 |
| edge pheno1134 | node pheno58 | 0.13 | 0.07 | 8.09E-02 |
| edge pheno1134 | node pheno64 | 0.10 | 0.09 | 2.89E-01 |
| edge pheno1134 | node pheno66 | 0.22 | 0.07 | 2.32E-03 |
| edge pheno1134 | node pheno67 | -0.04 | 0.07 | 5.68E-01 |
| edge pheno1134 | node pheno73 | 0.06 | 0.08 | 5.04E-01 |
| edge pheno1134 | node pheno76 | 0.03 | 0.08 | 7.31E-01 |
| edge pheno288 | edge pheno574 | -0.32 | 0.15 | 3.03E-02 |
| edge pheno288 | edge pheno698 | 0.09 | 0.16 | 5.74E-01 |
| edge pheno288 | node pheno29 | 0.07 | 0.09 | 4.55E-01 |
| edge pheno288 | node pheno36 | 0.11 | 0.09 | 2.42E-01 |
| edge pheno288 | node pheno50 | 0.12 | 0.09 | 1.97E-01 |
| edge pheno288 | node pheno52 | -0.05 | 0.11 | 6.46E-01 |
| edge pheno288 | node pheno58 | 0.04 | 0.11 | 7.47E-01 |
| edge pheno288 | node pheno64 | 0.21 | 0.09 | 2.68E-02 |
| edge pheno288 | node pheno66 | -0.05 | 0.10 | 6.01E-01 |
| edge pheno288 | node pheno67 | -0.11 | 0.10 | 2.74E-01 |
| edge pheno288 | node pheno73 | 0.11 | 0.11 | 3.45E-01 |
| edge pheno288 | node pheno76 | 0.00 | 0.11 | 9.69E-01 |
| edge pheno574 | edge pheno698 | -0.21 | 0.18 | 2.50E-01 |
| edge pheno574 | node pheno29 | 0.01 | 0.12 | 9.10E-01 |
| edge pheno574 | node pheno36 | -0.09 | 0.11 | 4.31E-01 |
| edge pheno574 | node pheno50 | 0.04 | 0.12 | 7.55E-01 |
| edge pheno574 | node pheno52 | 0.01 | 0.12 | 9.13E-01 |
| edge pheno574 | node pheno58 | 0.02 | 0.12 | 8.62E-01 |
| edge pheno574 | node pheno64 | 0.03 | 0.12 | 8.37E-01 |
| edge pheno574 | node pheno66 | 0.09 | 0.13 | 4.90E-01 |
| edge pheno574 | node pheno67 | 0.09 | 0.12 | 4.36E-01 |
| edge pheno574 | node pheno73 | 0.04 | 0.11 | 7.31E-01 |
| edge pheno574 | node pheno76 | 0.00 | 0.12 | 9.82E-01 |
| edge pheno698 | node pheno29 | -0.53 | 0.15 | 3.23E-04 |
| edge pheno698 | node pheno36 | -0.25 | 0.15 | 9.14E-02 |
| edge pheno698 | node pheno50 | 0.03 | 0.12 | 8.00E-01 |
| edge pheno698 | node pheno52 | 0.07 | 0.16 | 6.71E-01 |
| edge pheno698 | node pheno58 | -0.34 | 0.15 | 2.43E-02 |
| edge pheno698 | node pheno64 | -0.42 | 0.14 | 3.15E-03 |
| edge pheno698 | node pheno66 | -0.11 | 0.14 | 4.40E-01 |
| edge pheno698 | node pheno67 | -0.28 | 0.15 | 6.28E-02 |
| edge pheno698 | node pheno73 | -0.36 | 0.16 | 3.03E-02 |
| edge pheno698 | node pheno76 | -0.15 | 0.14 | 2.56E-01 |
| node pheno29 | node pheno36 | 0.62 | 0.13 | 6.78E-07 |
| node pheno29 | node pheno50 | 0.45 | 0.13 | 5.58E-04 |
| node pheno29 | node pheno52 | 0.41 | 0.12 | 6.74E-04 |
| node pheno29 | node pheno58 | 0.52 | 0.12 | 1.10E-05 |
| node pheno29 | node pheno64 | 0.47 | 0.14 | 5.81E-04 |
| node pheno29 | node pheno66 | 0.59 | 0.13 | 8.11E-06 |
| node pheno29 | node pheno67 | 0.38 | 0.11 | 8.98E-04 |
| node pheno29 | node pheno73 | 0.60 | 0.13 | 7.10E-06 |
| node pheno29 | node pheno76 | 0.56 | 0.13 | 1.63E-05 |
| node pheno36 | node pheno50 | 0.76 | 0.11 | 3.04E-11 |
| node pheno36 | node pheno52 | 0.76 | 0.12 | 8.82E-11 |
| node pheno36 | node pheno58 | 0.78 | 0.12 | 2.95E-11 |
| node pheno36 | node pheno64 | 0.66 | 0.12 | 1.36E-08 |
| node pheno36 | node pheno66 | 0.81 | 0.12 | 2.38E-11 |
| node pheno36 | node pheno67 | 0.62 | 0.11 | 1.59E-08 |
| node pheno36 | node pheno73 | 0.76 | 0.12 | 2.95E-10 |
| node pheno36 | node pheno76 | 0.79 | 0.12 | 9.10E-11 |
| node pheno50 | node pheno52 | 0.72 | 0.11 | 1.88E-10 |
| node pheno50 | node pheno58 | 0.73 | 0.11 | 6.14E-11 |
| node pheno50 | node pheno64 | 0.63 | 0.13 | 4.95E-07 |
| node pheno50 | node pheno66 | 0.79 | 0.13 | 8.45E-10 |
| node pheno50 | node pheno67 | 0.65 | 0.10 | 8.14E-11 |
| node pheno50 | node pheno73 | 0.66 | 0.12 | 3.60E-08 |
| node pheno50 | node pheno76 | 0.78 | 0.12 | 2.66E-10 |
| node pheno52 | node pheno58 | 0.74 | 0.12 | 3.08E-09 |
| node pheno52 | node pheno64 | 0.65 | 0.12 | 3.71E-08 |
| node pheno52 | node pheno66 | 0.78 | 0.13 | 3.21E-09 |
| node pheno52 | node pheno67 | 0.58 | 0.11 | 3.23E-07 |
| node pheno52 | node pheno73 | 0.71 | 0.11 | 6.36E-10 |
| node pheno52 | node pheno76 | 0.84 | 0.12 | 9.31E-12 |
| node pheno58 | node pheno64 | 0.67 | 0.12 | 1.29E-08 |
| node pheno58 | node pheno66 | 0.85 | 0.12 | 4.97E-12 |
| node pheno58 | node pheno67 | 0.52 | 0.11 | 5.04E-06 |
| node pheno58 | node pheno73 | 0.73 | 0.12 | 1.52E-09 |
| node pheno58 | node pheno76 | 0.81 | 0.12 | 3.68E-11 |
| node pheno64 | node pheno66 | 0.74 | 0.13 | 5.79E-09 |
| node pheno64 | node pheno67 | 0.60 | 0.11 | 3.28E-08 |
| node pheno64 | node pheno73 | 0.82 | 0.13 | 3.19E-10 |
| node pheno64 | node pheno76 | 0.68 | 0.13 | 7.88E-08 |
| node pheno66 | node pheno67 | 0.52 | 0.12 | 1.29E-05 |
| node pheno66 | node pheno73 | 0.76 | 0.13 | 6.08E-09 |
| node pheno66 | node pheno76 | 0.82 | 0.13 | 2.20E-10 |
| node pheno67 | node pheno73 | 0.78 | 0.12 | 3.95E-11 |
| node pheno67 | node pheno76 | 0.55 | 0.12 | 2.47E-06 |
| node pheno73 | node pheno76 | 0.81 | 0.12 | 2.45E-11 |

**Table S8. The heritability of investigated traits by linkage disequilibrium score regression.**

| **Trait** | **Mean Chisq** | **Lambda GC** | **Intercept** | **Intercept SE** | **Ratio** | **Ratio SE** | **h^2^** | **h^2^ SE** | **h^2^ Z** | **h^2^ *p* value** |
| --- | --- | --- | --- | --- | --- | --- | --- | --- | --- | --- |
| adulthood BMI | 3.942 | 2.746 | 1.072 | 0.031 | 0.024 | 0.011 | 0.212 | 0.007 | 31.337 | 1.46E-215 |
| birth weight | 1.316 | 1.220 | 1.066 | 0.013 | 0.210 | 0.040 | 0.087 | 0.007 | 12.237 | 1.96E-34 |
| childhood BMI | 1.179 | 1.154 | 1.016 | 0.009 | 0.087 | 0.049 | 0.206 | 0.018 | 11.396 | 4.39E-30 |
| edge pheno1020 | 1.009 | 1.004 | 0.999 | 0.007 | -0.108 | 0.737 | 0.014 | 0.012 | 1.130 | 2.58E-01 |
| edge pheno1134 | 1.114 | 1.098 | 1.009 | 0.007 | 0.082 | 0.065 | 0.150 | 0.015 | 9.709 | 2.76E-22 |
| edge pheno288 | 1.067 | 1.052 | 0.985 | 0.007 | -0.221 | 0.110 | 0.116 | 0.017 | 6.708 | 1.97E-11 |
| edge pheno574 | 1.059 | 1.058 | 1.010 | 0.007 | 0.163 | 0.125 | 0.070 | 0.014 | 4.902 | 9.48E-07 |
| edge pheno698 | 1.039 | 1.035 | 1.004 | 0.008 | 0.092 | 0.206 | 0.049 | 0.016 | 3.120 | 1.81E-03 |
| node pheno29 | 1.065 | 1.051 | 1.009 | 0.007 | 0.140 | 0.112 | 0.079 | 0.014 | 5.570 | 2.55E-08 |
| node pheno36 | 1.110 | 1.093 | 1.009 | 0.010 | 0.081 | 0.086 | 0.145 | 0.022 | 6.723 | 1.78E-11 |
| node pheno50 | 1.100 | 1.085 | 1.010 | 0.008 | 0.097 | 0.082 | 0.129 | 0.017 | 7.599 | 2.99E-14 |
| node pheno52 | 1.121 | 1.098 | 1.010 | 0.009 | 0.085 | 0.075 | 0.153 | 0.021 | 7.260 | 3.87E-13 |
| node pheno58 | 1.121 | 1.091 | 1.015 | 0.008 | 0.126 | 0.067 | 0.147 | 0.020 | 7.452 | 9.23E-14 |
| node pheno64 | 1.071 | 1.059 | 1.007 | 0.008 | 0.092 | 0.113 | 0.094 | 0.015 | 6.153 | 7.58E-10 |
| node pheno66 | 1.102 | 1.084 | 1.014 | 0.008 | 0.138 | 0.078 | 0.123 | 0.018 | 6.662 | 2.70E-11 |
| node pheno67 | 1.147 | 1.114 | 1.027 | 0.009 | 0.184 | 0.061 | 0.170 | 0.022 | 7.647 | 2.06E-14 |
| node pheno73 | 1.077 | 1.068 | 1.006 | 0.007 | 0.076 | 0.087 | 0.101 | 0.015 | 6.809 | 9.81E-12 |
| node pheno76 | 1.087 | 1.073 | 1.007 | 0.007 | 0.083 | 0.086 | 0.113 | 0.017 | 6.623 | 3.51E-11 |

**Table S9. Common genes between life-course body weight and rsfMRI traits in TWAS analysis.**

| **life-course body weight** | **rsfMRI traits** | **tissue** | **gene** | **TWAS of exposure trait** | | |  | **TWAS of outcome trait** | | |  | **Fisher’s Combined p** |
| --- | --- | --- | --- | --- | --- | --- | --- | --- | --- | --- | --- | --- |
|  |  |  |  | **TWAS p value** | **TWAS p value (FDR)** | **COLOC.PP4** |  | **TWAS p value** | **TWAS p value (FDR)** | **COLOC.PP4** |  |  |
| birth weight | node_pheno67 | GTExv8.EUR.Brain_Hippocampus | LINC01165 | 2.80E-02 | 2.95E-01 | 1.90E-02 |  | 8.56E-17 | 3.02E-13 | 1.20E-02 |  | 9.96E-17 |
| birth weight | node_pheno67 | GTExv8.EUR.Brain_Hippocampus | AMZ1 | 1.23E-05 | 4.19E-03 | 8.18E-01 |  | 9.17E-03 | 3.73E-01 | 8.15E-01 |  | 1.92E-06 |
| birth weight | node_pheno67 | GTExv8.EUR.Brain_Hippocampus | FBP1 | 1.30E-05 | 4.19E-03 | 6.92E-01 |  | 1.04E-02 | 3.73E-01 | 5.00E-03 |  | 2.26E-06 |
| birth weight | node_pheno67 | GTExv8.EUR.Brain_Hippocampus | PPIEL | 4.34E-05 | 1.03E-02 | 2.03E-01 |  | 9.64E-03 | 3.73E-01 | 4.00E-03 |  | 6.56E-06 |
| birth weight | node_pheno67 | GTExv8.EUR.Brain_Hippocampus | FLJ27354 | 9.58E-03 | 1.94E-01 | 2.00E-03 |  | 1.69E-04 | 3.97E-02 | 3.50E-02 |  | 2.32E-05 |
| birth weight | node_pheno67 | GTExv8.EUR.Brain_Hippocampus | RP11-1055B8.3 | 3.90E-04 | 3.36E-02 | 4.79E-01 |  | 9.76E-03 | 3.73E-01 | 8.80E-02 |  | 5.13E-05 |
| birth weight | node_pheno67 | GTExv8.EUR.Brain_Hippocampus | ENTPD5 | 1.40E-04 | 2.07E-02 | 1.20E-02 |  | 4.83E-02 | 6.13E-01 | 0.00E+00 |  | 8.72E-05 |
| birth weight | node_pheno67 | GTExv8.EUR.Brain_Hippocampus | LRWD1 | 2.93E-03 | 1.10E-01 | 2.00E-03 |  | 3.08E-03 | 2.13E-01 | 3.00E-03 |  | 1.14E-04 |
| birth weight | node_pheno67 | GTExv8.EUR.Brain_Hippocampus | ZNF680P1 | 2.75E-02 | 2.94E-01 | 3.00E-03 |  | 4.80E-04 | 8.05E-02 | 1.35E-01 |  | 1.62E-04 |
| birth weight | node_pheno67 | GTExv8.EUR.Brain_Hippocampus | SPATA7 | 1.10E-03 | 5.57E-02 | 2.57E-01 |  | 1.28E-02 | 4.18E-01 | 7.40E-02 |  | 1.71E-04 |
| birth weight | node_pheno67 | GTExv8.EUR.Brain_Hippocampus | RPS9 | 5.74E-04 | 4.33E-02 | 9.05E-01 |  | 2.80E-02 | 5.29E-01 | 1.06E-01 |  | 1.94E-04 |
| birth weight | node_pheno67 | GTExv8.EUR.Brain_Hippocampus | RP11-389C8.1 | 2.26E-03 | 9.42E-02 | 1.50E-02 |  | 1.01E-02 | 3.73E-01 | 9.20E-02 |  | 2.66E-04 |
| birth weight | node_pheno67 | GTExv8.EUR.Brain_Hippocampus | RAB3IP | 2.50E-02 | 2.87E-01 | 9.00E-03 |  | 3.95E-03 | 2.58E-01 | 4.70E-02 |  | 1.01E-03 |
| birth weight | node_pheno67 | GTExv8.EUR.Brain_Hippocampus | MYRFL | 2.26E-02 | 2.74E-01 | 1.80E-02 |  | 5.22E-03 | 3.04E-01 | 2.00E-01 |  | 1.19E-03 |
| birth weight | node_pheno67 | GTExv8.EUR.Brain_Hippocampus | SRR | 2.97E-03 | 1.11E-01 | 3.39E-01 |  | 4.25E-02 | 5.76E-01 | 4.40E-02 |  | 1.26E-03 |
| birth weight | node_pheno67 | GTExv8.EUR.Brain_Hippocampus | ZSCAN21 | 4.06E-02 | 3.53E-01 | 2.00E-03 |  | 3.61E-03 | 2.40E-01 | 4.00E-03 |  | 1.44E-03 |
| birth weight | node_pheno67 | GTExv8.EUR.Brain_Hippocampus | FADS1 | 2.60E-02 | 2.91E-01 | 2.00E-02 |  | 5.74E-03 | 3.16E-01 | 1.35E-01 |  | 1.46E-03 |
| birth weight | node_pheno67 | GTExv8.EUR.Brain_Hippocampus | RP5-943J3.1 | 2.80E-02 | 2.95E-01 | 1.90E-02 |  | 5.45E-03 | 3.10E-01 | 1.42E-01 |  | 1.49E-03 |
| birth weight | node_pheno67 | GTExv8.EUR.Brain_Hippocampus | ATP6V0A4 | 3.35E-02 | 3.23E-01 | 1.00E-03 |  | 4.63E-03 | 2.91E-01 | 1.42E-01 |  | 1.51E-03 |
| birth weight | node_pheno67 | GTExv8.EUR.Brain_Hippocampus | NPEPPS | 8.99E-03 | 1.86E-01 | 2.70E-02 |  | 2.05E-02 | 4.79E-01 | 4.10E-02 |  | 1.77E-03 |
| birth weight | node_pheno67 | GTExv8.EUR.Brain_Hippocampus | 45537 | 3.75E-03 | 1.21E-01 | 9.80E-02 |  | 4.98E-02 | 6.20E-01 | 1.10E-02 |  | 1.79E-03 |
| birth weight | node_pheno67 | GTExv8.EUR.Brain_Hippocampus | SLC25A17 | 8.27E-03 | 1.77E-01 | 2.00E-03 |  | 2.40E-02 | 4.98E-01 | 8.00E-03 |  | 1.89E-03 |
| birth weight | node_pheno67 | GTExv8.EUR.Brain_Hippocampus | MAP6 | 4.59E-03 | 1.39E-01 | 1.00E-03 |  | 4.39E-02 | 5.86E-01 | 1.00E-03 |  | 1.92E-03 |
| birth weight | node_pheno67 | GTExv8.EUR.Brain_Hippocampus | MXRA7 | 1.10E-02 | 2.05E-01 | 4.10E-02 |  | 2.16E-02 | 4.92E-01 | 4.80E-02 |  | 2.22E-03 |
| birth weight | node_pheno67 | GTExv8.EUR.Brain_Hippocampus | PSRC1 | 4.02E-02 | 3.53E-01 | 3.00E-03 |  | 5.97E-03 | 3.23E-01 | 9.30E-02 |  | 2.24E-03 |
| birth weight | node_pheno67 | GTExv8.EUR.Brain_Hippocampus | NLRC3 | 1.00E-02 | 1.97E-01 | 1.10E-02 |  | 2.58E-02 | 5.15E-01 | 1.80E-02 |  | 2.39E-03 |
| birth weight | node_pheno67 | GTExv8.EUR.Brain_Hippocampus | EFCAB13 | 1.23E-02 | 2.15E-01 | 3.20E-02 |  | 2.87E-02 | 5.29E-01 | 4.90E-02 |  | 3.16E-03 |
| birth weight | node_pheno67 | GTExv8.EUR.Brain_Hippocampus | YDJC | 2.59E-02 | 2.91E-01 | 1.70E-02 |  | 1.71E-02 | 4.59E-01 | 5.70E-02 |  | 3.85E-03 |
| birth weight | node_pheno67 | GTExv8.EUR.Brain_Hippocampus | MFN1 | 1.77E-02 | 2.55E-01 | 3.97E-01 |  | 3.49E-02 | 5.45E-01 | 9.00E-03 |  | 5.19E-03 |
| birth weight | node_pheno67 | GTExv8.EUR.Brain_Hippocampus | GBP3 | 2.02E-02 | 2.67E-01 | 2.40E-02 |  | 3.20E-02 | 5.33E-01 | 3.10E-02 |  | 5.40E-03 |
| birth weight | node_pheno67 | GTExv8.EUR.Brain_Hippocampus | CCDC116 | 3.89E-02 | 3.49E-01 | 1.40E-02 |  | 1.88E-02 | 4.76E-01 | 5.10E-02 |  | 6.01E-03 |
| birth weight | node_pheno67 | GTExv8.EUR.Brain_Hippocampus | RP3-508I15.9 | 3.32E-02 | 3.21E-01 | 8.00E-03 |  | 2.61E-02 | 5.15E-01 | 1.50E-02 |  | 6.97E-03 |
| birth weight | node_pheno67 | GTExv8.EUR.Brain_Hippocampus | CCDC163 | 3.60E-02 | 3.36E-01 | 1.30E-02 |  | 2.91E-02 | 5.29E-01 | 3.10E-02 |  | 8.24E-03 |
| birth weight | node_pheno67 | GTExv8.EUR.Brain_Hippocampus | RP11-109L13.1 | 4.88E-02 | 3.88E-01 | 1.00E-02 |  | 2.36E-02 | 4.98E-01 | 4.30E-02 |  | 8.94E-03 |
| birth weight | node_pheno67 | GTExv8.EUR.Brain_Hippocampus | CTD-2162K18.4 | 3.10E-02 | 3.06E-01 | 4.00E-03 |  | 4.02E-02 | 5.73E-01 | 3.00E-03 |  | 9.58E-03 |
| birth weight | node_pheno67 | GTExv8.EUR.Brain_Hippocampus | ADH5 | 4.70E-02 | 3.83E-01 | 3.00E-03 |  | 2.66E-02 | 5.15E-01 | 9.00E-03 |  | 9.61E-03 |
| birth weight | node_pheno67 | GTExv8.EUR.Brain_Hippocampus | TMEM167A | 4.75E-02 | 3.86E-01 | 3.00E-03 |  | 4.84E-02 | 6.13E-01 | 1.00E-02 |  | 1.63E-02 |
| childhood BMI | edge_pheno698 | GTExv8.EUR.Brain_Cerebellum | SEPT7P2 | 1.87E-03 | 1.74E-01 | 3.04E-01 |  | 5.63E-05 | 1.36E-01 | 9.15E-01 |  | 1.80E-06 |
| childhood BMI | edge_pheno698 | GTExv8.EUR.Brain_Cerebellum | RAB29 | 2.63E-04 | 5.96E-02 | 6.40E-01 |  | 2.17E-03 | 5.23E-01 | 2.79E-01 |  | 8.78E-06 |
| childhood BMI | edge_pheno698 | GTExv8.EUR.Brain_Cerebellum | SNX32 | 1.54E-03 | 1.58E-01 | 3.68E-01 |  | 2.59E-03 | 5.49E-01 | 2.48E-01 |  | 5.37E-05 |
| childhood BMI | edge_pheno698 | GTExv8.EUR.Brain_Cerebellum | BBS1 | 4.35E-04 | 8.26E-02 | 8.90E-02 |  | 1.63E-02 | 7.18E-01 | 4.00E-03 |  | 9.14E-05 |
| childhood BMI | edge_pheno698 | GTExv8.EUR.Brain_Cerebellum | POLK | 1.98E-03 | 1.80E-01 | 5.40E-02 |  | 5.53E-03 | 5.89E-01 | 2.24E-01 |  | 1.36E-04 |
| childhood BMI | edge_pheno698 | GTExv8.EUR.Brain_Cerebellum | SLC41A1 | 3.45E-04 | 7.15E-02 | 9.48E-01 |  | 4.23E-02 | 7.90E-01 | 6.86E-01 |  | 1.77E-04 |
| childhood BMI | edge_pheno698 | GTExv8.EUR.Brain_Cerebellum | SCRN2 | 1.86E-03 | 1.74E-01 | 6.90E-02 |  | 1.03E-02 | 7.04E-01 | 4.00E-02 |  | 2.28E-04 |
| childhood BMI | edge_pheno698 | GTExv8.EUR.Brain_Cerebellum | GCH1 | 9.96E-04 | 1.26E-01 | 5.70E-02 |  | 2.89E-02 | 7.78E-01 | 1.20E-01 |  | 3.30E-04 |
| childhood BMI | edge_pheno698 | GTExv8.EUR.Brain_Cerebellum | TCF7L2 | 1.57E-03 | 1.58E-01 | 2.08E-01 |  | 2.26E-02 | 7.65E-01 | 1.60E-02 |  | 4.00E-04 |
| childhood BMI | edge_pheno698 | GTExv8.EUR.Brain_Cerebellum | RP11-631N16.2 | 5.70E-03 | 3.08E-01 | 1.00E-02 |  | 1.29E-02 | 7.09E-01 | 7.00E-03 |  | 7.71E-04 |
| childhood BMI | edge_pheno698 | GTExv8.EUR.Brain_Cerebellum | SIL1 | 9.72E-03 | 3.73E-01 | 6.00E-02 |  | 7.72E-03 | 6.84E-01 | 1.00E-01 |  | 7.88E-04 |
| childhood BMI | edge_pheno698 | GTExv8.EUR.Brain_Cerebellum | L2HGDH | 3.05E-03 | 2.21E-01 | 9.30E-02 |  | 2.54E-02 | 7.78E-01 | 4.00E-02 |  | 8.10E-04 |
| childhood BMI | edge_pheno698 | GTExv8.EUR.Brain_Cerebellum | AGK | 4.54E-02 | 5.64E-01 | 2.00E-03 |  | 1.88E-03 | 5.23E-01 | 2.00E-03 |  | 8.86E-04 |
| childhood BMI | edge_pheno698 | GTExv8.EUR.Brain_Cerebellum | FAM90A25P | 2.69E-02 | 5.03E-01 | 2.00E-03 |  | 3.30E-03 | 5.69E-01 | 7.00E-03 |  | 9.18E-04 |
| childhood BMI | edge_pheno698 | GTExv8.EUR.Brain_Cerebellum | MED19 | 1.98E-03 | 1.80E-01 | 2.15E-01 |  | 4.49E-02 | 7.90E-01 | 1.40E-02 |  | 9.19E-04 |
| childhood BMI | edge_pheno698 | GTExv8.EUR.Brain_Cerebellum | NAGA | 2.84E-03 | 2.17E-01 | 2.17E-01 |  | 3.27E-02 | 7.83E-01 | 2.90E-02 |  | 9.55E-04 |
| childhood BMI | edge_pheno698 | GTExv8.EUR.Brain_Cerebellum | DRAP1 | 2.28E-03 | 1.99E-01 | 9.20E-02 |  | 4.20E-02 | 7.90E-01 | 2.00E-02 |  | 9.81E-04 |
| childhood BMI | edge_pheno698 | GTExv8.EUR.Brain_Cerebellum | AF131215.9 | 3.47E-02 | 5.26E-01 | 2.30E-02 |  | 3.03E-03 | 5.69E-01 | 1.88E-01 |  | 1.07E-03 |
| childhood BMI | edge_pheno698 | GTExv8.EUR.Brain_Cerebellum | XYLB | 2.00E-02 | 4.60E-01 | 9.30E-02 |  | 6.03E-03 | 5.89E-01 | 4.00E-03 |  | 1.21E-03 |
| childhood BMI | edge_pheno698 | GTExv8.EUR.Brain_Cerebellum | SESTD1 | 3.96E-02 | 5.53E-01 | 2.50E-02 |  | 3.34E-03 | 5.69E-01 | 2.26E-01 |  | 1.31E-03 |
| childhood BMI | edge_pheno698 | GTExv8.EUR.Brain_Cerebellum | AP4M1 | 4.00E-02 | 5.53E-01 | 3.00E-02 |  | 4.17E-03 | 5.69E-01 | 1.56E-01 |  | 1.62E-03 |
| childhood BMI | edge_pheno698 | GTExv8.EUR.Brain_Cerebellum | RP11-481A20.4 | 3.70E-02 | 5.35E-01 | 2.40E-02 |  | 4.81E-03 | 5.69E-01 | 1.08E-01 |  | 1.71E-03 |
| childhood BMI | edge_pheno698 | GTExv8.EUR.Brain_Cerebellum | ATP5S | 5.62E-03 | 3.06E-01 | 4.00E-03 |  | 3.41E-02 | 7.85E-01 | 2.00E-03 |  | 1.83E-03 |
| childhood BMI | edge_pheno698 | GTExv8.EUR.Brain_Cerebellum | GK5 | 1.84E-02 | 4.48E-01 | 7.00E-03 |  | 1.11E-02 | 7.04E-01 | 2.80E-02 |  | 1.94E-03 |
| childhood BMI | edge_pheno698 | GTExv8.EUR.Brain_Cerebellum | B4GALT7 | 4.65E-02 | 5.66E-01 | 1.20E-02 |  | 4.40E-03 | 5.69E-01 | 6.80E-02 |  | 1.94E-03 |
| childhood BMI | edge_pheno698 | GTExv8.EUR.Brain_Cerebellum | TNKS2-AS1 | 9.76E-03 | 3.73E-01 | 8.60E-02 |  | 2.10E-02 | 7.50E-01 | 4.90E-02 |  | 1.94E-03 |
| childhood BMI | edge_pheno698 | GTExv8.EUR.Brain_Cerebellum | RP11-524F11.1 | 7.65E-03 | 3.53E-01 | 1.00E-03 |  | 2.72E-02 | 7.78E-01 | 1.00E-03 |  | 1.97E-03 |
| childhood BMI | edge_pheno698 | GTExv8.EUR.Brain_Cerebellum | ZFP62 | 2.31E-02 | 4.88E-01 | 4.20E-02 |  | 9.57E-03 | 7.04E-01 | 9.40E-02 |  | 2.08E-03 |
| childhood BMI | edge_pheno698 | GTExv8.EUR.Brain_Cerebellum | RP4-753D10.3 | 1.28E-02 | 4.03E-01 | 1.06E-01 |  | 2.27E-02 | 7.65E-01 | 8.00E-03 |  | 2.66E-03 |
| childhood BMI | edge_pheno698 | GTExv8.EUR.Brain_Cerebellum | SRR | 1.12E-02 | 3.79E-01 | 7.80E-02 |  | 2.72E-02 | 7.78E-01 | 3.80E-02 |  | 2.77E-03 |
| childhood BMI | edge_pheno698 | GTExv8.EUR.Brain_Cerebellum | ITIH4 | 1.55E-02 | 4.41E-01 | 1.24E-01 |  | 2.01E-02 | 7.48E-01 | 1.79E-01 |  | 2.83E-03 |
| childhood BMI | edge_pheno698 | GTExv8.EUR.Brain_Cerebellum | TOM1L2 | 8.12E-03 | 3.53E-01 | 2.90E-02 |  | 3.89E-02 | 7.90E-01 | 9.00E-03 |  | 2.86E-03 |
| childhood BMI | edge_pheno698 | GTExv8.EUR.Brain_Cerebellum | TXNDC15 | 4.56E-02 | 5.64E-01 | 4.30E-02 |  | 7.96E-03 | 6.84E-01 | 1.51E-01 |  | 3.24E-03 |
| childhood BMI | edge_pheno698 | GTExv8.EUR.Brain_Cerebellum | AC005517.3 | 1.30E-02 | 4.03E-01 | 3.40E-02 |  | 2.88E-02 | 7.78E-01 | 1.50E-02 |  | 3.33E-03 |
| childhood BMI | edge_pheno698 | GTExv8.EUR.Brain_Cerebellum | PXMP4 | 3.29E-02 | 5.19E-01 | 3.00E-03 |  | 1.32E-02 | 7.09E-01 | 8.00E-03 |  | 3.80E-03 |
| childhood BMI | edge_pheno698 | GTExv8.EUR.Brain_Cerebellum | GOLGA3 | 4.89E-02 | 5.71E-01 | 3.00E-03 |  | 9.10E-03 | 6.92E-01 | 8.00E-03 |  | 3.88E-03 |
| childhood BMI | edge_pheno698 | GTExv8.EUR.Brain_Cerebellum | PMS2CL | 4.11E-02 | 5.53E-01 | 1.60E-02 |  | 1.14E-02 | 7.04E-01 | 4.10E-02 |  | 4.06E-03 |
| childhood BMI | edge_pheno698 | GTExv8.EUR.Brain_Cerebellum | CBFA2T2 | 3.19E-02 | 5.15E-01 | 3.20E-02 |  | 1.71E-02 | 7.37E-01 | 5.70E-02 |  | 4.64E-03 |
| childhood BMI | edge_pheno698 | GTExv8.EUR.Brain_Cerebellum | LRFN5 | 3.76E-02 | 5.40E-01 | 3.90E-02 |  | 1.60E-02 | 7.18E-01 | 7.40E-02 |  | 5.05E-03 |
| childhood BMI | edge_pheno698 | GTExv8.EUR.Brain_Cerebellum | PRRC1 | 3.37E-02 | 5.24E-01 | 1.66E-01 |  | 1.81E-02 | 7.48E-01 | 2.00E-03 |  | 5.12E-03 |
| childhood BMI | edge_pheno698 | GTExv8.EUR.Brain_Cerebellum | TACC2 | 2.60E-02 | 4.99E-01 | 1.00E-02 |  | 2.90E-02 | 7.78E-01 | 1.00E-02 |  | 6.16E-03 |
| childhood BMI | edge_pheno698 | GTExv8.EUR.Brain_Cerebellum | OSBPL7 | 1.96E-02 | 4.54E-01 | 3.00E-02 |  | 4.04E-02 | 7.90E-01 | 2.60E-02 |  | 6.44E-03 |
| childhood BMI | edge_pheno698 | GTExv8.EUR.Brain_Cerebellum | ZDHHC22 | 2.17E-02 | 4.76E-01 | 2.00E-03 |  | 3.98E-02 | 7.90E-01 | 1.00E-03 |  | 6.95E-03 |
| childhood BMI | edge_pheno698 | GTExv8.EUR.Brain_Cerebellum | CELP | 2.65E-02 | 5.01E-01 | 1.00E-02 |  | 3.56E-02 | 7.85E-01 | 5.00E-03 |  | 7.51E-03 |
| childhood BMI | edge_pheno698 | GTExv8.EUR.Brain_Cerebellum | B3GNT4 | 3.20E-02 | 5.15E-01 | 3.00E-03 |  | 3.21E-02 | 7.83E-01 | 1.00E-03 |  | 8.09E-03 |
| childhood BMI | edge_pheno698 | GTExv8.EUR.Brain_Cerebellum | LY75 | 3.13E-02 | 5.15E-01 | 1.00E-03 |  | 3.53E-02 | 7.85E-01 | 2.00E-03 |  | 8.62E-03 |
| childhood BMI | edge_pheno698 | GTExv8.EUR.Brain_Cerebellum | TOR3A | 3.60E-02 | 5.29E-01 | 2.10E-02 |  | 3.41E-02 | 7.85E-01 | 2.30E-02 |  | 9.46E-03 |
| childhood BMI | edge_pheno698 | GTExv8.EUR.Brain_Cerebellum | C8orf31 | 4.60E-02 | 5.66E-01 | 3.30E-02 |  | 2.92E-02 | 7.78E-01 | 1.54E-01 |  | 1.02E-02 |
| childhood BMI | edge_pheno698 | GTExv8.EUR.Brain_Cerebellum | MARS | 3.27E-02 | 5.19E-01 | 1.20E-02 |  | 4.34E-02 | 7.90E-01 | 1.10E-02 |  | 1.07E-02 |
| childhood BMI | edge_pheno698 | GTExv8.EUR.Brain_Cerebellum | RP11-430H10.1 | 4.12E-02 | 5.53E-01 | 2.20E-02 |  | 4.53E-02 | 7.90E-01 | 6.00E-03 |  | 1.36E-02 |
| childhood BMI | edge_pheno698 | GTExv8.EUR.Brain_Cerebellum | IFIT5 | 4.46E-02 | 5.64E-01 | 2.00E-02 |  | 4.23E-02 | 7.90E-01 | 2.40E-02 |  | 1.37E-02 |
| childhood BMI | edge_pheno698 | GTExv8.EUR.Brain_Cerebellum | AP3S2 | 4.93E-02 | 5.71E-01 | 2.20E-02 |  | 4.03E-02 | 7.90E-01 | 2.90E-02 |  | 1.43E-02 |
| adulthood BMI | node_pheno52 | GTExv8.EUR.Brain_Frontal_Cortex_BA9 | UHRF1BP1 | 5.62E-44 | 2.75E-41 | 9.52E-01 |  | 1.12E-02 | 3.52E-01 | 6.20E-02 |  | 6.60E-44 |
| adulthood BMI | node_pheno52 | GTExv8.EUR.Brain_Frontal_Cortex_BA9 | ZNF646 | 2.47E-28 | 4.94E-26 | 8.86E-01 |  | 1.52E-02 | 3.92E-01 | 7.00E-03 |  | 2.58E-28 |
| adulthood BMI | node_pheno52 | GTExv8.EUR.Brain_Frontal_Cortex_BA9 | C6orf106 | 8.61E-27 | 1.51E-24 | 2.00E-03 |  | 6.65E-03 | 2.79E-01 | 1.55E-01 |  | 3.78E-27 |
| adulthood BMI | node_pheno52 | GTExv8.EUR.Brain_Frontal_Cortex_BA9 | FAM86B3P | 2.53E-19 | 2.65E-17 | 6.05E-01 |  | 2.66E-03 | 1.92E-01 | 1.26E-01 |  | 3.35E-20 |
| adulthood BMI | node_pheno52 | GTExv8.EUR.Brain_Frontal_Cortex_BA9 | SMG1P7 | 1.45E-19 | 1.64E-17 | 1.30E-02 |  | 1.34E-02 | 3.82E-01 | 7.10E-02 |  | 9.48E-20 |
| adulthood BMI | node_pheno52 | GTExv8.EUR.Brain_Frontal_Cortex_BA9 | EXOSC6 | 1.51E-19 | 1.66E-17 | 1.20E-02 |  | 1.34E-02 | 3.82E-01 | 7.10E-02 |  | 9.86E-20 |
| adulthood BMI | node_pheno52 | GTExv8.EUR.Brain_Frontal_Cortex_BA9 | ALG1L11P | 9.11E-18 | 8.90E-16 | 9.27E-01 |  | 4.95E-03 | 2.57E-01 | 8.80E-02 |  | 2.05E-18 |
| adulthood BMI | node_pheno52 | GTExv8.EUR.Brain_Frontal_Cortex_BA9 | FAM85B | 2.98E-17 | 2.52E-15 | 8.40E-01 |  | 2.29E-03 | 1.73E-01 | 1.18E-01 |  | 3.08E-18 |
| adulthood BMI | node_pheno52 | GTExv8.EUR.Brain_Frontal_Cortex_BA9 | RP11-196G11.2 | 8.61E-18 | 8.61E-16 | 4.53E-01 |  | 1.96E-02 | 4.28E-01 | 7.00E-03 |  | 7.45E-18 |
| adulthood BMI | node_pheno52 | GTExv8.EUR.Brain_Frontal_Cortex_BA9 | PDXDC2P | 1.16E-17 | 1.11E-15 | 0.00E+00 |  | 1.87E-02 | 4.23E-01 | 2.40E-02 |  | 9.56E-18 |
| adulthood BMI | node_pheno52 | GTExv8.EUR.Brain_Frontal_Cortex_BA9 | SF3B3 | 6.19E-17 | 5.04E-15 | 9.79E-01 |  | 5.90E-03 | 2.73E-01 | 1.70E-02 |  | 1.59E-17 |
| adulthood BMI | node_pheno52 | GTExv8.EUR.Brain_Frontal_Cortex_BA9 | NLRC3 | 1.19E-17 | 1.11E-15 | 0.00E+00 |  | 3.47E-02 | 5.12E-01 | 2.30E-02 |  | 1.79E-17 |
| adulthood BMI | node_pheno52 | GTExv8.EUR.Brain_Frontal_Cortex_BA9 | OR7E161P | 2.22E-17 | 1.95E-15 | 7.00E-02 |  | 1.95E-02 | 4.28E-01 | 6.40E-02 |  | 1.87E-17 |
| adulthood BMI | node_pheno52 | GTExv8.EUR.Brain_Frontal_Cortex_BA9 | AF131215.2 | 9.00E-16 | 6.18E-14 | 6.00E-03 |  | 1.30E-03 | 1.28E-01 | 3.11E-01 |  | 4.94E-17 |
| adulthood BMI | node_pheno52 | GTExv8.EUR.Brain_Frontal_Cortex_BA9 | FAM66A | 5.56E-16 | 3.94E-14 | 9.91E-01 |  | 2.19E-03 | 1.69E-01 | 1.57E-01 |  | 5.14E-17 |
| adulthood BMI | node_pheno52 | GTExv8.EUR.Brain_Frontal_Cortex_BA9 | APOPT1 | 3.98E-17 | 3.30E-15 | 8.88E-01 |  | 3.15E-02 | 5.09E-01 | 3.30E-02 |  | 5.29E-17 |
| adulthood BMI | node_pheno52 | GTExv8.EUR.Brain_Frontal_Cortex_BA9 | AF131216.7 | 6.28E-15 | 3.78E-13 | 6.01E-01 |  | 4.43E-04 | 8.17E-02 | 3.36E-01 |  | 1.15E-16 |
| adulthood BMI | node_pheno52 | GTExv8.EUR.Brain_Frontal_Cortex_BA9 | AC145124.2 | 6.25E-16 | 4.36E-14 | 8.93E-01 |  | 1.12E-02 | 3.52E-01 | 3.60E-02 |  | 2.83E-16 |
| adulthood BMI | node_pheno52 | GTExv8.EUR.Brain_Frontal_Cortex_BA9 | MARK3 | 2.80E-16 | 2.09E-14 | 6.70E-02 |  | 2.75E-02 | 4.80E-01 | 4.00E-03 |  | 3.11E-16 |
| adulthood BMI | node_pheno52 | GTExv8.EUR.Brain_Frontal_Cortex_BA9 | LINC01165 | 1.80E-02 | 6.38E-02 | 1.00E-02 |  | 7.08E-16 | 3.17E-12 | 1.00E-02 |  | 5.09E-16 |
| adulthood BMI | node_pheno52 | GTExv8.EUR.Brain_Frontal_Cortex_BA9 | RP11-351I21.6 | 1.71E-14 | 1.00E-12 | 9.99E-01 |  | 1.91E-03 | 1.58E-01 | 2.81E-01 |  | 1.27E-15 |
| adulthood BMI | node_pheno52 | GTExv8.EUR.Brain_Frontal_Cortex_BA9 | MTMR9 | 7.70E-14 | 4.23E-12 | 4.10E-02 |  | 4.75E-04 | 8.17E-02 | 8.40E-02 |  | 1.42E-15 |
| adulthood BMI | node_pheno52 | GTExv8.EUR.Brain_Frontal_Cortex_BA9 | RP11-481A20.10 | 4.30E-15 | 2.66E-13 | 0.00E+00 |  | 2.15E-02 | 4.30E-01 | 4.70E-02 |  | 3.50E-15 |
| adulthood BMI | node_pheno52 | GTExv8.EUR.Brain_Frontal_Cortex_BA9 | AF131215.9 | 1.71E-13 | 8.74E-12 | 8.82E-01 |  | 6.00E-04 | 8.66E-02 | 2.87E-01 |  | 3.88E-15 |
| adulthood BMI | node_pheno52 | GTExv8.EUR.Brain_Frontal_Cortex_BA9 | TDH | 2.25E-13 | 1.12E-11 | 1.70E-01 |  | 1.32E-03 | 1.28E-01 | 2.59E-01 |  | 1.09E-14 |
| adulthood BMI | node_pheno52 | GTExv8.EUR.Brain_Frontal_Cortex_BA9 | RPL10P19 | 1.45E-13 | 7.50E-12 | 3.63E-01 |  | 3.51E-03 | 2.13E-01 | 1.19E-01 |  | 1.84E-14 |
| adulthood BMI | node_pheno52 | GTExv8.EUR.Brain_Frontal_Cortex_BA9 | FAM167A | 1.98E-13 | 1.00E-11 | 6.31E-01 |  | 6.42E-03 | 2.77E-01 | 1.58E-01 |  | 4.49E-14 |
| adulthood BMI | node_pheno52 | GTExv8.EUR.Brain_Frontal_Cortex_BA9 | PCDH17 | 1.10E-11 | 4.36E-10 | 1.60E-01 |  | 2.89E-04 | 8.17E-02 | 3.40E-02 |  | 1.09E-13 |
| adulthood BMI | node_pheno52 | GTExv8.EUR.Brain_Frontal_Cortex_BA9 | ASB16-AS1 | 6.12E-13 | 2.93E-11 | 9.40E-01 |  | 8.92E-03 | 3.33E-01 | 1.20E-02 |  | 1.85E-13 |
| adulthood BMI | node_pheno52 | GTExv8.EUR.Brain_Frontal_Cortex_BA9 | DPYSL4 | 6.85E-07 | 1.17E-05 | 8.05E-01 |  | 2.01E-08 | 4.50E-05 | 4.50E-02 |  | 4.53E-13 |
| adulthood BMI | node_pheno52 | GTExv8.EUR.Brain_Frontal_Cortex_BA9 | NEIL2 | 6.47E-13 | 3.06E-11 | 1.74E-01 |  | 3.53E-02 | 5.12E-01 | 5.80E-02 |  | 7.39E-13 |
| adulthood BMI | node_pheno52 | GTExv8.EUR.Brain_Frontal_Cortex_BA9 | SNX19 | 7.74E-12 | 3.12E-10 | 1.00E-02 |  | 4.20E-03 | 2.29E-01 | 1.52E-01 |  | 1.04E-12 |
| adulthood BMI | node_pheno52 | GTExv8.EUR.Brain_Frontal_Cortex_BA9 | AF131216.5 | 2.20E-11 | 8.06E-10 | 0.00E+00 |  | 3.73E-03 | 2.13E-01 | 1.65E-01 |  | 2.55E-12 |
| adulthood BMI | node_pheno52 | GTExv8.EUR.Brain_Frontal_Cortex_BA9 | CHMP3 | 6.76E-12 | 2.78E-10 | 9.76E-01 |  | 1.58E-02 | 3.96E-01 | 1.40E-02 |  | 3.30E-12 |
| adulthood BMI | node_pheno52 | GTExv8.EUR.Brain_Frontal_Cortex_BA9 | GTF2IRD2 | 3.70E-12 | 1.61E-10 | 3.30E-02 |  | 3.33E-02 | 5.11E-01 | 3.40E-02 |  | 3.79E-12 |
| adulthood BMI | node_pheno52 | GTExv8.EUR.Brain_Frontal_Cortex_BA9 | ECE2 | 2.91E-12 | 1.28E-10 | 9.93E-01 |  | 4.81E-02 | 5.71E-01 | 2.50E-02 |  | 4.28E-12 |
| adulthood BMI | node_pheno52 | GTExv8.EUR.Brain_Frontal_Cortex_BA9 | FAM90A25P | 3.20E-10 | 1.01E-08 | 6.03E-01 |  | 3.76E-03 | 2.13E-01 | 3.00E-03 |  | 3.42E-11 |
| adulthood BMI | node_pheno52 | GTExv8.EUR.Brain_Frontal_Cortex_BA9 | KRT18P34 | 1.37E-10 | 4.53E-09 | 9.26E-01 |  | 3.52E-02 | 5.12E-01 | 2.60E-02 |  | 1.31E-10 |
| adulthood BMI | node_pheno52 | GTExv8.EUR.Brain_Frontal_Cortex_BA9 | VCL | 8.70E-10 | 2.59E-08 | 9.16E-01 |  | 4.77E-02 | 5.71E-01 | 1.40E-02 |  | 1.03E-09 |
| adulthood BMI | node_pheno52 | GTExv8.EUR.Brain_Frontal_Cortex_BA9 | LINC00599 | 3.19E-09 | 9.05E-08 | 4.72E-01 |  | 3.85E-02 | 5.25E-01 | 1.01E-01 |  | 2.92E-09 |
| adulthood BMI | node_pheno52 | GTExv8.EUR.Brain_Frontal_Cortex_BA9 | BID | 6.54E-08 | 1.38E-06 | 9.73E-01 |  | 1.99E-03 | 1.59E-01 | 2.81E-01 |  | 3.10E-09 |
| adulthood BMI | node_pheno52 | GTExv8.EUR.Brain_Frontal_Cortex_BA9 | SGCB | 6.30E-09 | 1.66E-07 | 6.30E-01 |  | 3.13E-02 | 5.09E-01 | 6.00E-03 |  | 4.61E-09 |
| adulthood BMI | node_pheno52 | GTExv8.EUR.Brain_Frontal_Cortex_BA9 | CCDC57 | 1.47E-08 | 3.67E-07 | 9.19E-01 |  | 1.44E-02 | 3.88E-01 | 6.00E-02 |  | 4.93E-09 |
| adulthood BMI | node_pheno52 | GTExv8.EUR.Brain_Frontal_Cortex_BA9 | SRR | 2.93E-08 | 6.64E-07 | 0.00E+00 |  | 8.88E-03 | 3.33E-01 | 5.70E-02 |  | 6.00E-09 |
| adulthood BMI | node_pheno52 | GTExv8.EUR.Brain_Frontal_Cortex_BA9 | CTD-2284J15.1 | 4.71E-08 | 1.03E-06 | 8.86E-01 |  | 6.45E-03 | 2.77E-01 | 1.50E-02 |  | 6.96E-09 |
| adulthood BMI | node_pheno52 | GTExv8.EUR.Brain_Frontal_Cortex_BA9 | CANX | 3.91E-06 | 5.77E-05 | 9.56E-01 |  | 1.82E-04 | 8.17E-02 | 6.71E-01 |  | 1.57E-08 |
| adulthood BMI | node_pheno52 | GTExv8.EUR.Brain_Frontal_Cortex_BA9 | SGMS1-AS1 | 5.30E-06 | 7.57E-05 | 8.11E-01 |  | 4.34E-04 | 8.17E-02 | 3.81E-01 |  | 4.81E-08 |
| adulthood BMI | node_pheno52 | GTExv8.EUR.Brain_Frontal_Cortex_BA9 | RP11-140A10.3 | 2.70E-02 | 8.74E-02 | 1.90E-02 |  | 9.42E-08 | 1.40E-04 | 1.90E-02 |  | 5.29E-08 |
| adulthood BMI | node_pheno52 | GTExv8.EUR.Brain_Frontal_Cortex_BA9 | RAB15 | 4.15E-07 | 7.33E-06 | 1.07E-01 |  | 8.94E-03 | 3.33E-01 | 1.00E-03 |  | 7.57E-08 |
| adulthood BMI | node_pheno52 | GTExv8.EUR.Brain_Frontal_Cortex_BA9 | DEFB109P3 | 1.30E-07 | 2.54E-06 | 4.30E-02 |  | 3.36E-02 | 5.11E-01 | 2.00E-03 |  | 8.85E-08 |
| adulthood BMI | node_pheno52 | GTExv8.EUR.Brain_Frontal_Cortex_BA9 | UBE2U | 2.39E-06 | 3.70E-05 | 9.60E-01 |  | 3.08E-03 | 2.12E-01 | 1.95E-01 |  | 1.45E-07 |
| adulthood BMI | node_pheno52 | GTExv8.EUR.Brain_Frontal_Cortex_BA9 | PABPC1L | 1.91E-07 | 3.59E-06 | 9.78E-01 |  | 4.83E-02 | 5.71E-01 | 2.40E-02 |  | 1.80E-07 |
| adulthood BMI | node_pheno52 | GTExv8.EUR.Brain_Frontal_Cortex_BA9 | MRPL50 | 2.66E-07 | 4.81E-06 | 3.77E-01 |  | 3.58E-02 | 5.12E-01 | 1.00E-03 |  | 1.86E-07 |
| adulthood BMI | node_pheno52 | GTExv8.EUR.Brain_Frontal_Cortex_BA9 | ENPP7P4 | 1.10E-06 | 1.83E-05 | 4.29E-01 |  | 1.05E-02 | 3.44E-01 | 2.00E-03 |  | 2.23E-07 |
| adulthood BMI | node_pheno52 | GTExv8.EUR.Brain_Frontal_Cortex_BA9 | DESI1 | 2.39E-07 | 4.40E-06 | 2.14E-01 |  | 4.89E-02 | 5.71E-01 | 3.00E-03 |  | 2.25E-07 |
| adulthood BMI | node_pheno52 | GTExv8.EUR.Brain_Frontal_Cortex_BA9 | THBS3 | 9.50E-06 | 1.24E-04 | 4.00E-02 |  | 1.35E-03 | 1.28E-01 | 6.10E-02 |  | 2.45E-07 |
| adulthood BMI | node_pheno52 | GTExv8.EUR.Brain_Frontal_Cortex_BA9 | RAC3 | 5.52E-07 | 9.56E-06 | 3.50E-02 |  | 2.64E-02 | 4.65E-01 | 4.00E-03 |  | 2.78E-07 |
| adulthood BMI | node_pheno52 | GTExv8.EUR.Brain_Frontal_Cortex_BA9 | NTAN1P2 | 3.26E-05 | 3.64E-04 | 7.76E-01 |  | 4.61E-04 | 8.17E-02 | 2.90E-02 |  | 2.86E-07 |
| adulthood BMI | node_pheno52 | GTExv8.EUR.Brain_Frontal_Cortex_BA9 | RMND1 | 1.50E-06 | 2.42E-05 | 6.47E-01 |  | 1.90E-02 | 4.23E-01 | 2.00E-03 |  | 5.23E-07 |
| adulthood BMI | node_pheno52 | GTExv8.EUR.Brain_Frontal_Cortex_BA9 | PPL | 1.32E-04 | 1.16E-03 | 3.34E-01 |  | 5.98E-04 | 8.66E-02 | 7.40E-02 |  | 1.37E-06 |
| adulthood BMI | node_pheno52 | GTExv8.EUR.Brain_Frontal_Cortex_BA9 | METTL21A | 3.81E-06 | 5.68E-05 | 2.10E-02 |  | 2.17E-02 | 4.30E-01 | 3.50E-02 |  | 1.43E-06 |
| adulthood BMI | node_pheno52 | GTExv8.EUR.Brain_Frontal_Cortex_BA9 | HLTF | 9.60E-06 | 1.25E-04 | 7.29E-01 |  | 1.10E-02 | 3.51E-01 | 8.30E-02 |  | 1.80E-06 |
| adulthood BMI | node_pheno52 | GTExv8.EUR.Brain_Frontal_Cortex_BA9 | FAM114A2 | 7.44E-06 | 1.02E-04 | 3.10E-02 |  | 2.46E-02 | 4.45E-01 | 7.00E-03 |  | 3.02E-06 |
| adulthood BMI | node_pheno52 | GTExv8.EUR.Brain_Frontal_Cortex_BA9 | JAKMIP3 | 6.10E-05 | 6.18E-04 | 0.00E+00 |  | 4.29E-03 | 2.30E-01 | 0.00E+00 |  | 4.23E-06 |
| adulthood BMI | node_pheno52 | GTExv8.EUR.Brain_Frontal_Cortex_BA9 | TUBA1C | 6.11E-06 | 8.56E-05 | 7.90E-02 |  | 4.42E-02 | 5.59E-01 | 2.40E-02 |  | 4.35E-06 |
| adulthood BMI | node_pheno52 | GTExv8.EUR.Brain_Frontal_Cortex_BA9 | CTDNEP1 | 1.30E-05 | 1.61E-04 | 8.09E-01 |  | 2.12E-02 | 4.30E-01 | 2.90E-02 |  | 4.44E-06 |
| adulthood BMI | node_pheno52 | GTExv8.EUR.Brain_Frontal_Cortex_BA9 | RAPGEFL1 | 4.20E-05 | 4.53E-04 | 8.11E-01 |  | 7.62E-03 | 3.02E-01 | 5.00E-03 |  | 5.11E-06 |
| adulthood BMI | node_pheno52 | GTExv8.EUR.Brain_Frontal_Cortex_BA9 | RP11-10L7.1 | 4.55E-05 | 4.85E-04 | 1.40E-02 |  | 7.40E-03 | 2.98E-01 | 1.28E-01 |  | 5.35E-06 |
| adulthood BMI | node_pheno52 | GTExv8.EUR.Brain_Frontal_Cortex_BA9 | AC010884.1 | 2.90E-05 | 3.29E-04 | 1.30E-02 |  | 1.74E-02 | 4.14E-01 | 4.20E-02 |  | 7.80E-06 |
| adulthood BMI | node_pheno52 | GTExv8.EUR.Brain_Frontal_Cortex_BA9 | RAD52 | 1.71E-05 | 2.06E-04 | 8.00E-03 |  | 3.15E-02 | 5.09E-01 | 2.50E-02 |  | 8.32E-06 |
| adulthood BMI | node_pheno52 | GTExv8.EUR.Brain_Frontal_Cortex_BA9 | AC011330.5 | 1.29E-05 | 1.61E-04 | 9.20E-02 |  | 4.51E-02 | 5.62E-01 | 2.90E-02 |  | 8.93E-06 |
| adulthood BMI | node_pheno52 | GTExv8.EUR.Brain_Frontal_Cortex_BA9 | TARS2 | 8.50E-04 | 5.40E-03 | 1.10E-02 |  | 8.13E-04 | 1.10E-01 | 4.39E-01 |  | 1.05E-05 |
| adulthood BMI | node_pheno52 | GTExv8.EUR.Brain_Frontal_Cortex_BA9 | FNDC9 | 1.80E-02 | 6.38E-02 | 0.00E+00 |  | 5.41E-05 | 3.46E-02 | 3.00E-03 |  | 1.45E-05 |
| adulthood BMI | node_pheno52 | GTExv8.EUR.Brain_Frontal_Cortex_BA9 | PDXDC1 | 2.74E-05 | 3.13E-04 | 9.48E-01 |  | 3.75E-02 | 5.24E-01 | 4.00E-03 |  | 1.52E-05 |
| adulthood BMI | node_pheno52 | GTExv8.EUR.Brain_Frontal_Cortex_BA9 | GRK4 | 2.43E-05 | 2.81E-04 | 2.10E-02 |  | 4.80E-02 | 5.71E-01 | 1.10E-02 |  | 1.71E-05 |
| adulthood BMI | node_pheno52 | GTExv8.EUR.Brain_Frontal_Cortex_BA9 | MRPS9 | 3.62E-04 | 2.70E-03 | 2.10E-02 |  | 4.32E-03 | 2.30E-01 | 7.00E-02 |  | 2.25E-05 |
| adulthood BMI | node_pheno52 | GTExv8.EUR.Brain_Frontal_Cortex_BA9 | RP11-304L19.13 | 1.69E-03 | 9.50E-03 | 0.00E+00 |  | 1.23E-03 | 1.28E-01 | 2.79E-01 |  | 2.92E-05 |
| adulthood BMI | node_pheno52 | GTExv8.EUR.Brain_Frontal_Cortex_BA9 | PSMD13 | 5.32E-05 | 5.52E-04 | 4.84E-01 |  | 4.85E-02 | 5.71E-01 | 6.00E-03 |  | 3.58E-05 |
| adulthood BMI | node_pheno52 | GTExv8.EUR.Brain_Frontal_Cortex_BA9 | RIC8A | 6.33E-05 | 6.30E-04 | 4.30E-01 |  | 4.11E-02 | 5.41E-01 | 7.00E-03 |  | 3.61E-05 |
| adulthood BMI | node_pheno52 | GTExv8.EUR.Brain_Frontal_Cortex_BA9 | FBXO42 | 1.22E-04 | 1.09E-03 | 5.22E-01 |  | 2.14E-02 | 4.30E-01 | 4.50E-02 |  | 3.62E-05 |
| adulthood BMI | node_pheno52 | GTExv8.EUR.Brain_Frontal_Cortex_BA9 | TOM1L2 | 3.00E-04 | 2.31E-03 | 3.49E-01 |  | 9.03E-03 | 3.33E-01 | 7.60E-02 |  | 3.74E-05 |
| adulthood BMI | node_pheno52 | GTExv8.EUR.Brain_Frontal_Cortex_BA9 | ZNF266 | 2.70E-04 | 2.10E-03 | 3.25E-01 |  | 1.15E-02 | 3.54E-01 | 8.00E-02 |  | 4.26E-05 |
| adulthood BMI | node_pheno52 | GTExv8.EUR.Brain_Frontal_Cortex_BA9 | LACTB | 7.80E-03 | 3.32E-02 | 0.00E+00 |  | 4.20E-04 | 8.17E-02 | 6.35E-01 |  | 4.46E-05 |
| adulthood BMI | node_pheno52 | GTExv8.EUR.Brain_Frontal_Cortex_BA9 | METTL10 | 2.53E-04 | 2.00E-03 | 0.00E+00 |  | 1.67E-02 | 4.10E-01 | 3.20E-02 |  | 5.65E-05 |
| adulthood BMI | node_pheno52 | GTExv8.EUR.Brain_Frontal_Cortex_BA9 | RAB40C | 3.47E-03 | 1.74E-02 | 1.30E-02 |  | 1.43E-03 | 1.33E-01 | 1.63E-01 |  | 6.54E-05 |
| adulthood BMI | node_pheno52 | GTExv8.EUR.Brain_Frontal_Cortex_BA9 | RUSC1-AS1 | 1.02E-04 | 9.35E-04 | 1.85E-01 |  | 4.85E-02 | 5.71E-01 | 1.01E-01 |  | 6.54E-05 |
| adulthood BMI | node_pheno52 | GTExv8.EUR.Brain_Frontal_Cortex_BA9 | PTOV1-AS1 | 3.93E-03 | 1.94E-02 | 2.00E-02 |  | 1.28E-03 | 1.28E-01 | 2.60E-02 |  | 6.64E-05 |
| adulthood BMI | node_pheno52 | GTExv8.EUR.Brain_Frontal_Cortex_BA9 | C6orf201 | 3.22E-04 | 2.45E-03 | 1.94E-01 |  | 2.21E-02 | 4.30E-01 | 7.00E-03 |  | 9.15E-05 |
| adulthood BMI | node_pheno52 | GTExv8.EUR.Brain_Frontal_Cortex_BA9 | PNMAL2 | 4.48E-04 | 3.19E-03 | 2.17E-01 |  | 2.10E-02 | 4.30E-01 | 1.80E-02 |  | 1.18E-04 |
| adulthood BMI | node_pheno52 | GTExv8.EUR.Brain_Frontal_Cortex_BA9 | NME2 | 1.17E-03 | 6.99E-03 | 1.34E-01 |  | 8.96E-03 | 3.33E-01 | 9.80E-02 |  | 1.31E-04 |
| adulthood BMI | node_pheno52 | GTExv8.EUR.Brain_Frontal_Cortex_BA9 | WBP11 | 2.69E-04 | 2.10E-03 | 5.23E-01 |  | 4.43E-02 | 5.59E-01 | 5.00E-03 |  | 1.47E-04 |
| adulthood BMI | node_pheno52 | GTExv8.EUR.Brain_Frontal_Cortex_BA9 | CRHR1 | 4.77E-02 | 1.33E-01 | 1.00E-03 |  | 2.52E-04 | 8.17E-02 | 8.60E-02 |  | 1.48E-04 |
| adulthood BMI | node_pheno52 | GTExv8.EUR.Brain_Frontal_Cortex_BA9 | IRAK1BP1 | 4.20E-04 | 3.03E-03 | 1.89E-01 |  | 3.17E-02 | 5.09E-01 | 3.10E-02 |  | 1.63E-04 |
| adulthood BMI | node_pheno52 | GTExv8.EUR.Brain_Frontal_Cortex_BA9 | RFPL1S | 7.36E-04 | 4.82E-03 | 2.24E-01 |  | 2.03E-02 | 4.30E-01 | 4.90E-02 |  | 1.81E-04 |
| adulthood BMI | node_pheno52 | GTExv8.EUR.Brain_Frontal_Cortex_BA9 | RP11-946P6.6 | 4.30E-04 | 3.09E-03 | 4.40E-02 |  | 3.56E-02 | 5.12E-01 | 0.00E+00 |  | 1.85E-04 |
| adulthood BMI | node_pheno52 | GTExv8.EUR.Brain_Frontal_Cortex_BA9 | ENDOG | 5.04E-03 | 2.37E-02 | 1.10E-02 |  | 3.76E-03 | 2.13E-01 | 3.38E-01 |  | 2.25E-04 |
| adulthood BMI | node_pheno52 | GTExv8.EUR.Brain_Frontal_Cortex_BA9 | NICN1 | 8.53E-04 | 5.41E-03 | 4.00E-02 |  | 2.52E-02 | 4.50E-01 | 6.00E-03 |  | 2.52E-04 |
| adulthood BMI | node_pheno52 | GTExv8.EUR.Brain_Frontal_Cortex_BA9 | RNPS1 | 2.34E-03 | 1.26E-02 | 2.00E-03 |  | 9.96E-03 | 3.43E-01 | 6.10E-02 |  | 2.72E-04 |
| adulthood BMI | node_pheno52 | GTExv8.EUR.Brain_Frontal_Cortex_BA9 | AC006538.1 | 9.34E-04 | 5.83E-03 | 3.90E-02 |  | 2.59E-02 | 4.59E-01 | 2.10E-02 |  | 2.81E-04 |
| adulthood BMI | node_pheno52 | GTExv8.EUR.Brain_Frontal_Cortex_BA9 | RP11-890B15.3 | 7.20E-04 | 4.75E-03 | 0.00E+00 |  | 3.44E-02 | 5.12E-01 | 3.30E-02 |  | 2.87E-04 |
| adulthood BMI | node_pheno52 | GTExv8.EUR.Brain_Frontal_Cortex_BA9 | CENPBD1P1 | 6.90E-03 | 2.98E-02 | 3.00E-02 |  | 5.60E-03 | 2.69E-01 | 1.44E-01 |  | 4.31E-04 |
| adulthood BMI | node_pheno52 | GTExv8.EUR.Brain_Frontal_Cortex_BA9 | WFIKKN1 | 3.17E-03 | 1.62E-02 | 0.00E+00 |  | 1.33E-02 | 3.82E-01 | 4.20E-02 |  | 4.68E-04 |
| adulthood BMI | node_pheno52 | GTExv8.EUR.Brain_Frontal_Cortex_BA9 | MAN2C1 | 1.36E-03 | 7.96E-03 | 0.00E+00 |  | 3.20E-02 | 5.09E-01 | 1.20E-02 |  | 4.80E-04 |
| adulthood BMI | node_pheno52 | GTExv8.EUR.Brain_Frontal_Cortex_BA9 | PIGQ | 1.10E-02 | 4.34E-02 | 0.00E+00 |  | 4.62E-03 | 2.43E-01 | 1.64E-01 |  | 5.54E-04 |
| adulthood BMI | node_pheno52 | GTExv8.EUR.Brain_Frontal_Cortex_BA9 | CTB-171A8.1 | 3.44E-03 | 1.72E-02 | 1.70E-02 |  | 1.73E-02 | 4.14E-01 | 1.30E-02 |  | 6.37E-04 |
| adulthood BMI | node_pheno52 | GTExv8.EUR.Brain_Frontal_Cortex_BA9 | MIR124-2HG | 3.81E-03 | 1.88E-02 | 5.89E-01 |  | 1.69E-02 | 4.11E-01 | 2.00E-03 |  | 6.86E-04 |
| adulthood BMI | node_pheno52 | GTExv8.EUR.Brain_Frontal_Cortex_BA9 | RANBP10 | 1.23E-02 | 4.75E-02 | 2.00E-03 |  | 5.98E-03 | 2.73E-01 | 9.70E-02 |  | 7.74E-04 |
| adulthood BMI | node_pheno52 | GTExv8.EUR.Brain_Frontal_Cortex_BA9 | APIP | 2.98E-03 | 1.53E-02 | 8.00E-03 |  | 2.93E-02 | 4.93E-01 | 3.30E-02 |  | 9.03E-04 |
| adulthood BMI | node_pheno52 | GTExv8.EUR.Brain_Frontal_Cortex_BA9 | RP11-418J17.1 | 4.80E-03 | 2.27E-02 | 0.00E+00 |  | 1.88E-02 | 4.23E-01 | 5.40E-02 |  | 9.32E-04 |
| adulthood BMI | node_pheno52 | GTExv8.EUR.Brain_Frontal_Cortex_BA9 | CCDC116 | 1.68E-02 | 6.08E-02 | 4.00E-02 |  | 5.57E-03 | 2.69E-01 | 7.50E-02 |  | 9.62E-04 |
| adulthood BMI | node_pheno52 | GTExv8.EUR.Brain_Frontal_Cortex_BA9 | DGKQ | 2.35E-03 | 1.26E-02 | 2.30E-02 |  | 4.05E-02 | 5.39E-01 | 5.00E-03 |  | 9.76E-04 |
| adulthood BMI | node_pheno52 | GTExv8.EUR.Brain_Frontal_Cortex_BA9 | ZFAND4 | 2.06E-03 | 1.13E-02 | 9.00E-03 |  | 4.72E-02 | 5.71E-01 | 4.00E-03 |  | 9.96E-04 |
| adulthood BMI | node_pheno52 | GTExv8.EUR.Brain_Frontal_Cortex_BA9 | KDM7A | 5.81E-03 | 2.62E-02 | 4.39E-01 |  | 2.03E-02 | 4.30E-01 | 8.00E-03 |  | 1.18E-03 |
| adulthood BMI | node_pheno52 | GTExv8.EUR.Brain_Frontal_Cortex_BA9 | PSEN2 | 3.00E-02 | 9.40E-02 | 1.00E-03 |  | 4.19E-03 | 2.29E-01 | 2.60E-02 |  | 1.25E-03 |
| adulthood BMI | node_pheno52 | GTExv8.EUR.Brain_Frontal_Cortex_BA9 | CA3-AS1 | 5.80E-03 | 2.61E-02 | 2.00E-03 |  | 2.25E-02 | 4.32E-01 | 3.00E-03 |  | 1.30E-03 |
| adulthood BMI | node_pheno52 | GTExv8.EUR.Brain_Frontal_Cortex_BA9 | NOTUM | 3.20E-03 | 1.62E-02 | 2.86E-01 |  | 4.47E-02 | 5.62E-01 | 1.00E-03 |  | 1.41E-03 |
| adulthood BMI | node_pheno52 | GTExv8.EUR.Brain_Frontal_Cortex_BA9 | RP11-424M24.3 | 2.40E-02 | 7.95E-02 | 4.80E-02 |  | 6.11E-03 | 2.73E-01 | 1.00E-03 |  | 1.44E-03 |
| adulthood BMI | node_pheno52 | GTExv8.EUR.Brain_Frontal_Cortex_BA9 | SEL1L3 | 1.79E-02 | 6.37E-02 | 4.10E-02 |  | 8.96E-03 | 3.33E-01 | 3.00E-03 |  | 1.56E-03 |
| adulthood BMI | node_pheno52 | GTExv8.EUR.Brain_Frontal_Cortex_BA9 | SIRPB1 | 4.80E-03 | 2.27E-02 | 1.00E-03 |  | 3.56E-02 | 5.12E-01 | 3.20E-02 |  | 1.65E-03 |
| adulthood BMI | node_pheno52 | GTExv8.EUR.Brain_Frontal_Cortex_BA9 | C4orf36 | 3.80E-03 | 1.88E-02 | 3.00E-03 |  | 4.50E-02 | 5.62E-01 | 1.00E-03 |  | 1.65E-03 |
| adulthood BMI | node_pheno52 | GTExv8.EUR.Brain_Frontal_Cortex_BA9 | AC115522.3 | 5.00E-03 | 2.35E-02 | 3.70E-02 |  | 3.47E-02 | 5.12E-01 | 1.90E-02 |  | 1.67E-03 |
| adulthood BMI | node_pheno52 | GTExv8.EUR.Brain_Frontal_Cortex_BA9 | C16orf62 | 5.40E-03 | 2.48E-02 | 2.00E-03 |  | 3.27E-02 | 5.11E-01 | 3.30E-02 |  | 1.70E-03 |
| adulthood BMI | node_pheno52 | GTExv8.EUR.Brain_Frontal_Cortex_BA9 | RP4-561L24.3 | 6.46E-03 | 2.85E-02 | 3.00E-03 |  | 2.76E-02 | 4.81E-01 | 1.00E-03 |  | 1.72E-03 |
| adulthood BMI | node_pheno52 | GTExv8.EUR.Brain_Frontal_Cortex_BA9 | ZNF584 | 1.78E-02 | 6.34E-02 | 1.20E-02 |  | 1.14E-02 | 3.53E-01 | 7.10E-02 |  | 1.93E-03 |
| adulthood BMI | node_pheno52 | GTExv8.EUR.Brain_Frontal_Cortex_BA9 | BIN3 | 7.42E-03 | 3.17E-02 | 6.00E-03 |  | 2.83E-02 | 4.88E-01 | 1.70E-02 |  | 1.99E-03 |
| adulthood BMI | node_pheno52 | GTExv8.EUR.Brain_Frontal_Cortex_BA9 | DPH1 | 7.97E-03 | 3.36E-02 | 3.30E-02 |  | 2.89E-02 | 4.93E-01 | 3.80E-02 |  | 2.16E-03 |
| adulthood BMI | node_pheno52 | GTExv8.EUR.Brain_Frontal_Cortex_BA9 | DIDO1 | 3.64E-02 | 1.08E-01 | 4.40E-02 |  | 6.76E-03 | 2.80E-01 | 1.21E-01 |  | 2.29E-03 |
| adulthood BMI | node_pheno52 | GTExv8.EUR.Brain_Frontal_Cortex_BA9 | VPS9D1 | 1.55E-02 | 5.70E-02 | 6.00E-03 |  | 1.71E-02 | 4.13E-01 | 5.00E-02 |  | 2.45E-03 |
| adulthood BMI | node_pheno52 | GTExv8.EUR.Brain_Frontal_Cortex_BA9 | LYPD5 | 5.60E-03 | 2.54E-02 | 1.80E-02 |  | 4.80E-02 | 5.71E-01 | 1.80E-02 |  | 2.48E-03 |
| adulthood BMI | node_pheno52 | GTExv8.EUR.Brain_Frontal_Cortex_BA9 | ATPIF1 | 8.70E-03 | 3.62E-02 | 2.40E-02 |  | 3.26E-02 | 5.11E-01 | 3.40E-02 |  | 2.60E-03 |
| adulthood BMI | node_pheno52 | GTExv8.EUR.Brain_Frontal_Cortex_BA9 | TRMT61A | 1.68E-02 | 6.08E-02 | 3.62E-01 |  | 2.00E-02 | 4.30E-01 | 1.90E-02 |  | 3.02E-03 |
| adulthood BMI | node_pheno52 | GTExv8.EUR.Brain_Frontal_Cortex_BA9 | STAG3L4 | 2.79E-02 | 8.97E-02 | 3.00E-03 |  | 1.30E-02 | 3.78E-01 | 4.10E-02 |  | 3.23E-03 |
| adulthood BMI | node_pheno52 | GTExv8.EUR.Brain_Frontal_Cortex_BA9 | RP11-109A6.5 | 1.10E-02 | 4.34E-02 | 1.20E-02 |  | 3.82E-02 | 5.24E-01 | 1.30E-02 |  | 3.69E-03 |
| adulthood BMI | node_pheno52 | GTExv8.EUR.Brain_Frontal_Cortex_BA9 | MBLAC1 | 4.62E-02 | 1.30E-01 | 8.00E-03 |  | 1.01E-02 | 3.43E-01 | 4.70E-02 |  | 4.06E-03 |
| adulthood BMI | node_pheno52 | GTExv8.EUR.Brain_Frontal_Cortex_BA9 | COG4 | 1.44E-02 | 5.39E-02 | 1.50E-02 |  | 3.34E-02 | 5.11E-01 | 9.00E-03 |  | 4.16E-03 |
| adulthood BMI | node_pheno52 | GTExv8.EUR.Brain_Frontal_Cortex_BA9 | RP5-1092A3.4 | 1.16E-02 | 4.54E-02 | 2.70E-02 |  | 4.32E-02 | 5.59E-01 | 4.00E-02 |  | 4.31E-03 |
| adulthood BMI | node_pheno52 | GTExv8.EUR.Brain_Frontal_Cortex_BA9 | CCDC71 | 1.73E-02 | 6.21E-02 | 2.00E-03 |  | 2.90E-02 | 4.93E-01 | 2.80E-02 |  | 4.31E-03 |
| adulthood BMI | node_pheno52 | GTExv8.EUR.Brain_Frontal_Cortex_BA9 | RP11-166O4.6 | 2.96E-02 | 9.31E-02 | 3.00E-03 |  | 1.88E-02 | 4.23E-01 | 3.50E-02 |  | 4.72E-03 |
| adulthood BMI | node_pheno52 | GTExv8.EUR.Brain_Frontal_Cortex_BA9 | RP11-445H22.3 | 1.50E-02 | 5.56E-02 | 2.30E-02 |  | 3.74E-02 | 5.24E-01 | 1.40E-02 |  | 4.76E-03 |
| adulthood BMI | node_pheno52 | GTExv8.EUR.Brain_Frontal_Cortex_BA9 | ZNF273 | 4.43E-02 | 1.25E-01 | 3.00E-03 |  | 1.30E-02 | 3.78E-01 | 5.00E-03 |  | 4.86E-03 |
| adulthood BMI | node_pheno52 | GTExv8.EUR.Brain_Frontal_Cortex_BA9 | SERHL | 2.80E-02 | 8.98E-02 | 1.80E-02 |  | 2.07E-02 | 4.30E-01 | 3.00E-02 |  | 4.90E-03 |
| adulthood BMI | node_pheno52 | GTExv8.EUR.Brain_Frontal_Cortex_BA9 | ZNF443 | 2.71E-02 | 8.76E-02 | 4.90E-02 |  | 2.18E-02 | 4.30E-01 | 3.00E-03 |  | 4.98E-03 |
| adulthood BMI | node_pheno52 | GTExv8.EUR.Brain_Frontal_Cortex_BA9 | RP11-368I7.6 | 1.58E-02 | 5.80E-02 | 8.00E-02 |  | 3.76E-02 | 5.24E-01 | 5.20E-02 |  | 5.01E-03 |
| adulthood BMI | node_pheno52 | GTExv8.EUR.Brain_Frontal_Cortex_BA9 | PDLIM2 | 1.71E-02 | 6.14E-02 | 1.40E-02 |  | 3.77E-02 | 5.24E-01 | 3.50E-02 |  | 5.39E-03 |
| adulthood BMI | node_pheno52 | GTExv8.EUR.Brain_Frontal_Cortex_BA9 | MYRFL | 3.75E-02 | 1.11E-01 | 0.00E+00 |  | 1.88E-02 | 4.23E-01 | 9.30E-02 |  | 5.82E-03 |
| adulthood BMI | node_pheno52 | GTExv8.EUR.Brain_Frontal_Cortex_BA9 | RP11-522I20.3 | 3.93E-02 | 1.15E-01 | 7.20E-02 |  | 1.85E-02 | 4.23E-01 | 1.60E-02 |  | 5.97E-03 |
| adulthood BMI | node_pheno52 | GTExv8.EUR.Brain_Frontal_Cortex_BA9 | PRKRIP1 | 4.42E-02 | 1.25E-01 | 0.00E+00 |  | 1.65E-02 | 4.08E-01 | 4.00E-03 |  | 6.01E-03 |
| adulthood BMI | node_pheno52 | GTExv8.EUR.Brain_Frontal_Cortex_BA9 | CR2 | 2.93E-02 | 9.26E-02 | 4.10E-02 |  | 2.51E-02 | 4.50E-01 | 4.00E-03 |  | 6.04E-03 |
| adulthood BMI | node_pheno52 | GTExv8.EUR.Brain_Frontal_Cortex_BA9 | SMIM8 | 4.38E-02 | 1.25E-01 | 0.00E+00 |  | 1.81E-02 | 4.23E-01 | 1.80E-02 |  | 6.45E-03 |
| adulthood BMI | node_pheno52 | GTExv8.EUR.Brain_Frontal_Cortex_BA9 | ARV1 | 2.23E-02 | 7.52E-02 | 1.77E-01 |  | 3.64E-02 | 5.17E-01 | 5.30E-02 |  | 6.59E-03 |
| adulthood BMI | node_pheno52 | GTExv8.EUR.Brain_Frontal_Cortex_BA9 | SPATA33 | 3.51E-02 | 1.05E-01 | 3.50E-02 |  | 2.44E-02 | 4.45E-01 | 3.80E-02 |  | 6.91E-03 |
| adulthood BMI | node_pheno52 | GTExv8.EUR.Brain_Frontal_Cortex_BA9 | RP11-820I16.1 | 4.74E-02 | 1.32E-01 | 3.00E-03 |  | 1.84E-02 | 4.23E-01 | 3.40E-02 |  | 7.02E-03 |
| adulthood BMI | node_pheno52 | GTExv8.EUR.Brain_Frontal_Cortex_BA9 | RBAK | 3.93E-02 | 1.15E-01 | 3.00E-03 |  | 2.25E-02 | 4.32E-01 | 1.38E-01 |  | 7.10E-03 |
| adulthood BMI | node_pheno52 | GTExv8.EUR.Brain_Frontal_Cortex_BA9 | MRPS10 | 2.50E-02 | 8.19E-02 | 1.90E-02 |  | 3.93E-02 | 5.31E-01 | 8.00E-03 |  | 7.79E-03 |
| adulthood BMI | node_pheno52 | GTExv8.EUR.Brain_Frontal_Cortex_BA9 | LINC01933 | 3.60E-02 | 1.07E-01 | 5.00E-03 |  | 3.09E-02 | 5.09E-01 | 2.00E-03 |  | 8.68E-03 |
| adulthood BMI | node_pheno52 | GTExv8.EUR.Brain_Frontal_Cortex_BA9 | ASTN1 | 3.90E-02 | 1.15E-01 | 3.00E-03 |  | 2.92E-02 | 4.93E-01 | 3.40E-02 |  | 8.86E-03 |
| adulthood BMI | node_pheno52 | GTExv8.EUR.Brain_Frontal_Cortex_BA9 | WDHD1 | 3.98E-02 | 1.16E-01 | 1.40E-02 |  | 3.64E-02 | 5.17E-01 | 3.00E-03 |  | 1.09E-02 |
| adulthood BMI | node_pheno52 | GTExv8.EUR.Brain_Frontal_Cortex_BA9 | ZNF180 | 4.30E-02 | 1.23E-01 | 0.00E+00 |  | 3.48E-02 | 5.12E-01 | 7.00E-03 |  | 1.12E-02 |
| adulthood BMI | node_pheno52 | GTExv8.EUR.Brain_Frontal_Cortex_BA9 | PDZD8 | 4.75E-02 | 1.32E-01 | 3.30E-02 |  | 3.23E-02 | 5.11E-01 | 8.00E-03 |  | 1.15E-02 |
| adulthood BMI | node_pheno52 | GTExv8.EUR.Brain_Frontal_Cortex_BA9 | RP11-73M18.8 | 4.37E-02 | 1.25E-01 | 4.50E-02 |  | 4.24E-02 | 5.53E-01 | 2.20E-02 |  | 1.35E-02 |
| adulthood BMI | node_pheno52 | GTExv8.EUR.Brain_Frontal_Cortex_BA9 | AHSA2 | 4.03E-02 | 1.17E-01 | 2.90E-01 |  | 4.88E-02 | 5.71E-01 | 3.00E-02 |  | 1.42E-02 |
| adulthood BMI | node_pheno52 | GTExv8.EUR.Brain_Frontal_Cortex_BA9 | AC004448.5 | 4.77E-02 | 1.33E-01 | 8.00E-03 |  | 4.64E-02 | 5.67E-01 | 7.10E-02 |  | 1.57E-02 |
